# Impact of Nonpharmaceutical Interventions during the COVID-19 Pandemic on the Prevalence of Respiratory Syncytial Virus in Hospitalized Children with Lower Respiratory Tract Infections: A Systematic Review and Meta-Analysis

**DOI:** 10.3390/v16030429

**Published:** 2024-03-11

**Authors:** José J. Leija-Martínez, Luis A. Esparza-Miranda, Gerardo Rivera-Alfaro, Daniel E. Noyola

**Affiliations:** 1 Research Center in Health Sciences and Biomedicine (CICSaB), Universidad Autónoma de San Luis Potosí, San Luis Potosí 78210, Mexico; jesus.leija@uaslp.mx (J.J.L.-M.); a250764@alumnos.uaslp.mx (L.A.E.-M.); a238871@alumnos.uaslp.mx (G.R.-A.); 2 Microbiology Department, Facultad de Medicina, Universidad Autónoma de San Luis Potosí, San Luis Potosí 78210, Mexico

**Keywords:** respiratory syncytial virus, nonpharmaceutical interventions, lockdown, COVID-19, SARS-CoV-2, respiratory tract infection

## Abstract

During the COVID-19 pandemic, nonpharmaceutical interventions (NPIs) were implemented in order to control the transmission of SARS-CoV-2, potentially affecting the prevalence of respiratory syncytial virus (RSV). This review evaluated the impact of NPIs on RSV-related hospitalizations in children during the lockdown (2020–2021) compared to the pre-pandemic (2015–2020) and post-lockdown (2021–2022) periods. In this systematic review and meta-analysis, we searched through PubMed, Scopus, and Web of Science for studies published in English between 1 January 2015 and 31 December 2022. Additionally, we conducted hand searches of other records published between 1 January 2023 and 22 January 2024. Our target population was hospitalized children aged 0–18 years with RSV-related lower respiratory tract infections confirmed through immunofluorescence, antigen testing, or molecular assays. We focused on peer-reviewed observational studies, analyzing the primary outcome of pooled RSV prevalence. A generalized linear mixed model with a random-effects model was utilized to pool each RSV prevalence. Heterogeneity was assessed using Cochran’s Q and *I*^2^ statistics, while publication bias was evaluated through funnel plots and Egger’s tests. We identified and analyzed 5815 publications and included 112 studies with 308,985 participants. Notably, RSV prevalence was significantly lower during the lockdown period (5.03% [95% CI: 2.67; 9.28]) than during the pre-pandemic period (25.60% [95% CI: 22.57; 28.88], *p* < 0.0001). However, RSV prevalence increased notably in the post-lockdown period after the relaxation of COVID-19 prevention measures (42.02% [95% CI: 31.49; 53.33] vs. 5.03% [95% CI: 2.67; 9.28], *p* < 0.0001). Most pooled effect estimates exhibited significant heterogeneity (*I*^2^: 91.2% to 99.3%). Our findings emphasize the effectiveness of NPIs in reducing RSV transmission. NPIs should be considered significant public health measures to address RSV outbreaks.

## 1. Introduction

Respiratory syncytial virus (RSV) is the primary cause of early childhood lower respiratory tract infections (LRTIs), leading to severe illness and high mortality rates [1]. In 2019, RSV resulted in 33 million LRTI episodes, 3.6 million hospitalizations, and 101,400 deaths among children aged 0–60 months [1]. This virus exhibits seasonal transmission patterns, with epidemic peaks occurring in autumn and winter in temperate climates [2,3].

The declaration of coronavirus disease 2019 (COVID-19) as a global public health emergency by the World Health Organization (WHO) prompted the adoption of nonpharmaceutical interventions (NPIs) aimed at mitigating the transmission of severe acute respiratory syndrome coronavirus 2 (SARS-CoV-2) [4]. These interventions likely altered the seasonality patterns of RSV and profoundly impacted its prevalence among hospitalized children with LRTIs, particularly during the 2020–2021 season [5,6,7].

Ravkin et al. [7] utilized Google Trends search volumes as an indicator of viral circulation and observed a disruption between the peak latency and magnitude of RSV during the pandemic. This observed phenomenon could be attributed to NPIs, emphasizing the significant impact of population mobility on RSV incidence. Multiple countries have reported a substantial decrease in RSV-related LRTI hospitalizations, with some indicating a 90–99% reduction [5,6]. For instance, in England, Bardsley et al. [5] reported a dramatic fall in RSV test positivity through PCR of 99.6% among children under five years old, as documented by the Respiratory DataMart System (RDS). Remarkably, in Italy, Pruccoli et al. [6] reported only three cases of RSV-related hospital admissions among children under three years old across fifteen pediatric hospitals. As a result, the 2020–2021 RSV season presented a real-world opportunity to assess the effectiveness of NPIs in reducing RSV transmission.

Due to the similarities in transmission mechanisms between RSV and SARS-CoV-2, the global implementation of NPIs resulted in a decline in RSV infections [8]. However, following the relaxation of COVID-19 restrictions, RSV seasonality patterns and outbreaks have returned to pre-pandemic levels or even increased [5,9,10,11,12].

Presumably, NPIs may have influenced RSV-related hospitalizations, underscoring the importance of comprehensively examining their effects. To address this concern, this systematic review aimed to assess the impact of NPIs on the prevalence of RSV among hospitalized children with LRTIs during the early pandemic (lockdown) period (2020–2021) in comparison to the pre-pandemic (2015–2020) and post-lockdown periods (2021–2022). Of note, because winter seasons start in the second half of a given year and end during the first half of a given year, the periods appear to include a “repeated” year, but cases were assigned only to one period, based on the months of the year.

## 2. Materials and Methods

### 2.1. Search Strategy and Selection Criteria

The systematic review protocol was registered on the International Prospective Register of Systematic Reviews (PROSPERO) with the registration number CRD42022376951 and adhered to the Preferred Reporting Items for Systematic Reviews and Meta-Analyses (PRISMA) statement, published in 2020 [13,14]. For detailed information, please refer to Table S1 in the Supplementary Material SA for the PRISMA 2020 Checklist. The PICOS strategy was used to establish the eligibility criteria (population, intervention, comparison, outcome, and study design) [15]. Our research question was as follows: “What is the impact of NPIs during the COVID-19 pandemic on the prevalence of RSV-related LRTI hospitalizations in children?”

This study examined children aged 0–18 years hospitalized with RSV-related LRTIs, excluding nosocomial infections. RSV diagnosis was confirmed using immunofluorescence, antigen testing, or molecular assays. The prevalence of RSV hospitalizations was assessed for each winter season; studies in which pooled data from several seasons were reported were excluded. Peer-reviewed observational studies (cohort, case–control, and cross-sectional) were considered, with an RSV season defined as a six-month local epidemic. Case reports, reviews, editorials, and duplicates were excluded.

A comprehensive search through the PubMed, Scopus, and Web of Science databases was conducted to identify relevant articles published between 1 January 2015 and 31 December 2022. Additionally, other methods involving hand searching were carried out for articles published from 1 January 2023 to 22 January 2024. We exclusively incorporated studies conducted in the English language. Table S2 in the Supplementary Material SA includes the search terms employed for each database. Additionally, we thoroughly examined the bibliographies of pertinent research articles. The study selection process involved LAEM and JJLM independently screening records, reviewing full reports, and discussing articles. A third reviewer (DEN) made the final decision if disagreements arose. Pilot screening refined the process before the actual screening.

Two reviewers (LAEM and GRA) extracted data using Microsoft^®^ Excel 365 spreadsheets, conducting pilot extraction to ensure consistency. Cross-checking was conducted to ensure accurate extraction, and a final spreadsheet was obtained. A third reviewer (DEN) resolved any disagreements. Researchers emailed the corresponding author if additional data were needed for inclusion or clarification. The following items were collected: RSV prevalence, period, seasons, WHO region, age, sex, study design, timing of data collection, diagnostic technique, and sample type.

The primary outcome was the pooled RSV prevalence in hospitalized children with LRTIs across the pre-pandemic, lockdown, and post-lockdown periods. As a secondary outcome, we examined the prevalence of intensive care unit (ICU) admissions and deaths among children with LRTIs related to RSV during these three periods. The tool developed by Hoy et al. [16] for prevalence studies was employed to assess the risk of bias, categorizing it as low (8–10), moderate (5–7), or high (0–4) (Table S3 in Supplementary Material SA). The reviewers LAEM and JJLM independently analyzed and cross-checked bias assessments, resolving any disagreements through discussion with a third reviewer (DEN). The quality of evidence was assessed using the AMSTAR 2 instrument (A MeaSurement Tool to Assess systematic Reviews 2) [17].

### 2.2. Data Analysis

We summarized the study characteristics in tables and narrative synthesis. We determined the proportion of RSV-related LRTI cases by dividing the number of RSV-positive samples by the total number of samples tested. Then, we transformed the proportion into a prevalence by multiplying it by 100.

Our meta-analysis aimed to estimate and report the pooled prevalence with a 95% confidence interval (CI). We observed a wide prevalence range, varying from nearly 0% to 100%. This wide range could lead to overestimating precision due to significant variance. To address this issue, we performed a generalized linear mixed model using a maximum-likelihood estimator for τ^2^ and a random-effects model to pool each RSV prevalence [18]. Finally, the results were presented through forest plots, tables, and narratives to display the pooled effect estimates.

Heterogeneity was assessed visually through forest plots and using Cochran’s Q and *I*^2^ statistics. Cochran’s Q with *p* < 0.1 indicated significant heterogeneity among studies [15,19]. The *I*^2^ statistic categorized heterogeneity as follows: (a) <25%: low heterogeneity; (b) 25–50%: moderate heterogeneity; (c) ≥50%: high heterogeneity [15,19]. A random effects model was used due to the observed high heterogeneity. Funnel plots and Egger’s tests assessed potential publication bias, with a *p*-value < 0.1 being considered statistically significant. Analyses were performed using the RStudio (R version 4.2.3) “meta” package (version 6.2-1), with a *p*-value < 0.05 indicating statistical significance.

Subgroup, sensitivity, and meta-regression analyses explored sources of heterogeneity based on study and participant characteristics: period, seasons, WHO region, age, study design, timing of data collection, risk of bias, diagnostic technique, and sample type.

Because of the great variety of diagnostic techniques used, we grouped antigen tests and direct immunofluorescence into a single category called immune assays. Additionally, all types of PCR, including multiplex PCR, multiplex RT–PCR, PCR, qPCR, RT–PCR, or RT–qPCR, were grouped into a single category known as molecular assays.

Sensitivity analyses included only studies using molecular assays for RSV diagnosis. Furthermore, we conducted a stratified analysis based on age strata (<2, <5, <10, <15, and <18 years), WHO regions, and countries to compare the pandemic (lockdown, 2020–2021), pre-pandemic (2015–2020), and post-lockdown (2021–2022) periods. A bubble plot illustrates the relationship between RSV prevalence and the study participants’ average age (in months). In the meta-regression model, the presence of residual heterogeneity in RSV prevalence was suggested by the explained heterogeneity (*R^2^*).

## 3. Results

### 3.1. Search Results

**Figure 1:**
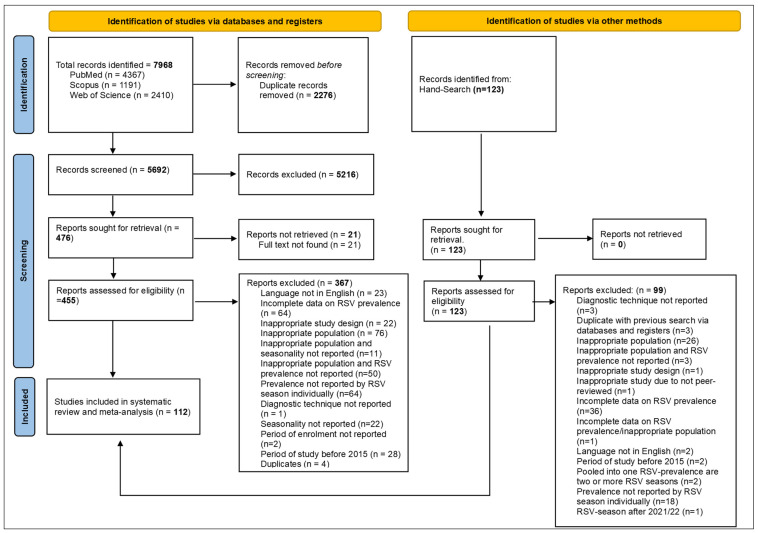
Study selection.

A total of 8091 records were identified from the PubMed (n = 4367), Scopus (n = 1191), and Web of Science (n = 2410) databases, along with hand searching (n = 123). After removing duplicate records (n = 2276), we screened 5815 unique records (Figure 1). Among these, we assessed 578 full reports for eligibility, excluding 466 reports due to them not meeting the inclusion criteria. Ultimately, we included 112 studies in this systematic review and meta-analysis. For the list of the 466 full reports that were excluded and the reasons for their exclusion, please see Supplementary Material SB.

### 3.2. Characteristics of the Included Articles

As illustrated in Table 1, most studies in this review focused on children under two years of age (35/112; 31.3%). All participants were recruited between January 2015 and December 2022. Cross-sectional studies were more common (75/112; 67.0%), and most employed a prospective recruitment strategy (58/112; 51.8%). Notably, the most commonly used sampling method was consecutive sampling (110/112; 98.2%), and most studies exhibited a low risk of bias (71/112; 63.4%). For a comprehensive analysis of the risk of bias in each study, please refer to Tables S3 and S4 in the Supplementary Material SA.

Across all six WHO regions, our review encompassed studies primarily reported from the European region (47/112; 42.0%), followed by the Western Pacific region (34/112; 30.4%). It is worth noting that these two regions accounted for 72.3% (81/112) of all included studies. Furthermore, among the 37 countries that provided the 112 studies in this review, a large proportion of studies were conducted in China (27/112; 24.1%) and Italy (20/112; 17.9%) (Table S5 in the Supplementary Material SA). Molecular assays, particularly RT–PCR and nasopharyngeal secretions, were the most frequently utilized diagnostic technique and type of sample, respectively (31/112; 27.7% and 62/112; 55.4%, respectively). For a comprehensive analysis of each study’s characteristics with detailed references, please refer to Tables S6 and S7 in the Supplementary Material SA.

The 112 included studies provide 221 RSV prevalences, which varied significantly across all studies, ranging from 0% to 82.24%. During the lockdown period, the proportions ranged from 0% to 77.78%. Notably, ten studies reported proportions of 0%, while two studies observed no occurrences of RSV-positive tests or hospitalizations for LRTIs during this lockdown period [6,20,21,22,23,24,25]. Of note, in some studies, such as the report by Stera et al. [25] and Pruccoli et al. [6], the numerator and denominator were zero during the 2020/21 season, making it mathematically implausible to calculate a proportion; therefore, they were excluded from the meta-analysis. The proportion ranges in the pre-pandemic and post-lockdown periods were 4.65% to 79.31% and 4.76% to 82.24%, respectively. Please refer to Figure S1 in the Supplementary Material SA for a comprehensive breakdown of the 221 RSV prevalences based on each study and RSV season.

**Figure 2:**
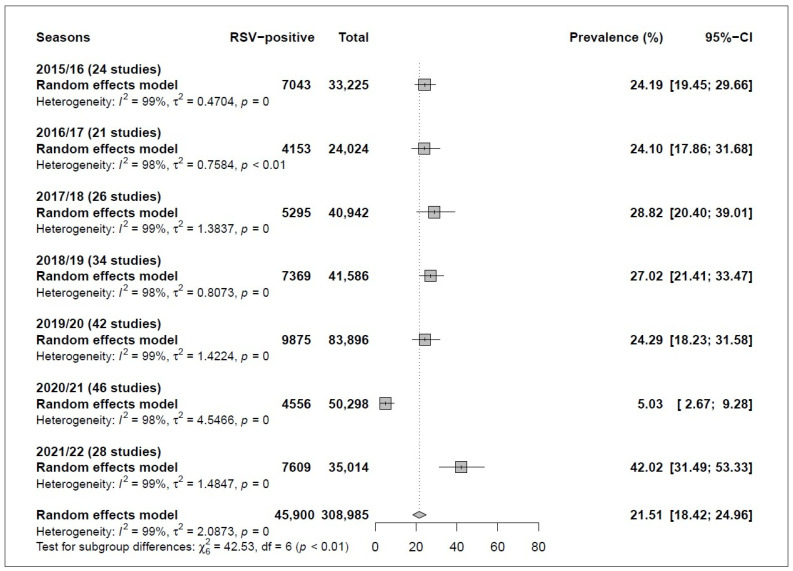
Respiratory syncytial virus (RSV) prevalences according to RSV season. For all 221 prevalences and their respective references, please refer to Figure S1 and the reference section at the end of the Supplementary Material SA (pages 39–59).

This review incorporated 112 studies encompassing 308,985 participants from 37 countries, yielding a total of 221 prevalences. The overall pooled prevalence of RSV was 21.51% [95% CI: 18.42; 24.96]. Notably, there was significant heterogeneity in the effect size (Q-value = 22,893.65, *I^2^* = 99.0%, *p* < 0.0001) (Figure 2 and Figure S1 in the Supplementary Material SA).

### 3.3. Subgroup Analysis

Regarding the subgroup analysis, the prevalence of RSV was significantly lower during the lockdown period than during the pre-pandemic period (5.03% [95% CI: 2.67; 9.28] vs. 25.6% [95% CI: 22.57; 28.88], *p* < 0.0001). Interestingly, the prevalence of RSV increased in the post-lockdown period after relaxing COVID-19 mitigation measures compared to the lockdown period (42.02% [95% CI: 31.49; 53.33] vs. 5.03% [95% CI: 2.67; 9.28], *p* < 0.0001). A detailed subgroup analysis is provided in Table 2.

Comparisons of RSV prevalence among pre-pandemic seasons (2015–2016 through 2019–2020) showed no difference (*p* = 0.8651). However, when each pre-pandemic season was compared individually with the lockdown period (2020–2021) and the post-lockdown season (2021–2022), similar results were observed as when all pre-pandemic seasons were compared as a single group: a lower prevalence during the lockdown period and a higher prevalence during the post-lockdown period (*p* < 0.0001) (Table 2 and Figure 2).

Overall, the European region exhibited a higher RSV prevalence than the other WHO regions (*p* = 0.0004), and children under two years of age had a higher prevalence than the other age groups (*p* < 0.0001). On the other hand, there was no significant difference in RSV prevalence between studies with low and moderate risk of bias (*p* = 0.1453) (Table 2). Noticeably, studies using molecular assays demonstrated a significantly higher prevalence than those based on immune assays (24.96% [95% CI: 20.77; 29.69] vs. 13.94% [95% CI: 10.65; 18.04], *p* = 0.0007). Specifically, the qPCR test showed the highest RSV prevalence among all diagnostic techniques (*p* = 0.0016). Moreover, nasopharyngeal secretions and sputum specimens were associated with a higher RSV prevalence than other specimen types (*p* = 0.0023) (Table 2).

**Figure 3:**
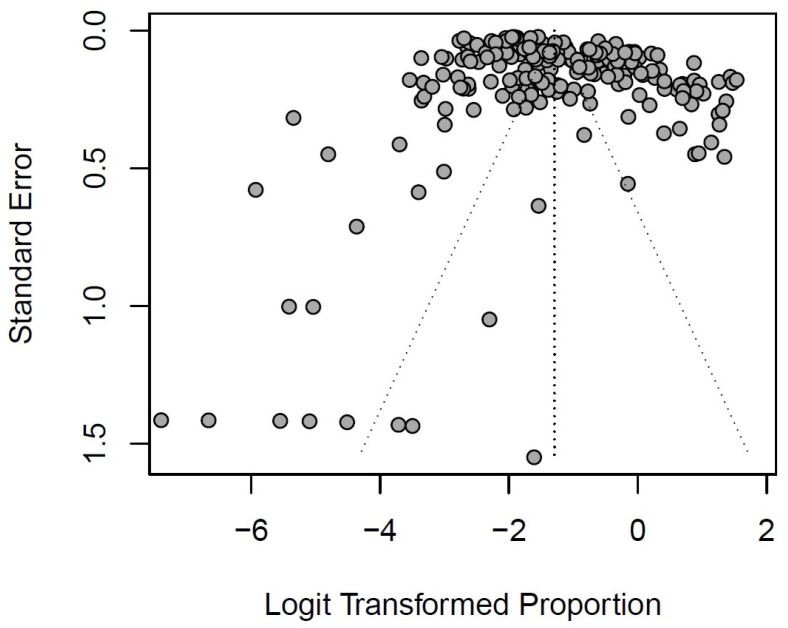
Funnel plot of overall pooled prevalence of respiratory syncytial virus in lower respiratory tract infections.

Most pooled effect estimates exhibit substantial heterogeneity, with *I*^2^ statistics ranging from 91.2% to 99.3%. Publication bias was detected for the overall pooled prevalence of RSV, as indicated by the funnel plot and corroborated through Egger’s test (*p* < 0.0001) (Table 2 and Figure 3).

Nevertheless, it is worth highlighting that there was no publication bias during the lockdown period, as indicated by Egger’s test (*p* = 0.5569) (Table 2). It is also important to note that most subgroup analyses found publication bias when analyzed using Egger’s test, except for the type of assay and diagnostic technique.

### 3.4. Sensitivity Analysis

We conducted a sensitivity analysis explicitly focusing on the impact of molecular assay diagnostic techniques on RSV proportions. The overall pooled prevalence using molecular assays was 24.96% [95% CI: 20.77; 29.69], which showed a 3.46% increase compared to the meta-analysis that included all diagnostic techniques. Similar to the initial meta-analysis, evidence of publication bias was found, demonstrated by the asymmetry in the funnel plot and corroborated by Egger’s test (*p* = 0.0350) (Table S8 and Figure S2 in the Supplementary Material SA). Nonetheless, this sensitivity analysis reported no publication bias during the lockdown (2020/21) period based on Egger’s test (*p* = 0.2778). The pooled RSV prevalence during the lockdown period was significantly lower than that in the pre-pandemic period (3.82% [95% CI: 1.53; 9.22] vs. 30.45% [95% CI: 26.68; 34.49], *p* < 0.0001). Interestingly, there was a significant increase in the pooled RSV prevalence during the post-lockdown period compared to the lockdown period (44.29% [95% CI: 32.61; 56.63] vs. 3.82% [95% CI: 1.53; 9.22], *p* < 0.0001) (Table S8 in Supplementary Material SA). Remarkably, the findings from the sensitivity analysis regarding RSV prevalence across different periods and seasons align with those of the initial meta-analysis.

### 3.5. Stratified Analysis by World Health Organizations Regions, Countries, and Age Stratum

As shown in Table 1, the vast majority of WHO regions and countries in this review were from the Western Pacific and Europe, with China and Italy representing their respective regions. Additionally, a high prevalence of RSV was observed in Europe, as reported in subgroup and meta-regression model analyses (Table 2 and Table S9 in the Supplementary Material SA).

In Europe, the impact of NPIs was evident; during the lockdown period, the pooled prevalence of RSV was significantly lower than during the pre-pandemic period (4.89% [95% CI: 1.68; 13.39] vs. 34.81% [95% CI: 29.47; 40.56], *p* < 0.0001). In contrast, there was a notable upward trend in RSV prevalence in the post-lockdown period after relaxing COVID-19 mitigation measures compared to the lockdown period (55.18% [95% CI: 42.96; 66.80] vs. 4.89% [95% CI: 1.68; 13.39], *p* < 0.0001) (Table 3).

Similar to the European region, Italy exhibited a low RSV prevalence during the lockdown period compared to the pre-pandemic period (5.93% [95% CI: 0.89; 30.68] vs. 51.87% [95% CI: 43.11; 60.51], *p* = 0.0024). After relaxing COVID-19 measures, Italy experienced a significant upward trend in RSV prevalence (63.59% [95% CI: 53.25; 72.81] vs. 5.93% [95% CI: 0.89; 30.68], *p* = 0.0024) (Table 3). On the other hand, though less pronounced, the Western Pacific region and China demonstrated similar trends in RSV prevalences during the three periods, showing a decline in the lockdown period and an increase in the post-lockdown period (*p* = 0.0002) (Table 3). It is worth underscoring that we were unable to perform these meta-analyses for the remaining WHO regions and countries due to the lack of enough studies for all three periods.

Moreover, as evident from subgroup and meta-regression model analyses, age plays a crucial role in RSV prevalence, especially among infants and toddlers under two years of age (Table 2 and Table S9 in the Supplementary Material SA). The effect of NPIs was more significant in the stratum under two years old. RSV prevalence during the lockdown period was lower than in the pre-pandemic period (6.46% [95% CI: 1.19; 28.29] vs. 47.82% [95% CI: 42.06; 53.65], *p* < 0.0001) (Table 4). Meanwhile, a major increase in RSV prevalence was observed in the post-lockdown period (67.61% [95% CI: 57.01; 76.67] vs. 6.46% [95% CI: 1.19; 28.29], *p* = 0.0024). The rest of the age strata showed similar trends in RSV prevalence during the pre-pandemic, lockdown, and post-lockdown periods as observed in the under two years old stratum; however, this was less marked (Table 4).

### 3.6. Meta-Regression Model Analysis

Similar to subgroup analysis, the meta-regression model confirms that the prevalence of RSV during the lockdown period was lower than in the pre-pandemic period, as observed in the subgroup analysis (coefficient = 0.1526 [95% CI: 0.0856, 0.2195], *p* < 0.0001, R^2^ = 16.47%, with the lockdown period as a reference) (Table S9 in the Supplementary Material SA). On the other hand, this analysis indicates that RSV prevalence increased in the post-lockdown period, as also reported in the subgroup analysis (coefficient = 0.3005 [95% CI: 0.2059, 0.3951], *p* = 0.0001, R^2^ = 16.47%, with the lockdown period as a reference) (Table S9 in the Supplementary Material SA).

Furthermore, the meta-regression analysis confirms that age and diagnostic technique significantly impact the prevalence of RSV. Age, especially among children under two years old, is a crucial factor influencing the magnitude of this effect. The bubble plot and meta-regression model further demonstrate a statistically significant negative relationship between RSV prevalence and age in months (coefficient = −0.0079 [−0.0105, −0.0054], *p* < 0.0001, R^2^ = 22.29%) (Table S9 and Figure S3 in Supplementary Material SA). Additionally, the pooled prevalence of RSV remains unaffected by the risk of bias (coefficient −0.0554 [−0.1157, 0.0049], *p* = 0.0716, R^2^ = 1.58%, with low risk as a reference), consistent with our subgroup analysis. Based on the AMSTAR 2 [17] criteria for assessing the quality of the body of evidence, the study meets all critical domains (items 2, 4, 7, 9, 11, 13, and 15), indicating a high-quality review (Table S10 in Supplementary Material SA).

### 3.7. Analysis of Intensive Care Unit Admissions and Mortality

We also analyzed the frequency of ICU admission and case fatality for children admitted with RSV infection. Most studies did not provide detailed information regarding these outcomes to allow assessment of the number of children with RSV infection who required admission to the ICU or who died. Although we had limited data on the prevalence of these outcomes, we conducted a meta-analysis using our dataset, which included 10 and 19 studies for ICU admission and RSV mortality, respectively. The analyses showed no significant differences in the prevalence of ICU admissions between the pre-pandemic and the lockdown periods (8.97% [95% CI: 2.60; 26.71] vs. 1.09% [95% CI: 0.15; 7.31], *p* = 0.07). The post-lockdown period was not included in these analyses due to the scarcity of data. Similarly, the comparison of mortality rates between the pre-pandemic, lockdown, and post-lockdown periods showed no significant difference (0.13% [95% CI: 0.01; 1.15], 3.57% [95% CI: 0.50; 21.42], and 0.0% [95% CI: 0.00; 100.00], respectively, *p* = 0.09) (Figures S4 and S5 in the Supplementary Material SA).

## 4. Discussion

This systematic review and meta-analysis aimed to assess the impact of NPIs during the COVID-19 pandemic on the prevalence of RSV in hospitalized children with LRTIs. Our comprehensive analysis of a large dataset provides valuable insights into the evolving prevalence of RSV across various pandemic phases and associated interventions.

Remarkably, our observations are similar to those of the meta-analysis conducted by Regassa et al. [26], which studied RSV prevalence in hospitalized children with LRTIs in Africa and reported a pooled prevalence of 23% [95% CI: 20.0; 25.0]. Similarly, a systematic review conducted by Pratt et al. [27] in hospitalized children with community-acquired pneumonia covering the pre-pandemic era showed an RSV pooled prevalence of 22.7% [95% CI: 20.9; 24.5], closely aligning with our results for the same period.

In most of our analyses, we confirmed a significant finding: a notable reduction in the prevalence of RSV during the lockdown period compared to the pre-pandemic period. This observation suggests that implementing NPIs such as social distancing, face masks, lockdowns, handwashing, and school closures to control the spread of SARS-CoV-2 had the unintended positive effect of effectively reducing the transmission of RSV.

The results of our study are consistent with previous research that suggests a decline in RSV-related hospitalizations during the COVID-19 pandemic [5,6]. For instance, Bardsley et al. [5], in an extensive study conducted in England focusing on children under five years of age and utilizing public health surveillance systems, reported an 80.8% decrease [95% CI: −80.9; −80.8] in admissions for RSV-attributable respiratory diseases from 2015–2019 to 2020–2021.

During the pandemic, similar trends were observed for other respiratory pathogens, demonstrating the efficacy of NPIs in controlling respiratory infections [28,29]. The substantial decline in positive tests for RSV and hospitalizations for LRTIs during the lockdown period can be attributed to reduced mobility and social interactions, which limited opportunities for RSV to spread among vulnerable children [7].

Interestingly, our review revealed a rebound effect as COVID-19 restrictions were eased. The prevalence of RSV in the post-lockdown period significantly increased compared to that in the lockdown period, and the proportion of RSV-associated hospitalizations surpassed that observed in the pre-pandemic period. This phenomenon was observed in various studies and countries, particularly during the summer of 2021 [5,9,10,11,12]. For example, Bardsley et al. [5] reported a staggering increase of 1258.3% [95% CI: 1178.3; 1345.8] in RSV cases in England. This increase could be attributed to the relaxation of NPIs, increased mobility, enhanced social interactions, and the subsequent spread of RSV [7].

Additionally, the population may have experienced a temporary decrease in immunity against RSV due to reduced exposure during the lockdown, a phenomenon known as “immunity debt”, which could have rendered them more susceptible when the virus started circulating again [30,31,32,33]. Importantly, in Canada, Reicherz et al. [32] documented a significant reduction in prefusion RSV F IgG levels in women of childbearing age in 2021 compared to 2020 (148,858 ± 2.4 vs. 197,806 ± 2.2 AU/mL; *p* = 0.0232). Similarly, RSV-neutralizing titers in women decreased by 12-fold in 2021 compared with 2020 (10.3 ± 2.0 vs. 120.9 ± 2.9; *p* < 0.0001). Likewise, infants sampled in 2021 exhibited approximately 15-fold lower prefusion RSV F IgG levels (4258 ± 8.8 vs. 63,530 ± 4.4 AU/mL; *p* < 0.0001) and 3.4-fold lower RSV neutralizing titers (6.7 ± 1.8 vs. 22.8 ± 2.0; *p* < 0.0001) than infants sampled in 2020. In the Netherlands, den Hartog et al. [33] also found similar findings in a population of all ages (1–89 years). They observed that postfusion F RSV-specific IgG antibody concentrations declined from 2020 to 2021 (*p* < 0.001). These findings suggest a potential waning immunity that may have contributed to the global resurgence of RSV during interseasonal periods.

Another plausible explanation for the resurgence of RSV could be viral interference, whereby the high prevalence of SARS-CoV-2 during the lockdown period suppressed the circulation of RSV and other respiratory viruses [34]. As COVID-19 cases declined and NPIs were relaxed, RSV found a susceptible population and rapidly started spreading again.

Other proposed hypotheses regarding the change in RSV epidemiology during the pandemic include immune dysregulation due to SARS-CoV-2 infection, enhanced RSV virulence, and behavioral modifications in RSV testing practices among healthcare workers during the lockdown [35]. However, these proposed mechanisms require further in-depth study.

An important issue is to assess whether the low numbers of RSV infections documented during the lockdown period are the result of a reduction in RSV prevalence or a decrease in testing as a result of prioritizing SARS-CoV-2 detection, as healthcare resources were redirected towards managing COVID-19 during the pandemic [35]. Although a reduction in non-SARS-CoV-2 testing occurred in some countries, the available data suggest that the reduced proportion of RSV detections during the lockdown period was a result of reduced circulation of this virus. For example, a study carried out in Germany reported that during the period between December 2020 and March 2021, testing for four viruses (influenza A, influenza B, RSV, and SARS-CoV-2) was routinely used in an emergency department; during that period, 4915 tests were carried out and none were positive for influenza A, influenza B or RSV, despite the fact that the number of tests represented a five-fold increase compared to pre-pandemic figures [36]. Groves et al. analyzed the number of RSV tests carried out at sentinel laboratories in Canada during the 2020/2021 season and compared this with pre-pandemic testing; the weekly number of RSV tests during the 2020/2021 season (8890) was higher than the weekly average number of tests before the pandemic (6207), although the proportion of positive results was notably lower [37]. As shown in the abovementioned studies, even in countries where RSV testing was maintained or increased during the lockdown period, the number and proportion of RSV-positive tests were reduced.

RSV resurgence during the post-lockdown period highlights the importance of balancing the relaxation of NPIs and continuous viral monitoring with early interventions to manage the health impacts associated with outbreaks of respiratory infections. Future policies should consider a gradual easing of restrictions and ongoing monitoring to prevent sudden surges, particularly during respiratory virus seasons.

Our subgroup and meta-regression analyses identified age as a critical factor influencing the prevalence of RSV, with children under two years of age exhibiting the highest prevalence. This negative relationship between RSV prevalence and age underscores the vulnerability of young children to RSV infections. This observation is consistent with the understanding that young children, particularly infants and toddlers, are more susceptible to RSV due to their immature immune systems and narrower airways [38,39]. The higher prevalence of RSV among younger children highlights the need for targeted preventive strategies for this vulnerable population, particularly in post-lockdown scenarios.

The geographic emphasis of the study data suggests that the European region exhibited a higher RSV prevalence than other WHO regions. This regional difference in prevalence may be attributed to factors such as population density, climate, the level of healthcare surveillance, and adherence to NPIs [38,39]. Further studies are warranted to fully understand the regional variations in RSV prevalence during and after the pandemic.

In our review, determining which specific NPIs were more effective in mitigating the spread of SARS-CoV-2 and, consequently, RSV for each WHO region and age group was challenging. Factors such as pandemic severity, demographics, and local policies can influence their effectiveness, complicating the ability to carry out a comprehensive overview. In this context, Billard et al. [40] analyzed RSV surveillance data from 11 countries, focusing on the impact of nine NPIs from 2020 to 2021. They concluded that school closures, workplace closures, and stay-at-home measures were the most effective in reducing RSV spread. Similarly, Liu et al. [41] assessed the impact of NPIs on SARS-CoV-2 spread across over 130 countries using panel regression analysis and hierarchical cluster analysis, finding that school closures and restrictions on internal movement were consistently effective in all models. Additionally, Suryanarayanan et al. [42] used the Worldwide Non-pharmaceutical Interventions Tracker for COVID-19 (WNTRAC) to compile data on NPIs carried out in 261 countries and territories. WNTRAC showed that entertainment/cultural section closure (24.1%), confinement (15.0%), and school closures (13.9%) were the most common measures, aligning with the findings of Billard et al. [40] and Liu et al. [41]. These results suggest that school closures, workplace and entertainment closures, and confinement (stay-at-home) measures contributed to the reduction in the number of SARS-CoV-2 and RSV cases in most regions and countries.

Diagnostic techniques played a significant role in determining RSV prevalence estimates. Our study revealed that molecular assays, particularly qPCR, were associated with a higher RSV prevalence than immune assays. This finding is not unexpected, as molecular assays have higher sensitivity and specificity in detecting viral RNA [43,44]. Moreover, the use of nasopharyngeal secretions and sputum specimens was linked to a higher prevalence of RSV compared to other sample types, possibly due to the higher viral load present in the nasopharynx [43,44]. These findings are consistent with a systematic review by Regassa et al. [26] involving African children, which supports the importance of sample types in estimating RSV prevalence. This observation underscores the significance of diagnostic techniques in accurately assessing RSV prevalence and suggests that molecular assays could be more reliable for surveillance and clinical management.

In this review, the overall quality of evidence evaluated using the AMSTAR 2 [17] criteria was high. However, a critical limitation of this study was the presence of publication bias. This may be because most study were from the WHO regions of Europe and the Western Pacific, and only published studies were included. Arguably, this may limit the generalizability of our findings to more densely populated areas such as Southeast Asia, parts of the Americas, and sub-Saharan Africa. This concern could affect the validity of the conclusions drawn from the included studies, as the available data may not have been fully captured. Nevertheless, most of our meta-analyses focusing on the lockdown period (2020/21) showed no publication bias, suggesting more robust and reliable results within this subgroup. To comprehensively assess RSV prevalence during the COVID-19 pandemic, future studies should address publication bias and consider including unpublished studies with a more balanced distribution across WHO regions.

Additionally, significant heterogeneity was observed among the included studies in the meta-analysis, which can affect the precision of the pooled estimates. Subgroup and meta-regression analyses revealed several factors contributing to RSV prevalence heterogeneity, including age range, geographic region, sample type, and diagnostic techniques. Although random-effects models accounted for this heterogeneity, it is vital to interpret the findings cautiously. Our data demonstrated significant similarity to five systematic reviews and meta-analyses that focused on the prevalence of RSV in hospitalized children with LRTIs [26,27,45,46,47]. These studies also reported high heterogeneity, with *I*^2^ values ranging from 90% to 99%, consistent with our findings.

Concerning the severity of RSV hospitalizations, our findings regarding ICU admissions and mortality are limited because few studies reported these outcomes in detail. No differences in ICU admissions and mortality were identified between the pre-pandemic, lockdown, and post-lockdown periods. Similarly, Nygaard et al. [48] analyzed a large dataset based on the Danish National Patient Registry. They reported that there were no differences in the use of mechanical ventilation in RSV-related hospitalizations in children (0–17 years) between the pre-pandemic and post-lockdown periods. It is important to underscore that our observations regarding this issue are limited. Thus, our results should be considered cautiously. This knowledge gap suggests that it is essential to study this topic in further detail in future research.

Finally, NPIs significantly reduced RSV cases; however, researchers have raised concerns about children’s health in relation to NPIs, particularly regarding mental health issues such as anxiety and depression linked to quarantine and school closures. These measures also disrupt healthcare access, impacting the management of non-communicable diseases. Careful evaluation of the benefits and drawbacks of NPIs is crucial. In severe pandemics, where specific treatments or vaccines are unavailable, NPIs may be the only viable option. This necessitates policymakers to balance the benefits and risks of NPIs to ensure global well-being [49].

## 5. Conclusions

In summary, this systematic review and meta-analysis provides valuable evidence on the impact of NPIs during the COVID-19 pandemic on the prevalence of RSV in hospitalized children with LRTIs. The findings highlight the effectiveness of NPIs in reducing RSV transmission, particularly during periods of increased respiratory virus circulation. The resurgence of RSV following the relaxation of COVID-19 restrictions emphasizes the ongoing need for surveillance and public health interventions to mitigate the burden of respiratory infections in the post-pandemic era. These findings have important implications for public health policies and interventions to control RSV infections, particularly in vulnerable populations such as young children. Further research is required to investigate the long-term effects of NPIs on the transmission of RSV and to evaluate the potential benefits of vaccination and long-acting RSV monoclonal antibodies in conjunction with NPIs for reducing hospitalizations caused by RSV.

## Figures and Tables

**Table 1:** Sociodemographic and clinical characteristics of the included studies.

Characteristics	N = 112	%
Age range		
2 years	35	31.3
<5 years	22	19.6
<10 years	4	3.6
<15 years	20	17.9
<18 years	27	24.1
Not reported	4	3.6
Male range (%)	39.1–73.8%	
Period of inclusion of participants; range	January 2015–December 2022	
Year of publication; range	2017–2023	
Study design		
Cross-sectional	75	67
Longitudinal	37	33
Sampling method		
Consecutive	110	98.2
Random	2	1.8
Timing of data collection		
Ambispective	4	3.6
Prospective	58	51.8
Retrospective	50	44.6
Study bias		
Low risk	71	63.4
Moderate risk	41	36.6
WHO region		
African	7	6.3
Americas	13	11.6
Eastern Mediterranean	3	2.7
European	47	42
South–East Asia	8	7.1
Western Pacific	34	30.4
Type of assay		
Immune assays ¥	22	19.6
Molecular assays ¥¥	85	75.9
Mixed assays ¥¥¥	5	4.5
Diagnostic technique $		
Antigen	6	5.4
Direct immunofluorescence	16	14.3
Mixed ¥¥¥	5	4.5
Multiplex PCR	14	12.5
Multiplex RT–PCR	18	16.1
PCR	16	14.3
qPCR	2	1.8
RT–PCR	31	27.7
RT–qPCR	4	3.6
Sample type		
Mixed *	7	6.3
Nasal secretions	9	8
Nasopharyngeal secretions	62	55.4
Nasopharyngeal secretions or BLF	11	9.8
Not reported	8	7.1
Oropharyngeal swab	7	6.3
Sputum	3	2.7
Throat swab	5	4.5

¥ Immune assays: antigen testing or direct immunofluorescence. ¥¥ Molecular assays: multiplex PCR, multiplex RT–PCR, PCR, qPCR, RT–PCR, or RT–qPCR. ¥¥¥ Mixed assays: antigen testing/RT–PCR, direct immunofluorescence/RT–qPCR/antigen testing, direct immunofluorescence/RT–PCR, or indirect immunofluorescence/PCR. $ The indicated diagnostic technique is according to the authors’ description. * Mixed specimens: nasopharyngeal secretions and nasal secretions or nasal and throat secretions. Abbreviations: WHO, World Health Organization; BLF, bronchoalveolar lavage fluid.

**Table 2:** Global and subgroup pooled prevalence of respiratory syncytial virus in children with lower respiratory tract infections.

Groups	Studies (n)	RSV-Positive (n)	Total (n)	Pooled Prevalence (95% CI)	Q-Value	*I*^2^ (%)	*p* Value Heterogeneity	*p* Value Egger Test	*p* Value Subgroup Difference
**Overall**	112	45,900	308,985	21.51 [18.42; 24.96]	22,893.65	99.0	<0.0001	<0.0001	NA
**Subgroup analyses**									
**Period**									<0.0001
Pre-pandemic (2015/20)	83	33,735	223,673	25.60 [22.57; 28.88]	15,032.9	99.0	<0.0001	<0.0001	
Lockdown (2020/21)	46	4556	50,298	5.03 [2.67; 9.28]	2581.79	98.3	<0.0001	0.5569	
Post-lockdown (2021/22)	28	7609	35,014	42.02 [31.49; 53.33]	3765.28	99.3	<0.0001	<0.0001	
**Seasons**									<0.0001
2015/16	24	7043	33,225	24.19 [19.45; 29.66]	1620.84	98.60	<0.0001	0.2232	
2016/17	21	4153	24,024	24.10 [17.86; 31.68]	1298.39	98.50	<0.0001	0.035	
2017/18	26	5295	40,942	28.82 [20.40; 39.01]	2855.18	99.10	<0.0001	0.0003	
2018/19	34	7369	41,586	27.02 [21.41; 33.47]	2139.94	98.50	<0.0001	0.0011	
2019/20	42	9875	83,896	24.29 [18.23; 31.58]	5676.49	99.30	<0.0001	0.0003	
2020/21	46	4556	50,298	5.03 [2.67; 9.28]	2581.79	98.30	<0.0001	0.5569	
2021/22	28	7609	35,014	42.02 [31.49; 53.33]	3765.28	99.30	<0.0001	<0.0001	
**WHO Region**									0.0004
African	7	837	3602	19.42 [14.19; 25.98]	224.25	96.0	<0.0001	0.0276	
Americas	13	2029	6370	11.17 [2.19; 41.42]	194.76	93.3	<0.0001	0.7522	
Eastern Mediterranean	3	151	568	26.53 [15.66; 41.25]	33.53	94.0	<0.0001	NA	
European	47	14,877	70,357	28.90 [22.73; 35.98]	7570.45	98.7	<0.0001	0.0004	
South–East Asia	8	1283	5987	24.85 [14.25; 39.67]	758.49	98.7	<0.0001	0.6948	
Western Pacific	34	26,723	222,101	14.71 [12.28; 17.52]	8245.53	99.0	<0.0001	0.1071	
**Age**									<0.0001
<2 years	35	6815	17,696	43.54 [35.56; 51.87]	1972.43	96.7	<0.0001	0.0017	
<5 years	22	7223	36,599	25.59 [21.03; 30.76]	2760.33	98.8	<0.0001	0.0781	
<10 years	4	7706	65,262	17.34 [11.89; 24.58]	1267.48	99.0	<0.0001	0.1347	
<15 years	20	5745	40,646	17.58 [12.82; 23.64]	4016.97	99.1	<0.0001	0.7428	
<18 years	27	17,408	139,980	10.17 [7.29; 14.02]	5,380.77	99.0	<0.0001	0.9850	
Not reported	4	1003	8802	9.94 [5.19; 18.18]	276.85	95.7	<0.0001	0.3230	
**Design**									0.0840
Cross-sectional	75	35,091	263,251	19.64 [16.55; 23.15]	15,271.27	98.9	<0.0001	<0.0001	
Longitudinal	37	10,809	45,734	27.34 [19.47; 36.94]	4589.11	98.8	<0.0001	0.0379	
**Timing of data collection**									0.5983
Ambispective	4	1449	11,714	15.30 [4.70; 39.82]	657.29	98.8	<0.0001	NA	
Prospective	58	12,676	66,479	23.44 [18.63; 29.05]	7496.05	98.9	<0.0001	0.1370	
Retrospective	50	31,775	230,792	20.82 [16.90; 25.37]	12,692.16	99.0	<0.0001	0.0007	
**Risk of bias**									0.1453
Low risk	71	31,179	222,366	23.29 [19.18; 27.98]	17,585.49	99.2	<0.0001	<0.0001	
Moderate risk	41	14,721	86,619	18.49 [14.32; 23.54]	5058.52	98.5	<0.0001	0.0752	
**Type of assay**									0.0007
Immune assays ¥	22	19,476	180,647	13.94 [10.65; 18.04]	4163.17	98.9	<0.0001	0.5255	
Molecular assays ¥¥	85	22,523	103,157	24.96 [20.77; 29.69]	10,441.27	98.5	<0.0001	0.0350	
Mixed assays ¥¥¥	5	3901	25,181	14.74 [8.27; 24.91]	872.62	96.3	<0.0001	0.3155	
**Diagnostic technique $**									0.0016
Antigen testing	6	8018	75,112	17.78 [10.71; 28.05]	1187.05	98.9	<0.0001	0.4242	
Direct immunofluorescence	16	11,458	105,535	12.46 [9.14; 16.77]	2892.56	99.0	<0.0001	0.8831	
Mixed assays ¥¥¥	5	3901	25,181	14.74 [8.27; 24.91]	1187.05	98.9	<0.0001	0.3155	
Multiplex PCR	14	2083	10,831	27.32 [14.57; 45.30]	2965.49	99.0	<0.0001	0.0439	
Multiplex RT–PCR	18	2215	7385	30.89 [22.03; 41.42]	872.62	98.3	<0.0001	0.3490	
PCR	16	7247	32,319	24.91 [16.89; 35.12]	1756.55	98.7	<0.0001	0.8591	
qPCR	2	52	140	38.93 [21.17; 60.20]	828.23	97.0	0.0008	NA	
RT–PCR	31	9193	46,668	22.15 [16.57; 28.95]	3648.00	99.1	<0.0001	0.0936	
RT–qPCR	4	1733	5814	17.82 [3.59; 55.79]	11.32	91.2	<0.0001	NA	
**Sample type**									0.0023
Mixed specimens *	7	3091	14,765	18.35 [8.59; 34.98]	1408.90	99.0	<0.0001	0.7840	
Nasal secretions	9	1209	3493	21.39 [6.94; 49.83]	263.44	95.8	<0.0001	0.5521	
Nasopharyngeal secretions	62	21,542	114,495	26.25 [21.48; 31.66]	9150.13	98.7	<0.0001	<0.0001	
Nasopharyngeal secretions or BLF	11	4374	52,107	12.12 [8.34; 17.28]	1848.89	98.6	<0.0001	0.0770	
Not reported	8	10,981	85,775	21.26 [10.99; 37.13]	2581.81	99.3	<0.0001	0.0036	
Oropharyngeal secretions	7	2663	20,375	15.48 [12.23; 19.39]	778.85	97.7	<0.0001	0.2721	
Sputum	3	549	2063	25.23 [18.38; 33.58]	44.03	95.5	<0.0001	NA	
Throat secretions	5	1491	15,912	16.79 [7.88; 32.26]	1097.90	99.3	<0.0001	NA	

¥ Immune assays: Antigen testing; or Direct immunofluorescence. ¥¥ Molecular assays: Multiplex PCR, Multiplex RT–PCR, PCR, qPCR, RT–PCR, or RT–qPCR. ¥¥¥ Mixed assays: Antigen testing/RT–PCR; direct immunofluorescence/RT–qPCR/antigen testing; direct immunofluorescence/RT–PCR; or indirect immunofluorescence/PCR. $ The indicated diagnostic technique is according to what is referred to by the authors. * Mixed specimens: nasopharyngeal secretions and nasal secretions or nasal and throat secretions. Abbreviations: WHO, World Health Organization; BLF, bronchoalveolar lavage fluid; NA, not applicable.

**Table 3:** Analysis by World Health Organization regions and countries.

Groups	Studies (n)	RSV-Positive (n)	Total (n)	Pooled Prevalence (95% CI)	Q-Value	*I*^2^ (%)	*p* Value Heterogeneity	*p* Value Egger Test	*p* Value Subgroup Difference
**WHO Region ##**									
**European**	47	14,877	70,357	28.90 [22.73; 35.98]	7570.45	98.7	<0.0001	0.0004	
**Period**									<0.0001
Pre-pandemic (2015/20)	28	10,348	48,140	34.81 [29.47; 40.56]	3905.86	98.50	<0.0001	0.0001	
Lockdown (2020/21)	23	1028	12,898	4.89 [1.68; 13.39]	853.67	97.40	<0.0001	0.7463	
Post-lockdown (2021/22)	18	3501	9319	55.18 [42.96; 66.80]	1479.62	98.90	<0.0001	0.0093	
**Western Pacific**	34	26,723	222,101	14.71 [12.28; 17.52]	8245.53	99.0	<0.0001	0.1071	
**Period**									0.0024
Pre-pandemic (2015/20)	29	19,728	162,313	16.12 [13.28; 19.43]	6290.45	99.10	<0.0001	0.0424	
Lockdown (2020/21)	16	3215	34,966	8.03 [4.99; 12.68]	1240.05	98.80	<0.0001	0.5502	
Post-lockdown (2021/22)	8	3780	24,822	22.83 [15.42; 32.42]	356.45	98.00	<0.0001	NA	
**Countries &&**									
**Italy**	20	5437	14,928	47.14 [37.07; 57.45]	1877.72	97.8	<0.0001	0.0011	
**Period**									0.0024
Pre-pandemic (2015/20)	10	2955	9600	51.87 [43.11; 60.51]	815.15	97.40	<0.0001	<0.0001	
Lockdown (2020/21)	8	43	801	5.93 [0.89; 30.68]	110.71	93.70	<0.0001	NA	
Post-lockdown (2021/22)	13	2439	4527	63.59 [53.25; 72.81]	381.83	96.90	<0.0001	0.0136	
**China**	27	23,416	211,831	11.33 [9.61; 13.30]	4416.56	98.6	<0.0001	0.6889	
**Period**									0.0002
Pre-pandemic (2015/20)	23	16,898	153,351	12.04 [10.12; 14.26]	3350.33	98.70	<0.0001	0.79	
Lockdown (2020/21)	12	2868	34,012	6.64 [4.83; 9.07]	347.18	96.80	<0.0001	0.0314	
Post-lockdown (2021/22)	6	3650	24,468	19.89 [12.49; 30.15]	237.17	97.90	<0.0001	NA	

## The World Health Organization (WHO) regions, including Africa, the Americas, the Eastern Mediterranean, and South East Asia, lack sufficient prevalence data for conducting this meta-analysis across periods. && Furthermore, the remaining countries also lack adequate prevalence data for conducting this meta-analysis across periods. Abbreviations: NA, not applicable.

**Table 4:** Analysis by age stratum.

Groups	Studies (n)	RSV-positive (n)	Total (n)	Pooled Prevalence (95%CI)	Q-Value	*I*^2^ (%)	*p* Value Heterogeneity	*p* Value Egger Test	*p* Value Subgroup Difference
**Age stratum**									
**Age < 2 years**	35	6815	17,696	43.54 [35.56; 51.87]	1972.43	96.7	<0.0001	0.0017	NA
**Period**									<0.0001
Pre-pandemic (2015/20)	23	4844	12,939	47.82 [42.06; 53.65]	1019.71	96.10	<0.0001	<0.0001	
Lockdown (2020/21)	14	261	1359	6.46 [1.19; 28.29]	137.86	90.60	<0.0001	0.1192	
Post-lockdown (2021/22)	12	1710	3398	67.61 [57.01; 76.67]	698.1	98.40	<0.0001	0.0004	
**Age < 5 years**	22	7223	36,599	25.59 [21.03; 30.76]	2760.33	98.8	<0.0001	0.0781	NA
**Period**									0.1193
Pre-pandemic (2015/20)	19	5424	23,892	25.58 [20.94; 30.84]	1464.93	98.10	<0.0001	0.4885	
Lockdown (2020/21)	3	1422	11,913	17.34 [7.61; 34.84]	380.63	99.50	<0.0001	NA	
Post-lockdown (2021/22)	2	377	794	42.69 [24.30; 63.35]	57.54	98.30	<0.0001	NA	
**Age < 10 years**	4	7706	65,262	17.34 [11.89; 24.58]	1267.48	99.0	<0.0001	0.1347	NA
**Period**									0.0384
Pre-pandemic (2015/20)	4	5554	48,007	20.48 [13.90; 29.11]	1249.79	99.20	<0.0001	0.1212	
Lockdown (2020/21)	2	795	6989	7.39 [2.93; 17.40]	5.34	81.30	0.0208	NA	
Post-lockdown (2021/22)	1	1357	10,266	13.22 [12.58; 13.89]	0	NA	NA	NA	
**Age < 15 years**	20	5745	40,646	17.58 [12.82; 23.64]	4016.97	99.1	<0.0001	0.7428	NA
**Period**									<0.0001
Pre-pandemic (2015/20)	15	3963	31,279	19.46 [14.11; 26.23]	1939.82	98.70	<0.0001	0.0083	
Lockdown (2020/21)	7	304	5790	6.02 [2.38; 14.39]	168.99	96.40	<0.0001	NA	
Post-lockdown (2021/22)	6	1478	3577	31.37 [17.22; 50.12]	87.88	94.30	<0.0001	NA	
**Age < 18 years**	27	17,408	139,980	10.17 [7.29; 14.02]	5380.77	99.0	<0.0001	0.9850	NA
**Period**									<0.0001
Pre-pandemic (2015/20)	19	13,026	99,877	16.68 [13.29; 20.72]	3843.26	99.20	<0.0001	0.1671	
Lockdown (2020/21)	17	1695	23,124	2.53 [1.07; 5.85]	1230.46	98.70	<0.0001	0.1565	
Post-lockdown (2021/22)	7	2687	16,979	17.61 [14.30; 21.51]	48.9	87.70	<0.0001	NA	

Abbreviations: NA, not applicable.

## Data Availability

All data are available in the main article and Supplementary Materials SA and SB.

## Supplementary Materials

The following supporting information can be downloaded at: https://www.mdpi.com/article/10.3390/v16030429/s1, Supplementary Material SA: Table S1: Preferred reporting items for systematic reviews and meta-analyses (PRISMA) 2020 checklist; Table S2: Search strategies from the PubMed, Scopus, and Web of Science databases; Table S3: Risk of bias tool by Hoy et al.; Table S4: Risk of bias assessment; Table S5: Countries of origin of the studies included in this systematic review and meta-analysis; Table S6: Characteristics of all included studies; Table S7: Definitions of lower respiratory tract infection according to each study; Table S8: Sensitivity analysis of pooled respiratory syncytial virus (RSV) prevalence in children diagnosed with RSV-related lower respiratory tract infection using molecular assays; Table S9: Univariable meta-regression model for the prevalence of the respiratory syncytial virus in hospitalized children with lower respiratory tract infections; Table S10: The AMSTAR 2 (A Measurement Tool to Assess Systematic Reviews) instrument; Figure S1: Respiratory syncytial virus (RSV) prevalences according to RSV season; Figure S2: Funnel plot of sensitivity analysis of pooled respiratory syncytial virus (RSV) prevalence in children diagnosed with RSV-related lower respiratory tract infection by molecular assays: Figure S3: Bubble plot illustrating the association between average age in months and prevalence of respiratory syncytial virus; Figure S4. Prevalence of intensive care unit (ICU) admissions in hospitalized children with lower respiratory tract infections related to respiratory syncytial virus (RSV); Figure S5. Mortality among hospitalized children with lower respiratory tract infections related to respiratory syncytial virus (RSV), references of all 112 of the included studies are cited in the Tables S4, S6 and S7, Figures S1, S4 and S5; Supplementary Material SB: Table S1: Reasons for excluding full-text articles from the systematic review via databases, registers, and hand-searching.
