# Supplementary material for: Impact of Nonpharmaceutical Interventions during the COVID-19 Pandemic on the Prevalence of Respiratory Syncytial Virus in Hospitalized Children with Lower Respiratory Tract Infections: A Systematic Review and Meta-Analysis
Source: Viruses. 2024 Mar 11;16(3):429. doi: 10.3390/v16030429 (PMC10974159; doi:10.3390/v16030429)
Supplement: Supplementary file 1 [file viruses-16-00429-s001.zip › viruses-2827065_Supplementary_Material_A.pdf]

Supplementary Material SA.

Table S1. Preferred reporting items for systematic reviews and meta-analyses (PRISMA) 2020 checklist.

Section and Topic	Item #	Checklist item	Location where item is reported
**TITLE**			
Title	1	Identify the report as a systematic review.	Title
**ABSTRACT**			
Abstract	2	See the PRISMA 2020 for Abstracts checklist.	Abstract
**INTRODUCTION**			
Rationale	3	Describe the rationale for the review in the context of existing knowledge.	Introduction
Objectives	4	Provide an explicit statement of the objective(s) or question(s) the review addresses.	Introduction in the last paragraph and Methods.
**METHODS**			
Eligibility criteria	5	Specify the inclusion and exclusion criteria for the review and how studies were grouped for the syntheses.	Methods
Information sources	6	Specify all databases, registers, websites, organisations, reference lists and other sources searched or consulted to identify studies. Specify the date when each source was last searched or consulted.	Methods
Search strategy	7	Present the full search strategies for all databases, registers, and websites, including any filters and limits used.	Table S2 in Supplementary Material.
Selection process	8	Specify the methods used to decide whether a study met the inclusion criteria of the review, including how many reviewers screened each record and each report retrieved, whether they worked independently, and if applicable, details of automation tools used in the process.	Methods
Data collection process	9	Specify the methods used to collect data from reports, including how many reviewers collected data from each report, whether they worked independently, any processes for obtaining or confirming data from study investigators, and if applicable, details of automation tools used in the process.	Methods
Data items	10a	List and define all outcomes for which data were sought. Specify whether all results that were compatible with each outcome domain in each study were sought (e.g. for all measures, time points, analyses), and if not, the methods used to decide which results to collect.	Methods
	10b	List and define all other variables for which data were sought (e.g. participant and intervention characteristics, funding sources). Describe any assumptions made about any missing or unclear information.	Methods
Study risk of bias assessment	11	Specify the methods used to assess risk of bias in the included studies, including details of the tool(s) used, how many reviewers assessed each study and whether they worked independently, and if applicable, details of automation tools used in the process.	Methods. Tables S3 and S4 in Supplementary

Section and Topic	Item #	Checklist item	Location where item is reported
			Material.
Effect measures	12	Specify for each outcome the effect measure(s) (e.g. risk ratio, mean difference) used in the synthesis or presentation of results.	Methods
Synthesis methods	13a	Describe the processes used to decide which studies were eligible for each synthesis (e.g. tabulating the study intervention characteristics and comparing against the planned groups for each synthesis (item #5)).	Methods
	13b	Describe any methods required to prepare the data for presentation or synthesis, such as handling of missing summary statistics, or data conversions.	Methods
	13c	Describe any methods used to tabulate or visually display results of individual studies and syntheses.	Methods
	13d	Describe any methods used to synthesize results and provide a rationale for the choice(s). If meta-analysis was performed, describe the model(s), method(s) to identify the presence and extent of statistical heterogeneity, and software package(s) used.	Methods
	13e	Describe any methods used to explore possible causes of heterogeneity among study results (e.g. subgroup analysis, meta-regression).	Methods
	13f	Describe any sensitivity analyses conducted to assess robustness of the synthesized results.	Methods
Reporting bias assessment	14	Describe any methods used to assess risk of bias due to missing results in a synthesis (arising from reporting biases).	Not applicable because this review has no missing data.
Certainty assessment	15	Describe any methods used to assess certainty (or confidence) in the body of evidence for an outcome.	Methods in the last paragraph. Table S10 in Supplementary Material.
**RESULTS**			
Study selection	16a	Describe the results of the search and selection process, from the number of records identified in the search to the number of studies included in the review, ideally using a flow diagram.	Figure 1
	16b	Cite studies that might appear to meet the inclusion criteria, but which were excluded, and explain why they were excluded.	Appendix A.
Study characteristics	17	Cite each included study and present its characteristics.	Results Table 1. Tables S6 and S7 in Supplementary Material.
Risk of bias in studies	18	Present assessments of risk of bias for each included study.	Tables S3 and S4 in Supplementary

Section and Topic	Item #	Checklist item	Location where item is reported
			Material.
Results of individual studies	19	For all outcomes, present, for each study: (a) summary statistics for each group (where appropriate) and (b) an effect estimate and its precision (e.g. confidence/credible interval), ideally using structured tables or plots.	Results Table 2 and Figure 2. Tables S8 and Figure S1 in Supplementary Material.
Results of syntheses	20a	For each synthesis, briefly summarise the characteristics and risk of bias among contributing studies.	Results Table 2 and Table S9 in Supplementary Material. Discussion section.
	20b	Present results of all statistical syntheses conducted. If meta-analysis was done, present for each the summary estimate and its precision (e.g. confidence/credible interval) and measures of statistical heterogeneity. If comparing groups, describe the direction of the effect.	Results Table 2 and Figure 2. Tables S8 and Figure S1 in Supplementary Material.
	20c	Present results of all investigations of possible causes of heterogeneity among study results.	Results Table 2. Tables S8 and S9 and Figure S3 in Supplementary Material.
	20d	Present results of all sensitivity analyses conducted to assess the robustness of the synthesized results.	Table S8 in Supplementary Material.
Reporting biases	21	Present assessments of risk of bias due to missing results (arising from reporting biases) for each synthesis assessed.	Not applicable because this review has no missing data.
Certainty of	22	Present assessments of certainty (or confidence) in the body of evidence for each outcome assessed.	Table S10 in

Section and Topic	Item #	Checklist item	Location where item is reported
evidence			Supplementary Material.
**DISCUSSION**			
Discussion	23a	Provide a general interpretation of the results in the context of other evidence.	Discussion
	23b	Discuss any limitations of the evidence included in the review.	Discussion
	23c	Discuss any limitations of the review processes used.	Discussion
	23d	Discuss implications of the results for practice, policy, and future research.	Discussion
**OTHER INFORMATION**			
Registration and protocol	24a	Provide registration information for the review, including register name and registration number, or state that the review was not registered.	Abstract and Methods.
	24b	Indicate where the review protocol can be accessed, or state that a protocol was not prepared.	Methods
	24c	Describe and explain any amendments to information provided at registration or in the protocol.	No amendments were made to the protocol.
Support	25	Describe sources of financial or non-financial support for the review, and the role of the funders or sponsors in the review.	Funding statement section.
Competing interests	26	Declare any competing interests of review authors.	Competing interests section.
Availability of data, code and other materials	27	Report which of the following are publicly available and where they can be found: template data collection forms; data extracted from included studies; data used for all analyses; analytic code; any other materials used in the review.	Main manuscript, Supplementary Material, and Appendix A.

From: Page MJ, McKenzie JE, Bossuyt PM, Boutron I, Hoffmann TC, Mulrow CD, et al. The PRISMA 2020 statement: an updated guideline for reporting systematic reviews. BMJ 2021;372:n71. doi: 10.1136/bmj.n71
For more information, visit: http://www.prisma-statement.org/

Table S2. Search strategies from the PubMed, Scopus, and Web of Science databases.

PubMed search strategy:

(((respiratory syncytial virus or RSV) AND epidemiology) or ((respiratory syncytial virus or RSV) and seasonality) or ((respiratory syncytial virus or RSV) and hospitalization)) NOT (resveratrol OR Rous sarcoma virus OR bovine OR mice OR ferret OR macrophages OR in vitro OR nosocomial OR mathematical) Filters: from 2015/1/1 - 2022/12/31

Scopus search strategy:

(((TITLE-ABS ((("respiratory syncytial virus" OR "RSV") AND "epidemiology"))) OR (TITLE-ABS ((("respiratory syncytial virus" OR "RSV") AND "seasonality"))) OR ((TITLE-ABS ("respiratory syncytial virus" OR "RSV")) AND (TITLE-ABS ("hospitalization"))))) AND NOT (ALL (("resveratrol" OR "Rous sarcoma virus" OR "bovine" OR "mice" OR "ferret" OR "macrophages" OR "in vitro" OR "nosocomial" OR "mathematical")))) AND (PUBYEAR > 2014 AND PUBYEAR < 2023)

Web of Science search strategy:

1: ALL=(((respiratory syncytial virus or RSV) AND epidemiology))

2: ALL=(((respiratory syncytial virus or RSV) and seasonality))

3: ALL=((respiratory syncytial virus or RSV) and hospitalization)

4: ALL=((resveratrol OR Rous sarcoma virus OR bovine OR mice OR ferret OR macrophages OR in vitro OR nosocomial OR mathematical))

5: ((#1) OR #2) OR #3 Timespan: 2015-01-01 to 2022-12-31

6: (#5) NOT #4 Timespan: 2015-01-01 to 2022-12-31

Table S3. Risk of bias tool by Hoy et al.

The Hoy et al. tool for assessing bias in prevalence studies.	Yes (1)/No (0)
**Domains of external validity**	
1. Was the study's target population a close representation of the national population in relation to relevant variables?	
2. Was the sampling frame a true or close representation of the target population?	
3. Was some form of random selection used to select the sample, OR was a census undertaken?	
4. Was the likelihood of nonresponse bias minimal?	
**Domains of internal validity**	
5. Were data collected directly from the subjects (as opposed to a proxy)?	
6. Was an acceptable case definition used in the study?	
7. Was the study instrument that measured the parameter of interest shown to have validity and reliability?	
8. Was the same mode of data collection used for all subjects?	
9. Was the length of the shortest prevalence period for the parameter of interest appropriate?	
10. Were the numerator(s) and denominator(s) for the parameter of interest appropriate?	

Comment: In this review, we identified the target population as children who were hospitalized due to lower respiratory tract infection (LRTI). Furthermore, we utilized the term "LRTI" as the case definition.

Interpretation of the risk of bias tool

• 7-10: Low risk of bias

• 4-6: Moderate risk of bias

• 0-3: High risk of bias.

Reference:

Hoy D, Brooks P, Woolf A, et al. Assessing risk of bias in prevalence studies: modification of an existing tool and evidence of interrater agreement. J Clin Epidemiol. 2012;65(9):934-939. doi:10.1016/j.jclinepi.2011.11.014

Table S4. Risk of bias assessment.

Author	D1	D2	D3	D4	D5	D6	D7	D8	D9	D10	Score	Risk of bias
Agca et al. [1]	0	0	0	1	1	0	1	1	1	1	6	Moderate risk
Akkoc et al. [2]	0	1	0	1	1	1	1	1	1	1	8	Low risk
Al-Zayadneh et al. [3]	1	1	0	1	1	1	1	1	1	1	9	Low risk
Assane et al. [4]	0	0	0	0	1	0	1	1	1	1	5	Moderate risk
Baldassarre et al. [5]	1	1	0	1	1	1	1	1	1	1	9	Low risk
Barbati et al. [6]	0	0	0	0	1	0	1	1	1	1	5	Moderate risk
Bedir Demirdağ et al. [7]	0	0	0	0	1	0	1	1	1	1	5	Moderate risk
Ben-Shimol et al. [8]	0	1	0	1	1	1	1	1	1	1	8	Low risk
Berdah et al. [9]	1	1	0	1	1	1	1	1	1	1	9	Low risk
Bermúdez Barrezueta et al. [10]	1	1	0	1	1	1	1	1	1	1	9	Low risk
Bhardwaj et al. [11]	0	0	0	1	1	0	1	1	1	1	6	Moderate risk
Brisca et al. [12]	1	1	0	1	1	1	1	1	1	1	9	Low risk
Buendia et al. [13]	1	1	0	1	1	1	1	1	1	1	9	Low risk
Buendía et al. [14]	1	1	0	1	1	1	1	1	1	1	9	Low risk
Camporesi et al. [15]	1	1	0	1	1	1	1	1	1	1	9	Low risk
Camporesi et al. [16]	0	1	0	1	1	1	1	1	1	1	8	Low risk
Cao et al. [17]	0	1	1	1	1	1	1	1	1	1	9	Low risk
Carlone et al. [18]	0	0	0	1	1	0	1	0	1	1	5	Low risk
Chandy et al. [19]	1	1	0	1	1	1	1	1	1	1	9	Low risk
Chu et al. [20]	1	1	0	1	1	1	1	1	1	1	9	Low risk
Coma et al. [21]	1	1	0	1	1	1	1	0	1	1	8	Low risk
Curatola et al. [22]	0	0	0	1	1	0	1	1	0	1	5	Moderate risk
Curatola et al. [23]	0	0	0	1	1	0	1	1	1	1	6	Moderate risk
De Rose et al. [24]	0	1	0	1	1	1	1	1	1	1	8	Low risk
Diesner-Treiber et al. [25]	1	1	0	1	1	1	1	1	1	1	9	Low risk
Esen et al. [26]	1	1	0	1	1	1	1	1	1	1	9	Low risk

Faraguna et al. [27]	0	1	0	1	1	1	1	1	1	1	8	Low risk
Feng et al. [28]	0	1	0	1	1	1	1	1	1	1	8	Low risk
Foley et al. [29]	1	1	0	1	1	1	1	1	1	1	9	Low risk
Giamberardino et al. [30]	0	0	0	0	1	0	1	1	1	1	5	Moderate risk
Gonapaladeniya et al. [31]	1	1	0	1	1	1	1	1	1	1	9	Low risk
Grochowska et al. [32]	1	1	0	1	1	1	1	0	1	1	8	Low risk
Guerrero-Del-Cueto et al. [33]	1	1	0	1	1	1	1	0	1	1	8	Low risk
Guitart et al. [34]	1	1	0	1	1	1	1	1	1	1	9	Low risk
Haddadin et al. [35]	0	1	0	1	1	1	1	1	1	1	8	Low risk
Havdal et al. [36]	1	1	0	1	1	1	1	1	1	1	9	Low risk
Hu et al. [37]	1	1	0	1	1	0	1	1	1	1	8	Low risk
Ippolito et al. [38]	0	0	0	1	1	0	1	1	1	1	6	Moderate risk
Jiang et al. [39]	1	1	0	1	1	1	1	1	1	1	9	Low risk
Jiang et al. [40]	1	1	0	1	1	1	1	1	1	1	9	Low risk
Jiang et al. [41]	0	1	0	1	1	1	1	1	1	1	8	Low risk
Jullien et al. [42]	1	1	0	1	1	1	1	1	1	1	9	Low risk
Kang et al. [43]	0	0	0	1	1	0	1	1	1	1	6	Moderate risk
Kıymet et al. [44]	0	0	0	1	1	0	1	1	1	1	6	Moderate risk
Knudsen et al. [45]	1	1	0	1	1	1	1	1	1	1	9	Low risk
Kockuzu et al. [46]	0	0	0	1	1	0	1	1	1	1	6	Moderate risk
Komoyo et al. [47]	0	0	0	1	1	0	1	1	1	1	6	Moderate risk
Korsun et al. [48]	0	0	0	1	1	0	1	1	1	1	6	Moderate risk
Kume et al. [49]	0	0	0	1	1	0	1	1	1	1	6	Moderate risk
Kurskaya et al. [50]	0	1	0	1	1	1	1	1	1	1	8	Low risk
Lagare et al. [51]	1	1	0	1	1	1	1	1	1	1	9	Low risk
Lamrani Hanchi et al. [52]	1	1	0	0	1	1	1	1	1	1	8	Low risk
Li et al. [53]	0	1	0	1	1	1	1	1	1	1	8	Low risk
Li et al. [54]	0	1	0	1	1	1	1	1	1	1	8	Low risk
Li et al. [55]	1	1	0	1	1	1	1	1	1	1	9	Low risk

Li et al. [56]	0	0	0	1	1	0	1	1	1	1	6	Moderate risk
Liu et al. [57]	1	1	0	1	1	1	1	1	1	1	9	Low risk
Liu et al. [58]	1	1	0	1	1	1	1	1	1	1	9	Low risk
Lucion et al. [59]	0	1	0	1	1	1	1	0	1	1	7	Low risk
Ma et al. [60]	1	1	0	1	1	1	1	1	1	1	9	Low risk
Mackenzie et al. [61]	1	1	0	1	1	1	1	1	1	1	9	Low risk
Maglione et al. [62]	0	0	0	1	1	0	1	0	0	1	4	Moderate risk
Maruo et al. [63]	0	0	0	1	1	0	1	1	1	1	6	Moderate risk
Mira-Iglesias et al. [64]	1	1	0	1	1	1	1	1	1	1	9	Low risk
Moreira et al. [65]	0	1	0	1	1	0	1	1	1	1	7	Low risk
Nenna et al. [66]	0	0	0	1	1	0	1	1	1	1	6	Moderate risk
Nenna et al. [67]	1	1	0	1	1	1	1	1	1	1	9	Low risk
Ng et al. [68]	0	1	0	1	1	1	1	1	1	1	8	Low risk
Núñez-Samudio et al. [69]	0	1	0	1	1	1	1	1	1	1	8	Low risk
Nunziata et al. [70]	1	1	0	1	1	1	1	1	1	1	9	Low risk
Oumei et al. [71]	1	1	0	1	1	1	1	1	1	1	9	Low risk
Pale et al. [72]	1	1	0	1	1	1	1	1	1	1	9	Low risk
Pierangeli et al. [73]	0	0	0	1	1	0	1	1	1	1	6	Moderate risk
Pogonowska et al. [74]	0	0	0	1	1	0	1	1	1	1	6	Moderate risk
Prasad et al. [75]	1	1	1	1	1	1	1	1	1	1	10	Low risk
Prasad et al. [76]	1	1	0	1	1	1	1	1	1	1	9	Low risk
Presti et al. [77]	0	0	0	1	1	0	1	1	1	1	6	Moderate risk
Pruccoli et al. [78]	1	0	0	1	1	0	1	0	1	1	6	Moderate risk
Qiu et al. [79]	1	1	0	1	1	1	1	1	1	1	9	Low risk
Ramezannia et al. [80]	0	0	0	1	1	0	1	1	1	1	6	Moderate risk
Rha et al. [81]	1	1	0	1	1	1	1	1	1	1	9	Low risk
Roland et al. [82]	0	0	0	1	1	0	1	1	1	1	6	Moderate risk
Sachs et al. [83]	0	1	0	1	1	1	1	1	1	1	8	Low risk
Sarkar et al. [84]	0	1	0	1	1	1	1	1	1	1	8	Low risk

Savaş Şen et al. [85]	1	1	0	1	1	1	1	1	1	1	9	Low risk
Shen et al. [86]	0	1	0	1	1	1	1	1	1	1	8	Low risk
Shen et al. [87]	0	0	0	1	1	0	1	1	1	1	6	Moderate risk
Shi et al. [88]	0	0	0	1	1	0	1	1	1	1	6	Moderate risk
Singh et al. [89]	0	0	0	1	1	0	1	1	1	1	6	Moderate risk
Sobrinho et al. [90]	1	1	0	1	1	1	1	0	1	1	8	Low risk
Song et al. [91]	0	0	0	1	1	0	1	1	1	1	6	Moderate risk
Stera et al. [92]	1	1	0	1	1	1	1	1	1	1	9	Low risk
Tang et al. [93]	0	0	0	0	1	0	1	1	1	1	5	Moderate risk
Thongpan et al. [94]	0	0	0	1	1	0	1	1	1	1	6	Moderate risk
Thwaites et al. [95]	1	1	0	1	1	1	1	1	1	1	9	Low risk
Trenholme et al. [96]	1	1	0	1	1	1	1	1	1	1	9	Low risk
Tsergouli et al. [97]	0	0	0	1	1	0	1	1	1	1	6	Moderate risk
Valley-Omar et al. [98]	0	0	0	1	1	0	1	1	1	1	6	Moderate risk
Varela et al. [99]	0	0	0	0	1	0	1	1	1	1	5	Moderate risk
Virant et al. [100]	1	1	0	1	1	0	1	1	1	1	8	Low risk
Vittucci et al. [101]	0	0	0	1	1	0	1	1	1	1	6	Moderate risk
Wang et al. [102]	0	0	0	1	1	0	1	1	1	1	6	Moderate risk
Watanabe et al. [103]	0	0	0	1	1	0	1	1	1	1	6	Moderate risk
Weldetsadik et al. [104]	0	1	0	1	1	1	1	1	1	1	8	Low risk
Wen et al. [105]	0	0	0	1	1	0	1	1	1	1	6	Moderate risk
Windsor et al. [106]	0	0	1	0	1	0	1	1	1	1	6	Moderate risk
Xia et al. [107]	1	1	0	1	1	1	1	1	1	1	9	Low risk
Xu et al. [108]	1	1	0	1	1	1	1	1	1	1	9	Low risk
Yan et al. [109]	0	1	0	1	1	1	1	1	1	1	8	Low risk
Ye et al. [110]	1	1	0	1	1	1	1	1	1	1	9	Low risk
Zhao et al. [111]	0	0	0	1	1	0	1	1	1	1	6	Moderate risk
Zhao et al. [112]	0	0	0	1	1	0	1	1	1	1	6	Moderate risk

Abbreviations: D, Domain.

Table S5. Countries of the studies included in this systematic review and meta-analysis.

Country	N=112	Percent (%)
Argentina	1	0.9
Australia	1	0.9
Austria	1	0.9
Belgium	1	0.9
Bhutan	1	0.9
Brazil	5	4.5
Bulgaria	1	0.9
Central African Republic	1	0.9
China	27	24.1
Colombia	2	1.8
Ethiopia	1	0.9
France	1	0.9
Gambia	1	0.9
Greece	1	0.9
India	5	4.5
Iran	1	0.9
Israel	2	1.8
Italy	20	17.9
Japan	2	1.8
Jordan	1	0.9
Malaysia	1	0.9
Morocco	1	0.9
Mozambique	1	0.9
New Zealand	3	2.7
Niger	1	0.9
Norway	2	1.8
Panama	1	0.9
Poland	2	1.8
Russia	1	0.9
Senegal	1	0.9
Slovenia	1	0.9
South Africa	1	0.9
Spain	5	4.5
Sri Lanka	1	0.9

Thailand	1	0.9
Turkey	7	6.3
United Kingdom	2	1.8
United States of America	4	3.6
**Total**	112	100

Table S6. Characteristics of all included studies.

Study	Period	Design	Sampling	Timing	Country	WHO-Region	Sample type	Diagnostic technique$	Age	Male (%)
Agca et al. [1] 2021	March 2020 to February 2021.	Cross-sectional	Consecutive	Retrospective	Turkey	European	Nasopharyngeal secretions	Multiplex RT-PCR	< 18 years	62.4%
Akkoc et al. [2] 2022	January 2019 to February 2020	Cross-sectional	Consecutive	Retrospective	Turkey	European	Nasopharyngeal secretions	Multiplex RT-PCR	1 month to 5 years	73.8%
Al-Zayadneh et al. [3] 2021	December 2018 to April 2019	Longitudinal	Consecutive	Prospective	Jordan	Eastern Mediterranean	Nasopharyngeal secretions	Multiplex RT-PCR	1 month to 15 years	62.5%
Assane et al. [4] 2018	January 2015 to January 2016	Cross-sectional	Consecutive	Prospective	Senegal	African	Nasopharyngeal secretions	Multiplex RT-PCR	< 5 years	59.9%
Baldassarre et al. [5] 2023	1 January to 31 December 2021.	Cross-sectional	Consecutive	Retrospective	Italy	European	Not reported	RT-PCR	0–12 months	55.30%
Barbati et al. [6] 2020	September 2014 to August 2019	Cross-sectional	Consecutive	Retrospective	Italy	European	Nasopharyngeal secretions or BLF	RT-qPCR	< 6 years	55.4%
Bedir Demirdağ et al. [7] 2022	October 1, 2021 to January 1, 2022	Cross-sectional	Consecutive	Retrospective	Turkey	European	Nasopharyngeal secretions	Multiplex PCR	18 days to 17.9 years	64.3%
Ben-Shimol et al. [8] 2023	January 2019 to March 2020	Longitudinal	Consecutive	Prospective	Israel	European	Nasopharyngeal secretions	RT-PCR	2–17 months	68.40%
Berdah et al. [9] 2022	October 1, 2017 to March 31, 2021.	Cross-sectional	Consecutive	Retrospective	France	European	Nasopharyngeal secretions	PCR	< 1 year	58.0%
Bermúdez Barrezueta et al. [10] 2022	March 15, 2020, to August 31, 2021	Cross-sectional	Consecutive	Ambispective	Spain	European	Nasopharyngeal secretions	Direct immunofluoresce nce	< 2 years	58.3%
Bhardwaj et al. [11] 2022	January 2017 to December 2021	Cross-sectional	Consecutive	Retrospective	India	South-East Asia	Throat swab	RT-PCR	< 15 years	61.7%
Brisca et al. [12] 2023	September 1, 2017, to June 30, 2022.	Cross-sectional	Consecutive	Retrospective	Italy	European	Nasopharyngeal secretions	Multiplex PCR	0–2 years	50.20%
Buendia et al. [13] 2021	January 2019 to December 2019.	Longitudinal	Consecutive	Retrospective	Colombia	Americas	Nasopharyngeal secretions	Direct immunofluoresce nce	< 2 years	60.3%
Buendía et al. [14] 2021	January 2015 to December 2016	Cross-sectional	Consecutive	Retrospective	Colombia	Americas	Not reported	Direct immunofluoresce nce	< 2 years	58.55%
Camporesi et al. [15] 2023	1 July 2021 to March 2023.	Longitudinal	Consecutive	Prospective	Italy	European	Nasal secretions	RT-PCR	< 2 years	56.90%
Camporesi et al. [16] 2022	July 1, 2021 to January 31, 2022.	Longitudinal	Consecutive	Prospective	Italy	European	Nasopharyngeal secretions	PCR	< 2 years	53.09%

Cao et al. [17] 2023	January 2015 to December 2021.	Cross-sectional	Consecutive	Retrospective	China	Western Pacific	Oropharyngeal secretions	RT-PCR	< 18 years	62.50%
Carlone et al. [18] 2023	October 2021 and February 2022	Longitudinal	Consecutive	Retrospective	Italy	European	Nasal secretions	PCR	< 2 years	56.80%
Chandy et al. [19] 2022	September 2019 to February 2020	Cross-sectional	Consecutive	Prospective	India	South-East Asia	Nasopharyngeal secretions	Multiplex RT-PCR	< 5 years	66.4%
Chu et al. [20] 2022	January 2016 to December 2019	Cross-sectional	Consecutive	Retrospective	China	Western Pacific	Nasopharyngeal secretions	PCR	< 14 years	63.42%
Coma et al. [21] 2022	September 1, 2009 to July 11, 2021	Longitudinal	Consecutive	Retrospective	Spain	European	Not reported	Mixed¥	< 5 years	56.0%
Curatola et al. [22] 2023	September 2021 and March 2022	Cross-sectional	Consecutive	Retrospective	Italy	European	Nasopharyngeal secretions	RT-PCR	0 to 2 years	53.10%
Curatola et al. [23] 2021	February 2020 to February 2021	Cross-sectional	Consecutive	Retrospective	Italy	European	Nasopharyngeal secretions	PCR	< 2 years	72.7%
De Rose et al. [24] 2022	January 1, 2018 to February 28, 2022	Cross-sectional	Consecutive	Retrospective	Italy	European	Nasopharyngeal secretions	Multiplex RT-PCR	< 3 months	Not reported
Diesner-Treiber et al. [25] 2021	November 2020 to April 2021	Longitudinal	Consecutive	Prospective	Austria	European	Nasal secretions	Multiplex PCR	0 to 24 months	59.0%
Esen et al. [26] 2019	November 2015 and April 2016	Cross-sectional	Consecutive	Prospective	Turkey	European	Nasopharyngeal secretions	RT-PCR	< 2 years	52.67%
Faraguna et al. [27] 2023	September and April of 2017–2022	Longitudinal	Consecutive	Retrospective	Italy	European	Nasopharyngeal secretions	RT-PCR	< 1 year	Not reported
Feng et al. [28] 2023	November 1, 2018, to April 30, 2019	Cross-sectional	Consecutive	Prospective	China	Western Pacific	Nasopharyngeal secretions	Multiplex RT-PCR	1 month and 18 years	59.50%
Foley et al. [29] 2022	January 1, 2019 to December 31, 2020.	Longitudinal	Consecutive	Prospective	Australia	Western Pacific	Nasopharyngeal secretions	PCR	< 16 years	51.0%
Giamberardino et al. [30] 2022	March 2017 to September 2018.	Longitudinal	Consecutive	Prospective	Brazil	Americas	Nasopharyngeal secretions	Multiplex RT-PCR	< 2 years	52.9%
Gonapaladeniya et al. [31] 2021	June 2018 to April 2019	Cross-sectional	Consecutive	Prospective	Sri Lanka	South-East Asia	Nasopharyngeal secretions	Multiplex RT-PCR	3 months to < 5 years	39.1%
Grochowska et al. [32] 2022	April 1, 2016 to March 31, 2021.	Cross-sectional	Consecutive	Retrospective	Poland	European	Nasopharyngeal secretions	Mixed¥		Not reported
Guerrero-Del-Cueto et al. [33] 2023	April 2020 to December 2021.	Longitudinal	Consecutive	Ambispective	Spain	European	Not reported	Multiplex PCR	< 2 years	51.50%

Guitart et al. [34] 2022	September 2010 to June 2021	Cross-sectional	Consecutive	Prospective	Spain	European	Nasopharyngeal secretions or BLF	Multiplex PCR		50.8%
Haddadin et al. [35] 2021	December 16, 2019 to April 30, 2020	Longitudinal	Consecutive	Prospective	United States of America	Americas	Nasal secretions	PCR	< 1 year	53.0%
Havdal et al. [36] 2022	2015 to 2018	Cross-sectional	Consecutive	Prospective	Norway	European	Nasopharyngeal secretions	PCR	< 5 years	41.3%
Hu et al. [37] 2023	January 2014 to June 2022	Longitudinal	Consecutive	Retrospective	China	Western Pacific	Nasopharyngeal secretions	Direct immunofluorescence	< 18 years	59.84%
Ippolito et al. [38] 2021	2018-2021	Cross-sectional	Consecutive	Retrospective	Italy	European	Nasopharyngeal secretions	Multiplex PCR	< 2 years	69.0%
Jiang et al. [39] 2023	July 1, 2017 to December 31, 2021	Cross-sectional	Consecutive	Prospective	China	Western Pacific	Mixed*	Multiplex PCR	< 5 years	58.53%
Jiang et al. [40] 2017	January 2015 to December 2015	Cross-sectional	Consecutive	Prospective	China	Western Pacific	Nasopharyngeal secretions	Direct immunofluorescence	1 month to 14 years	57.8%
Jiang et al. [41] 2022	February 1, 2019 to January 31, 2021.	Cross-sectional	Consecutive	Retrospective	China	Western Pacific	Nasopharyngeal secretions	Direct immunofluorescence	1 month to 1 year old	68.2%
Jullien et al. [42] 2020	July 1, 2017 to June 30, 2018	Longitudinal	Consecutive	Prospective	Bhutan	South-East Asia	Nasopharyngeal secretions	Multiplex RT-PCR	< 5 years	57.7%
Kang et al. [43] 2023	October 2019 to July 2022.	Longitudinal	Consecutive	Prospective	India	South-East Asia	Mixed*	Multiplex PCR	1 month to 12 years	Not reported
Kıymet et al. [44] 2021	March 11, 2020 to March 11, 2021.	Cross-sectional	Consecutive	Prospective	Turkey	European	Nasopharyngeal secretions	Multiplex RT-PCR	≤ 18 years	57.5%
Knudsen et al. [45] 2022	2017-2021	Cross-sectional	Consecutive	Ambispective	Norway	European	Nasopharyngeal secretions	PCR	0-18 years	57.5%
Kockuzu et al. [46] 2019	October 2016 to February 2017	Cross-sectional	Consecutive	Retrospective	Turkey	European	Nasal secretions	PCR	1 month to 18 years	63.0%
Komoyo et al. [47] 2021	2015 to 2018	Cross-sectional	Consecutive	Prospective	Central African Republic	African	Nasopharyngeal secretions	Multiplex RT-PCR	< 5 years	51.9%
Korsun et al. [48] 2021	October 2016 to September 2018	Cross-sectional	Consecutive	Prospective	Bulgaria	European	Nasopharyngeal secretions	RT-PCR	< 5 years	55.5%
Kume et al. [49] 2022	January 2018 to December 2021	Cross-sectional	Consecutive	Prospective	Japan	Western Pacific	Nasopharyngeal secretions	RT-PCR	< 15 years	53.7%
Kurskaya et al. [50] 2023	2019-2022	Longitudinal	Consecutive	Prospective	Russia	European	Mixed*	RT-PCR	0–17 years	55.0%

Lagare et al. [51] 2019	2015-2016	Cross-sectional	Consecutive	Prospective	Niger	African	Nasopharyngeal secretions	RT-qPCR	< 5 years	56.4%
Lamrani Hanchi et al. [52] 2022	January 2018 to December 2021	Cross-sectional	Consecutive	Retrospective	Morocco	Eastern Mediterranean	Nasopharyngeal secretions or BLF	Multiplex PCR	< 14 years	58.5%
Li et al. [53] 2023	January 2010 to December 2019	Cross-sectional	Consecutive	Retrospective	China	Western Pacific	Oropharyngeal secretions	RT-PCR	≤14 years	67.94%
Li et al. [54] 2023	From 1 March 2021 through 28 February 2022.	Cross-sectional	Consecutive	Prospective	China	Western Pacific	Oropharyngeal secretions	Multiplex RT-PCR	≤5 years	69.8%
Li et al. [55] 2020	August 2018 to February 2019	Cross-sectional	Consecutive	Prospective	China	Western Pacific	Nasopharyngeal secretions	RT-PCR	< 18 years	67.8%
Li et al. [56] 2022	September 1, 2010 to December 31, 2019.	Cross-sectional	Consecutive	Retrospective	China	Western Pacific	Nasopharyngeal secretions or BLF	Direct immunofluorescence		Not reported
Liu et al. [57] 2018	January 2013 to December 2015	Longitudinal	Consecutive	Prospective	China	Western Pacific	Nasopharyngeal secretions or BLF	Direct immunofluorescence	< 18 years	62.1%
Liu et al. [58] 2021	2019-2020	Cross-sectional	Consecutive	Prospective	China	Western Pacific	Nasopharyngeal secretions or BLF	Direct immunofluorescence	< 18 years	60.0%
Lucion et al. [59] 2022	2019 to 2020	Cross-sectional	Consecutive	Retrospective	Argentina	Americas	Mixed*	Mixed¥	< 2 years	65.0%
Ma et al. [60] 2022	September 1, 2018 to December 31, 2020.	Cross-sectional	Consecutive	Prospective	China	Western Pacific	Throat swab	Antigen	< 12 years	56.7%
Mackenzie et al. [61] 2019	February 10 and December 31, 2015.	Cross-sectional	Consecutive	Prospective	Gambia	African	Oropharyngeal secretions	RT-PCR	2-23 months	54.5%
Maglione et al. [62] 2022	1 July 2021 to 31 March 2022	Longitudinal	Consecutive	Retrospective	Italy	European	Nasopharyngeal secretions	PCR	< 14 years	53.7%
Maruo et al. [63] 2022	2017-2020	Cross-sectional	Consecutive	Retrospective	Japan	Western Pacific	Nasopharyngeal secretions	Antigen	0–14 years	59.2%
Mira-Iglesias et al. [64] 2022	2014 to 2018.	Longitudinal	Consecutive	Prospective	Spain	European	Nasopharyngeal secretions	Multiplex RT-PCR	< 1 year	Not reported
Moreira et al. [65] 2023	August 2020 to September 2021	Longitudinal	Consecutive	Prospective	Brazil	Americas	Nasopharyngeal secretions	RT-PCR	0 to 13 years	53.90%
Nenna et al. [66] 2022	March 9, 2016 to February 28, 2017. March 9, 2020 to February 28, 2021.	Longitudinal	Consecutive	Ambispective	Italy	European	Nasal secretions	RT-PCR	0-18 years	Not reported

Nenna et al. [67] 2022	September 1, 2021 to 15 March 15, 2022	Cross-sectional	Consecutive	Prospective	Italy	European	Nasopharyngeal secretions	Multiplex RT-PCR	< 12 years	52.6%
Ng et al. [68] 2022	April 1, 2021 to October 31, 2021.	Cross-sectional	Consecutive	Retrospective	Malaysia	Western Pacific	Nasopharyngeal secretions or BLF	Multiplex RT-PCR	≤ 12 years	59.5%
Núñez-Samudio et al. [69] 2021	2016-2017	Cross-sectional	Consecutive	Retrospective	Panama	Americas	Oropharyngeal secretions	qPCR	29 days to 14 years	44.7%
Nunziata et al. [70] 2023	November 2021 and April 2022	Longitudinal	Consecutive	Retrospective	Italy	European	Nasal secretions	Multiplex PCR	< 2 years	Not reported
Oumei et al. [71] 2018	January to December 2015	Cross-sectional	Consecutive	Prospective	China	Western Pacific	Oropharyngeal secretions	Direct immunofluorescence nce	6 months to 14 years	Not reported
Pale et al. [72] 2017	January 2015 to January 2016	Cross-sectional	Consecutive	Prospective	Mozambiq ue	African	Nasopharyngeal secretions	RT-PCR	≤ 2 years	51.5%
Pierangeli et al. [73] 2022	December 1, 2018 to April 30, 2019.	Cross-sectional	Consecutive	Prospective	Italy	European	Mixed*	RT-PCR	< 3 years	Not reported
Pogonowska et al. [74] 2022	2020-2021	Cross-sectional	Consecutive	Retrospective	Poland	European	Nasopharyngeal secretions	Antigen	< 2 years	48.0%
Prasad et al. [75] 2019	2012-2015	Longitudinal	Random	Prospective	New Zealand	Western Pacific	Nasopharyngeal secretions	RT-PCR	< 5 years	Not reported
Prasad et al. [76] 2020	2014-2016	Longitudinal	Consecutive	Prospective	New Zealand	Western Pacific	Nasopharyngeal secretions	RT-PCR	< 1 year	Not reported
Presti et al. [77] 2023	October 2021 to 15 March 2023.	Longitudinal	Consecutive	Retrospective	Italy	European	Nasal secretions	PCR	1 month to 18 years	Not reported
Pruccoli et al. [78] 2023	2019-2021	Longitudinal	Consecutive	Prospective	Italy	European	Mixed*	Mixed¥	< 3 years	54.30%
Qiu et al. [79] 2022	January 1, 2019 to December 31, 2021.	Cross-sectional	Consecutive	Retrospective	China	Western Pacific	Oropharyngeal secretions	Direct immunofluorescence nce	28 days to 18 years old.	Not reported
Ramezannia et al. [80] 2021	April 2019 to March 2020.	Cross-sectional	Consecutive	Retrospective	Iran	Eastern Mediterranean	Throat swab	RT-PCR	< 5 years	56.0%
Rha et al. [81] 2020	November 1, 2015 to June 30, 2016	Longitudinal	Consecutive	Prospective	United States of America	Americas	Mixed*	RT-qPCR	< 5 years	56.3%
Roland et al. [82] 2022	1 June to 5 December 2021.	Longitudinal	Consecutive	Prospective	United Kingdom	European	Not reported	PCR	< 2 years	Not reported
Sachs et al. [83] 2023	November 2018 to January 2021	Longitudinal	Consecutive	Prospective	Israel	European	Nasal secretions	PCR	0-18 years	Not reported
Sarkar et al. [84] 2022	December 2013 to March 2016.	Cross-sectional	Consecutive	Prospective	India	South-East Asia	Nasopharyngeal secretions	RT-PCR	> 2 months to > 5 years	65.0%

Savaş Şen et al. [85] 2022	March 2016 to February 2017	Cross-sectional	Consecutive	Retrospective	Turkey	European	Nasopharyngeal secretions	PCR	< 18 years	55.7%
Shen et al. [86] 2022	November 2016 to April 2017	Cross-sectional	Consecutive	Prospective	China	Western Pacific	Throat swab	Direct immunofluorescence		68.86%
Shen et al. [87] 2022	April 16, 2017 to April 15, 2021.	Cross-sectional	Consecutive	Retrospective	Belgium	European	Nasopharyngeal secretions or BLF	RT-PCR	< 6 years	53.6%
Shi et al. [88] 2023	1 September 2021 to 31 December 2022	Cross-sectional	Consecutive	Retrospective	China	Western Pacific	Nasopharyngeal secretions	RT-PCR	< 16 years	56.40%
Singh et al. [89] 2021	January to December 2018	Cross-sectional	Consecutive	Retrospective	India	South-East Asia	Nasopharyngeal secretions	Antigen	< 10 years	70.0%
Sobrinho et al. [90] 2021	April 1, 2017 to August 31, 2018.	Longitudinal	Consecutive	Retrospective	Brazil	Americas	Nasopharyngeal secretions	Mixed¥	< 5 years	63.0%
Song et al. [91] 2022	April 1 to December 31, 2020.	Cross-sectional	Consecutive	Retrospective	United States of America	Americas	Not reported	RT-qPCR	< 18 years	56.0%
Stera et al. [92] 2021	October 1, 2017 to April 30, 2021.	Cross-sectional	Consecutive	Retrospective	Italy	European	Not reported	RT-PCR	< 1 year	Not reported
Tang et al. [93] 2022	January 2018 to December 2020.	Cross-sectional	Consecutive	Retrospective	China	Western Pacific	Nasopharyngeal secretions or BLF	Direct immunofluorescence	1 month to 15 years	57.63%
Thongpan et al. [94] 2021	July–December 2020	Longitudinal	Consecutive	Prospective	Thailand	South-East Asia	Nasopharyngeal secretions	RT-PCR	≤5 years	56.0%
Thwaites et al. [95] 2018	2016-2017	Longitudinal	Consecutive	Prospective	United Kingdom	European	Nasopharyngeal secretions	qPCR	2 weeks to 2 years	Not reported
Trenholme et al. [96] 2021	2015-2020	Cross-sectional	Consecutive	Retrospective	New Zealand	Western Pacific	Nasopharyngeal secretions	PCR	< 2 years	Not reported
Tsergouli et al. [97] 2019	2016-2018	Cross-sectional	Consecutive	Prospective	Greece	European	Throat swab	RT-PCR	< 2 years	53.5%
Valley-Omar et al. [98] 2022	January 2012 to December 2015	Cross-sectional	Consecutive	Prospective	South Africa	African	Nasopharyngeal secretions	Multiplex RT-PCR	3 months to < 5 years	57.4%
Varela et al. [99] 2022	May to November 2020.	Longitudinal	Consecutive	Prospective	Brazil	Americas	Nasopharyngeal secretions	RT-PCR	2 months to 18 years	44.4%
Virant et al. [100] 2023	From January 2018 to December 2021	Cross-sectional	Consecutive	Prospective	Slovenia	European	Nasopharyngeal secretions	RT-PCR	0 to 18 years	56.30%
Vittucci et al. [101] 2021	September 2018 to February 2021.	Cross-sectional	Consecutive	Retrospective	Italy	European	Nasopharyngeal secretions	RT-PCR	< 18 years	Not reported

Wang et al. [102] 2019	January to June 2018	Cross-sectional	Consecutive	Prospective	China	Western Pacific	Sputum	Multiplex PCR	< 13 years	61.1%
Watanabe et al. [103] 2022	March to August 2019.	Cross-sectional	Consecutive	Prospective	Brazil	Americas	Nasopharyngeal secretions	RT-PCR	< 2 years	65.8%
Weldetsadik et al. [104] 2021	June 2018 to May 2019.	Cross-sectional	Consecutive	Prospective	Ethiopia	African	Nasopharyngeal secretions	Antigen	29 days to 6 months	65.0%
Wen et al. [105] 2020	January 1, 2008, to December 31, 2017.	Cross-sectional	Consecutive	Retrospective	China	Western Pacific	Nasopharyngeal secretions	Direct immunofluoresce nce	< 18 years	Not reported
Windsor et al. [106] 2022	December 2018 to December 2019.	Longitudinal	Random	Prospective	United States of America	Americas	Nasopharyngeal secretions	Multiplex PCR	1 month to 18 years	56.0%
Xia et al. [107] 2023	January 2017 to December 2021	Cross-sectional	Consecutive	Retrospective	China	Western Pacific	Not reported	Antigen	≤ 7 years	59.6%
Xu et al. [108] 2022	February 2019 to January 2021.	Cross-sectional	Consecutive	Retrospective	China	Western Pacific	Nasopharyngeal secretions or BLF	Multiplex PCR	< 18 years	60.76%
Yan et al. [109] 2023	January 2018 to December 2019	Longitudinal	Consecutive	Prospective	China	Western Pacific	Sputum	Multiplex PCR	< 15 years	57.90%
Ye et al. [110] 2022	January 2019 to December 2020	Cross-sectional	Consecutive	Retrospective	China	Western Pacific	Nasopharyngeal secretions or BLF	Direct immunofluoresce nce	< 18 years	57.32%
Zhao et al. [111] 2019	December 2017 to March 2018	Cross-sectional	Consecutive	Prospective	China	Western Pacific	Nasopharyngeal secretions	Direct immunofluoresce nce	< 5 years	Not reported
Zhao et al. [112] 2022	January 2019 to March 2020	Longitudinal	Consecutive	Prospective	China	Western Pacific	Sputum	Multiplex RT-PCR	< 13 years	61.04%

¥Mixed assays: Antigen testing/RT-PCR; or Direct immunofluorescence/RT-qPCR/antigen testing; or Direct immunofluorescence/RT-PCR; or Indirect immunofluorescence/PCR. $The indicated diagnostic technique is according to what is referred to by the authors. *Mixed specimens: Nasopharyngeal secretions and nasal secretions; or Nasal and throat secretions. Abbreviations: WHO, World Health Organization; BLF, Bronchoalveolar lavage fluid.

Table S7. Definitions of lower respiratory tract infection according to each study.

Study	Definition of lower respiratory tract infection
Agca et al. [1]	Children with nasal congestion, sneezing, sore throat, rhin-orrhea, nasal discharge, and cough.
Akkoc et al. [2]	LRTIs were diagnosed clinically and defined as having at least two of the complaints following fever, cough or wheezing and crackles, and/or rhonchi on physical examination.
Al-Zayadneh et al. [3]	Children hospitalized with febrile illness (>38.0°C) and were diagnosed as LRTI if they presented with cough, difficulty breathing, and signs indicative of LRTI such as tachypnea, retraction, grunting, flaring, and auscultatory findings such as wheezing or crackles.
Assane et al. [4]	Upper or lower airway infection.
Baldassarre et al. [5]	Classification of Diseases, Ninth Revision, Clinical Modification (ICD-9-CM): 079.6 (RSV infection), 466.1 (acute bronchiolitis), 466.11 (acute bronchiolitis due to RSV), and 466.19 (acute bronchiolitis due to pathogens other than RSV).
Barbati et al. [6]	LRTI in hospitalized children
Bedir Demirdağ et al. [7]	LRTI in hospitalized children
Ben-Shimol et al. [8]	Community-acquired Alveolar Pneumonia: This includes hospitalized children less than 18 months old (2–17 months) with radiographically confirmed alveolar pneumonia as per the World Health Organization pneumonia protocol for radiography reading. Bronchiolitis: This includes hospitalized children less than 18 months old (2–17 months) with acute respiratory infection judged by the clinician to have bronchiolitis (including dyspnea, wheezing, and negative chest radiography for alveolar infiltrates). Since ruling out pneumonia in children with clinical bronchiolitis is difficult, we further excluded those with peripheral white blood cell (WBC) counts of ≥20,000/mm3 and rectal temperature ≥39°C.
Berdah et al. [9]	ICD-10 code diagnoses of bronchiolitis (J21.0, J21.1, J21.8 and J21.9).
Bermúdez Barrezueta et al. [10]	Bronchiolitis was definied as the first episode of respiratory distress coursing with wheezing and/or cracklingrales and is preceded by catarrhal clinical symptoms of the upperairways
Bhardwaj et al. [11]	Patients with a history of cough that began during the past seven days and that required overnight hospitalization. SARI was defined in babies < 2 months of age as a physician diagnosis consistent with an acute lower respiratory infection (pneumonia, bronchitis, bronchiolitis, sepsis) necessitating overnight hospitalization.
Brisca et al. [12]	International Classification of Diseases 9th review (ICD-9) diagnosis of “acute bronchiolitis” (code 466.19)
Buendia et al. [13]	Children admitted to the pediatric ward diagnosed with bronchiolitis, according to the colombian national clinical guideline of bronchiolitis (first wheezing episode younger than 24 monthsof age).
Buendía et al. [14]	RSV LRTI (ICD-10 code: J21.0) according to the National clinical guideline of bronchiolitis (first wheezing episode younger than 24 months of age).

Camporesi et al. [15]	Bronchiolitis was defined as the presence of respiratory distress and signs of lower respiratory tract infection [1] (e.g., cough, tachypnoea or chest recession, and wheeze or crackles on chest auscultation) or a first episode of acute viral wheeze.
Camporesi et al. [16]	Clinical diagnosis of bronchiolitis (e.g., children with cough, tachypnoea or chest recession, and wheeze or crackles on chest auscultation) or a first episode of acute viral wheeze.
Cao et al. [17]	Severe acute respiratory infection (SARI) case definition is based on the 2011 WHO criteria for children > 5 years, characterized by a sudden onset of fever (> 38ºC) along with cough or sore throat, in the absence of an alternative diagnosis, coupled with shortness of breath or difficulty in breathing, necessitating hospital admission. For patients < 5 years, SARI is identified by cough or difficulty in breathing with a respiratory rate exceeding 40 breaths/min (ages 1–5 years) or 50 breaths/min (ages 2–12 months). Other indicators include difficulty in drinking or breastfeeding, vomiting, convulsions, lethargy, unconsciousness, chest indrawing, or stridor in a calm child, all requiring hospital admission.
Carlone et al. [18]	Bronchiolitis
Chandy et al. [19]	WHO criteria for pneumonia with tachypnea or wheeze with or without hypoxia.
Chu et al. [20]	LRTI is defined by symptoms of cough or difficult breathing and any of the following: lower chest wall in-drawing, fast breathing in accordance with the WHO clinical criteria. Clinical syndromes of bronchitis, bronchiolitis, pneumonia, and acute asthma exacerbations are included in LRTI.
Coma et al. [21]	Children with clinical diagnosis of suspected RSV infection according to ICD-10 codes registered in the EHR: J21 (Acute bronchiolitis), J21.0 (Acute bronchiolitis due to RSV), J21.9 (Acute bronchiolitis, unspecified), and B97.4 (Respiratory syncytial virus as the cause of diseases classified elsewhere).
Curatola et al. [22]	Acute bronchiolitis
Curatola et al. [23]	Diagnosis of acute bronchiolitis admitted to hospital.
De Rose et al. [24]	Bronchiolitis was characterized by coryza, persistent cough, and respiratory distress in the presence of wheezing or crackles during chest auscultation.
Diesner-Treiber et al. [25]	Infants with with at least one of the following symptoms: rhinitis, nasal congestion, cough, sore throat or fever of unknown origin (e.g., without gastroenteritis or urinary tract infection) with a maximum duration of one week.
Esen et al. [26]	Lower tract symptoms were classified by the presence of fever, tachypnoea, rhonchi with a respiratory rate as per minute, wheezing, focal or diffuse crackles or decreased vesicular sounds on auscultation and retraction at the end of expiration. Pneumonia was diagnosed according to both clinical or radiologic manifestations in the patient.
Faraguna et al. [27]	Clinical diagnosis of bronchiolitis (e.g., children with rhinorrhea, cough, crackles, wheezing, dyspnea, polypnea, feeding difficulties, apnea, lethargy).
Feng et al. [28]	Patients diagnosed with CAP by meeting any of the following criteria were included: Increased symptoms of cough, expectoration, or preexisting respiratory diseases and purulent sputum with or without chest pain; fever; signs of pulmonary consolidation and/or audible and moist rales; white blood cell (WBC) count >10 × 10^9/L or <4 × 10^9/L, with or without nucleus left; chest X-ray showing patchy, invasive, or interstitial changes with or without pleural effusion.

Foley et al. [29]	ICD 10th edition Australian Modification for LRTI (J9–J18, J20 and J22) and bronchiolitis (J21).
Giamberardino et al. [30]	LRTI in hospitalized children
Gonapaladeniya et al. [31]	Community-Acquired Pneumonia was defined as the presence of fever, cough, abnormal chest signs (localized crackles or increased vocal resonance or reduced breath sounds), and tachypnoea.
Grochowska et al. [32]	LRTI based on ICD-10 code (1) viral infections (J12, J20 , J21 , J40), including bronchiolitis (J21); (2) influenza or flu-like infection (J10 , J11); (3) bacterial infections (J13; J14; J15 , J16 , J18, J20.
Guerrero-Del-Cueto et al. [33]	ICD-10 code "J21—Acute bronchiolitis"
Guitart et al. [34]	Clinical findings of sever bronchiolitis according to Bronchiolitis Score of Sant Joan de Déu (BROSJOD) and the Pediatric Risk of Mortality Score III scale (PRISM III).
Haddadin et al. [35]	Infants with fever and/or respiratory symptoms (ie, wheezing, crackles, rales, diminished breath sounds, shortness of breath cough, earache, nasal congestion, rhinorrhea, and/or sore throat) within 14 days of symptom onset.
Havdal et al. [36]	Diagnosis with RSV-specific ICD-10 codes (J20.5; J21.0; J12.1; B97.4).
Hu et al. [37]	LRTI included bronchiolitis and pneumonia.
Ippolito et al. [38]	Diagnosis of bronchiolitis or community acquired pneumonia which was confirmed by an expert physician.
Jiang et al. [39]	Severe community-acquired pneumonia cases were defined as meeting any of the following criteria: Poor general condition, Disturbance of consciousness, Hypoxemia: cyanosis, rapid respiration, RR ≥ 70 breaths/min (infant), RR ≥ 50 breaths/min (1-year-old or older), assisted breathing (groaning, nasal fanning, trisplegia), intermittent apnea, oxygen saturation <92%, Hyperthermia, persistent hyperthermia for more than 5 days, Dehydration or food refusal, Chest radiograph or CT ≥ 2/3 side lung infiltration, lobar lung infiltration, pleural effusion, pneumothorax, atelectasis, lung necrosis, and lung abscess.
Jiang et al. [40]	LRTI was considered as fever (defined as body temperature ≥ 38 °C), and had clinical (chest retractions, tachypnea, nasal flaring, hypoxia, or abnormal auscultatory findings) and radiologic evidence of pneumonia.
Jiang et al. [41]	Clinical evidence of bronchiolitis (tachypnea, wheeze, prolonged expiratory phase, and crackles on auscultation).
Jullien et al. [42]	WHO-defined pneumonia
Kang et al. [43]	Clinical diagnosis of acute lower respiratory tract infections (ALRTIs), including Acute Viral Bronchiolitis (AVB), pneumonia, and Acute Respiratory Distress Syndrome (ARDS), was established, and these cases were confirmed as negative for SARS-CoV-2 during the pandemic.
Kıymet et al. [44]	LRTI in hospitalized children
Knudsen et al. [45]	ICD-10 diagnoses; J21 Acute bronchiolitis, J12 Viral pneumonia and J13 + J14 + J15 Bacterial pneumonia.
Kockuzu et al. [46]	Respiratory tract infections in patients hospitalized in pediatric intensive care.
Komoyo et al. [47]	History of fever ≥38°c and cough with onset within the last 10 days requiring hospitalization.

Korsun et al. [48]	ARIs were defined according to the ECDC.
Kume et al. [49]	LRTI in hospitalized children
Kurskaya et al. [50]	Children who had at least one of the systemic symptoms (fever, headache, myalgia, or malaise) and one of the respiratory symptoms (cough, rhinorrhea, nasal congestion, sore throat, shortness of breath, lung auscultation abnormalities, chest pain).
Lagare et al. [51]	ARI was defined as a hospitalized child aged younger than 5 years with onset of fever 38°C or higher and cough within 10 days prior to admission and at least one of the following signs: inability to drink or breastfeed, lethargy, vomiting, convulsions, nasal flaring, chest indrawing, stridor in a calm child, or tachypnea.
Lamrani Hanchi et al. [52]	As a diagnosis of severe bronchiolitis, pneumonia, respiratory distress, influenza syndrome in immunocompromised children, or a clinical suspicion of pertussis that required a hospitalization in the pediatric departments.cion
Li et al. [53]	Pneumonia cases were defined according to the Subspecialty Group of Respiratory Diseases, The Society of Pediatrics, Chinese Medical Association; Editorial Board, Chinese Journal of Pediatrics. Guidelines for management of community-acquired pneumonia in children (the revised edition of 2013) (I). Zhonghua Er Ke Za Zhi 2013;51:745-52.
Li et al. [54]	ALRTI was defined as the presence of signs and symptoms of respiratory tract infection (i.e., fever ≥38 ◦C, coughing, rhinorrhea, oropharyngeal hyperemia, and swelling of tonsils), lower respiratory signs (tachypnea, dyspnea, retractions, wheezing, or rales upon auscultation), and lung infiltrates indicated by chest radiography.
Li et al. [55]	Children with ≤ 3 days of fever (temperature ≥ 37.5 °C), and with cough, sputum, throat sore, dyspnea and/or other acute respiratory tract infection symptoms.
Li et al. [56]	Community-acquired pneumonia
Liu et al. [57]	LRTI was considered if children have at least 1 of the following manifestations: confirmed fever (38°C), abnormal white blood cell (WBC) differential, leukocytosis (a WBC count of ＞10,000/mL) or leukopenia (a WBC count of＜4,000/mL), and chills; and at least 1 of the following signs/symptoms of LRTI: sputum, shortness of breath, lung auscultation abnormality (rale or wheeze), tachypnea, and chest pain.
Liu et al. [58]	Children were considered to have an LRTI if they had at least one of the following manifestations of acute infection: confirmed fever (38 °C), abnormal white blood cells differential such as leukocytosis or leukopenia, and chills; at least one of the following signs/symptoms of respiratory tract infection: cough, sputum, shortness of breath, lung auscultation abnormality (rale or wheeze), tachypnea, and chest pain.
Lucion et al. [59]	Bronchiolitis: first wheezing event associated with clinical evidence of viral infection in children younger than 2 years. Pneumonia: acute infection of the lung parenchyma with clinical signs of invasion of the alveolar space.
Ma et al. [60]	LRTI in hospitalized children
Mackenzie et al. [61]	LRTI was definied as cough or difficulty breathing for 14 days or less and one or more of the following: raised respiratory rate for age, lower chest wall indrawing, nasal flaring, grunting, oxygen saturation <92%, altered consciousness, prostration, seizures, dull chest percussion note, coarse crackles, or bronchial breathing.

Maglione et al. [62]	ARI was defined as an acute respiratoryi llness with a history of fever or measured fever of ≥ 38 °C and cough, with onset within the past 10 days, requiring hospitalization. Also included patients without fever, but with cough, nasal obstruction, nasal flaring, tachypnea, and/or hypoxia, fulfilling the clinical definition of bronchiolitis
Maruo et al. [63]	LRTI in hospitalized children
Mira-Iglesias et al. [64]	The onset of symptoms that led to hospitalization was required to be 7 days prior to admission, and patients had to be in hospital between 8 and 48 h before their inclusion in the study. Infants with acute respiratory infection according to ICD-10 code; LRTI (J09-J22), bronchiolitis (J21), and pneumonia (J12-J18).
Moreira et al. [65]	Acute respiratory symptoms and were admitted to the infirmary or pediatric intensive care units (ICU), severe acute respiratory syndrome (SARS) who were seen at integrated health care centers or other inpatient services.
Nenna et al. [66]	Bronchiolitis, asthmatic bronchitis and pneumonia.
Nenna et al. [67]	The respiratory score included the child's color, respiratory rate, presence of wheeze, use of accessory muscles, mental status, and oxygen saturation.
Ng et al. [68]	As the presence of cough or difficulty breathing with signs of severe respiratory distress, requiring high dependency care in the pediatric respiratory ward or admission to the pediatric ICU.
Núñez-Samudio et al. [69]	Children with respiratory symptoms, and the following admission diagnoses from the ICD-10 [1]: suspected influenza due to unidentified influenza virus with other respiratory manifestations (J11.1), pneumonia, unspecified organism (J18), acute bronchiolitis (J21) and unspecified asthma with acute exacerbation (J45.901).
Nunziata et al. [70]	Bronchiolitis was defined according to the Guideline Development Group and Technical Team. Bronchiolitis in children: Summary of NICE guidance. BMJ 2015, 350, h2305.
Oumei et al. [71]	Pneumonia cases were defined using the Child Community-Acquired Pneumonia Guidelines of the Chinese Medicine Association.
Pale et al. [72]	SARI was defined according to WHO definition, as any severe patient (requiring hospitalization) with acute symptoms (within the last 10 days of onset of disease); and respiratory infection (defined as the presence of cough, but in some sites defined as cough or shortness of breath).
Pierangeli et al. [73]	ECDC definition of ILI, i.e., fever (temperature ≥ 37.8 °C), with at least one systemic symptom (i.e., headache, asthenia, myalgia), and at least one respiratory symptom (i.e., cough, dyspnea, acute pharyngitis).
Pogonowska et al. [74]	Children with a diagnosis of bronchiolitis, pneumonia or obstructive bronchitis of RSV aetiology were included in the study.
Prasad et al. [75]	ARI was defined based on ICD-10 code such as B974, J121, J210, and J205.
Prasad et al. [76]	ARI-associated if infants presented with at least one of the following signs or symptoms: apnea, cyanosis, shortness of breath, cough, wheeze, increased work of breathing, stridor, and fever.
Presti et al. [77]	Respiratory illness

Pruccoli et al. [78]	Bronchiolitis and/or pneumonia
Qiu et al. [79]	Fever and lower respiratory tract symptoms, such as tachypnea, nonproductive cough, wheeze, and increased breath sound such as rales or wheezes or chest imaging findings (bronchitis or pneumonia) to be met, including the diagnosis of pneumonia, bronchitis, bronchiolitis and prolonged pneumonia.
Ramezannia et al. [80]	Children with fever, wheezing, cough, vomiting, diarrhea, pneumonia, runny nose, cyanosis, tachypnea, and dyspnea.
Rha et al. [81]	LRTI in hospitalized children
Roland et al. [82]	Infant diagnosed as bronchiolitis, lower respiratory tract infection or first episode of acute wheeze.
Sachs et al. [83]	LRTI was defined when wheezing or increased breath sound over the lungs or signs of inflammation over the lung parenchyma in chest X-ray were detected.
Sarkar et al. [84]	LRTI such as bronchiolitis, pneumonia, and wheezing illness, attending a pediatric emergency room and intensive care unit were included.
Savaş Şen et al. [85]	Patients < 2 years with mild fever and wheezing, sibilant rhonchi and rals on auscultation, normal or slightly elevated white blood cell count in laboratory tests, and increased aeration and/or peribronchial infiltration on chest X-ray were diagnosed with acute bronchiolitis. Patients with consolidation on chest X-rays were defined as pneumonia. Patients > 2 years and admitted to the hospital for the first time with a wheezing episode were evaluated as virus-induced wheezing.
Shen et al. [86]	Patients with respiratory symptoms such as cough, rhinorrea, nasal congestion, sneezing and signs of LRTI such as respiratory distress, crackles, wheezing, or chest-X ray infiltrates.
Shen et al. [87]	LRTI in hospitalized children
Shi et al. [88]	All children who were admitted to the Children's Hospital of Soochow University were hospitalized with symptoms of respiratory distress.
Singh et al. [89]	LRTI in hospitalized children
Sobrinho et al. [90]	The clinical diagnosis of LRTI was defined by the presence of cough, tachypnea, respiratory distress with prolonged expiratory time and wheezing or crackles on auscultation.
Song et al. [91]	LRTI in hospitalized children
Stera et al. [92]	Bronchiolitis was defined by the ICD-9 (466.11 and 466.19). Hospitalization criteria were considered according to the most recently updated Italian Inter-Society Consensus for bronchiolitis.
Tang et al. [93]	LRTI in hospitalized children
Thongpan et al. [94]	Influenza-like illness
Thwaites et al. [95]	LRTI diagnosis was based on standard clinical criteria (cough, tachypnea, wheezes and crackles on auscultation, and hyperinflation).
Trenholme et al. [96]	LRTI (codes J22, A37, J47, J10.0 J10.1 J11.1, J12–16, J20, J21, and J18 from the ICD-10)
Tsergouli et al. [97]	Bronchiolitis

Valley-Omar et al. [98]	Children with physician-diagnosed LRTI, including bronchitis, bronchiolitis, pneumonia, and pleural effusion.
Varela et al. [99]	The main inclusion criteria were the presence of at least one sign or symptom suggestive of COVID-19 (cough, fever, or sore throat).
Virant et al. [100]	Lower respiratory tract infections.
Vittucci et al. [101]	LRTI in hospitalized children
Wang et al. [102]	LRTI was diagnosed based on the clinical and radiologic findings, including pneumonia, bronchitis and bronchiolitis.
Watanabe et al. [103]	Clinical diagnosis of bronchiolitis and no expected discharge in the next 24 hours.
Weldetsadik et al. [104]	Severe LRTI: Acute onset (less than 7 days) with respiratory symptoms (cough, dyspnea, wheezing, tachypnea, and grunting) requiring hospitalization.
Wen et al. [105]	LRTI was considered as bronchitis, bronchiolitis, or pneumonia.
Windsor et al. [106]	LRTI in hospitalized children
Xia et al. [107]	Clinical signs and symptoms of respiratory infection such as fever, pneumonia, bronchitis, capillary bronchitis, tonsillitis, and pharyngitis.
Xu et al. [108]	LRTI was definied as presenting with at least one of the following signs/symptoms: fever, cough, sputum, shortness of breath, lung auscultation abnormality (rale or wheeze), tachypnea, and chest pain.
Yan et al. [109]	All patients were examined and diagnosed clinically by the pediatrician, and the clinical criteria of pneumonia, bronchitis, and bronchiolitis are defined according to World Health Organization guidelines of common childhood illnesses including respiratory symptoms and chest radiographic findings.
Ye et al. [110]	Fever (> 37.5 °C); one or more respiratory symptoms within 14 days of onset (cough, sore throat, sputum, shortness of breath, lung auscultation abnormality (rale or wheeze), tachypnea, and chest pain).
Zhao et al. [111]	ARI was mainly presenting with bronchiolitis or pneumonia.
Zhao et al. [112]	1. Acute infection: fever or hypothermia , or an abnormal WBC distribution, 2. Respiratory tract symptom(s): at least one of cough, runny nose, wheezing/gasping, pharyngeal congestion, enlargement of lymph nodes, enlargement of tonsils, moist rales/moist crackles, dry rales/rhonchi, coarse breath sounds, shortness of breath, dyspnoea, or cyanosis, temporary sleep apnoea, or hypoxemia (oxygen saturation < 92%). 3. Radiological manifestations. Patients were enrolled if they met both criteria 1 and 2, criterion 3, or all three criteria.

Abbreviations: ARI, Acute respiratory infections; ECDC, European Centre for Disease Prevention and Control; ICD-10, International Classification of Diseases-10th revision; LRTI, Lower respiratory tract infections; RSV, Respiratory syncytial virus; SARI, Severe acute respiratory infection; WHO, World Health Organization.

Figure S1. Respiratory syncytial virus (RSV) prevalences according to RSV season.

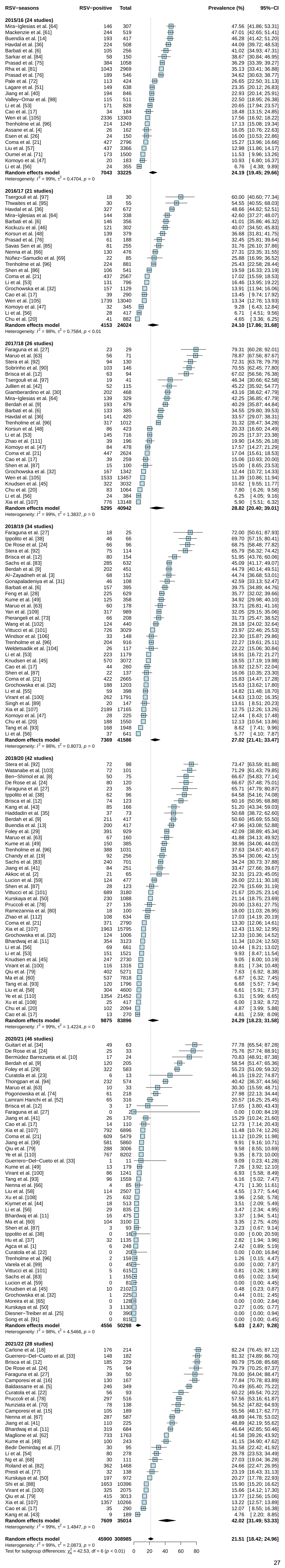

Table S8. Sensitivity analysis of pooled respiratory syncytial virus (RSV) prevalence in children diagnosed with RSV-related lower respiratory tract infection by molecular assays.

Groups	Studies (n)	RSV-positive (n)	Total (n)	Pooled prevalence (95%CI)	Q-value	I^2 (%)	P-Value Heterogeneity	P-Value Egger test	P-Value subgroup difference
**Overall**	85	22523	103157	24.96 [20.77; 29.69]	10441.27	98.5	< 0.0001	0.0350	NA
**Subgroup analyses**									
**Period**									< 0.0001
Pre-pandemic (2015/20)	59	15483	65335	30.45 [26.68; 34.49]	5723.37	98.20	< 0.0001	0.0243	
Lockdown (2020/21)	31	1610	16828	3.82 [1.53; 9.22]	1767.6	98.30	< 0.0001	0.2778	
Post-lockdown (2021/22)	24	5430	20994	44.29 [32.61; 56.63]	2586.72	99.10	< 0.0001	0.0012	
**Seasons**									< 0.0001
2015/16	17	3259	10642	27.90 [22.59; 33.91]	450.87	96.50	< 0.0001	0.2012	
2016/17	16	1686	6330	28.52 [20.61; 38.02]	556	97.30	< 0.0001	0.4548	
2017/18	18	2150	9574	32.47 [23.22; 43.33]	975.65	98.30	< 0.0001	0.0953	
2018/19	26	4259	17522	32.06 [25.38; 39.56]	1050.15	97.60	< 0.0001	0.0273	
2019/20	28	4129	21267	30.57 [21.84; 40.95]	2381.7	98.90	< 0.0001	0.1051	
2020/21	31	1610	16828	3.82 [1.53; 9.22]	1767.6	98.30	< 0.0001	0.2778	
2021/22	24	5430	20994	44.29 [32.61; 56.63]	2586.72	99.10	< 0.0001	0.0012	
**WHO Region**									0.0204
African	6	811	3485	19.13 [13.52; 26.35]	223.84	96.40	< 0.0001	0.2012	
Americas	9	1409	4832	6.45 [0.56; 45.74]	78.1	89.80	< 0.0001	0.4548	
Eastern Mediterranean	3	151	568	26.53 [15.66; 41.25]	33.53	94.00	< 0.0001	0.1051	
European	42	11125	45638	31.46 [24.26; 39.68]	5534.17	98.40	< 0.0001	0.0086	
South-East Asia	7	1263	5840	26.25 [14.52; 42.72]	747.12	98.80	< 0.0001	0.5942	
Western Pacific	18	7764	42794	17.88 [14.24; 22.22]	2852.47	98.60	< 0.0001	0.962	
**Age**									< 0.0001
< 2 years	28	5974	15299	46.55 [37.88; 55.43]	1829.49	96.90	< 0.0001	0.0004	
< 5 years	18	4044	16685	26.90 [21.99; 32.46]	1411.69	98.40	< 0.0001	0.7802	
< 10 years	2	609	1845	23.89 [14.51; 36.74]	79.58	91.20	< 0.0001	NA	
< 15 years	15	4160	21637	18.03 [12.65; 25.04]	2178.11	98.80	< 0.0001	0.2582	
< 18 years	21	7687	47628	10.44 [6.77; 15.76]	2478.92	98.30	< 0.0001	0.1767	
Not reported	1	49	63	77.78 [65.90; 86.37]	0	NA	NA	NA	
**Design**									0.3220

Cross-sectional	54	15523	82059	23 [19.00; 28.19]	7535.17	98.50	< 0.0001	0.0213	
Longitudinal	31	7000	21098	29 [19.31; 41.33]	1328.51	96.60	< 0.0001	0.6375	
**Timing of data collection**									0.3410
Ambispective	3	1432	11690	11.57 [3.45; 32.42]	623.24	98.90	< 0.0001	NA	
Prospective	49	10318	41237	25.49 [19.89; 32.03]	3854.24	98.20	< 0.0001	0.6615	
Retrospective	33	10773	50230	26.32 [20.15; 33.57]	5223.79	98.50	< 0.0001	0.0461	
**Risk of bias**									0.0516
Low risk	52	14642	66588	28.28 [22.64; 34.69]	7966.97	98.70	< 0.0001	0.0415	
Moderate risk	33	7881	36569	19.59 [14.27; 26.30]	2433.69	97.70	< 0.0001	0.4702	
**Diagnostic technique$**									0.4697
Multiplex PCR	14	2083	10831	27.32 [14.57; 45.30]	1756.55	98.70	< 0.0001	0.0439	
Multiplex RT-PCR	18	2215	7385	30.89 [22.03; 41.42]	828.23	97.00	< 0.0001	0.349	
PCR	16	7247	32319	24.91 [16.89; 35.12]	3648	99.10	< 0.0001	0.8591	
qPCR	2	52	140	38.93 [21.17; 60.20]	11.32	91.20	0.0008	NA	
RT-PCR	31	9193	46668	22.15 [16.57; 28.95]	3348.97	98.00	< 0.0001	0.0936	
RT-qPCR	4	1733	5814	17.82 [3.59; 55.79]	50.62	88.10	< 0.0001	NA	
**Sample type**									0.3386
Mixed specimens*	5	2643	13556	19.57 [8.56; 38.74]	1156.71	99.10	< 0.0001	0.7849	
Nasal secretions	9	1209	3493	21.39 [6.94; 49.83]	263.44	95.80	< 0.0001	0.5521	
Nasopharyngeal secretions	49	14192	65456	27.19 [21.61; 33.60]	6453.98	98.60	< 0.0001	0.0303	
Nasopharyngeal secretions or BLF	6	803	3384	21.68 [12.47; 34.97]	378.02	96.80	< 0.0001	0.0862	
Not reported	5	998	3167	31.87 [5.64; 78.54]	469.54	98.50	< 0.0001	NA	
Oropharyngeal secretions	5	1385	7585	17.25 [13.26; 22.14]	379.74	96.30	0.0004	0.3843	
Sputum	3	549	2063	25.23 [18.38; 33.58]	44.03	95.50	< 0.0001	NA	
Throat secretions	3	744	4453	23.95 [9.93; 47.36]	506.84	99.00	< 0.0001	NA	

$The indicated diagnostic technique is according to what is referred to by the authors. *Mixed specimens: Nasopharyngeal secretions and nasal secretions; or Nasal and throat secretions. Abbreviations: WHO, World Health Organization; BLF, Bronchoalveolar lavage fluid; NA, Not applicable.

Figure S2. Funnel plot of sensitivity analysis of pooled respiratory syncytial virus (RSV) prevalence in children diagnosed with RSV-related lower respiratory tract infection by molecular assays.

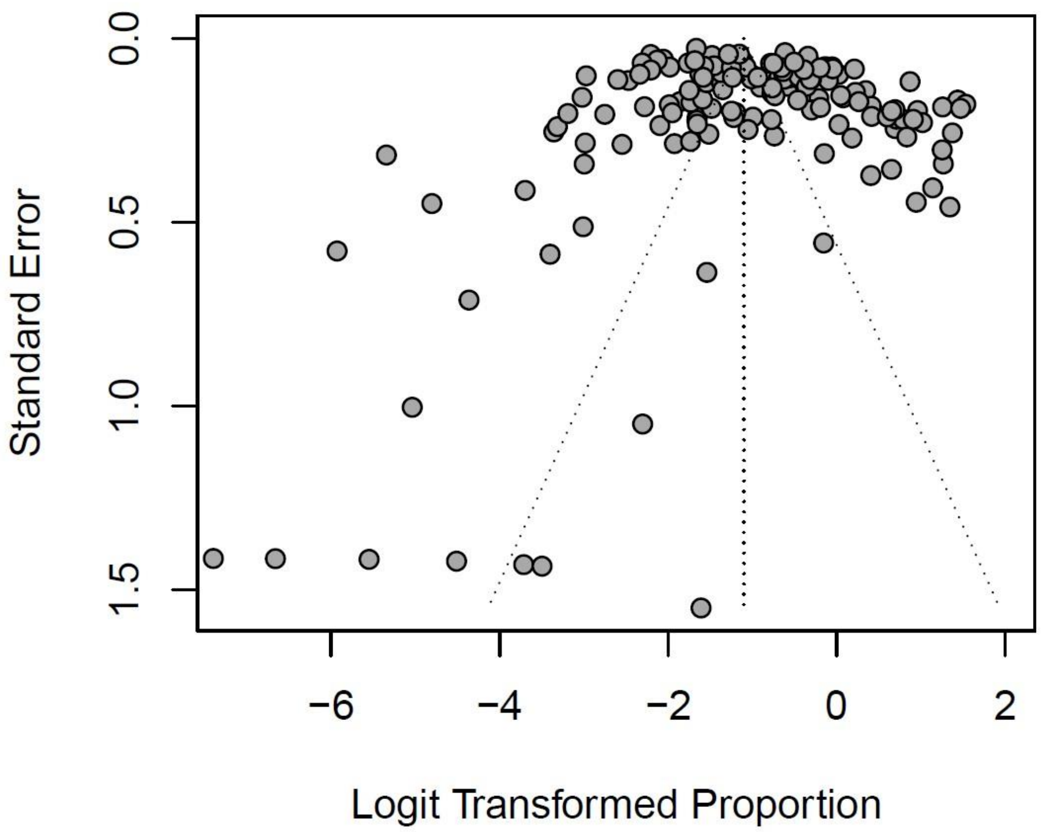

Table S9. Univariable meta-regression model for the prevalence of the respiratory syncytial virus in hospitalized children with lower respiratory tract infection.

	Coefficient [95%-CI]	P-Value	R^2
**Period**			16.47%
Lockdown [2020-2021]	Reference		
Pre-pandemic [2015-2020]	0.1526 [0.0856, 0.2195]	< 0.0001	
Post-lockdown [2021-2022]	0.3005 [0.2059, 0.3951]	< 0.0001	
**Seasons**			17.03%
2020/21	Reference		
2015/16	0.1224 [0.0237; 0.2211]	0.0150	
2016/17	0.1328 [0.0292; 0.2365]	0.0120	
2017/18	0.1874 [0.0908; 0.2841]	0.0001	
2018/19	0.1588 [0.0697; 0.2479]	0.0005	
2019/20	0.1533 [0.0693; 0.2374]	0.0004	
2021/22	0.3005 [0.2062; 0.3948]	< 0.0001	
**WHO Region**			14.39%
Western Pacific	Reference		
African	0.0258 [-0.1070, 0.1586]	0.7035	
Americas	0.1283 [0.0132, 0.2433]	0.029	
Eastern Mediterranean	0.0947 [-0.1401, 0.3295]	0.429	
European	0.1780 [0.1188, 0.2373]	< 0.0001	
South-East Asia	0.1191 [-0.0088, 0.2469]	0.0679	
**Age**			34.54%
< 2 years	Reference		
< 5 years	-0.1883 [-0.2620, -0.1145]	< 0.0001	
< 10 years	-0.2646 [-0.3669, -0.1623]	< 0.0001	
< 15 years	-0.2398 [-0.3103, -0.1692]	< 0.0001	
< 18 years	-0.3141 [-0.3781, -0.2501]	< 0.0001	
**Age [months]**			22.29%
Months	-0.0079 [-0.0105, -0.0054]	< 0.0001	
**Type of assay**			
Immune assays¥	Reference		7.68%
Molecular assays¥¥	0.1365 [0.0669, 0.2060]	0.0001	
Mixed assays¥¥¥	0.0175 [-0.1020, 0.1369]	0.7745	
**Diagnostic technique$**			
Direct immunofluorescence	Reference		9.72%
Antigen testing	0.0591 [-0.0723, 0.1905]	0.3783	
Mixed assays¥¥¥	0.0357 [-0.0892, 0.1606]	0.575	
Multiplex PCR	0.2053 [0.0925, 0.3181]	0.0004	
Multiplex RT-PCR	0.1880 [0.0795, 0.2966]	0.0007	
PCR	0.1551 [0.0546, 0.2555]	0.0025	
qPCR	0.2366 [-0.0692, 0.5423]	0.1294	
RT-PCR	0.1230 [0.0344, 0.2116]	0.0065	
RT-qPCR	0.1441 [-0.0260, 0.3141]	0.0968	
**Sample type**			9.08%
Nasopharyngeal secretions	Reference		
Mixed specimens*	-0.0493 [-0.1583, 0.0597]	0.3753	
Nasal secretions	0.0261 [-0.0948, 0.1470]	0.6723	
Nasopharyngeal secretions or BLF	-0.1574 [-0.2423, -0.0725]	0.0003	
Oropharyngeal secretions	-0.1516 [-0.2499, -0.0534]	0.0025	
Sputum	-0.0642 [-0.2956, 0.1673]	0.587	
Throat secretions	-0.0924 [-0.2315, 0.0467]	0.1931	
**Design**			3.94%

Cross-sectional	Reference		
Longitudinal	0.0962 [0.0314, 0.1609]	0.0036	
**Timing of data collection**			0.30%
Prospective	Reference		
Ambispective	-0.0419 [-0.1936, 0.1099]	0.5888	
Retrospective	-0.0197 [-0.0801, 0.0407]	0.522	
**Risk of bias**			1.58%
Low risk	Reference		
Moderate risk	-0.0554 [-0.1157, 0.0049]	0.0716	

¥Immune assays: Antigen testing; or Direct immunofluorescence. ¥¥Molecular assays: Multiplex PCR; or Multiplex RT-PCR; or PCR; or qPCR; or RT-PCR; or RT-qPCR. ¥¥¥Mixed assays: Antigen testing/RT-PCR; or Direct immunofluorescence/RT-qPCR/antigen testing; or Direct immunofluorescence/RT-PCR; or Indirect immunofluorescence/PCR. $The indicated diagnostic technique is according to what is referred to by the authors. *Mixed specimens: Nasopharyngeal secretions and nasal secretions; or Nasal and throat secretions. Abbreviations: WHO, World Health Organization; BLF, Bronchoalveolar lavage fluid.

Figure S3. Bubble plot illustrating the association between average age in months and prevalence of respiratory syncytial virus.

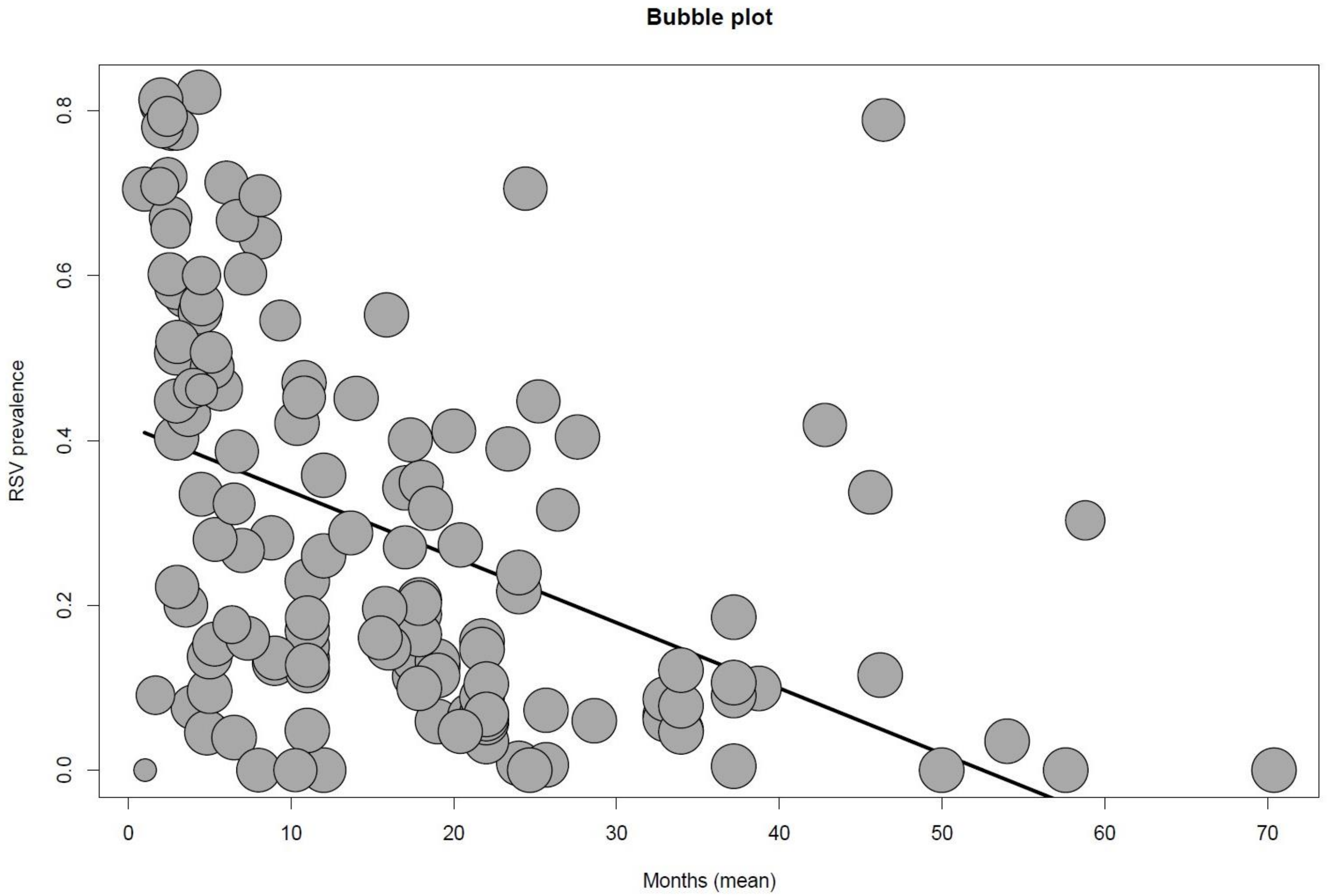

Abbreviations: RSV, respiratory syncytial virus.

Table S10. **The AMSTAR 2 (A Measurement Tool to Assess Systematic Reviews) instrument.**

AMSTAR 2

1. Did the research questions and inclusion criteria for the review include the components of PICO?

For Yes:<br>☒ Population<br>☒ Intervention<br>☒ Comparator group<br>☒ Outcome	Optional (recommended)<br>☒ Timeframe for follow-up	☒ Yes<br>☐ No

2. Did the report of the review contain an explicit statement that the review methods were established prior to the conduct of the review and did the report justify any significant deviations from the protocol?

For Partial Yes:<br>The authors state that they had a written protocol or guide that included ALL the following:<br>☒ review question(s)<br>☒ a search strategy<br>☒ inclusion/exclusion criteria<br>☒ a risk of bias assessment	For Yes:<br>As for partial yes, plus the protocol should be registered and should also have specified:<br>☒ a meta-analysis/synthesis plan, if appropriate, *and*<br>☒ a plan for investigating causes of heterogeneity<br>☒ justification for any deviations from the protocol	☒ Yes<br>☐ Partial Yes<br>☐ No

3. Did the review authors explain their selection of the study designs for inclusion in the review?

For Yes, the review should satisfy ONE of the following:<br>☐ *Explanation for* including only RCTs<br>☒ OR *Explanation for* including only NRSI<br>☐ OR *Explanation for* including both RCTs and NRSI	☒ Yes<br>☐ No

4. Did the review authors use a comprehensive literature search strategy?

For Partial Yes (all the following):<br>☒ searched at least 2 databases (relevant to research question)<br>☒ provided key word and/or search strategy<br>☒ justified publication restrictions (eg, language)	For Yes, should also have (all the following):<br>☐ searched the reference lists/bibliographies of included studies<br>☐ searched trial/study registries<br>☐ included/consulted content experts in the field<br>☐ where relevant, searched for grey literature<br>☐ conducted search within 24 months of completion of the review	☐ Yes<br>☒ Partial Yes<br>☐ No

5. Did the review authors perform study selection in duplicate?

For Yes, either ONE of the following:<br>☒ at least two reviewers independently agreed on selection of eligible studies and achieved consensus on which studies to include<br>☐ OR two reviewers selected a sample of eligible studies and achieved good agreement (at least 80 per cent), with the remainder selected by one reviewer	☒ Yes<br>☐ No

6. Did the review authors perform data extraction in duplicate?

For Yes, either ONE of the following:<br>☒ at least two reviewers achieved consensus on which data to extract	☒ Yes

from included studies<br>☐ OR two reviewers extracted data from a sample of eligible studies and achieved good agreement (at least 80 per cent), with the remainder extracted by one reviewer		☐ No

7. Did the review authors provide a list of excluded studies and justify the exclusions?

For Partial Yes:	For Yes, must also have:	
☐ provided a list of all potentially relevant studies that were read in full text form but excluded from the review	☒ Justified the exclusion from the review of each potentially relevant study	☒ Yes<br>☐ Partial Yes<br>☐ No

8. Did the review authors describe the included studies in adequate detail?

For Partial Yes (ALL the following):	For Yes, should also have ALL the following:	
☒ described populations<br>☒ described interventions<br>☒ described comparators<br>☒ described outcomes<br>☒ described research designs	☐ described population in detail<br>☐ described intervention and comparator in detail (including doses where relevant)<br>☐ described study's setting<br>☐ timeframe for follow-up	☐ Yes<br>☒ Partial Yes<br>☐ No

9. Did the review authors use a satisfactory technique for assessing the risk of bias (RoB) in individual studies that were included in the review?

**RCTs**<br>For Partial Yes, must have assessed RoB from	For Yes, must also have assessed RoB from:	
☐ unconcealed allocation, *and*<br>☐ lack of blinding of patients and assessors when assessing outcomes (unnecessary for objective outcomes such as all cause mortality)	☐ allocation sequence that was not truly random, *and*<br>☐ selection of the reported result from among multiple measurements or analyses of a specified outcome	☐ Yes<br>☐ Partial Yes<br>☐ No<br>☐ Includes only NRSI
**NRSI**<br>For Partial Yes, must have assessed RoB:	For Yes, must also have assessed RoB:	
☒ from confounding, *and*<br>☒ from selection bias	☐ methods used to ascertain exposures and outcomes, *and*<br>☐ selection of the reported result from among multiple measurements or analyses of a specified outcome	☐ Yes<br>☒ Partial Yes<br>☐ No<br>☐ Includes only RCTs

10. Did the review authors report on the sources of funding for the studies included in the review?

For Yes	
☐ Must have reported on the sources of funding for individual studies included in the review. Note: Reporting that the reviewers looked for this information but it was not reported by study authors also qualifies	☐ Yes<br>☒ No

11. If meta-analysis was performed did the review authors use appropriate methods for statistical combination of results?

**RCTs**<br>For Yes:	
☐ The authors justified combining the data in a meta-analysis<br>☐ AND they used an appropriate weighted technique to combine study results and adjusted for heterogeneity if present	☐ Yes<br>☐ No<br>☐ No meta-analysis

- [ ] AND investigated the causes of any heterogeneity — conducted

For NRSI
For Yes:

- [x] The authors justified combining the data in a meta-analysis
  - [x] AND they used an appropriate weighted technique to combine study results, adjusting for heterogeneity if present
  - [x] AND they statistically combined effect estimates from NRSI that were adjusted for confounding, rather than combining raw data, or justified combining raw data when adjusted effect estimates were not available
  - [ ] AND they reported separate summary estimates for RCTs and NRSI separately when both were included in the review

- [x] Yes
- [ ] No
- [ ] No meta-analysis conducted

12. If meta-analysis was performed, did the review authors assess the potential impact of RoB in individual studies on the results of the meta-analysis or other evidence synthesis?

For Yes:

- [ ] included only low risk of bias RCTs
- [x] OR, if the pooled estimate was based on RCTs and/or NRSI at variable RoB, the authors performed analyses to investigate possible impact of RoB on summary estimates of effect

- [x] Yes
- [ ] No
- [ ] No meta-analysis conducted

13. Did the review authors account for RoB in individual studies when interpreting/discussing the results of the review?

For Yes:

- [ ] included only low risk of bias RCTs
- [x] OR, if RCTs with moderate or high RoB, or NRSI were included the review provided a discussion of the likely impact of RoB on the results

- [x] Yes
- [ ] No

14. Did the review authors provide a satisfactory explanation for, and discussion of, any heterogeneity observed in the results of the review?

For Yes:

- [ ] There was no significant heterogeneity in the results
- [x] OR if heterogeneity was present the authors performed an investigation of sources of any heterogeneity in the results and discussed the impact of this on the results of the review

- [x] Yes
- [ ] No

15. If they performed quantitative synthesis did the review authors carry out an adequate investigation of publication bias (small study bias) and discuss its likely impact on the results of the review?

For Yes:

- [x] performed graphical or statistical tests for publication bias and discussed the likelihood and magnitude of impact of publication bias

- [x] Yes
- [ ] No
- [ ] No meta-analysis conducted

16. Did the review authors report any potential sources of conflict of interest, including any funding they received for conducting the review?

For Yes:

- [ ] The authors reported no competing interests OR
- [x] The authors described their funding sources and how they managed potential conflicts of interest

- [x] Yes
- [ ] No

Figure S4. Prevalence of intensive care unit (ICU) admissions in hospitalized children with lower respiratory tract infections related to respiratory syncytial virus (RSV).

Periods	ICU−admissions	RSV−cases	Prevalence (%)	95%−CI
**Pre−lockdown (2015/20)**				
Giamberardino et al. [30]	69	202	34.16	[27.65; 41.14]
Akkoc et al. [2]	7	21	33.33	[14.59; 56.97]
Haddadin et al. [35]	9	33	27.27	[13.30; 45.52]
Rha et al. [81]	182	1043	17.45	[15.19; 19.89]
Weldetsadik et al. [104]	4	26	15.38	[4.36; 34.87]
Gonapaladeniya et al. [31]	1	46	2.17	[0.06; 11.53]
Xu et al. [108]	0	25	0.00	[0.00; 13.72]
Stera et al. [92]	0	75	0.00	[0.00; 4.80]
**Random effects model**	**272**	**1471**	**8.97**	**[2.60; 26.71]**
Heterogeneity: $I^2 = 81\%$, $\tau^2 = 2.6824$, $p < 0.01$				
**Lockdown (2020/21)**				
Curatola et al. [23]	0	6	0.00	[0.00; 45.93]
Xu et al. [108]	0	25	0.00	[0.00; 13.72]
Pogonowska et al. [74]	1	61	1.64	[0.04; 8.80]
**Random effects model**	**1**	**92**	**1.09**	**[0.15; 7.31]**
Heterogeneity: $I^2 = 0\%$, $\tau^2 = 0$, $p = 1.00$				
**Random effects model**	**273**	**1563**	**4.99**	**[1.29; 17.47]**
Heterogeneity: $I^2 = 78\%$, $\tau^2 = 3.5096$, $p < 0.01$				
Test for subgroup differences: $\chi^2_1 = 3.31$, df = 1 ($p = 0.07$)				

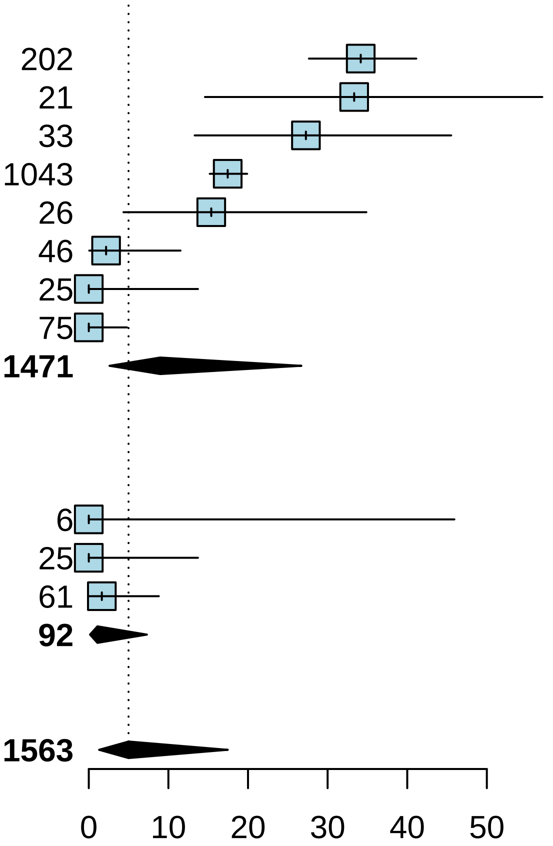

Figure S5. Mortality among hospitalized children with lower respiratory tract infections related to respiratory syncytial virus (RSV).

Periods	Deaths	RSV−cases	Prevalence (%)	95%−CI
**Pre−lockdown (2015/20)**				
Sobrinho et al. [90]	10	103	9.71	[4.75; 17.13]
Singh et al. [89]	1	20	5.00	[0.13; 24.87]
Weldetsadik et al. [104]	1	26	3.85	[0.10; 19.64]
Akkoc et al. [2]	0	21	0.00	[0.00; 16.11]
Lagare et al. [51]	3	149	2.01	[0.42; 5.77]
Xu et al. [108]	0	25	0.00	[0.00; 13.72]
Gonapaladeniya et al. [31]	0	46	0.00	[0.00; 7.71]
Jullien et al. [42]	0	52	0.00	[0.00; 6.85]
Pale et al. [72]	1	113	0.88	[0.02; 4.83]
Stera et al. [92]	0	72	0.00	[0.00; 4.99]
Stera et al. [92]	0	75	0.00	[0.00; 4.80]
Chandy et al. [19]	0	92	0.00	[0.00; 3.93]
Stera et al. [92]	0	94	0.00	[0.00; 3.85]
Buendía et al. [14]	0	193	0.00	[0.00; 1.89]
Giamberardino et al. [30]	0	202	0.00	[0.00; 1.81]
Brisca et al. [12]	0	217	0.00	[0.00; 1.69]
Rha et al. [81]	0	1043	0.00	[0.00; 0.35]
**Random effects model**	**16**	**2543**	**0.13**	**[0.01; 1.15]**
Heterogeneity: $I^2 = 0\%$, $\tau^2 = 6.0680$, $p = 0.86$				
**Lockdown (2020/21)**				
Brisca et al. [12]	0	3	0.00	[0.00; 70.76]
Xu et al. [108]	1	25	4.00	[0.10; 20.35]
**Random effects model**	**1**	**28**	**3.57**	**[0.50; 21.42]**
Heterogeneity: $I^2 = 0\%$, $\tau^2 = 0$, $p = 1.00$				
**Post−lockdown (2021/22)**				
Ng et al. [68]	0	30	0.00	[0.00; 11.57]
Faraguna et al. [27]	0	39	0.00	[0.00; 9.03]
Camporesi et al. [15]	0	105	0.00	[0.00; 3.45]
Camporesi et al. [16]	0	130	0.00	[0.00; 2.80]
Brisca et al. [12]	0	185	0.00	[0.00; 1.97]
**Random effects model**	**0**	**489**	**0.00**	**[0.00; 100.00]**
Heterogeneity: $I^2 = 0\%$, $\tau^2 = 0$, $p = 1.00$				
**Random effects model**	**17**	**3060**	**0.10**	**[0.01; 0.80]**
Heterogeneity: $I^2 = 0\%$, $\tau^2 = 6.0000$, $p = 0.99$				
Test for subgroup differences: $\chi^2_2 = 4.92$, df = 2 ($p = 0.09$)				

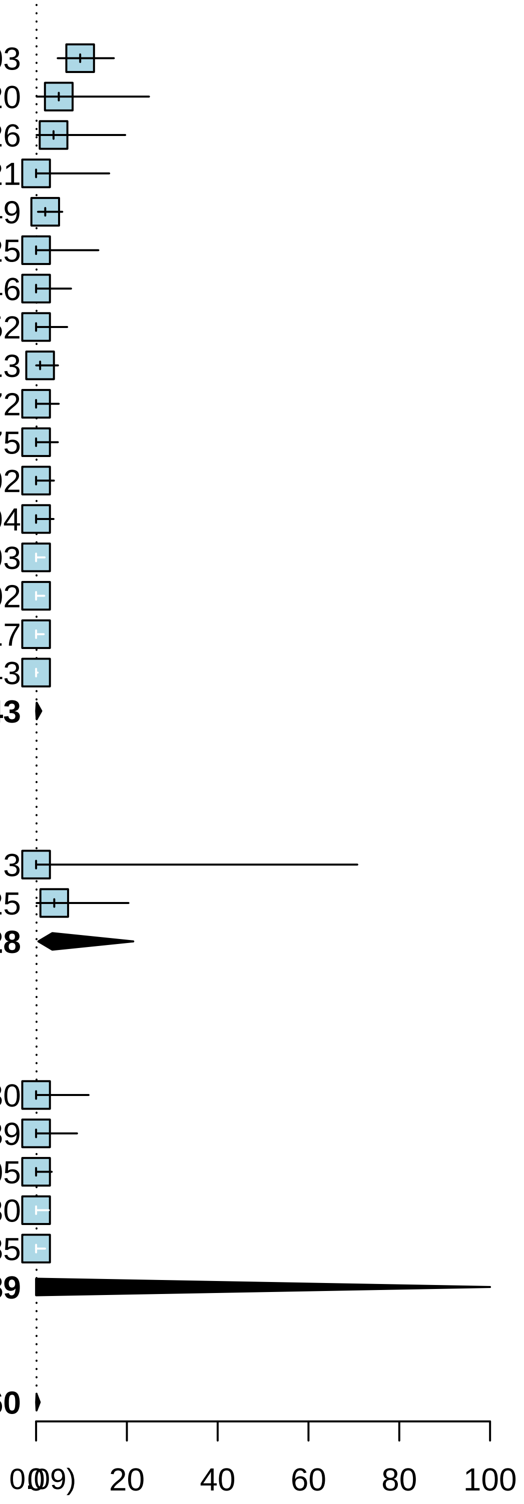

References:

1. Agca H, Akalin H, Saglik I, Hacimustafaoglu M, Celebi S, Ener B. Changing epidemiology of influenza and other respiratory viruses in the first year of COVID-19 pandemic. *J Infect Public Health*. 2021 Sep;14(9):1186-1190. doi: 10.1016/j.jiph.2021.08.004. Epub 2021 Aug 8. PMID: 34399190.

2. Akkoc G, Dogan C, Bayraktar S, Sahin K, Elevli M. Evaluation of viral respiratory pathogens in children aged under five hospitalized with lower respiratory tract infections. *North Clin Istanb*. 2022 Apr 14;9(2):162-172. doi: 10.14744/nci.2021.69923. PMID: 35582505; PMCID: PMC9039642.

3. Al-Zayadneh E, Mohammad Abu Assab D, Adeeb Arabiat E, Al-Iede M, Ahmad Kayed H, Daher A. The burden of influenza and other respiratory viruses in hospitalized infants and children in a university hospital, Jordan. *Multidiscip Respir Med*. 2021 Nov 11;16(1):763. doi: 10.4081/mrm.2021.763. PMID: 34900252; PMCID: PMC8607186.

4. Assane D, Makhtar C, Abdoulaye D, Amary F, Djibril B, Amadou D, Niokhor DJB, Amadou D, Cheikh L, Ndongo D, Mbayame N, Lamine F, Bouh BCS. Viral and Bacterial Etiologies of Acute Respiratory Infections Among Children Under 5 Years in Senegal. *Microbiol Insights*. 2018 Feb 13;11:1178636118758651. doi: 10.1177/1178636118758651. PMID: 29467579; PMCID: PMC5815418.

5. Baldassarre ME, Loconsole D, Centrone F, Caselli D, Martire B, Quartulli L, Acquafredda A, D'Amato G, Maffei G, Latorre G, Riganti A, Di Noia M, Chironna M, Laforgia N. Hospitalization for bronchiolitis in children aged ≤ 1year, Southern Italy, year 2021: need for new preventive strategies? *Ital J Pediatr*. 2023 Jun 6;49(1):66. doi: 10.1186/s13052-023-01455-2. PMID: 37280662; PMCID: PMC10243233.

6. Barbati F, Moriondo M, Pisano L, Calistri E, Lodi L, Ricci S, Giovannini M, Canessa C, Indolfi G, Azzari C. Epidemiology of Respiratory Syncytial Virus-Related Hospitalization Over a 5-Year Period in Italy: Evaluation of Seasonality and Age Distribution Before Vaccine

Introduction. *Vaccines (Basel)*. 2020 Jan 4;8(1):15. doi: 10.3390/vaccines8010015. PMID: 31947976; PMCID: PMC7157234.

7. Bedir Demirdağ T, Atay Ünal N, Tapısız A, Kara N, Güdeloğlu E, Tezer H, et al. Distribution and clinical features of viral respiratory infections in children after face-to-face education in 2021-2022 winter period. J Pediatr Inf. 2022;16:e1-e5.

8. Ben-Shimol S, Ramilo O, Leber AL, van der Beek BA, Everhart K, Mertz S, Mejias A, Dagan R. A Hypothesis-Generating Prospective Longitudinal Study to Assess the Relative Contribution of Common Respiratory Viruses to Severe Lower Respiratory Infections in Young Children. *Pediatr Infect Dis J*. 2023 May 1;42(5):396-404. doi: 10.1097/INF.0000000000003865. Epub 2023 Mar 8. PMID: 36917029.

9. Berdah L, Romain AS, Rivière S, Schnuriger A, Perrier M, Carbajal R, Lorrot M, Guedj R, Corvol H. Retrospective observational study of the influence of the COVID-19 outbreak on infants' hospitalisation for acute bronchiolitis. *BMJ Open*. 2022 Oct 31;12(10):e059626. doi: 10.1136/bmjopen-2021-059626. PMID: 36316083; PMCID: PMC9627576.

10. Bermúdez Barrezueta L, Gutiérrez Zamorano M, López-Casillas P, Brezmes-Raposo M, Sanz Fernández I, Pino Vázquez MA. Influence of the COVID-19 pandemic on the epidemiology of acute bronchiolitis. *Enferm Infecc Microbiol Clin (Engl Ed)*. 2023 Jun-Jul;41(6):348-351. doi: 10.1016/j.eimce.2022.09.001. Epub 2022 Sep 20. PMID: 36175287; PMCID: PMC9485429.

11. Bhardwaj S, Choudhary ML, Jadhav S, Vipat V, Ghuge R, Salvi S, Kulkarni R, Kinikar A, Padbidri V, Bafna S, Bavdekare A, D'costa P, Gujar N, Potdar V. A retrospective analysis of respiratory virus transmission before and during the COVID-19 pandemic in Pune, the western region of India. *Front Public Health*. 2022 Sep 8;10:936634. doi: 10.3389/fpubh.2022.936634. PMID: 36159243; PMCID: PMC9494283.

12. Brisca G, Mariani M, Buratti S, Ferretti M, Pirlo D, Buffoni I, Mallamaci M, Salvati P, Tagliarini G, Piccotti E, Sacco O, Castagnola E, Moscatelli A. How has the SARS-CoV-2 pandemic changed the epidemiology and management of acute bronchiolitis? *Pediatr*

Pulmonol. 2023 Apr;58(4):1169-1177. doi: 10.1002/ppul.26315. Epub 2023 Jan 20. PMID: 36636959.

13. Buendia JA, Guerrero Patino D. Importance of respiratory syncytial virus as a predictor of hospital length of stay in bronchiolitis. *F1000Res*. 2021 Feb 15;10:110. doi: 10.12688/f1000research.40670.3. PMID: 35903216.

14. Buendía JA, Polack FP, Patiño DG. Clinical Manifestations and Outcomes of Respiratory Syncytial Virus Infection in Children Less Than Two Years in Colombia. *Indian Pediatr*. 2021 Nov 15;58(11):1091-1092. Epub 2020 Aug 19. PMID: 32893827.

15. Camporesi A, Morello R, Pierucci UM, Proli F, Lazzareschi I, Bersani G, Valentini P, Roland D, Buonsenso D. 2021/22 and 2022/23 Post-Pandemic Bronchiolitis Seasons in Two Major Italian Cities: A Prospective Study. *Children (Basel)*. 2023 Jun 20;10(6):1081. doi: 10.3390/children10061081. PMID: 37371312; PMCID: PMC10297298.

16. Camporesi A, Morello R, Ferro V, Pierantoni L, Rocca A, Lanari M, Trobia GL, Sciacca T, Bellinvia AG, De Ferrari A, Valentini P, Roland D, Buonsenso D. Epidemiology, Microbiology and Severity of Bronchiolitis in the First Post-Lockdown Cold Season in Three Different Geographical Areas in Italy: A Prospective, Observational Study. *Children (Basel)*. 2022 Apr 1;9(4):491. doi: 10.3390/children9040491. PMID: 35455535; PMCID: PMC9024462.

17. Cao R, Du Y, Tong J, Xia D, Song Q, Xia Z, Liu M, Du H, Han J, Gao C. Influence of COVID-19 pandemic on the virus spectrum in children with respiratory infection in Xuzhou, China: a long-term active surveillance study from 2015 to 2021. *BMC Infect Dis*. 2023 Jul 13;23(1):467. doi: 10.1186/s12879-023-08247-3. PMID: 37442963; PMCID: PMC10339539.

18. Carlone G, Graziano G, Trotta D, Cafagno C, Aricò MO, Campodipietro G, Marabini C, Lizzi M, Fornaro M, Caselli D, Valletta E, Aricò M. Bronchiolitis 2021-2022 epidemic: multicentric analysis of the characteristics and treatment approach in 214 children from different areas in Italy. *Eur J Pediatr*. 2023 Apr;182(4):1921-1927. doi: 10.1007/s00431-023-04853-0. Epub 2023 Feb 20. PMID: 36807514; PMCID: PMC9943040.

19. Chandy S, Manoharan A, Hameed A, Jones LK, Nachiyar GS, Ramya MS, et al. A study on pediatric respiratory tract infections in hospitalised children from Chennai. Clin Epidemiol Glob Health. 2022;15:101067.

20. Chu FL, Li C, Chen L, Dong B, Qiu Y, Liu Y. Respiratory viruses among pediatric inpatients with acute lower respiratory tract infections in Jinan, China, 2016-2019. *J Med Virol*. 2022 Sep;94(9):4319-4328. doi: 10.1002/jmv.27875. Epub 2022 May 28. PMID: 35593042.

21. Coma E, Vila J, Méndez-Boo L, Antón A, Mora N, Fina F, Fàbregas M, Medina M. Respiratory Syncytial Virus Infections in Young Children Presenting to Primary Care in Catalonia During the COVID-19 Pandemic. *J Pediatric Infect Dis Soc*. 2022 Feb 23;11(2):69-72. doi: 10.1093/jpids/piab121. PMID: 34922377.

22. Curatola A, Graglia B, Ferretti S, Covino M, Pansini V, Eftimiadi G, Chiaretti A, Gatto A. The acute bronchiolitis rebound in children after COVID-19 restrictions: a retrospective, observational analysis. *Acta Biomed.* 2023 Feb 13;94(1):e2023031. doi: 10.23750/abm.v94i1.13552. PMID: 36786263; PMCID: PMC9987502.

23. Curatola A, Lazzareschi I, Bersani G, Covino M, Gatto A, Chiaretti A. Impact of COVID-19 outbreak in acute bronchiolitis: Lesson from a tertiary Italian Emergency Department. *Pediatr Pulmonol*. 2021 Aug;56(8):2484-2488. doi: 10.1002/ppul.25442. Epub 2021 May 7. PMID: 33961732; PMCID: PMC8242382.

24. De Rose DU, Caoci S, Auriti C, Maddaloni C, Capolupo I, Salvatori G, Brusco C, Coltella L, Concato C, Russo C, Colagrossi L, Perno CF, Braguglia A, Villani A, Dotta A, Raponi M. Lessons from SARS-CoV-2 Pandemics: How Restrictive Measures Impacted the Trend of Respiratory Infections in Neonates and Infants up to Three Months of Age. *Pathogens*. 2022 Sep 23;11(10):1086. doi: 10.3390/pathogens11101086. PMID: 36297143; PMCID: PMC9611509.

25. Diesner-Treiber SC, Voitl P, Voitl JJM, Langer K, Kuzio U, Riepl A, Patel P, Mühl-Riegler A, Mühl B. Respiratory Infections in Children During a Covid-19 Pandemic Winter. *Front Pediatr.*

2021 Oct 18;9:740785. doi: 10.3389/fped.2021.740785. PMID: 34733808; PMCID: PMC8558488.

26. Esen AB, Erol M, Kafadar D, Gayret OB, Yigit O, Dilek TD, et al. Progression of Disease and Viral Agents in Infants Hospitalized for Lower Respiratory Tract Infections. J Pediatr Res. 2019 Dec;6(4):314-321.

27. Faraguna MC, Lepri I, Clavenna A, Bonati M, Vimercati C, Sala D, Cattoni A, Melzi ML, Biondi A. The bronchiolitis epidemic in 2021-2022 during the SARS-CoV-2 pandemic: experience of a third level centre in Northern Italy. *Ital J Pediatr*. 2023 Feb 21;49(1):26. doi: 10.1186/s13052-023-01425-8. PMID: 36803828; PMCID: PMC9942300.

28. Feng Z, Xu B, Zhong L, Chen J, Deng J, Luo Z, Cao L, Tang Y, Li C, Jin R, Deng L, Shang Y, Wu Y, Zhao H, Feng Q, Chen X, Xu L, Xie Z. A multicentre study on the incidence of respiratory viruses in children with community-acquired pneumonia requiring hospitalization in the setting of the zero-COVID policy in China. *Arch Virol*. 2023 Jan 13;168(2):64. doi: 10.1007/s00705-023-05698-6. PMID: 36639581; PMCID: PMC9839207.

29. Foley DA, Phuong LK, Peplinski J, Lim SM, Lee WH, Farhat A, Minney-Smith CA, Martin AC, Mace AO, Sikazwe CT, Le H, Levy A, Hoeppner T, Borland ML, Hazelton B, Moore HC, Blyth C, Yeoh DK, Bowen AC. Examining the interseasonal resurgence of respiratory syncytial virus in Western Australia. *Arch Dis Child*. 2022 Mar;107(3):e7. doi: 10.1136/archdischild-2021-322507. Epub 2021 Aug 25. PMID: 34433552.

30. I G Giamberardino H, O Pacheco AP, Pereira LA, Debur MDC, Genehold G, Raboni SM. Respiratory syncytial virus: host genetic susceptibility and factors associated with disease severity in a cohort of pediatric patients. *J Trop Pediatr*. 2022 Oct 6;68(6):fmac091. doi: 10.1093/tropej/fmac091. PMID: 36323460.

31. Gonapaladeniya M, Dissanayake T, Liyanage G. Burden of Respiratory Syncytial Virus Associated Severe Pneumonia in Hospitalized Children. *Int J Pediatr*. 2021 Jun 30;2021:8269400. doi: 10.1155/2021/8269400. PMID: 34306103; PMCID: PMC8266452.

32. Grochowska M, Ambrożej D, Wachnik A, Demkow U, Podsiadły E, Feleszko W. The Impact of the COVID-19 Pandemic Lockdown on Pediatric Infections-A Single-Center Retrospective Study. *Microorganisms*. 2022 Jan 14;10(1):178. doi: 10.3390/microorganisms10010178. PMID: 35056625; PMCID: PMC8778921.

33. Guerrero-Del-Cueto F, Ramos-Fernandez JM, Leiva-Gea I, Reina-Moreno E, Ortiz-Ortigosa A, Carazo-Gallego B, Cordon-Martinez AM, Moreno-Perez D, Nuñez-Cuadros E. Bronchiolitis before and after the SARS-CoV-2 pandemic: Twelve years of experience in a Spanish paediatric hospital. *Pediatr Pulmonol*. 2023 Apr;58(4):1201-1209. doi: 10.1002/ppul.26322. Epub 2023 Jan 24. PMID: 36653064.

34. Guitart C, Bobillo-Perez S, Alejandre C, Armero G, Launes C, Cambra FJ, Balaguer M, Jordan I; Hospital Network for R. S. V. surveillance in Catalonia. Bronchiolitis, epidemiological changes during the SARS-CoV-2 pandemic. BMC Infect Dis. 2022 Jan 24;22(1):84. doi: 10.1186/s12879-022-07041-x. PMID: 35073855; PMCID: PMC8785150.

35. Haddadin Z, Rankin DA, Lipworth L, Suh M, McHenry R, Blozinski A, George SS, Fernandez KN, Varjabedian R, Spieker AJ, Shepard DS, Halasa NB. Respiratory Virus Surveillance in Infants across Different Clinical Settings. J Pediatr. 2021 Jul;234:164-171.e2. doi: 10.1016/j.jpeds.2021.03.036. Epub 2021 Mar 25. PMID: 33774057.

36. Havdal LB, Bøås H, Bekkevold T, Kran AB, Rojahn AE, Størdal K, Debes S, Døllner H, Nordbø SA, Barstad B, Haarr E, Fernández LV, Nakstad B, Inchley C, Flem E. The burden of respiratory syncytial virus in children under 5 years of age in Norway. J Infect. 2022 Feb;84(2):205-215. doi: 10.1016/j.jinf.2021.12.008. Epub 2021 Dec 11. Erratum in: J Infect. 2023 Apr;86(4):420. PMID: 34906596.

37. Hu XW, Zhou Y, Yi S, Zhang WX, Wang XR, Du J, Lu QB. Epidemiological Characteristics of Respiratory Syncytial Virus Infection Among Hospitalized Children With Acute Respiratory Tract Infections From 2014 to 2022 in a Hospital in Hubei Province, China: Longitudinal

Surveillance Study. JMIR Public Health Surveill. 2023 Apr 27;9:e43941. doi: 10.2196/43941. PMID: 36975172; PMCID: PMC10176131.

38. Ippolito G, La Vecchia A, Umbrello G, Di Pietro G, Bono P, Scalia Catenacci S, Pinzani R, Tagliabue C, Bosis S, Agostoni C, Marchisio PG. Disappearance of Seasonal Respiratory Viruses in Children Under Two Years Old During COVID-19 Pandemic: A Monocentric Retrospective Study in Milan, Italy. Front Pediatr. 2021 Aug 5;9:721005. doi: 10.3389/fped.2021.721005. Erratum in: Front Pediatr. 2022 Aug 23;10:1006948. PMID: 34422733; PMCID: PMC8374241.

39. Jiang ML, Xu YP, Wu H, Zhu RN, Sun Y, Chen DM, Wang F, Zhou YT, Guo Q, Wu A, Qian Y, Zhou HY, Zhao LQ. Changes in endemic patterns of respiratory syncytial virus infection in pediatric patients under the pressure of nonpharmaceutical interventions for COVID-19 in Beijing, China. J Med Virol. 2023 Jan;95(1):e28411. doi: 10.1002/jmv.28411. PMID: 36524893; PMCID: PMC9878212.

40. Jiang W, Wu M, Zhou J, Wang Y, Hao C, Ji W, Zhang X, Gu W, Shao X. Etiologic spectrum and occurrence of coinfections in children hospitalized with community-acquired pneumonia. BMC Infect Dis. 2017 Dec 20;17(1):787. doi: 10.1186/s12879-017-2891-x. PMID: 29262797; PMCID: PMC5738861.

41. Jiang X, Wang T, Dai G, Sun H, Jiang W, Chen Z, Yan Y. Clinical characteristics and etiology of children with bronchiolitis before and during the COVID-19 pandemic in Suzhou, China. Front Pediatr. 2022 Nov 14;10:974769. doi: 10.3389/fped.2022.974769. PMID: 36452360; PMCID: PMC9703066.

42. Jullien S, Pradhan D, Tshering T, Sharma R, Dema K, Garcia-Garcia S, Ribó JL, Muñoz-Almagro C, Bassat Q. Pneumonia in children admitted to the national referral hospital in Bhutan: A prospective cohort study. Int J Infect Dis. 2020 Jun;95:74-83. doi: 10.1016/j.ijid.2020.04.017. Epub 2020 Apr 10. PMID: 32283281; PMCID: PMC7195124.

43. Kang M, Sarkar S, Angurana SK, Singh P, Rana M, Bora I, Chetanya R, Singh B, Muralidharan J, Ratho RK. Paradigm shift of respiratory viruses causing lower respiratory tract infection in children during COVID-19 pandemic in India. J Infect Dev Ctries. 2023 Jul 27;17(7):961-970. doi: 10.3855/jidc.17727. PMID: 37515805.

44. Kıymet E, Böncüoğlu E, Şahinkaya Ş, Cem E, Çelebi MY, Düzgöl M, Kara AA, Arıkan KÖ, Aydın T, İşgüder R, Yılmazer MM, Ayhan Y, Gülfidan G, Bayram A, Bayram N, Çelik T, Alp YT, Devrim İ. Distribution of spreading viruses during COVID-19 pandemic: Effect of mitigation strategies. Am J Infect Control. 2021 Sep;49(9):1142-1145. doi: 10.1016/j.ajic.2021.06.002. Epub 2021 Jun 9. PMID: 34116082; PMCID: PMC8187742.

45. Knudsen PK, Lind A, Klundby I, Dudman S. The incidence of infectious diseases and viruses other than SARS-CoV-2 amongst hospitalised children in Oslo, Norway during the Covid-19 pandemic 2020-2021. J Clin Virol Plus. 2022 Feb;2(1):100060. doi: 10.1016/j.jcvp.2021.100060. Epub 2021 Dec 22. PMID: 35262033; PMCID: PMC8692238.

46. Kockuzu E, Bayrakcı B, Kesici S, Cıtak A, Karapınar B, Emeksiz S, Anıl AB, Kendirli T, Yukselmis U, Sevketoglu E, Paksu Ş, Kutlu O, Agın H, Yıldızdas D, Keskin H, Kalkan G, Hasanoglu A, Yazıcı MU, Sık G, Kılınc A, Durak F, Perk O, Talip M, Yener N, Uzuner S. Comprehensive Analysis of Severe Viral Infections of Respiratory Tract admitted to PICUs during the Winter Season in Turkey. Indian J Crit Care Med. 2019 Jun;23(6):263-269. doi: 10.5005/jp-journals-10071-23177. PMID: 31435144; PMCID: PMC6698354.

47. Komoyo GF, Yambiyo BM, Manirakiza A, Gody JC, Muller CP, Hübschen JM, Nakoune E, Snoeck CJ. Epidemiology and genetic characterization of respiratory syncytial virus in children with acute respiratory infections: Findings from the influenza sentinel surveillance network in Central African Republic, 2015 to 2018. Health Sci Rep. 2021 May 11;4(2):e298. doi: 10.1002/hsr2.298. PMID: 34013070; PMCID: PMC8112815.

48. Korsun N, Angelova S, Trifonova I, Voleva S, Grigorova I, Tzotcheva I, Mileva S, Alexiev I, Perenovska P. Predominance of ON1 and BA9 genotypes of respiratory syncytial virus (RSV)

in Bulgaria, 2016-2018. J Med Virol. 2021 Jun;93(6):3401-3411. doi: 10.1002/jmv.26415. Epub 2020 Oct 30. PMID: 32779756.

49. Kume Y, Hashimoto K, Chishiki M, Norito S, Suwa R, Ono T, Mochizuki I, Mashiyama F, Ishibashi N, Suzuki S, Sakuma H, Takahashi H, Takeda M, Shirato K, Hosoya M. Changes in virus detection in hospitalized children before and after the severe acute respiratory syndrome coronavirus 2 pandemic. Influenza Other Respir Viruses. 2022 Sep;16(5):837-841. doi: 10.1111/irv.12995. Epub 2022 Apr 29. PMID: 35488324; PMCID: PMC9343337.

50. Kurskaya OG, Prokopyeva EA, Sobolev IA, Solomatina MV, Saroyan TA, Dubovitskiy NA, Derko AA, Nokhova AR, Anoshina AV, Leonova NV, Simkina OA, Komissarova TV, Shestopalov AM, Sharshov KA. Changes in the Etiology of Acute Respiratory Infections among Children in Novosibirsk, Russia, between 2019 and 2022: The Impact of the SARS-CoV-2 Virus. Viruses. 2023 Apr 9;15(4):934. doi: 10.3390/v15040934. PMID: 37112913; PMCID: PMC10141072.

51. Lagare A, Ousmane S, Dano ID, Issaka B, Issa I, Mainassara HB, Testa J, Tempia S, Mamadou S. Molecular detection of respiratory pathogens among children aged younger than 5 years hospitalized with febrile acute respiratory infections: A prospective hospital-based observational study in Niamey, Niger. Health Sci Rep. 2019 Oct 11;2(11):e137. doi: 10.1002/hsr2.137. PMID: 31768420; PMCID: PMC6869554.

52. Lamrani Hanchi A, Guennouni M, Ben Houmich T, Echchakery M, Draiss G, Rada N, Younous S, Bouskraoui M, Soraa N. Changes in the Epidemiology of Respiratory Pathogens in Children during the COVID-19 Pandemic. Pathogens. 2022 Dec 15;11(12):1542. doi: 10.3390/pathogens11121542. PMID: 36558876; PMCID: PMC9781834.

53. Li Y, Zhai Y, Lin Y, Lu C, He Z, Wu S, Yang C, Zhou R, Liu W, Chen D. Epidemiology of respiratory syncytial virus in hospitalized children with community-acquired pneumonia in Guangzhou: a 10-year study. J Thorac Dis. 2023 Mar 31;15(3):967-976. doi: 10.21037/jtd-22-331. Epub 2023 Feb 16. PMID: 37065548; PMCID: PMC10089879.

54. Li M, Li C, Jian X, Han D, Zhao J, Jiang L. Viral etiology of acute respiratory tract infection among children under 5 years of age in Kunming City, China: a matched case-case-control study. J Appl Microbiol. 2023 Jul 4;134(7):lxad138. doi: 10.1093/jambio/lxad138. PMID: 37418247.

55. Li W, Wang Y, Yu B, Tan Q, Zhou J, Hu J, Wu Y, Wang B, Li H. Disease severity of respiratory syncytial virus (RSV) infection correlate to a novel set of five amino acid substitutions in the RSV attachment glycoprotein (G) in China. Virus Res. 2020 May;281:197937. doi: 10.1016/j.virusres.2020.197937. Epub 2020 Mar 16. PMID: 32194139.

56. Li F, Zhang Y, Shi P, Cao L, Su L, Zhang Y, Peng K, Lu R, Tan W, Shen J. Epidemiology of Viruses Causing Pediatric Community Acquired Pneumonia in Shanghai During 2010-2020: What Happened Before and After the COVID-19 Outbreak? Infect Dis Ther. 2022 Feb;11(1):165-174. doi: 10.1007/s40121-021-00548-x. Epub 2021 Oct 25. PMID: 34694579; PMCID: PMC8542501.

57. Liu P, Xu M, He L, Su L, Wang A, Fu P, Lu L, Wang C, Xu J. Epidemiology of Respiratory Pathogens in Children with Lower Respiratory Tract Infections in Shanghai, China, from 2013 to 2015. Jpn J Infect Dis. 2018 Jan 23;71(1):39-44. doi: 10.7883/yoken.JJID.2017.323. Epub 2017 Dec 26. PMID: 29279451.

58. Liu P, Xu M, Cao L, Su L, Lu L, Dong N, Jia R, Zhu X, Xu J. Impact of COVID-19 pandemic on the prevalence of respiratory viruses in children with lower respiratory tract infections in China. Virol J. 2021 Aug 3;18(1):159. doi: 10.1186/s12985-021-01627-8. PMID: 34344406; PMCID: PMC8329611.

59. Lucion MF, Del Valle Juárez M, Pejito MN, Orqueda AS, Romero Bollón L, Mistchenko AS, Gentile Á. Impact of COVID-19 on the circulation of respiratory viruses in a children's hospital: an expected absence. Arch Argent Pediatr. 2022 Apr;120(2):99-105. English, Spanish. doi: 10.5546/aap.2022.eng.99. Epub 2022 Jan 12. PMID: 35338813.

60. Ma JE, Ma QF, Wang W, Zeng Y, Cui XH, Wang GL, Liu SY, Wang Z, Xiao X, Xia ZN, Zhu HC, Zhu CL. Analysis of Common Respiratory Infected Pathogens in 3100 Children after the Coronavirus Disease 2019 Pandemic. Curr Med Sci. 2022 Oct;42(5):1094-1098. doi: 10.1007/s11596-022-2635-z. Epub 2022 Oct 3. PMID: 36184728; PMCID: PMC9527073.

61. Mackenzie GA, Vilane A, Salaudeen R, Hogerwerf L, van den Brink S, Wijsman LA, Overduin P, Janssens TKS, de Silva TI, van der Sande MAB, Kampmann B, Meijer A. Respiratory syncytial, parainfluenza and influenza virus infection in young children with acute lower respiratory infection in rural Gambia. Sci Rep. 2019 Nov 29;9(1):17965. doi: 10.1038/s41598-019-54059-4. PMID: 31784567; PMCID: PMC6884537.

62. Maglione M, Pascarella A, Botti C, Ricci G, Morelli F, Camelia F, Micillo A, Calì C, Savoia F, Tipo V, Giannattasio A. Changing Epidemiology of Acute Viral Respiratory Infections in Hospitalized Children: The Post-Lockdown Effect. Children (Basel). 2022 Aug 17;9(8):1242. doi: 10.3390/children9081242. PMID: 36010132; PMCID: PMC9406795.

63. Maruo Y, Ishikawa S, Oura K, Shiraishi H, Sato N, Suganuma T, Mikawa M, Sato T. The impact of the coronavirus disease 2019 pandemic on pediatric hospitalization in Kitami, Japan. Pediatr Int. 2022 Jan;64(1):e14937. doi: 10.1111/ped.14937. PMID: 34324763; PMCID: PMC8447412.

64. Mira-Iglesias A, Demont C, López-Labrador FX, Mengual-Chuliá B, García-Rubio J, Carballido-Fernández M, Tortajada-Girbés M, Mollar-Maseres J, Schwarz-Chavarri G, Puig-Barberà J, Díez-Domingo J; Valencia Hospital Network for the Study of Influenza and other Respiratory Viruses. Role of age and birth month in infants hospitalized with RSV-confirmed disease in the Valencia Region, Spain. Influenza Other Respir Viruses. 2022 Mar;16(2):328-339. doi: 10.1111/irv.12937. Epub 2021 Nov 24. PMID: 34821055; PMCID: PMC8818825.

65. Moreira ALE, da Silva PAN, Assunção LDP, Santos MO, Ito CRM, de Araújo KM, Cunha MO, Rabelo VDC, de Souza PP, Maia SBS, Peixoto FAO, Wastowski IJ, Carneiro LC, Avelino MAG. Profile analysis of emerging respiratory virus in children. Eur J Clin Microbiol Infect

Dis. 2023 Jul;42(7):873-882. doi: 10.1007/s10096-023-04615-8. Epub 2023 May 9. PMID: 37160574; PMCID: PMC10169160.

66. Nenna R, Matera L, Pierangeli A, Oliveto G, Viscido A, Petrarca L, La Regina DP, Mancino E, Di Mattia G, Villani A, Midulla F. First COVID-19 lockdown resulted in most respiratory viruses disappearing among hospitalised children, with the exception of rhinoviruses. Acta Paediatr. 2022 Jul;111(7):1399-1403. doi: 10.1111/apa.16326. Epub 2022 Mar 16. PMID: 35266576; PMCID: PMC9111239.

67. Nenna R, Matera L, Licari A, Manti S, Di Bella G, Pierangeli A, Palamara AT, Nosetti L, Leonardi S, Marseglia GL, Midulla F; ICHRIS Group. An Italian Multicenter Study on the Epidemiology of Respiratory Syncytial Virus During SARS-CoV-2 Pandemic in Hospitalized Children. Front Pediatr. 2022 Jul 14;10:930281. doi: 10.3389/fped.2022.930281. PMID: 35911833; PMCID: PMC9329524.

68. Ng DC, Tan KK, Ting GSS, Ling C, Fadzilah NFB, Tan SF, Subramaniam T, Zailanalhuddin NEB, Lim HY, Baharuddin SB, Lee YL, Mohamad Nor A, Khoo EJ. Comparison of Severe Viral Pneumonia Caused by SARS-CoV-2 and Other Respiratory Viruses Among Malaysian Children During the COVID-19 Pandemic. Front Pediatr. 2022 Apr 25;10:865099. doi: 10.3389/fped.2022.865099. PMID: 35547549; PMCID: PMC9082799.

69. Núñez-Samudio V, Landires I. Epidemiology of viral respiratory infections in a pediatric reference hospital in Central Panama. BMC Infect Dis. 2021 Jan 9;21(1):43. doi: 10.1186/s12879-020-05720-1. PMID: 33422002; PMCID: PMC7796567.

70. Nunziata F, Salomone S, Catzola A, Poeta M, Pagano F, Punzi L, Lo Vecchio A, Guarino A, Bruzzese E. Clinical Presentation and Severity of SARS-CoV-2 Infection Compared to Respiratory Syncytial Virus and Other Viral Respiratory Infections in Children Less than Two Years of Age. Viruses. 2023 Mar 9;15(3):717. doi: 10.3390/v15030717. PMID: 36992426; PMCID: PMC10055850.

71. Oumei H, Xuefeng W, Jianping L, Kunling S, Rong M, Zhenze C, Li D, Huimin Y, Lining W, Zhaolan L, Xinmin L, Hua X, Zhiyan J, Yanning L, Yan H, Baoqing Z, Xiaochun F, Chunhui H, Yonghong J, Xue Z, Wei W, Zi W. Etiology of community-acquired pneumonia in 1500 hospitalized children. J Med Virol. 2018 Mar;90(3):421-428. doi: 10.1002/jmv.24963. Epub 2017 Nov 9. PMID: 28975629; PMCID: PMC7166354.

72. Pale M, Nacoto A, Tivane A, Nguenha N, Machalele L, Gundane F, Muteto D, Chilundo J, Mavale S, Semá-Baltazar C, Pires G, Augusto O, Mussá T, Gudo E. Respiratory syncytial and influenza viruses in children under 2 years old with severe acute respiratory infection (SARI) in Maputo, 2015. PLoS One. 2017 Nov 30;12(11):e0186735. doi: 10.1371/journal.pone.0186735. PMID: 29190684; PMCID: PMC5708764.

73. Pierangeli A, Piralla A, Uceda Renteria S, Giacomel G, Lunghi G, Pagani E, Giacobazzi E, Vian E, Biscaro V, Piccirilli G, Lazzarotto T, Menzo S, Ferreri ML, Novazzi F, Petrarca L, Licari A, Ferrari G, Oliveto G, Antonelli G, Binda S, Galli C, Pellegrinelli L, Pariani E, Baldanti F. Multicenter epidemiological investigation and genetic characterization of respiratory syncytial virus and metapneumovirus infections in the pre-pandemic 2018-2019 season in northern and central Italy. Clin Exp Med. 2023 Oct;23(6):2725-2737. doi: 10.1007/s10238-022-00973-3. Epub 2022 Dec 15. PMID: 36522554; PMCID: PMC9754777.

74. Pogonowska M, Guzek A, Goscinska A, Rustecka A, Kalicki B. Compensatory epidemic of RSV infections during the COVID-19 pandemic. An analysis of infections in children hospitalised in the Department of Paediatrics, Paediatric Nephrology and Allergology of the Military Medical Institute in Warsaw in 2020-2021. Pediatr Med Rodz. 2022;18(1):52-57.

75. Prasad N, Newbern EC, Trenholme AA, Wood T, Thompson MG, Aminisani N, Huang QS, Grant CC. Respiratory syncytial virus hospitalisations among young children: a data linkage study. Epidemiol Infect. 2019 Jan;147:e246. doi: 10.1017/S0950268819001377. Erratum in: Epidemiol Infect. 2020 Aug 28;148:e179. PMID: 31364578; PMCID: PMC6805750.

76. Prasad N, Trenholme AA, Huang QS, Duque J, Grant CC, Newbern EC. Respiratory Virus-related Emergency Department Visits and Hospitalizations Among Infants in New Zealand. Pediatr Infect Dis J. 2020 Aug;39(8):e176-e182. doi: 10.1097/INF.0000000000002681. PMID: 32675757.

77. Presti S, Manti S, Gambilonghi F, Parisi GF, Papale M, Leonardi S. Comparative Analysis of Pediatric Hospitalizations during Two Consecutive Influenza and Respiratory Virus Seasons Post-Pandemic. Viruses. 2023 Aug 28;15(9):1825. doi: 10.3390/v15091825. PMID: 37766232; PMCID: PMC10535437.

78. Pruccoli G, Castagno E, Raffaldi I, Denina M, Barisone E, Baroero L, Timeus F, Rabbone I, Monzani A, Terragni GM, Lovera C, Brach Del Prever A, Manzoni P, Barbaglia M, Roasio L, De Franco S, Calitri C, Lupica M, Felici E, Marciano C, Santovito S, Militerno G, Abrigo E, Curtoni A, Quarello P, Bondone C, Garazzino S. The Importance of RSV Epidemiological Surveillance: A Multicenter Observational Study of RSV Infection during the COVID-19 Pandemic. Viruses. 2023 Jan 19;15(2):280. doi: 10.3390/v15020280. PMID: 36851494; PMCID: PMC9963567.

79. Qiu W, Zheng C, Huang S, Zhang Y, Chen Z. Epidemiological Trend of RSV Infection Before and During COVID-19 Pandemic: A Three-Year Consecutive Study in China. Infect Drug Resist. 2022 Nov 28;15:6829-6837. doi: 10.2147/IDR.S388231. PMID: 36465809; PMCID: PMC9717604.

80. Ramezannia Z, Sadeghi J, Abdoli Oskouie S, Ahangarzadeh Rezaee M, Bannazadeh Baghi H, Azadi A, Ahangar Oskouee M. Evaluation of Human Respiratory Syncytial Virus and Human Parainfluenza Virus Type 3 among Hospitalized Children in Northwest of Iran. Can J Infect Dis Med Microbiol. 2021 Sep 22;2021:2270307. doi: 10.1155/2021/2270307. PMID: 34603564; PMCID: PMC8481064.

81. Rha B, Curns AT, Lively JY, Campbell AP, Englund JA, Boom JA, Azimi PH, Weinberg GA, Staat MA, Selvarangan R, Halasa NB, McNeal MM, Klein EJ, Harrison CJ, Williams JV, Szilagyi PG, Singer MN, Sahni LC, Figueroa-Downing D, McDaniel D, Prill MM, Whitaker BL, Stewart LS, Schuster JE, Pahud BA, Weddle G, Avadhanula V, Munoz FM, Piedra PA, Payne DC, Langley G, Gerber SI. Respiratory Syncytial Virus-Associated Hospitalizations Among Young Children: 2015-2016. Pediatrics. 2020 Jul;146(1):e20193611. doi: 10.1542/peds.2019-3611. Epub 2020 Jun 16. PMID: 32546583.

82. Roland D, Williams T, Lyttle MD, Marlow R, Hardelid P, Sinha I, Swann O, Maxwell-Hodkinson A, Cunningham S. Features of the transposed seasonality of the 2021 RSV epidemic in the UK and Ireland: analysis of the first 10 000 patients. Arch Dis Child. 2022 Nov;107(11):1062-1063. doi: 10.1136/archdischild-2022-324241. Epub 2022 Sep 2. PMID: 36261149.

83. Sachs N, Goldberg L, Levinsky Y, Dizitzer Y, Vardi Y, Krause I, Scheuerman O, Livni G, Bilavsky E, Bilavsky-Yarden H. The Effect of the COVID-19 Pandemic on Pediatric Respiratory Hospitalizations. Isr Med Assoc J. 2023 Mar;25(3):171-176. PMID: 36946659.

84. Sarkar S, Ratho RK, Singh M, Singh MP, Singh A, Sharma M. Comparative Analysis of Epidemiology, Clinical Features, and Cytokine Response of Respiratory Syncytial and Human Metapneumovirus Infected Children with Acute Lower Respiratory Infections. Jpn J Infect Dis. 2022 Jan 24;75(1):56-62. doi: 10.7883/yoken.JJID.2021.151. Epub 2021 Jun 30. PMID: 34193665.

85. Savaş Şen Z, Vezir E, Ertürk P. The relationship between viruses and clinical findings in hospitalized children diagnosed with acute lower respiratory tract infection. J Pediatr Inf. 2022;16(1):e13-e19.

86. Shen Z, Zhang Y, Li H, Du L. Rapid typing diagnosis and clinical analysis of subtypes A and B of human respiratory syncytial virus in children. Virol J. 2022 Jan 21;19(1):15. doi: 10.1186/s12985-022-01744-y. PMID: 35062975; PMCID: PMC8781464.

87. Shen DP, Vermeulen F, Debeer A, Lagrou K, Smits A. Impact of COVID-19 on viral respiratory infection epidemiology in young children: A single-center analysis. Front Public Health. 2022 Sep 20;10:931242. doi: 10.3389/fpubh.2022.931242. PMID: 36203684; PMCID: PMC9530989.

88. Shi T, Huang L. Prevalence of respiratory pathogens and risk of developing pneumonia under non-pharmaceutical interventions in Suzhou, China. Epidemiol Infect. 2023 May 2;151:e82. doi: 10.1017/S0950268823000626. PMID: 37127406; PMCID: PMC10226187.

89. Singh C, Angurana SK, Bora I, Jain N, Kaur K, Sarkar S. Clinico demographic profiling of the Respiratory syncytial virus (RSV) infected children admitted in tertiary care hospital in North India. J Family Med Prim Care. 2021 May;10(5):1975-1980. doi: 10.4103/jfmpc.jfmpc_2406_20. Epub 2021 May 31. PMID: 34195134; PMCID: PMC8208215.

90. Sobrinho FLDV, Aragon DC, Carlotti APCP. Epidemiology and factors associated with the severity of viral acute lower respiratory infection in children hospitalized in Manaus, Amazonas, in 2017-2018: An observational study. Medicine (Baltimore). 2021 May 7;100(18):e25799. doi: 10.1097/MD.0000000000025799. PMID: 33950979; PMCID: PMC8104226.

91. Song X, Delaney M, Shah RK, Campos JM, Wessel DL, DeBiasi RL. Common seasonal respiratory viral infections in children before and during the coronavirus disease 2019 (COVID-19) pandemic. Infect Control Hosp Epidemiol. 2022 Oct;43(10):1454-1458. doi: 10.1017/ice.2021.430. Epub 2021 Oct 5. PMID: 34607617.

92. Stera G, Pierantoni L, Masetti R, Leardini D, Biagi C, Buonsenso D, Pession A, Lanari M. Impact of SARS-CoV-2 Pandemic on Bronchiolitis Hospitalizations: The Experience of an Italian Tertiary Center. Children (Basel). 2021 Jun 28;8(7):556. doi: 10.3390/children8070556. PMID: 34203542; PMCID: PMC8304517.

93. Tang X, Dai G, Jiang X, Wang T, Sun H, Chen Z, Huang L, Wang M, Zhu C, Yan Y, Jiang W. Clinical Characteristics of Pediatric Respiratory Tract Infection and Respiratory Pathogen Isolation During the Coronavirus Disease 2019 Pandemic. Front Pediatr. 2022 Jan 5;9:759213. doi: 10.3389/fped.2021.759213. PMID: 35071128; PMCID: PMC8767000.

94. Thongpan I, Vichaiwattana P, Vongpunsawad S, Poovorawan Y. Upsurge of human rhinovirus infection followed by a delayed seasonal respiratory syncytial virus infection in Thai children during the coronavirus pandemic. Influenza Other Respir Viruses. 2021 Nov;15(6):711-720. doi: 10.1111/irv.12893. Epub 2021 Aug 4. PMID: 34350701; PMCID: PMC8542963.

95. Thwaites RS, Coates M, Ito K, Ghazaly M, Feather C, Abdulla F, Tunstall T, Jain P, Cass L, Rapeport G, Hansel TT, Nadel S, Openshaw P. Reduced Nasal Viral Load and IFN Responses in Infants with Respiratory Syncytial Virus Bronchiolitis and Respiratory Failure. Am J Respir Crit Care Med. 2018 Oct 15;198(8):1074-1084. doi: 10.1164/rccm.201712-2567OC. PMID: 29688024; PMCID: PMC6221568.

96. Trenholme A, Webb R, Lawrence S, Arrol S, Taylor S, Ameratunga S, Byrnes CA. COVID-19 and Infant Hospitalizations for Seasonal Respiratory Virus Infections, New Zealand, 2020. Emerg Infect Dis. 2021 Feb;27(2):641-643. doi: 10.3201/eid2702.204041. Epub 2020 Dec 12. PMID: 33263515; PMCID: PMC7853573.

97. Tsergouli K, Pappa S, Haidopoulou K, Gogou M, Giannopoulos A, Papa A. Respiratory Syncytial Virus in Greece, 2016-2018. Intervirology. 2019;62(5-6):210-215. doi: 10.1159/000506049. Epub 2020 Apr 29. PMID: 32348998.

98. Valley-Omar Z, Tempia S, Hellferscee O, Walaza S, Variava E, Dawood H, Kahn K, McMorrow M, Pretorius M, Mtshali S, Mamorobela E, Wolter N, Venter M, von Gottberg A, Cohen C, Treurnicht FK. Human respiratory syncytial virus diversity and epidemiology among patients hospitalized with severe respiratory illness in South Africa, 2012-2015. Influenza Other Respir Viruses. 2022 Mar;16(2):222-235. doi: 10.1111/irv.12905. Epub 2021 Sep 16. PMID: 34528769; PMCID: PMC8818822.

99. Varela FH, Sartor ITS, Polese-Bonatto M, Azevedo TR, Kern LB, Fazolo T, de David CN, Zavaglia GO, Fernandes IR, Krauser JRM, Stein RT, Scotta MC. Rhinovirus as the main co-circulating virus during the COVID-19 pandemic in children. J Pediatr (Rio J). 2022 Nov-Dec;98(6):579-586. doi: 10.1016/j.jped.2022.03.003. Epub 2022 Apr 19. PMID: 35490727; PMCID: PMC9015957.

100. Virant MJ, Luštrek M, Kogoj R, Petrovec M, Uršič T. Changes in HRSV Epidemiology but Not Circulating Variants in Hospitalized Children due to the Emergence of SARS-CoV-2. Viruses. 2023 May 23;15(6):1218. doi: 10.3390/v15061218. PMID: 37376518; PMCID: PMC10302487.

101. Vittucci AC, Piccioni L, Coltella L, Ciarlitto C, Antilici L, Bozzola E, Midulla F, Palma P, Perno CF, Villani A. The Disappearance of Respiratory Viruses in Children during the COVID-19 Pandemic. Int J Environ Res Public Health. 2021 Sep 10;18(18):9550. doi: 10.3390/ijerph18189550. PMID: 34574472; PMCID: PMC8467075.

102. Wang L, Yang S, Yan X, Liu T, Feng Z, Li G. Comparing the yield of oropharyngeal swabs and sputum for detection of 11 common pathogens in hospitalized children with lower respiratory tract infection. Virol J. 2019 Jun 24;16(1):84. doi: 10.1186/s12985-019-1177-x. PMID: 31234918; PMCID: PMC6591818.

103. Watanabe RAS, Cruz JS, de Souza Luna LK, Alves VRG, Conte DD, Lyra L, Nishiyama F, Camargo BS, Bellei N. Respiratory syncytial virus: viral load, viral decay, and disease progression in children with bronchiolitis. Braz J Microbiol. 2022 Sep;53(3):1241-1247. doi: 10.1007/s42770-022-00742-0. Epub 2022 Apr 1. PMID: 35362939; PMCID: PMC9433556.

104. Weldetsadik AY, Riedel F. Respiratory syncytial virus in severe lower respiratory infections in previously healthy young Ethiopian infants. BMC Pediatr. 2021 Apr 28;21(1):201. doi: 10.1186/s12887-021-02675-3. PMID: 33910510; PMCID: PMC8080344.

105. Wen S, Yu M, Zheng G, Lv F, Chen X, Lin L, Li C, Zhang H. Changes in the etiology of viral lower respiratory tract infections in hospitalized children in Wenzhou, China: 2008-2017. J Med Virol. 2020 Aug;92(8):982-987. doi: 10.1002/jmv.25660. Epub 2020 Jan 17. PMID: 31889316.

106. Windsor WJ, Lamb MM, Dominguez SR, Mistry RD, Rao S. Clinical characteristics and illness course based on pathogen among children with respiratory

illness presenting to an emergency department. J Med Virol. 2022 Dec;94(12):6103-6110. doi: 10.1002/jmv.28031. Epub 2022 Aug 4. PMID: 35882541.

107. Xia J, Zhu Y, Bi H, Wu X, Hao M, Chen J, Wu C. Respiratory virus infections among hospitalized children aged 7 years and younger in Wuhan, China, 2017-2021. J Infect. 2023 Jan;86(1):e1-e4. doi: 10.1016/j.jinf.2022.08.044. Epub 2022 Sep 8. PMID: 36087744; PMCID: PMC9451932.

108. Xu M, Liu P, Su L, Cao L, Zhong H, Lu L, Jia R, Xu J. Comparison of Respiratory Pathogens in Children With Lower Respiratory Tract Infections Before and During the COVID-19 Pandemic in Shanghai, China. Front Pediatr. 2022 Jun 30;10:881224. doi: 10.3389/fped.2022.881224. PMID: 35844747; PMCID: PMC9279931.

109. Yan Y, Sun J, Ji K, Guo J, Han L, Li F, Sun Y. High incidence of the virus among respiratory pathogens in children with lower respiratory tract infection in northwestern China. J Med Virol. 2023 Jan;95(1):e28367. doi: 10.1002/jmv.28367. PMID: 36458544; PMCID: PMC9877598.

110. Ye Q, Liu H. Impact of non-pharmaceutical interventions during the COVID-19 pandemic on common childhood respiratory viruses - An epidemiological study based on hospital data. Microbes Infect. 2022 Feb;24(1):104911. doi: 10.1016/j.micinf.2021.104911. Epub 2021 Dec 3. PMID: 34871774; PMCID: PMC8641407.

111. Zhao T, Ye Z, Wang B, Cui Y, Nie Y, Yang B, Chen K, Zhang H, Hu F, Yu F. Virus isolation and genotype identification of human respiratory syncytial virus in Guizhou Province, China. Braz J Infect Dis. 2019 Nov-Dec;23(6):427-434. doi:

10.1016/j.bjid.2019.10.007. Epub 2019 Nov 14. PMID: 31734172; PMCID: PMC9428243.

112. Zhao X, Wang C, Jiang H, Zhang H, Fang F, Chen M, Yuan Z, Teng Z, Liu J, Zhang X. Analysis of circulating respiratory syncytial virus A strains in Shanghai, China identified a new and increasingly prevalent lineage within the dominant ON1 genotype. Front Microbiol. 2022 Aug 11;13:966235. doi: 10.3389/fmicb.2022.966235. PMID: 36033866; PMCID: PMC9403419.
