# Supplementary material for: Impact of Nonpharmaceutical Interventions during the COVID-19 Pandemic on the Prevalence of Respiratory Syncytial Virus in Hospitalized Children with Lower Respiratory Tract Infections: A Systematic Review and Meta-Analysis
Source: Viruses. 2024 Mar 11;16(3):429. doi: 10.3390/v16030429 (PMC10974159; doi:10.3390/v16030429)
Supplement: Supplementary file 1 [file viruses-16-00429-s001.zip › viruses-2827065_Supplementary_Material_B.pdf]

Supplementary Material SB.

Table S1. Reasons for excluding full-text articles from the systematic review.

No.	Studies excluded from the systematic review via databases and registers.	Reasons for excluding
1	Abdulhaq AA, Basode VK, Hashem AM, Alshrari AS, Badroon NA, Hassan AM, Alsubhi TL, Solan Y, Ejeeli S, Azhar EI. Patterns of Human Respiratory Viruses and Lack of MERS-Coronavirus in Patients with Acute Upper Respiratory Tract Infections in Southwestern Province of Saudi Arabia. Adv Virol. 2017;2017:4247853. doi: 10.1155/2017/4247853. Epub 2017 Feb 27. PMID: 28348590; PMCID: PMC5350310.	Period of study before 2015.
2	Abduljabbar HL, Hussein AA, Al-Mayah QS, Aufi IM. Phylogenetic Analysis of Respiratory Syncytial Virus Isolated from Children with Respiratory Tract Infections in Baghdad City, Iraq. J Phys Conf Ser. 2019;1234(1):012082. doi: 10.1088/1742-6596/1234/1/012082.	Inappropriate study design. Abstract for Congress.
3	Abo YN, Clifford V, Lee LY, Costa AM, Crawford N, Wurzel D, Daley AJ. COVID-19 public health measures and respiratory viruses in children in Melbourne. J Paediatr Child Health. 2021 Dec;57(12):1886-1892. doi: 10.1111/jpc.15601. Epub 2021 Jun 3. PMID: 34080245; PMCID: PMC8242487.	Inappropriate population and RSV prevalence not reported.
4	Agha R, Avner JR. Delayed Seasonal RSV Surge Observed During the COVID-19 Pandemic. Pediatrics. 2021 Sep;148(3):e2021052089. doi: 10.1542/peds.2021-052089. Epub 2021 Jun 9. PMID: 34108234.	Inappropriate population and RSV prevalence not reported.
5	Ahmed A, Alsenaidy AM, Mobaireek KF, AlSaadi MM. Viral etiology of acute respiratory infections during 2014-16 in Riyadh, Saudi Arabia. Future Virology. 2022;17(5):269-280. DOI: 10.2217/fvl-2020-0071. Published: May 2022.	Prevalence not reported by RSV season individually.
6	Ahuja N, Gorain S, Pal PP, Das M. Viral Aetiology of Severe Acute Lower Respiratory Tract Infection in Children from the Paediatric Intensive Care Unit at a Tertiary Care Hospital, Eastern India- A Retrospective Study. Journal of Clinical and Diagnostic Research. 2022;16(10):SC06-SC10. DOI: 10.7860/JCDR/2022/57925.17026. Published: Oct 2022. ISSN: 2249-782X, eISSN: 0973-709X.	Prevalence not reported by RSV season individually.
7	Al Amad MA, Al Mahaqri AA, Al Serouri AA, Khader YS. Severe Acute Respiratory Infections With Influenza and Noninfluenza Respiratory Viruses: Yemen, 2011-2016. Inquiry. 2019 Jan-Dec;56:46958019850731. doi: 10.1177/0046958019850731. PMID: 31137990; PMCID: PMC6542124.	Prevalence not reported by RSV season individually.

8	Al Shibli A, Nouredin MB, Al Amri A, Iram D, Narchi H. Epidemiology of Bronchiolitis in Hospitalized Infants at Tawam Hospital, Al Ain, United Arab Emirates. Open Respir Med J. 2021 May 24;15:7-13. doi: 10.2174/1874306402115010007. PMID: 34249176; PMCID: PMC8227460.	Period of study before 2015.
9	Alharbi S. Clinical outcomes of lower respiratory tract infections: an epidemiological study comparing viral and non-viral lower respiratory tract infections in Jeddah. Indo American Journal of Pharmaceutical Sciences. 2019 Mar;6(3):6710-6721. DOI: 10.5281/zenodo.2609207.	Period of study before 2015.
10	Al-Jwadi RF, Mills EHA, Torp-Pedersen C, Andersen MP, Jørgensen IM. "Consequences of COVID-19-related lockdowns and reopenings on emergency hospitalizations in pediatric patients in Denmark during 2020-2021." Eur J Pediatr. 2023 Jan;182(1):285-293. doi: 10.1007/s00431-022-04682-7. Epub 2022 Nov 4. PMID: 36331619; PMCID: PMC9638206.	Incomplete data on RSV prevalence
11	Alkan Ozdemir S, Soysal B, Calkavur S, Gökmen Yıldırım T, Kıymet E, Kalkanlı O, Çolak R, Devrim İ. Is respiratory syncytial virus infection more dangerous than COVID 19 in the neonatal period? J Matern Fetal Neonatal Med. 2022 Nov;35(22):4398-4403. doi: 10.1080/14767058.2020.1849125. Epub 2020 Nov 22. PMID: 33225779.	Seasonality not reported.
12	Alkharsah KR. "The Scope of Respiratory Syncytial Virus Infection in a Tertiary Hospital in the Eastern Province of Saudi Arabia and the Change in Seasonal Pattern during and after the COVID-19 Pandemic." Medicina (Kaunas). 2022 Nov 10;58(11):1623. doi: 10.3390/medicina58111623. PMID: 36363580; PMCID: PMC9693047.	Inappropriate population and RSV prevalence not reported.
13	Alrayes T, Wait A, Spencer P, Merolla DM, Lampe K, Salimnia H, Kannikeswaran N. "Features of an Atypical RSV Surge During the COVID-19 Pandemic." Clin Pediatr (Phila). 2023 May;62(4):265-268. doi: 10.1177/00099228221124677. Epub 2022 Oct 1. PMID: 36189937; PMCID: PMC9527150.	Incomplete data on RSV prevalence
14	Al-Romaihi HE, Smatti MK, Al-Khatib HA, Coyle PV, Ganesan N, Nadeem S, Farag EA, Al Thani AA, Al Khal A, Al Ansari KM, Al Maslamani MA, Yassine HM. Molecular epidemiology of influenza, RSV, and other respiratory infections among children in Qatar: A six years report (2012-2017). Int J Infect Dis. 2020 Jun;95:133-141. doi: 10.1016/j.ijid.2020.04.008. Epub 2020 Apr 9. PMID: 32278934; PMCID: PMC7194828.	Inappropriate population.
15	Altay-Kocak A, Sarzhanova S, Tapisiz A, Dizbay M, Basustaoglu A, Bozdayi G. Retrospective evaluation of viral respiratory tract infections in a university hospital in Ankara, Turkey (2016-2019). J Infect Dev Ctries. 2022 May 30;16(5):857-863. doi: 10.3855/jidc.14427. PMID: 35656958.	Inappropriate population and RSV prevalence not reported.
16	Alvares PA. SARS-CoV-2 and Respiratory Syncytial Virus Coinfection in Hospitalized Pediatric Patients. Pediatr Infect Dis J. 2021 Apr 1;40(4):e164-e166. doi: 10.1097/INF.0000000000003057. PMID: 33464015.	Inappropriate population.

17	Ambati S, Mihic M, Wilkinson K, Sanchez JL, Pezzano C. Impact of COVID-19 on Respiratory Admissions in a Tertiary Pediatric Intensive Care Unit. Cureus. 2022 May 26;14(5):e25369. doi: 10.7759/cureus.25369. PMID: 35765404; PMCID: PMC9233569.	Inappropriate population.
18	An der Heiden M, Buchholz U, Buda S. Estimation of influenza- and respiratory syncytial virus-attributable medically attended acute respiratory infections in Germany, 2010/11-2017/18. Influenza Other Respir Viruses. 2019 Jul 24;13(5):517–21. doi: 10.1111/irv.12666. Epub ahead of print. PMID: 31339223; PMCID: PMC6692544.	Inappropriate population.
19	Anderson EJ, DeVincenzo JP, Simões EAF, Krilov LR, Forbes ML, Pannaraj PS, Espinosa CM, Welliver RC, Wolkoff LI, Yogev R, Checchia PA, Domachowske JB, Halasa N, McBride SJ, Kumar VR, McLaurin KK, Rizzo CP, Ambrose CS. SENTINEL1: Two-Season Study of Respiratory Syncytial Virus Hospitalizations among U.S. Infants Born at 29 to 35 Weeks' Gestational Age Not Receiving Immunoprophylaxis. Am J Perinatol. 2020 Mar;37(4):421-429. doi: 10.1055/s-0039-1681014. Epub 2019 Apr 16. PMID: 30991438.	Incomplete data on RSV prevalence
20	Anderson J, Oeum M, Verkolf E, Licciardi PV, Mulholland K, Nguyen C, Chow K, Waller G, Costa AM, Daley A, Crawford NW, Babl FE, Duke T, Do LAH, Wurzel D. Factors associated with severe respiratory syncytial virus disease in hospitalised children: a retrospective analysis. Arch Dis Child. 2022 Apr;107(4):359-364. doi: 10.1136/archdischild-2021-322435. Epub 2021 Sep 15. PMID: 34526293.	Incomplete data on RSV prevalence
21	Ang LW, Mak TM, Cui L, Leo YS, Lee VJM, Lin RT. Characterisation of respiratory syncytial virus activity in children and adults presenting with acute respiratory illness at primary care clinics in Singapore, 2014-2018. Influenza Other Respir Viruses. 2020 Jul;14(4):412-419. doi: 10.1111/irv.12730. Epub 2020 Feb 24. PMID: 32090482; PMCID: PMC7298310.	Inappropriate population.
22	Appak Ö, Duman M, Belet N, Sayiner AA. Viral respiratory infections diagnosed by multiplex polymerase chain reaction in pediatric patients. J Med Virol. 2019 May;91(5):731-737. doi: 10.1002/jmv.25379. Epub 2019 Jan 3. PMID: 30570759; PMCID: PMC7167103.	Inappropriate population and RSV prevalence not reported.
23	Avolio M, Venturini S, De Rosa R, Crapis M, Basaglia G. Epidemiology of respiratory virus before and during COVID-19 pandemic. Infez Med. 2022 Mar 1;30(1):104-108. doi: 10.53854/liim-3001-12. PMID: 35350252; PMCID: PMC8929737.	Inappropriate population.
24	Awad S, Khader Y, Mansi M, Yusef D, Alawadin S, Qudah W, Khasawneh R. Viral Surveillance of Children with Acute Respiratory Infection in Two Main Hospitals in Northern Jordan, Irbid, during Winter of 2016. J Pediatr Infect Dis. 2020 Jan;15(1):1-10. doi: 10.1055/s-0039-1692972. Epub 2019 Jul 4. PMID: 32300275; PMCID: PMC7117070.	Seasonality not reported.
25	Aygün D, Erbek F, Kuşkucu M, Şener D, Köşker M, Varol F, Midilli K, Çokuğraş H, Camcıoğlu Y. The epidemiologic and clinical features of viral agents among hospitalized children with lower respiratory tract infections. Turk Pediatri Ars. 2020 Jun 19;55(2):166-173. doi: 10.14744/TurkPediatriArs.2020.39114. PMID: 32684762; PMCID: PMC7344125.	Prevalence not reported by RSV season individually.

26	Babachenko IV, Samodova OV, Anokhin VA, Mikhaylova EV, Bogdanova AV, Evdokimov KV, Sharipova EV, Rogushina NL, Khaliullina SV, Chudakova TK, Yarushkina MS, Grigor'ev SG. Clinical and epidemiological characteristics of respiratory syncytial virus infection in children the first year of life. J Infektol [Internet]. 2018;10(3):70-6. Available from: www.scopus.com	Language not in English.
27	Baker RE, Park SW, Yang W, Vecchi GA, Metcalf CJE, Grenfell BT. The impact of COVID-19 nonpharmaceutical interventions on the future dynamics of endemic infections. Proc Natl Acad Sci U S A. 2020 Dec 1;117(48):30547-30553. doi: 10.1073/pnas.2013182117. Epub 2020 Nov 9. PMID: 33168723; PMCID: PMC7720203.	Inappropriate population.
28	Barbosa J, Parra B, Alarcón L, Quiñones FI, López E, Cortés MAF. Prevalence and periodicity of respiratory syncytial virus in Colombia. Rev Aca Colomb Cien Exact Fisicas Natur. 2017;41(161):435-46. Available from: www.scopus.com	Language not in English.
29	Bardsley M, Morbey RA, Hughes HE, Beck CR, Watson CH, Zhao H, Ellis J, Smith GE, Elliot AJ. "Epidemiology of respiratory syncytial virus in children younger than 5 years in England during the COVID-19 pandemic, measured by laboratory, clinical, and syndromic surveillance: a retrospective observational study." Lancet Infect Dis. 2023 Jan;23(1):56-66. doi: 10.1016/S1473-3099(22)00525-4. Epub 2022 Sep 2. PMID: 36063828; PMCID: PMC9762748.	Incomplete data on RSV prevalence
30	Baumeister E, Duque J, Varela T, Palekar R, Couto P, Savy V, Giovacchini C, Haynes AK, Rha B, Arriola CS, Gerber SI, Azziz-Baumgartner E. Timing of respiratory syncytial virus and influenza epidemic activity in five regions of Argentina, 2007-2016. Influenza Other Respir Viruses. 2019 Jan;13(1):10-17. doi: 10.1111/irv.12596. Epub 2018 Nov 20. PMID: 30051595; PMCID: PMC6304310.	Prevalence not reported by RSV season individually.
31	Becerra-Gutiérrez LK, Aguilar-Gamboa FR, Reynoso-Tantaleán JL, Mera-Villasis KM. Infección por virus sincicial respiratorio. Reporte de casos de pacientes en área crítica pediátrica de un hospital del norte de Perú. Rev. Cuerpo Med. HNAAA [Internet]. 2022 Jun 20 [citado 2022 Jun 20];15(1):135-40. DOI: https://doi.org/10.35434/rcmhnaaa.2022.151.950.	Inappropriate study design. Case report.
32	Bermúdez Barrezueta L, Matías Del Pozo V, López-Casillas P, Brezmes Raposo M, Gutiérrez Zamorano M, Pino Vázquez MA. Variation in the seasonality of the respiratory syncytial virus during the COVID-19 pandemic. Infection. 2022 Aug;50(4):1001-1005. doi: 10.1007/s15010-022-01794-y. Epub 2022 Mar 22. PMID: 35316529; PMCID: PMC8938970.	Duplicate with Bermúdez Barrezueta et al. Enferm Infecc Microbiol Clin (Engl Ed). 2023;41(6):348-351. doi:10.1016/j.eimce.2022.09.001

33	Benítez-Guerra D, Piña-Flores C, Zamora-López M, Escalante-Padrón F, Lima- Rogel V, González-Ortiz AM, Guevara-Tovar M, Bernal-Silva S, Benito-Cruz B, Castillo-Martínez F, Martínez-Rodríguez LE, Ramírez-Ojeda V, Tello-Martínez N, Lomelí-Valdez R, Salto-Quintana J, Cadena-Mota S, Noyola DE. Respiratory syncytial virus acute respiratory infection-associated hospitalizations in preterm Mexican infants: A cohort study. Influenza Other Respir Viruses. 2020 Mar;14(2):182-188. doi: 10.1111/irv.12708. Epub 2020 Jan 9. PMID: 31917902; PMCID: PMC7040972.	Prevalence not reported by RSV season individually.
34	Berg AS, Inchley CS, Aase A, Fjaerli HO, Bull R, Aaberge I, Leegaard TM, Nakstad B. Etiology of Pneumonia in a Pediatric Population with High Pneumococcal Vaccine Coverage: A Prospective Study. Pediatr Infect Dis J. 2016 Mar;35(3):e69-75. doi: 10.1097/INF.0000000000001009. PMID: 26599568.	Period of study before 2015.
35	Berksoy E, Kanik A, Çiçek A, Bardak Ş, Elibol P, Demir G, Yilmaz N, Nalbant T, Gökalp G, Yilmaz Çiftdoğan D. Clinical and laboratory characteristics of children with SARS-CoV-2 infection. Pediatr Pulmonol. 2021 Dec;56(12):3674-3681. doi: 10.1002/ppul.25654. Epub 2021 Sep 13. PMID: 34516721; PMCID: PMC8661911.	Inappropriate population.
36	Bermúdez Barrezueta L, Gutiérrez Zamorano M, López-Casillas P, Brezmes-Raposo M, Sanz Fernández I, Pino Vázquez MA. Influencia de la pandemia COVID-19 sobre la epidemiología de la bronquiolitis aguda [Influence of the COVID-19 pandemic on the epidemiology of acute bronchiolitis]. Enferm Infecc Microbiol Clin. 2023 Jun-Jul;41(6):348-351. Spanish. doi: 10.1016/j.eimc.2021.11.014. Epub 2021 Dec 22. PMID: 34955579; PMCID: PMC8692059.	Language not in English.
37	Bi J, Deng G, Su Q, Deng J. Clinical study of acute lower respiratory tract infection caused by respiratory syncytial virus in neonates. Chin J Appl Clin Pediatr. 2021;36(24):1871-5. Available from: www.scopus.com	Language not in English.
38	Bigna JJ, Kenmoe S, Well EA, Simo FBN, Penlap VB, Vabret A, Njouom R. Contemporaneous data on the prevalence of Human Respiratory Syncytial Virus infection in people with acute respiratory tract infections in Africa (2000-2017). Data Brief. 2018 Aug 22;20:940-947. doi: 10.1016/j.dib.2018.08.039. PMID: 30225305; PMCID: PMC6138983.	Incomplete data on RSV prevalence
39	Billard MN, van de Ven PM, Baraldi B, Kragten-Tabatabaie L, Bont LJ, Wildenbeest JG. International changes in respiratory syncytial virus (RSV) epidemiology during the COVID-19 pandemic: Association with school closures. Influenza Other Respir Viruses. 2022 Sep;16(5):926-936. doi: 10.1111/irv.12998. Epub 2022 Jun 22. PMID: 35733362; PMCID: PMC9343326.	Inappropriate population and RSV prevalence not reported.
40	Bimouhen A, El Falaki F, Ihazmad H, Regragui Z, Benkerroum S, Barakat A. Circulation of Respiratory Syncytial Virus in Morocco during 2014-2016: Findings from a sentinel-based virological surveillance system for influenza. East Mediterr Health J. 2016 Oct 2;22(7):483-490. PMID: 27714743.	Inappropriate population and

		seasonality not reported.
41	Boender TS, Cai W, Schranz M, Kocher T, Wagner B, Ullrich A, Buda S, Zöllner R, Greiner F, Diercke M, Grabenhenrich L. Using routine emergency department data for syndromic surveillance of acute respiratory illness, Germany, week 10 2017 until week 10 2021. Euro Surveill. 2022 Jul;27(27):2100865. doi: 10.2807/1560-7917.ES.2022.27.27.2100865. PMID: 35801521; PMCID: PMC9264729.	Inappropriate population and RSV prevalence not reported.
42	Bögli J, Güsewell S, Strässle R, Kahlert CR, Albrich WC. "Pediatric hospital admissions, case severity, and length of hospital stay during the first 18 months of the COVID-19 pandemic in a tertiary children's hospital in Switzerland." Infection. 2023 Apr;51(2):439-446. doi: 10.1007/s15010-022-01911-x. Epub 2022 Sep 5. PMID: 36065045; PMCID: PMC9444086.	Inappropriate population and RSV prevalence not reported.
43	Bowyer SA, Bryant WA, Key D, Booth J, Briggs L, Spiridou A, Cortina-Borja M, Davies G, Taylor AM, Sebire NJ. Machine learning forecasting for COVID-19 pandemic-associated effects on paediatric respiratory infections. Arch Dis Child. 2022 Dec;107(12):e36. doi: 10.1136/archdischild-2022-323822. Epub 2022 Aug 10. PMID: 35948401; PMCID: PMC9685698.	Inappropriate population and RSV prevalence not reported.
44	Bridge R, Brady S, Erhart LM, Komatsu K. Notes from the Field: Age Distribution of Patients with Laboratory-Detected Respiratory Syncytial Virus - Arizona, 2013-2017. MMWR Morb Mortal Wkly Rep. 2019 Mar 1;68(8):203-204. doi: 10.15585/mmwr.mm6808a4. PMID: 30817747; PMCID: PMC6394387.	Inappropriate population.
45	Buchan SA, Chung H, Karnauchow T, McNally JD, Campitelli MA, Gubbay JB, Katz K, McGeer AJ, Richardson DC, Richardson SE, Simor A, Smieja M, Zahariadis G, Tran D, Crowcroft NS, Rosella LC, Kwong JC. Characteristics and Outcomes of Young Children Hospitalized With Laboratory-confirmed Influenza or Respiratory Syncytial Virus in Ontario, Canada, 2009-2014. Pediatr Infect Dis J. 2019 Apr;38(4):362-369. doi: 10.1097/INF.0000000000002164. PMID: 30882725.	Period of study before 2015.
46	Cai W, Buda S, Schuler E, Hirve S, Zhang W, Haas W. Risk factors for hospitalized respiratory syncytial virus disease and its severe outcomes. Influenza Other Respir Viruses. 2020 Nov;14(6):658-670. doi: 10.1111/irv.12729. Epub 2020 Feb 16. PMID: 32064773; PMCID: PMC7578333.	Prevalence not reported by RSV season individually.
47	Cai W, Dürrwald R, Biere B, Schweiger B, Haas W, Wolff T, Buda S, Reiche J. Determination of respiratory syncytial virus epidemic seasons by using 95% confidence interval of positivity rates, 2011-2021, Germany. Influenza Other Respir Viruses. 2022 Sep;16(5):854-857. doi: 10.1111/irv.12996. Epub 2022 Apr 29. PMID: 35485999; PMCID: PMC9343324.	Inappropriate population.

48	Caini S, de Mora D, Olmedo M, Portugal D, Becerra MA, Mejía M, Pacurucu MC, Ojeda J, Bonaccorsi G, Lorini C, Paget J, Bruno A. The epidemiology and severity of respiratory viral infections in a tropical country: Ecuador, 2009-2016. J Infect Public Health. 2019 May-Jun;12(3):357-363. doi: 10.1016/j.jiph.2018.12.003. Epub 2018 Dec 17. PMID: 30573330; PMCID: PMC7102740.	Inappropriate population.
49	Caini S, Stolyarov K, Sominina A, Smorodintseva E, Staadegaard L, Paget J, Danilenko D. A comparative analysis of the epidemiology of influenza and respiratory syncytial virus in Russia, 2013/14 to 2018/19. J Glob Health. 2022 Feb 5;12:04009. doi: 10.7189/jogh.12.04009. PMID: 35136600; PMCID: PMC8818296.	Incomplete data on RSV prevalence
50	Calderaro A, De Conto F, Buttrini M, Piccolo G, Montecchini S, Maccari C, Martinelli M, Di Maio A, Ferraglia F, Pinardi F, Montagna P, Arcangeletti MC, Chezzi C. Human respiratory viruses, including SARS-CoV-2, circulating in the winter season 2019-2020 in Parma, Northern Italy. Int J Infect Dis. 2021 Jan;102:79-84. doi: 10.1016/j.ijid.2020.09.1473. Epub 2020 Oct 2. PMID: 33017694; PMCID: PMC7530558.	Seasonality not reported.
51	Callahan ZY, Smith TK, Ingersoll C, Gardner R, Korgenski EK, Sloan CD. Comparative Seasonal Respiratory Virus Epidemic Timing in Utah. Viruses. 2020 Feb 29;12(3):275. doi: 10.3390/v12030275. PMID: 32121465; PMCID: PMC7150790.	Inappropriate population.
52	Calvo C, Moreno-Pérez D, Miguélez SA, Álvarez CA, González FA, Fernández JA, et al. Epidemiology and clinical evidence of infection by respiratory syncytial virus in children over 2 of age. National multi-center study (FIVE). Acta Pediatr Esp. 2015;73(1):5-9.	Period of study before 2015.
53	Cardenas J, Pringle C, Filipp SL, Gurka MJ, Ryan KA, Avery KL. Changes in Critical Bronchiolitis After COVID-19 Lockdown. Cureus. 2022 May 17;14(5):e25064. doi: 10.7759/cureus.25064. PMID: 35719813; PMCID: PMC9203253.	Diagnostic technique not reported.
54	Caserta MT, Yang H, Gill SR, Holden-Wiltse J, Pryhuber G. Viral Respiratory Infections in Preterm Infants during and after Hospitalization. J Pediatr. 2017 Mar;182:53-58.e3. doi: 10.1016/j.jpeds.2016.11.077. Epub 2016 Dec 30. PMID: 28041669; PMCID: PMC5328856.	Seasonality not reported.
55	Cason C, Zamagni G, Cozzi G, Tonegutto D, Ronfani L, Oretti C, De Manzini A, Barbi E, Comar M, Amaddeo A. Spread of Respiratory Pathogens During the COVID-19 Pandemic Among Children in the Northeast of Italy. Front Microbiol. 2022 Mar 24;13:804700. doi: 10.3389/fmicb.2022.804700. PMID: 35401434; PMCID: PMC8988150.	Inappropriate population.
56	Castagno E, Raffaldi I, Del Monte F, Garazzino S, Bondone C. "New epidemiological trends of respiratory syncytial virus bronchiolitis during COVID-19 pandemic." World J Pediatr. 2023 May;19(5):502-504. doi: 10.1007/s12519-022-00623-4. Epub 2022 Sep 26. PMID: 36163542; PMCID: PMC9512956.	Seasonality not reported.
57	Cattoir L, Vankeerberghen A, Boel A, Van Vaerenbergh K, De Beenhouwer H. Epidemiology of RSV and hMPV in Belgium: a 10-year follow-up. Acta Clin Belg. 2019 Aug;74(4):229-235. doi: 10.1080/17843286.2018.1492509. Epub 2018 Jul 20. PMID: 30029583.	Prevalence not reported by RSV season individually.

58	Çelebi Ö, Çelebi D. Viral Respiratory Tract Pathogens During the COVID-19 Pandemic. Eurasian J Med. 2021 Jun;53(2):123-126. doi: 10.5152/eurasianjmed.2021.20459. PMID: 34177295; PMCID: PMC8184047.	Inappropriate population.
59	Cerar S, Pirnovar V. A comparative analysis of the occurrence of lower respiratory tract infections caused by respiratory syncytial virus among newborns in the years before and during the COVID-19 pandemic at a tertiary referral hospital in Slovenia. Central Eur J Paed [Internet]. 2022;18(2):100-7. Available from: www.scopus.com	Inappropriate population.
60	Cerone JB, Santos RP, Tristram D, Lamson DM, Stellrecht KA, St George K, Horgan MJ, Rios A. Incidence of respiratory viral infection in infants with respiratory symptoms evaluated for late-onset sepsis. J Perinatol. 2017 Aug;37(8):922-926. doi: 10.1038/jp.2017.69. Epub 2017 May 18. PMID: 28518131; PMCID: PMC7100264.	Inappropriate population.
61	Chadha M, Hirve S, Bancej C, Barr I, Baumeister E, Caetano B, Chittaganpitch M, Darmaa B, Ellis J, Fasce R, Kadjo H, Jackson S, Leung V, Pisareva M, Moyes J, Naguib A, Tivane A, Zhang W; WHO RSV Surveillance Group. Human respiratory syncytial virus and influenza seasonality patterns-Early findings from the WHO global respiratory syncytial virus surveillance. Influenza Other Respir Viruses. 2020 Nov;14(6):638-646. doi: 10.1111/irv.12726. Epub 2020 Mar 12. PMID: 32163226; PMCID: PMC7578323.	Incomplete data on RSV prevalence
62	Chakhunashvili G, Wagner AL, Power LE, Janusz CB, Machablishvili A, Karseladze I, Tarkhan-Mouravi O, Zakhashvili K, Imnadze P, Gray GC, Anderson B, Boulton ML. Severe Acute Respiratory Infection (SARI) sentinel surveillance in the country of Georgia, 2015-2017. PLoS One. 2018 Jul 30;13(7):e0201497. doi: 10.1371/journal.pone.0201497. PMID: 30059540; PMCID: PMC6066249.	Prevalence not reported by RSV season individually.
63	Chavez D, Gonzales-Armayo V, Mendoza E, Palekar R, Rivera R, Rodriguez A, Salazar C, Veizaga A, Añez A. Estimation of influenza and respiratory syncytial virus hospitalizations using sentinel surveillance data-La Paz, Bolivia. 2012-2017. Influenza Other Respir Viruses. 2019 Sep;13(5):477-483. doi: 10.1111/irv.12663. Epub 2019 Jun 17. PMID: 31206257; PMCID: PMC6692540.	Incomplete data on RSV prevalence
64	Chawla K, Kumar A, Hegde A, Govindakarnavar A. A pilot study on the etiology of acute lower respiratory tract infections among children hospitalized for respiratory illness at a rural hospital in South Coastal Karnataka. Biomed Pharmacol J. 2022;15(2).	Seasonality not reported.
65	Checovich MM, Barlow S, Shult P, Reisdorf E, Temte JL. Evaluation of Viruses Associated With Acute Respiratory Infections in Long-Term Care Facilities Using a Novel Method: Wisconsin, 2016–2019. J Am Med Dir Assoc. 2020 Jan;21(1):29-33. doi: 10.1016/j.jamda.2019.09.003. Epub 2019 Oct 19. PMID: 31636034; PMCID: PMC7106273.	Inappropriate population.
66	Chirinos-Saire Y, Reyna-García R, Aguilar-Huauya E, Santillán-Salas C. Respiratory viruses and clinical-epidemiological characteristics in episodes of acute respiratory infection. Rev Peru Med Exp Salud Publica. 2021 Jan-Mar;38(1):101-107. Spanish, English. doi: 10.17843/rpmesp.2021.381.6346. Epub 2021 Jun 25. PMID: 34190900.	Inappropriate population.

67	Chiu SS, Cowling BJ, Peiris JSM, Chan ELY, Wong WHS, Lee KP. Effects of Nonpharmaceutical COVID-19 Interventions on Pediatric Hospitalizations for Other Respiratory Virus Infections, Hong Kong. Emerg Infect Dis. 2022 Jan;28(1):62-68. doi: 10.3201/eid2801.211099. PMID: 34932446; PMCID: PMC8714236.	Incomplete data on RSV prevalence
68	Choe YJ, Smit MA, Mermel LA. Comparison of Common Respiratory Virus Peak Incidence Among Varying Age Groups in Rhode Island, 2012-2016. JAMA Netw Open. 2020 May 1;3(5):e207041. doi: 10.1001/jamanetworkopen.2020.7041. PMID: 32401314; PMCID: PMC7221508.	Inappropriate population and seasonality not reported.
69	Chou CA, Lin TI, Chen YS, Liu PY, Huang YF, Chen YY, Hsieh KS, Chen YS, Ger LP. Comparisons of etiology and diagnostic tools of lower respiratory tract infections in hospitalized young children in Southern Taiwan in two seasons. J Microbiol Immunol Infect. 2016 Aug;49(4):539-45. doi: 10.1016/j.jmii.2014.08.029. Epub 2014 Nov 1. PMID: 25442857.	Period of study before 2015.
70	Chow EJ, Uyeki TM, Chu HY. "The effects of the COVID-19 pandemic on community respiratory virus activity." Nat Rev Microbiol. 2023 Mar;21(3):195-210. doi: 10.1038/s41579-022-00807-9. Epub 2022 Oct 17. PMID: 36253478; PMCID: PMC9574826.	Inappropriate study design. Review.
71	Christou E, Bourousis E, Pouliakis A, Douros K, Varela P, Delis D, Priftis KN. "The Differences Between RSV and no RSV Acute Bronchiolitis in Hospitalized Infants: A Cross-Sectional Study." Glob Pediatr Health. 2022 Dec 2;9:2333794X221138437. doi: 10.1177/2333794X221138437. PMID: 36479308; PMCID: PMC9720787.	Prevalence not reported by RSV season individually.
72	Chu HY, Katz J, Tielsch J, Khatry SK, Shrestha L, LeClerq SC, Magaret A, Kuypers J, Steinhoff M, Englund JA. Respiratory syncytial virus infection in infants in rural Nepal. J Infect. 2016 Aug;73(2):145-54. doi: 10.1016/j.jinf.2016.05.007. Epub 2016 May 27. PMID: 27241525; PMCID: PMC4942356.	Period of study before 2015.
73	Comte A, Bour JB, Darniot M, Pitoiset C, Aho-Glélé LS, Manoha C. Epidemiological characteristics and clinical outcomes of human rhinovirus infections in a hospitalized population. Severity is independently linked to RSV coinfection and comorbidities. J Clin Virol. 2020 Apr;125:104290. doi: 10.1016/j.jcv.2020.104290. Epub 2020 Feb 24. PMID: 32135487.	Inappropriate population.
74	Correia W, Dorta-Guerra R, Sanches M, Almeida Semedo CJB, Valladares B, de Pina-Araújo IIM, Carmelo E. Study of the Etiology of Acute Respiratory Infections in Children Under 5 Years at the Dr. Agostinho Neto Hospital, Praia, Santiago Island, Cabo Verde. Front Pediatr. 2021 Sep 28;9:716351. doi: 10.3389/fped.2021.716351. PMID: 34650939; PMCID: PMC8505963.	Seasonality not reported.
75	Costa LF, Da Silveira HL, Queiróz DAO, Mantese OC, Yokosawa J. Respiratory virus infections in hospitalized and non-hospitalized children: determinants of severe course of the disease. J Infect Dev Ctries. 2022 Jan 31;16(1):196-205. doi: 10.3855/jidc.15117. PMID: 35192538.	Prevalence not reported by RSV season individually.

76	Cozzi G, Cortellazzo Wiel L, Amaddeo A, Gatto A, Giangreco M, Klein-Kremer A, Bosis S, Silvagni D, Debbia C, Nanni L, Chiappa S, Minute M, Corsini I, Morabito G, Gortan AJ, Colombo M, Marchetti F, Garelli D, Piffer A, Cardinale F, Levy N, Curatola A, Gojsina B, Basu S, Barbi E, Sovtic A; SARS-CoV-2 bronchiolitis study group. Prevalence of SARS-CoV-2 positivity in infants with bronchiolitis: a multicentre international study. Arch Dis Child. 2022 Jun 15:archdischild-2021-323559. doi: 10.1136/archdischild-2021-323559. Epub ahead of print. PMID: 35705371; PMCID: PMC9240451.	Inappropriate population.
77	Cui A, Xie Z, Xu J, Hu K, Zhu R, Li Z, Li Y, Sun L, Xiang X, Xu B, Zhang R, Gao Z, Zhang Y, Xu W. "Comparative analysis of the clinical and epidemiological characteristics of human influenza virus versus human respiratory syncytial virus versus human metapneumovirus infection in nine provinces of China during 2009-2021." J Med Virol. 2022 Dec;94(12):5894-5903. doi: 10.1002/jmv.28073. Epub 2022 Aug 29. PMID: 35981880.	Incomplete data on RSV prevalence
78	Da Cunha GZ, De Souza EM, Dos Santos MLA. Bronchiolitis Hospitalization in Southern Brazil from 2002 to 2012: A Count Time-series Approach. Revista da Sociedade Brasileira de Medicina Tropical. 2019;52:e20180329. DOI: 10.1590/0037-8682-0329-2018.	Inappropriate population.
79	Dang JL, Zhao JJ. Viral respiratory tract infections and their correlation with clinical presentations and outcomes among young children attending emergency department of tertiary care hospital in China. BIOMEDICAL RESEARCH-INDIA. 2017;28(5):2327-2333. Accession Number: WOS:000396838200076. ISSN: 0970-938X, eISSN: 0976-1683	Period of study before 2015.
80	Davis W, Duque J, Huang QS, Olson N, Grant CC, Newbern EC, Thompson M, Waite B, Prasad N, Trenholme A, Azziz-Baumgartner E. Sensitivity and specificity of surveillance case definitions in detection of influenza and respiratory syncytial virus among hospitalized patients, New Zealand, 2012-2016. J Infect. 2022 Feb;84(2):216-226. doi: 10.1016/j.jinf.2021.12.012. Epub 2021 Dec 22. PMID: 34953903.	Inappropriate population and seasonality not reported.
81	Davydova I.V, Degtyareva E.A., Keshishyan E.S., et al. Clinical outcomes of hospitalization of premature infants (gestational age 33–35 weeks) with lower respiratory tract infections, associated and not associated with the respiratory syncytial virus, in the PONI international study. Pediatria. 2017; 96 (4): 8–15.	Language not in English.
82	de Carvalho FC, da Silva ET, de Almeida WAF, Maroneze MA, Schwartz JA, Jardim JPV, Peixoto HM. Clinical and epidemiological aspects of severe acute respiratory infection: before and during the first year of the COVID-19 pandemic in Brazil. Trans R Soc Trop Med Hyg. 2023 Mar 1;117(3):161-173. doi: 10.1093/trstmh/trac074. PMID: 35929810; PMCID: PMC9384673.	Inappropriate population and RSV prevalence not reported.
83	De Luca M, D'Amore C, Romani L, Tripiciano C, Clemente V, Mercadante S, Perrotta D, Nunziata J, Cecchetti C, Rossetti E, Bianchi R, Perno CF, Bernaschi P, Russo C, Lancella L, Raponi M, Ciofi Degli Atti ML. "Severe viral respiratory infections in the pre-COVID era: A 5-year	Incomplete data on RSV prevalence

	experience in two pediatric intensive care units in Italy." Influenza Other Respir Viruses. 2023 Jan;17(1):e13038. doi: 10.1111/irv.13038. Epub 2022 Oct 3. PMID: 36193621; PMCID: PMC9835419.	
84	Dearden CX, Jeevarathnum AC, Havinga J, Green RJ. The epidemiology of respiratory syncytial virus: A retrospective review from Steve Biko Academic Hospital 2013 - 2016. Afr J Thorac Crit Care Med. 2018 Apr 3;24(1):10.7196/AJTCCM.2017.v24i1.163. doi: 10.7196/AJTCCM.2017.v24i1.163. PMID: 34541489; PMCID: PMC8432915.	Inappropriate population.
85	Delestrain C, Danis K, Hau I, Behillil S, Billard MN, Krajten L, Cohen R, Bont L, Epaud R. Impact of COVID-19 social distancing on viral infection in France: A delayed outbreak of RSV. Pediatr Pulmonol. 2021 Dec;56(12):3669-3673. doi: 10.1002/ppul.25644. Epub 2021 Sep 2. PMID: 34473914; PMCID: PMC8662089.	Inappropriate population and RSV prevalence not reported.
86	Doroshenko A, Lee N, MacDonald C, Zelyas N, Asadi L, Kanji JN. Decline of Influenza and Respiratory Viruses With COVID-19 Public Health Measures: Alberta, Canada. Mayo Clin Proc. 2021 Dec;96(12):3042-3052. doi: 10.1016/j.mayocp.2021.09.004. Epub 2021 Sep 20. PMID: 34863395; PMCID: PMC8450272.	Inappropriate population and RSV prevalence not reported.
87	Du X, Wu G, Zhu Y, Zhang S. Exploring the epidemiological changes of common respiratory viruses since the COVID-19 pandemic: a hospital study in Hangzhou, China. Arch Virol. 2021 Nov;166(11):3085-3092. doi: 10.1007/s00705-021-05214-8. Epub 2021 Sep 4. PMID: 34480636; PMCID: PMC8417671.	Inappropriate population.
88	Duan Y, He J, Cui Y, Li W, Jiang Y. Characteristics and forecasting of respiratory viral epidemics among children in west China. Medicine (Baltimore). 2021 Apr 23;100(16):e25498. doi: 10.1097/MD.0000000000025498. PMID: 33879683; PMCID: PMC8078258.	Inappropriate population.
89	Durigon EL, Botosso VF, de Oliveira DBL. Human respiratory syncytial virus: Biology, epidemiology, and control. In: Human Virology in Latin America: From Biology to Control. 2017. p. 235-54. doi: 10.1007/978-3-319-54567-7_12.	Inappropriate study design. Book chapter.
90	Duyu M, Karakaya Z. VIRAL ETIOLOGY AND OUTCOME OF SEVERE LOWER RESPIRATORY TRACT INFECTIONS AMONG CRITICALLY ILL CHILDREN ADMITTED TO THE PICU. Med Intensiva (Engl Ed). 2020 May 13. doi: 10.1016/j.medin.2020.04.023. Epub ahead of print. PMID: 32405129; PMCID: PMC7218367.	Prevalence not reported by RSV season individually.
91	Eden JS, Sikazwe C, Xie R, Deng YM, Sullivan SG, Michie A, Levy A, Cutmore E, Blyth CC, Britton PN, Crawford N, Dong X, Dwyer DE, Edwards KM, Horsburgh BA, Foley D, Kennedy K, Minney-Smith C, Speers D, Tulloch RL, Holmes EC, Dhanasekaran V, Smith DW, Kok J,	Inappropriate population.

	Barr IG; Australian RSV study group. Off-season RSV epidemics in Australia after easing of COVID-19 restrictions. Nat Commun. 2022 May 24;13(1):2884. doi: 10.1038/s41467-022-30485-3. PMID: 35610217; PMCID: PMC9130497.	
92	El Kholy AA, Mostafa NA, Ali AA, Soliman MM, El-Sherbini SA, Ismail RI, El Basha N, Magdy RI, El Rifai N, Hamed DH. The use of multiplex PCR for the diagnosis of viral severe acute respiratory infection in children: a high rate of co-detection during the winter season. Eur J Clin Microbiol Infect Dis. 2016 Oct;35(10):1607-13. doi: 10.1007/s10096-016-2698-5. Epub 2016 Jun 10. PMID: 27287764; PMCID: PMC7088036.	Period of study before 2015.
93	El-Heneidy A, Ware RS, Robson JM, Cherian SG, Lambert SB, Grimwood K. Respiratory virus detection during the COVID-19 pandemic in Queensland, Australia. Aust N Z J Public Health. 2022 Feb;46(1):10-15. doi: 10.1111/1753-6405.13168. Epub 2021 Oct 14. PMID: 34648214; PMCID: PMC8652525.	Inappropriate population and RSV prevalence not reported.
94	Elliott SP, Ray CG. Viral Infections of the Lower Respiratory Tract. Pediatric Respiratory Medicine. 2008;481-489. doi:10.1016/B978-032304048-8.50037-2	Inappropriate study design. Book chapter.
95	El-Nawawy A, Antonios MA, Meheissen MA, Fahim MM. Respiratory viruses associated with severe mechanically ventilated pneumonia in children. J Med Virol. 2022 Feb;94(2):461-468. doi: 10.1002/jmv.27284. Epub 2021 Aug 30. PMID: 34415627; PMCID: PMC8426888.	Inappropriate population.
96	Engels G, Sack J, Weissbrich B, Hartmann K, Knies K, Härtel C, Streng A, Dölken L, Liese JG; CoPraKid Study Group. Very Low Incidence of SARS-CoV-2, Influenza and RSV but High Incidence of Rhino-, Adeno- and Endemic Coronaviruses in Children With Acute Respiratory Infection in Primary Care Pediatric Practices During the Second and Third Wave of the SARS-CoV-2 Pandemic. Pediatr Infect Dis J. 2022 Apr 1;41(4):e146-e148. doi: 10.1097/INF.0000000000003460. PMID: 35175993; PMCID: PMC8919947.	Inappropriate population.
97	Fairweather SM, Chang CL, Mansell CJ, Shafuddin E, Hancox RJ. Impact of COVID-19 pandemic restrictions on the cardio-respiratory health of New Zealanders. Respirology. 2021 Nov;26(11):1041-1048. doi: 10.1111/resp.14119. Epub 2021 Aug 8. PMID: 34365699; PMCID: PMC8447448.	Inappropriate population.
98	Falsey AR, Cameron A, Branche AR, Walsh EE. "Perturbations in Respiratory Syncytial Virus Activity During the SARS-CoV-2 Pandemic." J Infect Dis. 2022 Dec 28;227(1):83-86. doi: 10.1093/infdis/jiac434. PMID: 36315855.	Inappropriate population and RSV prevalence not reported.

99	Fedorczak A, Zielińska N, Nosek-Wasilewska P, Mikołajczyk K, Lisiak J, Zeman K, Tkaczyk M. "Comparison of COVID-19 and RSV Infection Courses in Infants and Children under 36 Months Hospitalized in Paediatric Department in Fall and Winter Season 2021/2022." J Clin Med. 2022 Nov 29;11(23):7088. doi: 10.3390/jcm11237088. PMID: 36498663; PMCID: PMC9741168.	Incomplete data on RSV prevalence
100	Fergie J, Suh M, Jiang X, Fryzek JP, Gonzales T. Respiratory Syncytial Virus and All-Cause Bronchiolitis Hospitalizations Among Preterm Infants Using the Pediatric Health Information System (PHIS). J Infect Dis. 2022 Apr 1;225(7):1197-1204. doi: 10.1093/infdis/jiaa435. PMID: 32691037; PMCID: PMC8974836.	Prevalence not reported by RSV season individually.
101	Fernandes-Matano L, Monroy-Muñoz IE, Angeles-Martínez J, Sarquiz-Martinez B, Palomec-Nava ID, Pardavé-Alejandre HD, Santos Coy-Arechavaleta A, Santacruz- Tinoco CE, González-Ibarra J, González-Bonilla CR, Muñoz-Medina JE. Prevalence of non-influenza respiratory viruses in acute respiratory infection cases in Mexico. PLoS One. 2017 May 3;12(5):e0176298. doi: 10.1371/journal.pone.0176298. PMID: 28467515; PMCID: PMC5415110.	Period of study before 2015.
102	Fernandez-Sarmiento J, Corrales SC, Obando E, Amin J, Bastidas Goyes A, Barrera Lopez PA, Bernal Ortiz N. Factors Associated with Severe Acute Respiratory Infections Due to Rhinovirus/Enterovirus Complex in Children and Their Comparison with Those of Respiratory Syncytial Virus. Arch Pediatr Infect Dis. 2022 Apr;10(2):e115548. Published online 2021 Nov 17. doi: 10.5812/pedinfect.115548.	Prevalence not reported by RSV season individually.
103	Fieldhouse JK, Toh TH, Lim WH, Ting J, Ha SJ, Hii KC, Kong CI, Wong TM, Wong SC, Warkentien TE, Gray GC. Surveillance for respiratory syncytial virus and parainfluenza virus among patients hospitalized with pneumonia in Sarawak, Malaysia. PLoS One. 2018 Aug 15;13(8):e0202147. doi: 10.1371/journal.pone.0202147. PMID: 30110367; PMCID: PMC6093684.	Seasonality not reported.
104	Fillatre A, François C, Segard C, Duverlie G, Hecquet D, Pannier C, Roussel C, Zawadzki P, Brochot E, Castelain S. Epidemiology and seasonality of acute respiratory infections in hospitalized children over four consecutive years (2012-2016). J Clin Virol. 2018 May;102:27-31. doi: 10.1016/j.jcv.2018.02.010. Epub 2018 Feb 20. PMID: 29477833; PMCID: PMC7106524.	Prevalence not reported by RSV season individually.
105	Foley DA, Phuong LK, Peplinski J, Lim SMJ, Lee WH, Keane A, Wong JWS, Minney-Smith CA, Martin AC, Mace AO, et al. Examining the entire delayed respiratory syncytial virus season in Western Australia. Arch Dis Child. 2022 May;107(5):517-519. doi: 10.1136/archdischild-2021-323375. Epub 2021 Dec 20. PMID: 34930725.	Duplicate with Foley DA, et al. Arch Dis Child 2022;107:e1. doi:10.1136/archdischild-2021-322507
106	Foley DA, Yeoh DK, Minney-Smith CA, Martin AC, Mace AO, Sikazwe CT, Le H, Levy A, Moore HC, Blyth CC. The Interseasonal Resurgence of Respiratory Syncytial Virus in Australian Children Following the Reduction of Coronavirus Disease 2019-Related Public Health Measures. Clin Infect Dis. 2021 Nov 2;73(9):e2829-e2830. doi: 10.1093/cid/ciaa1906. PMID: 33594407; PMCID: PMC7929151.	Inappropriate study design. Editorial letter.

107	Fourgeaud J, Toubiana J, Chappuy H, Delacourt C, Moulin F, Parize P, Scemla A, Abid H, Leruez-Ville M, Frange P. Impact of public health measures on the post-COVID-19 respiratory syncytial virus epidemics in France. Eur J Clin Microbiol Infect Dis. 2021 Nov;40(11):2389-2395. doi: 10.1007/s10096-021-04323-1. Epub 2021 Aug 4. PMID: 34347190; PMCID: PMC8331994.	Inappropriate population and RSV prevalence not reported.
108	Fourgeaud J, Toubiana J, Chappuy H, Delacourt C, Moulin F, Parize P, Scemla A, Abid H, Leruez-Ville M, Frange P. No durable impact of COVID-19 measures on the hospital burden of respiratory syncytial virus (France, 2018-2022). J Infect. 2022 Oct;85(4):436-480. doi: 10.1016/j.jinf.2022.06.019. Epub 2022 Jun 26. PMID: 35760301; PMCID: PMC9233884.	Incomplete data on RSV prevalence
109	Fukuda Y, Tsugawa T, Nagaoka Y, Ishii A, Nawa T, Togashi A, Kunizaki J, Hirakawa S, Iida J, Tanaka T, Kizawa T, Yamamoto D, Takeuchi R, Sakai Y, Kikuchi M, Nagai K, Asakura H, Tanaka R, Yoshida M, Hamada R, Kawasaki Y. Surveillance in hospitalized children with infectious diseases in Japan: Pre- and post- coronavirus disease 2019. J Infect Chemother. 2021 Nov;27(11):1639-1647. doi: 10.1016/j.jiac.2021.07.024. Epub 2021 Aug 4. PMID: 34389224; PMCID: PMC8332734.	Incomplete data on RSV prevalence
110	Furuse Y, Tamaki R, Okamoto M, Saito-Obata M, Suzuki A, Saito M, Imamura T, Khandaker I, Dapat I, Ueno F, Alday PP, Tan AG, Inobaya MT, Segubre-Mercado E, Tallo V, Lupisan S, Oshitani H. Association Between Preceding Viral Respiratory Infection and Subsequent Respiratory Illnesses Among Children: A Prospective Cohort Study in the Philippines. J Infect Dis. 2019 Jan 7;219(2):197-205. doi: 10.1093/infdis/jiy515. PMID: 30189092; PMCID: PMC6306022.	Inappropriate population and seasonality not reported.
111	Furuse Y, Tamaki R, Suzuki A, Kamigaki T, Okamoto M, Saito-Obata M, Nakagawa E, Saito M, Segubre-Mercado E, Tallo V, Lupisan S, Oshitani H. Epidemiological and clinical characteristics of children with acute respiratory viral infections in the Philippines: a prospective cohort study. Clin Microbiol Infect. 2021 Jul;27(7):1037.e9-1037.e14. doi: 10.1016/j.cmi.2020.09.017. Epub 2020 Sep 17. PMID: 32950713.	Prevalence not reported by RSV season individually.
112	Gantenberg JR, van Aalst R, Zimmerman N, Limone B, Chaves SS, La Via WV, Nelson CB, Rizzo C, Savitz DA, Zullo AR. "Medically Attended Illness due to Respiratory Syncytial Virus Infection Among Infants Born in the United States Between 2016 and 2020." J Infect Dis. 2022 Aug 15;226(Suppl 2):S164-S174. doi: 10.1093/infdis/jiac185. PMID: 35968869; PMCID: PMC9377038.	Inappropriate population and RSV prevalence not reported.
113	García-Corzo JR, Niederbacher-Velásquez J, González-Rugeles C, Rodríguez-Villamizar L, Machuca-Pérez M, Torres-Prieto A, et al. Etiology and seasonality of viral respiratory infections in children under 5 years of age in Bucaramanga, Colombia. Iatreia. 2017;30(2):107-116. DOI: 10.17533/udea.iatreia.v30n2a01.	Period of study before 2015.
114	García-García E, Rodríguez-Pérez M, Melón García S, Fernández Montes R, Suárez Castañón C, Amigo Bello MC, Rodríguez Dehli C, Pérez-Méndez C, Alonso Álvarez MA, Calle-Miguel L. "Change on the Circulation of Respiratory Viruses and Pediatric Healthcare Utilization	Inappropriate population and RSV

	during the COVID-19 Pandemic in Asturias, Northern Spain." Children (Basel). 2022 Sep 24;9(10):1464. doi: 10.3390/children9101464. PMID: 36291400; PMCID: PMC9601124.	prevalence not reported.
115	Ge X, Guo Y, Chen J, Hu R, Feng X. Epidemiology and Seasonality of Respiratory Viruses Detected from Children with Respiratory Tract Infections in Wuxi, East China. Med Sci Monit. 2018 Mar 30;24:1856-1862. doi: 10.12659/msm.908483. PMID: 29599424; PMCID: PMC5892462.	Incomplete data on RSV prevalence
116	Gentile Á, Lucion MF, Juárez MDV, Castellano V, Bakir J, Pacchiotti A, Areso MS, Viegas M, Goya S, Mistchenko A. Respiratory syncytial virus in preterm infants: 19 years of active epidemiological surveillance in a children's hospital. Arch Argent Pediatr. 2020 Dec;118(6):386-392. English, Spanish. doi: 10.5546/aap.2020.eng.386. PMID: 33231045.	Prevalence not reported by RSV season individually.
117	Ghazaly M, Nadel S. Characteristics of children admitted to intensive care with acute bronchiolitis. Eur J Pediatr. 2018 Jun;177(6):913-920. doi: 10.1007/s00431-018-3138-6. Epub 2018 Apr 13. PMID: 29654399; PMCID: PMC5958152.	Prevalence not reported by RSV season individually.
118	Ghazaly MMH, Abu Faddan NH, Raafat DM, Mohammed NA, Nadel S. Acute viral bronchiolitis as a cause of pediatric acute respiratory distress syndrome. Eur J Pediatr. 2021 Apr;180(4):1229-1234. doi: 10.1007/s00431-020-03852-9. Epub 2020 Nov 7. PMID: 33161501; PMCID: PMC7648537.	Prevalence not reported by RSV season individually.
119	Gimferrer L, Vila J, Piñana M, Andrés C, Rodrigo-Pendás JA, Peremiquel- Trillas P, Codina MG, C Martín MD, Esperalba J, Fuentes F, Rubio S, Campins- Martí M, Pumarola T, Antón A. Virological surveillance of human respiratory syncytial virus A and B at a tertiary hospital in Catalonia (Spain) during five consecutive seasons (2013-2018). Future Microbiol. 2019 Mar;14:373-381. doi: 10.2217/fmb-2018-0261. Epub 2019 Mar 12. PMID: 30860397.	Inappropriate population.
120	Giraud-Gatineau A, Kaba L, Boschi C, Devaux C, Casalta JP, Gautret P, Chaudet H, Colson P, Raoult D. Control of common viral epidemics but not of SARS-CoV-2 through the application of hygiene and distancing measures. J Clin Virol. 2022 Jun;150-151:105163. doi: 10.1016/j.jcv.2022.105163. Epub 2022 Apr 16. PMID: 35472752; PMCID: PMC9013017.	Incomplete data on RSV prevalence
121	Glatman-Freedman A, Kaufman Z, Applbaum Y, Dichtiar R, Steiman A, Gordon ES, Keinan-Boker L, Shohat T, Haklai Z. Respiratory Syncytial Virus hospitalization burden: a nation-wide population-based analysis, 2000-2017. J Infect. 2020 Aug;81(2):297-303. doi: 10.1016/j.jinf.2020.05.078. Epub 2020 Jun 3. PMID: 32504738.	Prevalence not reported by RSV season individually.
122	Gokce S, Kurugol Z, Cerit Z, Cicek C. The Effect of Respiratory Syncytial Virus on the Severity of Acute Bronchiolitis in Hospitalized Infants: A Prospective Study from Turkey. Iranian Journal of Pediatrics. 2018;28(2):e61034. DOI: 10.5812/ijp.61034. Published: Apr 2018.	Incomplete data on RSV prevalence

123	Goldstein E, Finelli L, O'Halloran A, Liu P, Karaca Z, Steiner CA, Viboud C, Lipsitch M. Hospitalizations Associated with Respiratory Syncytial Virus and Influenza in Children, Including Children Diagnosed with Asthma. Epidemiology. 2019 Nov;30(6):918-926. doi: 10.1097/EDE.0000000000001092. PMID: 31469696; PMCID: PMC6768705.	Period of study before 2015.
124	Gong L, Wu C, Lu M, Huang C, Chen Y, Li Z, Huang G, Liu D, Tang X. Analysis of Incidence and Clinical Characteristics of RSV Infection in Hospitalized Children: A Retrospective Study. Risk Manag Healthc Policy. 2021 Apr 14;14:1525-1531. doi: 10.2147/RMHP.S305370. PMID: 33889036; PMCID: PMC8054821.	Incomplete data on RSV prevalence
125	Grilc E, Prosenc Trilar K, Lajovic J, Sočan M. Determining the seasonality of respiratory syncytial virus in Slovenia. Influenza Other Respir Viruses. 2021 Jan;15(1):56-63. doi: 10.1111/irv.12779. Epub 2020 Jul 12. PMID: 32656961; PMCID: PMC7767947.	Inappropriate population.
126	Groves HE, Piché-Renaud PP, Peci A, Farrar DS, Buckrell S, Bancej C, Sevenhuysen C, Campigotto A, Gubbay JB, Morris SK. The impact of the COVID-19 pandemic on influenza, respiratory syncytial virus, and other seasonal respiratory virus circulation in Canada: A population-based study. Lancet Reg Health Am. 2021 Sep;1:100015. doi: 10.1016/j.lana.2021.100015. Epub 2021 Jul 17. PMID: 34386788; PMCID: PMC8285668.	Inappropriate population and RSV prevalence not reported.
127	Guan X, Gao S, Zhao H, Zhou H, Yang Y, Yu S, Wang J. Clinical characteristics of hospitalized term and preterm infants with community-acquired viral pneumonia. BMC Pediatr. 2022 Jul 27;22(1):452. doi: 10.1186/s12887-022-03508-7. PMID: 35897053; PMCID: PMC9325944.	Seasonality not reported.
128	Guiomar R, Cristovao P, Conde P, Pechirra P. Molecular epidemiology of respiratory syncytial virus between 2010-2015 in Portugal. J Clin Virol. 2015 Sep;70(Supplement 1):S49-S49. doi: 10.1016/j.jcv.2015.07.117.	Inappropriate study design. Abstract for Congress.
129	Guitart C, Alejandre C, Torrús I, Balaguer M, Esteban E, Cambra FJ, Jordan I. Impact of a modification of the clinical practice guide of the American Academy of Pediatrics in the management of severe acute bronchiolitis in a pediatric intensive care unit. Med Intensiva (Engl Ed). 2021 Jun- Jul;45(5):289-297. English, Spanish. doi: 10.1016/j.medin.2019.10.006. Epub 2019 Dec 28. PMID: 31892419; PMCID: PMC7115415.	Prevalence not reported by RSV season individually.
130	Haapanen M, Renko M, Artama M, Kuitunen I. The impact of the lockdown and the re-opening of schools and day cares on the epidemiology of SARS-CoV-2 and other respiratory infections in children - A nationwide register study in Finland. EClinicalMedicine. 2021 Apr;34:100807. doi: 10.1016/j.eclinm.2021.100807. Epub 2021 Mar 29. PMID: 33817612; PMCID: PMC8007090.	Inappropriate population and RSV prevalence not reported.

131	Haddadin Z, Beveridge S, Fernandez K, Rankin DA, Probst V, Spieker AJ, Markus TM, Stewart LS, Schaffner W, Lindegren ML, Halasa N. Respiratory Syncytial Virus Disease Severity in Young Children. Clin Infect Dis. 2021 Dec 6;73(11):e4384-e4391. doi: 10.1093/cid/ciaa1612. PMID: 33095882; PMCID: PMC8826377.	Duplicate with Rha et al. Pediatrics. 2020;146(1):e20193611. doi:10.1542/peds.2019-3611
132	Haddadin Z, Schuster JE, Spieker AJ, Rahman H, Blozinski A, Stewart L, Campbell AP, Lively JY, Michaels MG, Williams JV, Boom JA, Sahni LC, Staat M, McNeal M, Selvarangan R, Harrison CJ, Weinberg GA, Szilagyi PG, Englund JA, Klein EJ, Curns AT, Rha B, Langley GE, Hall AJ, Patel MM, Halasa NB. Acute Respiratory Illnesses in Children in the SARS-CoV-2 Pandemic: Prospective Multicenter Study. Pediatrics. 2021 Aug;148(2):e2021051462. doi: 10.1542/peds.2021-051462. Epub 2021 May 13. PMID: 33986150; PMCID: PMC8338906.	Incomplete data on RSV prevalence
133	Haddadin Z, Spieker AJ, Rahman H, Rankin DA, Talj R, Yanis A, Amarin JZ, Schmitz J, Chappell J, Halasa NB. Respiratory pathogens during the COVID-19 pandemic: Alterations in detection and seasonality in Nashville, Tennessee. PLoS One. 2022 Aug 3;17(8):e0270469. doi: 10.1371/journal.pone.0270469. PMID: 35921608; PMCID: PMC9348857.	Inappropriate population.
134	Halabi KC, Saiman L, Zachariah P. The Epidemiology of Respiratory Syncytial Virus in New York City during the Coronavirus Disease-2019 Pandemic Compared with Previous Years. J Pediatr. 2022 Mar;242:242-244.e1. doi: 10.1016/j.jpeds.2021.10.057. Epub 2021 Oct 30. PMID: 34728233; PMCID: PMC8556683.	Incomplete data on RSV prevalence
135	Hallmann-Szelińska E, Bednarska K, Kondratiuk K, Rabczenko D, Brydak LB. Viral Infections in Children in the 2014/2015 Epidemic Season in Poland. Adv Exp Med Biol. 2016;912:51-6. doi: 10.1007/5584_2016_209. PMID: 26987329; PMCID: PMC7119986.	Period of study before 2015.
136	Hamamoto I, Shimasaki N. The importance of monitoring viral respiratory infections during the COVID-19 crisis. J Disaster Res. 2022 Jan;17(1):73-81. doi: 10.20965/jdr.2022.p0073.	Inappropriate study design.
137	Hatem AM, Abuelhassan UE, Mohamed SAA, Rizk MS, El-kholy A, Al-Harras M. Viral and atypical bacterial etiologies of severe acute respiratory infection (SARI) in Egyptian patients: epidemiological patterns and results from the sentinel surveillance study 2010-2014. EGYPTIAN JOURNAL OF CHEST DISEASES AND TUBERCULOSIS. 2019;68(1):88-95. DOI: 10.4103/ejcdt.ejcdt_96_18. Published: JAN-MAR 2019. Accession Number: WOS:000461542100016	Period of study before 2015.
138	Heimdal I, Valand J, Krokstad S, Moe N, Christensen A, Risnes K, Nordbø SA, Døllner H. Hospitalized Children With Common Human Coronavirus Clinical Impact of Codetected Respiratory Syncytial Virus and Rhinovirus. Pediatr Infect Dis J. 2022 Mar 1;41(3):e95-e101. doi: 10.1097/INF.0000000000003433. PMID: 35001055; PMCID: PMC8826606.	Inappropriate population and RSV

		prevalence not reported.
139	Heppe-Montero M, Walter S, Hernández-Barrera V, Gil-Prieto R, Gil-de-Miguel Á. Burden of respiratory syncytial virus-associated lower respiratory infections in children in Spain from 2012 to 2018. BMC Infect Dis. 2022 Mar 31;22(1):315. doi: 10.1186/s12879-022-07261-1. PMID: 35361139; PMCID: PMC8969337.	Incomplete data on RSV prevalence
140	Hernández-Rivas L, Pedraz T, Calvo C, San Juan I, Mellado MªJ, Robustillo A. Respiratory syncytial virus outbreak during the COVID-19 pandemic. How has it changed? Enferm Infecc Microbiol Clin. 2023 Jun-Jul;41(6):352-355. doi: 10.1016/j.eimc.2021.12.003. Epub 2021 Dec 22. PMID: 34955580; PMCID: PMC8692060.	Incomplete data on RSV prevalence
141	Hindupur A, Menon T, Dhandapani P. Epidemiology of respiratory syncytial virus infections in Chennai, south India. Clinical Epidemiology and Global Health. 2019;7(3):288-292. DOI: 10.1016/j.cegh.2018.10.004.	Inappropriate population.
142	Hodjat P, Christensen PA, Subedi S, Bernard DW, Olsen RJ, Long SW. The Reemergence of Seasonal Respiratory Viruses in Houston, Texas, after Relaxing COVID-19 Restrictions. Microbiol Spectr. 2021 Oct 31;9(2):e0043021. doi: 10.1128/Spectrum.00430-21. Epub 2021 Sep 8. PMID: 34494861; PMCID: PMC8557899.	Inappropriate population and RSV prevalence not reported.
143	Hsu HT, Huang FL, Ting PJ, Chang CC, Chen PY. The epidemiological features of pediatric viral respiratory infection during the COVID-19 pandemic in Taiwan. J Microbiol Immunol Infect. 2022 Dec;55(6 Pt 1):1101-1107. doi: 10.1016/j.jmii.2021.09.017. Epub 2021 Oct 9. PMID: 34756671; PMCID: PMC8501510.	Inappropriate population.
144	Huang XB, Yuan L, Ye CX, Zhu X, Lin CJ, Zhang DM, He KS, Niu RX, Cao KY, Xu L. Epidemiological characteristics of respiratory viruses in patients with acute respiratory infections during 2009-2018 in southern China. Int J Infect Dis. 2020 Sep;98:21-32. doi: 10.1016/j.ijid.2020.06.051. Epub 2020 Jun 17. PMID: 32562851.	Prevalence not reported by RSV season individually.
145	Hughes B, Duong D, White BJ, Wigginton KR, Chan EMG, Wolfe MK, Boehm AB. Respiratory Syncytial Virus (RSV) RNA in Wastewater Settled Solids Reflects RSV Clinical Positivity Rates. Environmental Science & Technology Letters. 2022;9(2):173-178. DOI: 10.1021/acs.estlett.1c00963. Published: Jan 12, 2022.	Inappropriate population.
146	Hussain F, Delgado Thompson M, Vick D, West J, Edwards M. Clinical severity of RSV bronchiolitis. Health Sci Rep. 2022 Mar 22;5(2):e543. doi: 10.1002/hsr2.543. PMID: 35356804; PMCID: PMC8939497.	Prevalence not reported by RSV season individually.

147	Ihling CM, Schnitzler P, Heinrich N, Mangu C, Sudi L, Souares A, Gies S, Sié A, Coulibaly B, Ouédraogo AT, Mordmüller B, Held J, Adegnika AA, Fernandes JF, Eckerle I, May J, Hogan B, Eibach D, Tabatabai J. Molecular epidemiology of respiratory syncytial virus in children in sub-Saharan Africa. Trop Med Int Health. 2021 Jul;26(7):810-822. doi: 10.1111/tmi.13573. Epub 2021 Mar 28. PMID: 33683751.	Inappropriate population and seasonality not reported.
148	Irfan A, Lankachandra K. The Changing Epidemiology of the Respiratory Syncytial Virus in Light of the COVID-19 Pandemic. American Journal of Clinical Pathology. 2022;158(Supp 1, Special Issue: SI):S132-S133. Published: Nov 9, 2022. Conference presentation at ASCP and ACLPS Annual Meeting, held on Sep 7-9, 2022, via ELECTR NETWORK. ISSN: 0002-9173, eISSN: 1943-7722.	Inappropriate population.
149	Jain S. Epidemiology of Viral Pneumonia. Clin Chest Med. 2017 Mar;38(1):1-9. doi: 10.1016/j.ccm.2016.11.012. Epub 2016 Dec 22. PMID: 28159152; PMCID: PMC7115731.	Inappropriate study design. Review.
150	Jamieson N, Akande M, Karsies T, Smith RM, Kline D, Spencer SP. Respiratory Pathogen Detection in Pediatric Patients Intubated for Presumed Infection. Pediatr Emerg Care. 2022 Jan 1;38(1):e398-e403. doi: 10.1097/PEC.0000000000002301. PMID: 33201137.	Prevalence not reported by RSV season individually.
151	Jeena PM. The epidemiology and risk factors of respiratory syncytial virus and its impact on the timing of immunoprophylaxis. Afr J Thorac Crit Care Med. 2018;24(1):10.7196/SARJ.2018.v24i1.202. doi: 10.7196/SARJ.2018.v24i1.202.	Inappropriate study design. Editorial letter.
152	Jensen A, A F Simões E, Bohn Christiansen C, Graff Stensballe L. Respiratory syncytial virus and influenza hospitalizations in Danish children 2010-2016. Vaccine. 2021 Jul 5;39(30):4126-4134. doi: 10.1016/j.vaccine.2021.05.097. Epub 2021 Jun 9. PMID: 34116876.	Prevalence not reported by RSV season individually.
153	Jeon JH, Han M, Chang HE, Park SS, Lee JW, Ahn YJ, Hong DJ. Incidence and seasonality of respiratory viruses causing acute respiratory infections in the Northern United Arab Emirates. J Med Virol. 2019 Aug;91(8):1378-1384. doi: 10.1002/jmv.25464. Epub 2019 Apr 7. PMID: 30900750; PMCID: PMC7166826.	Prevalence not reported by RSV season individually.
154	Jerbi A, Fodha I, Ben Hamida-Rebai M, Ben Hadj Fredj M, Ataoui I, Bennour H, Abroug S, Khlifa M, Mathlouthi J, Mahdhaoui N, Boussetta K, Trabelsi A. Molecular characterization of respiratory syncytial virus circulating in Tunisia between 2015 and 2018. J Med Microbiol. 2020 Sep;69(9):1203-1212. doi: 10.1099/jmm.0.001240. Epub 2020 Aug 5. PMID: 32755531.	Inappropriate population.
155	Jerbi A, Fodha I, Ben Hmida M, Bennour H, Ataoui I, Fredj MB, Trabelsi A. Molecular epidemiology of respiratory syncytial virus in hospitalized children in Tunisia. European Respiratory Journal. 2019;54(Supplement 63):PA1051. DOI: 10.1183/13993003.congress-	Prevalence not reported by RSV season individually.

	2019.PA1051. Published: Sep 28, 2019. Conference presentation at the International Congress of the European-Respiratory-Society (ERS), held on Sep 28-Oct 02, 2019, in Madrid, Spain. ISSN: 0903-1936, eISSN: 1399-3003.	
156	Jia R, Lu L, Su L, Lin Z, Gao D, Lv H, Xu M, Liu P, Cao L, Xu J. Resurgence of Respiratory Syncytial Virus Infection During COVID-19 Pandemic Among Children in Shanghai, China. Front Microbiol. 2022 Jul 1;13:938372. doi: 10.3389/fmicb.2022.938372. PMID: 35875547; PMCID: PMC9298468.	Incomplete data on RSV prevalence
157	Jiang S, Liu P, Xiong G, Yang Z, Wang M, Li Y, Yu XJ. Coinfection of SARS-CoV-2 and multiple respiratory pathogens in children. Clin Chem Lab Med. 2020 Jun 25;58(7):1160-1161. doi: 10.1515/cclm-2020-0434. PMID: 32301747.	Inappropriate study design. Case report
158	Kahanowitch R, Gaviria S, Aguilar H, Gayoso G, Chorvinksy E, Bera B, Rodríguez-Martínez CE, Gutierrez MJ, Nino G. How did respiratory syncytial virus and other pediatric respiratory viruses change during the COVID-19 pandemic? Pediatr Pulmonol. 2022 Oct;57(10):2542-2545. doi: 10.1002/ppul.26053. Epub 2022 Aug 26. PMID: 35774020; PMCID: PMC9349531.	Inappropriate population and RSV prevalence not reported.
159	Kamata K, Thein KN, Di Ja L, Win NC, Win SMK, Suzuki Y, Ito A, Osada H, Chon I, Phyu WW, Aizawa Y, Ikuse T, Ota T, Kyaw Y, Tin HH, Shobugawa Y, Watanabe H, Saito R, Saitoh A. Clinical manifestations and outcome of viral acute lower respiratory infection in hospitalized children in Myanmar. BMC Infect Dis. 2022 Apr 8;22(1):350. doi: 10.1186/s12879-022-07342-1. PMID: 35395744; PMCID: PMC8992414.	Prevalence not reported by RSV season individually.
160	Kang HM, Park KC, Park J, Kil HR, Yang EA. Circulating Respiratory Syncytial Virus Genotypes and Genetic Variability of the G Gene during 2017 and 2018/2019 Seasonal Epidemics Isolated from Children with Lower Respiratory Tract Infections in Daejeon, Korea. J Korean Med Sci. 2020 Dec 21;35(49):e422. doi: 10.3346/jkms.2020.35.e422. PMID: 33350185; PMCID: PMC7752254.	Incomplete data on RSV prevalence
161	Kanık A, Eliaçık K, Koyun B, İnce OT, Derici YK, Yılmaz NÖ, Çiftdoğan DY. Viral etiology of acute bronchiolitis in hospitalized infants and the effect on clinical course. Cocuk Enfeksiyon Derg [Internet]. 2016;10(3):93-8. Available from: www.scopus.com	Language not in English.
162	Kasap T, Takci S, Ozcan P. Evaluation of Babies with Viral Lower Respiratory Tract Infections in Neonatal Intensive Care Unit. Guncel Pediatri - Journal of Current Pediatrics. 2021;19(1):106-112. DOI: 10.4274/jcp.2021.0015. Published: Apr 2021.	Language not in English.
163	Kasuya F, Mori K, Harada S, Kumagai R, Suzuki A, Amano A, Kosugi T, Hasegawa M, Nagashima M, Suzuki J, Sadamasu K. "Molecular and Epidemiological Analysis of Respiratory Syncytial Virus Detected in Tokyo, Japan in 2021 Season." Jpn J Infect Dis. 2023 Jan 24;76(1):87-90. doi: 10.7883/yoken.JJID.2022.035. Epub 2022 Sep 30. PMID: 36184395.	Inappropriate population.
164	Khomenko VE, Iemets OV, Volosovets OP, Kryvopustov SP, Kryvopustova MV, Mozyrska OV. EPIDEMIOLOGY OF RESPIRATORY PATHOGENS IN CHILDREN WITH ACUTE RESPIRATORY TRACT INFECTION IN UKRAINE DURING 2018-2020 YEARS. Wiad Lek. 2021;74(6):1389-1395. PMID: 34159925.	Prevalence not reported by RSV season individually.

165	Kim JH, Kim HY, Lee M, Ahn JG, Baek JY, Kim MY, Huh K, Jung J, Kang JM. "Respiratory Syncytial Virus Outbreak Without Influenza in the Second Year of the Coronavirus Disease 2019 Pandemic: A National Sentinel Surveillance in Korea, 2021-2022 Season." J Korean Med Sci. 2022 Aug 29;37(34):e258. doi: 10.3346/jkms.2022.37.e258. PMID: 36038956; PMCID: PMC9424700.	Incomplete data on RSV prevalence
166	Kim JH, Roh YH, Ahn JG, Kim MY, Huh K, Jung J, Kang JM. Respiratory syncytial virus and influenza epidemics disappearance in Korea during the 2020-2021 season of COVID-19. Int J Infect Dis. 2021 Sep;110:29-35. doi: 10.1016/j.ijid.2021.07.005. Epub 2021 Jul 7. PMID: 34245886.	Inappropriate population and RSV prevalence not reported.
167	Kim YK, Song SH, Ahn B, Lee JK, Choi JH, Choi SH, Yun KW, Choi EH. Shift in Clinical Epidemiology of Human Parainfluenza Virus Type 3 and Respiratory Syncytial Virus B Infections in Korean Children Before and During the COVID-19 Pandemic: A Multicenter Retrospective Study. J Korean Med Sci. 2022 Jul 18;37(28):e215. doi: 10.3346/jkms.2022.37.e215. PMID: 35851860; PMCID: PMC9294504.	Incomplete data on RSV prevalence
168	Kiseleva IV, Larionova NV, Grigorieva EP, Ksenafontov AD, Al Farroukh M, Rudenko LG. Salient features of circulating respiratory viruses in the pre- and pandemic influenza and COVID-19 seasons. INFEKTSIYA I IMMUNITET. 2021;11(6):1009-1019. DOI: 10.15789/2220-7619-SFO-1662. Published: NOV-DEC 2021.	Language not in English.
169	Kobayashi Y, Togo K, Agosti Y, McLaughlin JM. Epidemiology of respiratory syncytial virus in Japan: A nationwide claims database analysis. Pediatr Int. 2022 Jan;64(1):e14957. doi: 10.1111/ped.14957. Epub 2021 Dec 17. PMID: 34388302; PMCID: PMC9300113.	Incomplete data on RSV prevalence
170	Kohns Vasconcelos M, Loens K, Sigfrid L, Iosifidis E, Epalza C, Donà D, Matheeussen V, Papachristou S, Roilides E, Gijon M, Rojo P, Minotti C, Da Dalt L, Islam S, Jarvis J, Syggelou A, Tsolia M, Nyirenda Nyang'wa M, Keers S, Renk H, Gemmel AL, D'Amore C, Ciofi Degli Atti M, Rodríguez-Tenreiro Sánchez C, Martinón-Torres F, Burokienė S, Goetghebuer T, Spoulou V, Riordan A, Calvo C, Gkentzi D, Hufnagel M, Openshaw PJ, de Jong MD, Koopmans M, Goossens H, Ieven M, Fraaij PLA, Giaquinto C, Bielicki JA, Horby P, Sharland M. Aetiology of acute respiratory infection in preschool children requiring hospitalisation in Europe- results from the PED-MERMAIDS multicentre case-control study. BMJ Open Respir Res. 2021 Jul;8(1):e000887. doi: 10.1136/bmjresp-2021-000887. PMID: 34326154; PMCID: PMC8323363.	Prevalence not reported by RSV season individually.
171	Kolawole O, Oguntoye M, Dam T, Chunara R. Etiology of respiratory tract infections in the community and clinic in Ilorin, Nigeria. BMC Res Notes. 2017 Dec 7;10(1):712. doi: 10.1186/s13104-017-3063-1. PMID: 29212531; PMCID: PMC5719735.	Seasonality not reported.
172	Korsun N, Angelova S, Trifonova I, Georgieva I, Voleva S, Tzotcheva I, Mileva S, Ivanov I, Tcherveniakova T, Perenovska P. Viral pathogens associated with acute lower respiratory tract infections in children younger than 5 years of age in Bulgaria. Braz J Microbiol. 2019 Jan;50(1):117-125. doi: 10.1007/s42770-018-0033-2. Epub 2018 Dec 5. PMID: 30637646; PMCID: PMC6863252.	Prevalence not reported by RSV season individually.

173	Korsun N, Angelova S, Tzotcheva I, Georgieva I, Lazova S, Parina S, Alexiev I, Perenovska P. Prevalence and genetic characterisation of respiratory syncytial viruses circulating in Bulgaria during the 2014/15 and 2015/16 winter seasons. Pathog Glob Health. 2017 Oct;111(7):351-361. doi: 10.1080/20477724.2017.1375708. Epub 2017 Sep 26. PMID: 28948867; PMCID: PMC5694888.	Prevalence not reported by RSV season individually.
174	Kuczborska K, Rustecka A, Wawrzyniak A, Bedzichowska A, Kalicki B. Manifestations and Risk Factors in Children Hospitalized with Respiratory Syncytial Virus Infection. Archives of Pediatric Infectious Diseases. 2021;9(2):e108723. DOI: 10.5812/pedinfect.108723. Published: Apr 2021.	Prevalence not reported by RSV season individually.
175	Kuitunen I, Artama M, Haapanen M, Renko M. Respiratory virus circulation in children after relaxation of COVID-19 restrictions in fall 2021-A nationwide register study in Finland. J Med Virol. 2022 Sep;94(9):4528-4532. doi: 10.1002/jmv.27857. Epub 2022 May 23. PMID: 35577532; PMCID: PMC9347728.	Inappropriate population.
176	Kuitunen I, Artama M, Mäkelä L, Backman K, Heiskanen-Kosma T, Renko M. Effect of Social Distancing Due to the COVID-19 Pandemic on the Incidence of Viral Respiratory Tract Infections in Children in Finland During Early 2020. Pediatr Infect Dis J. 2020 Dec;39(12):e423-e427. doi: 10.1097/INF.0000000000002845. PMID: 32773660.	Seasonality not reported.
177	Kumar P, Medigeshi GR, Mishra VS, Islam M, Randev S, Mukherjee A, Chaudhry R, Kapil A, Ram Jat K, Lodha R, Kabra SK. Etiology of Acute Respiratory Infections in Infants: A Prospective Birth Cohort Study. Pediatr Infect Dis J. 2017 Jan;36(1):25-30. doi: 10.1097/INF.0000000000001359. PMID: 27753796.	Period of study before 2015.
178	Kume Y, Hashimoto K, Shirato K, Norito S, Suwa R, Chishiki M, Ono T, Mashiyama F, Mochizuki I, Sato M, Ishibashi N, Suzuki S, Sakuma H, Takahashi H, Takeda M, Hosoya M. Epidemiological and clinical characteristics of infections with seasonal human coronavirus and respiratory syncytial virus in hospitalized children immediately before the coronavirus disease 2019 pandemic. J Infect Chemother. 2022 Jul;28(7):859-865. doi: 10.1016/j.jiac.2022.03.001. Epub 2022 Mar 15. PMID: 35307263; PMCID: PMC8920880.	Prevalence not reported by RSV season individually.
179	Kurskaya O, Ryabichenko T, Leonova N, Shi W, Bi H, Sharshov K, Kazachkova E, Sobolev I, Prokopyeva E, Kartseva T, Alekseev A, Shestopalov A. Viral etiology of acute respiratory infections in hospitalized children in Novosibirsk City, Russia (2013 - 2017). PLoS One. 2018 Sep 18;13(9):e0200117. doi: 10.1371/journal.pone.0200117. PMID: 30226876; PMCID: PMC6143185.	Prevalence not reported by RSV season individually.
180	Kwon Y, Cho WJ, Kim HM, Lee J. Single or dual infection with respiratory syncytial virus and human rhinovirus: Epidemiology and clinical characteristics in hospitalized children in a rural area of South Korea. Pediatr Infect Vaccine. 2019;26(2):99-111. Available from: www.scopus.com	Language not in English.
181	Lagacé-Wiens P, Bullard J, Cole R, Van Caeseele P. Seasonality of coronaviruses and other respiratory viruses in Canada: Implications for COVID-19. Can Commun Dis Rep. 2021 Mar 31;47(3):132-138. doi: 10.14745/ccdr.v47i03a02. PMID: 34012336; PMCID: PMC8109286.	Inappropriate population and RSV

		prevalence not reported.
182	Lai SY, Liu YL, Jiang YM, Liu T. "Precautions against COVID-19 reduce respiratory virus infections among children in Southwest China." Medicine (Baltimore). 2022 Sep 16;101(37):e30604. doi: 10.1097/MD.0000000000030604. PMID: 36123935; PMCID: PMC9477712.	Inappropriate population.
183	Langley JM, Bianco V, Domachowske JB, Madhi SA, Stoszek SK, Zaman K, Bueso A, Ceballos A, Cousin L, D'Andrea U, Dieussaert I, Englund JA, Gandhi S, Gruselle O, Haars G, Jose L, Klein NP, Leach A, Maleux K, Nguyen TLA, Puthanakit T, Silas P, Tangsathapornpong A, Teeratakulpisarn J, Vesikari T, Cohen RA. Incidence of Respiratory Syncytial Virus Lower Respiratory Tract Infections During the First 2 Years of Life: A Prospective Study Across Diverse Global Settings. J Infect Dis. 2022 Aug 26;226(3):374-385. doi: 10.1093/infdis/jiac227. PMID: 35668702; PMCID: PMC9417131.	Inappropriate population and seasonality not reported.
184	Lee S-J, Lee S-H, Ha E-K, Sheen Y-H, Sung M-S, Jung Y-H, Lee K-S, Jee H-M, Han M-Y. Prevalence of respiratory virus infection with regard to age, sex, and seasonality factors: A single-center experience against children hospitalized during the 10 years. Allergy Asthma Respir Dis. 2017;5(6):320-325. https://doi.org/10.4168/aard.2017.5.6.320	Language not in English.
185	Lei C, Yang L, Lou CT, Yang F, SiTou KI, Hu H, Io K, Cheok KT, Pan B, Ung COL. Viral etiology and epidemiology of pediatric patients hospitalized for acute respiratory tract infections in Macao: a retrospective study from 2014 to 2017. BMC Infect Dis. 2021 Mar 26;21(1):306. doi: 10.1186/s12879-021-05996-x. PMID: 33771128; PMCID: PMC7995389.	Prevalence not reported by RSV season individually.
186	Leone MB, Ponti DA, Fernández Berengeno MN, Grisolía NA, Aprea VP, Yazde de Puleio ML, Svartz A, Haleblian E. Screening for common respiratory viruses in pediatric outpatients 2 years after the onset of the COVID-19 pandemic. Arch Argent Pediatr. 2022 Aug;120(4):264-268. English, Spanish. doi: 10.5546/aap.2022.eng.264. Epub 2022 May 4. PMID: 35900953.	Inappropriate population.
187	Leuzinger K, Roloff T, Gosert R, Sogaard K, Naegele K, Rentsch K, Bingisser R, Nickel CH, Pargger H, Bassetti S, Bielicki J, Khanna N, Tschudin Sutter S, Widmer A, Hinic V, Battegay M, Egli A, Hirsch HH. Epidemiology of Severe Acute Respiratory Syndrome Coronavirus 2 Emergence Amidst Community-Acquired Respiratory Viruses. J Infect Dis. 2020 Sep 14;222(8):1270-1279. doi: 10.1093/infdis/jiaa464. Erratum in: J Infect Dis. 2021 Feb 24;223(4):734-735. PMID: 32726441.	Inappropriate population and seasonality not reported.
188	Li L, Wang H, Liu A, Wang R, Zhi T, Zheng Y, Bao Y, Chen Y, Wang W. Comparison of 11 respiratory pathogens among hospitalized children before and during the COVID-19 epidemic in Shenzhen, China. Virol J. 2021 Oct 9;18(1):202. doi: 10.1186/s12985-021-01669-y. PMID: 34627307; PMCID: PMC8501916.	Seasonality not reported.

189	Li Y, Wang J, Wang C, Yang Q, Xu Y, Xu J, Li Y, Yu X, Zhu H, Liu J. Characteristics of respiratory virus infection during the outbreak of 2019 novel coronavirus in Beijing. Int J Infect Dis. 2020 Jul;96:266-269. doi: 10.1016/j.ijid.2020.05.008. Epub 2020 May 7. PMID: 32389850; PMCID: PMC7204690.	Seasonality not reported.
190	Li Y, Wang X, Cong B, Deng S, Feikin DR, Nair H. Understanding the Potential Drivers for Respiratory Syncytial Virus Rebound During the Coronavirus Disease 2019 Pandemic. J Infect Dis. 2022 Mar 15;225(6):957-964. doi: 10.1093/infdis/jiab606. PMID: 35030633; PMCID: PMC8807230.	Inappropriate population and RSV prevalence not reported.
191	Li YT, Liang Y, Ling YS, Duan MQ, Pan L, Chen ZG. The spectrum of viral pathogens in children with severe acute lower respiratory tract infection: A 3-year prospective study in the pediatric intensive care unit. J Med Virol. 2019 Sep;91(9):1633-1642. doi: 10.1002/jmv.25502. Epub 2019 Jun 13. PMID: 31081548; PMCID: PMC7167151.	Prevalence not reported by RSV season individually.
192	Li ZJ, Yu LJ, Zhang HY, Shan CX, Lu QB, Zhang XA, Ren X, Zhang CH, Wang YF, Lin SH, Xu Q, Jiang BG, Jiang T, Lv CL, Chen JJ, Gao GF, Yang WZ, Wang LP, Yang Y, Fang LQ, Liu W; Chinese Centers for Disease Control and Prevention (CDC) Etiology Surveillance Study Team of Acute Respiratory Infections. Broad Impacts of Coronavirus Disease 2019 (COVID-19) Pandemic on Acute Respiratory Infections in China: An Observational Study. Clin Infect Dis. 2022 Aug 24;75(1):e1054-e1062. doi: 10.1093/cid/ciab942. PMID: 34788811; PMCID: PMC8767888.	Inappropriate population.
193	Liang J, Wang Z, Liu Y, Zeng L, Li Z, Liang J, Liang H, Jiang M, Yang Z. Epidemiology and co-infection patterns in patients with respiratory tract infections in southern China between 2018 and 2020. J Infect. 2021 Sep;83(3):e6-e8. doi: 10.1016/j.jinf.2021.07.013. Epub 2021 Jul 21. PMID: 34302865.	Inappropriate population.
194	Lin CX, Lian HB, Lin GY, Zhang DG, Cai XY, Cai ZW, Wen FQ. Pathogen spectrum changes of respiratory tract infections in children in Chaoshan area under the influence of COVID-19. Epidemiol Infect. 2021;149:e170. doi: 10.1017/S0950268821001606.	Seasonality not reported.
195	Lin TY, Chi H, Kuo CY, Tsai HP, Wang JR, Liu CC, Shen CF. "Outbreak of respiratory syncytial virus subtype ON1 among children during COVID-19 pandemic in Southern Taiwan." J Microbiol Immunol Infect. 2022 Dec;55(6 Pt 2):1168-1179. doi: 10.1016/j.jmii.2022.08.015. Epub 2022 Sep 13. PMID: 36137926.	Incomplete data on RSV prevalence
196	Linssen RS, Bem RA, Kapitein B, Rengerink KO, Otten MH, den Hollander B, Bont L, van Woensel JBM; PICE Study Group. Burden of respiratory syncytial virus bronchiolitis on the Dutch pediatric intensive care units. Eur J Pediatr. 2021 Oct;180(10):3141-3149. doi: 10.1007/s00431-021-04079-y. Epub 2021 Apr 23. PMID: 33891158; PMCID: PMC8429147.	Inappropriate population and RSV prevalence not reported.

197	Linssen RS, Teirlinck AC, van Boven M, Biarent D, Stona L, Amigoni A, Comoretto RI, Leteurtre S, Bruandet A, Bentsen GK, Drage IM, Wang X, Campbell H, van Woensel JBM, Bont L, Bem RA. Increasing burden of viral bronchiolitis in the pediatric intensive care unit; an observational study. J Crit Care. 2022 Apr;68:165-168. doi: 10.1016/j.jcrc.2021.07.009. Epub 2021 Jul 23. PMID: 34304966.	Prevalence not reported by RSV season individually.
198	Liu W, Chen D, Tan W, Xu D, Qiu S, Zeng Z, Li X, Zhou R. Epidemiology and Clinical Presentations of Respiratory Syncytial Virus Subgroups A and B Detected with Multiplex Real-Time PCR. PLoS One. 2016 Oct 20;11(10):e0165108. doi: 10.1371/journal.pone.0165108. PMID: 27764220; PMCID: PMC5072546.	Prevalence not reported by RSV season individually.
199	Liu WK, Chen DH, Tan WP, Qiu SY, Xu D, Zhang L, Gu SJ, Zhou R, Liu Q. Paramyxoviruses respiratory syncytial virus, parainfluenza virus, and human metapneumovirus infection in pediatric hospitalized patients and climate correlation in a subtropical region of southern China: a 7-year survey. Eur J Clin Microbiol Infect Dis. 2019 Dec;38(12):2355-2364. doi: 10.1007/s10096-019-03693-x. Epub 2019 Sep 5. PMID: 31489496; PMCID: PMC6858468.	Prevalence not reported by RSV season individually.
200	Loconsole D, Centrone F, Rizzo C, Caselli D, Orlandi A, Cardinale F, Serio C, Giordano P, Lassandro G, Milella L, Ficarella MT, Baldassarre ME, Laforgia N, Chironna M. Out-of-Season Epidemic of Respiratory Syncytial Virus during the COVID-19 Pandemic: The High Burden of Child Hospitalization in an Academic Hospital in Southern Italy in 2021. Children (Basel). 2022 Jun 8;9(6):848. doi: 10.3390/children9060848. PMID: 35740785; PMCID: PMC9221938.	Incomplete data on RSV prevalence
201	Loevinsohn G, Hamahuwa M, Hardick J, Sinywimaanzi P, Fenstermacher KZJ, Munachoonga P, Weynand A, Monze M, Manabe YC, Gaydos CA, Rothman RE, Pekosz A, Thuma PE, Simulundu E, Sutcliffe CG. Respiratory viruses in rural Zambia before and during the COVID-19 pandemic. Trop Med Int Health. 2022 Jul;27(7):647-654. doi: 10.1111/tmi.13781. Epub 2022 Jun 8. PMID: 35611546; PMCID: PMC9348166.	Inappropriate population.
202	Lokida D, Farida H, Triasih R, Mardian Y, Kosasih H, Naysilla AM, Budiman A, Hayuningsih C, Anam MS, Wastoro D, Mujahidah M, Dipayana S, Setyati A, Aman AT, Lukman N, Karyana M, Kline A, Neal A, Lau CY, Lane C. Epidemiology of community-acquired pneumonia among hospitalised children in Indonesia: a multicentre, prospective study. BMJ Open. 2022 Jun 21;12(6):e057957. doi: 10.1136/bmjopen-2021-057957. PMID: 35728910; PMCID: PMC9214401.	Prevalence not reported by RSV season individually.
203	Low YL, Wong SY, Lee EKH, Muhammed MH. Prevalence of respiratory viruses among paediatric patients in acute respiratory illnesses in Malaysia. PLoS One. 2022 Aug 3;17(8):e0265288. doi: 10.1371/journal.pone.0265288. PMID: 35921317; PMCID: PMC9348681.	Inappropriate population.
204	Lu L, Robertson G, Ashworth J, Pham Hong A, Shi T, Ivens A, Thwaites G, Baker S, Woolhouse M. Epidemiology and Phylogenetic Analysis of Viral Respiratory Infections in Vietnam. Front Microbiol. 2020 May 15;11:833. doi: 10.3389/fmicb.2020.00833. PMID: 32499763; PMCID: PMC7242649.	Prevalence not reported by RSV season individually.

205	Lumley SF, Richens N, Lees E, Cregan J, Kalimeris E, Oakley S, Morgan M, Segal S, Dawson M, Walker AS, et al. Changes in paediatric respiratory infections at a UK teaching hospital 2016-2021; impact of the SARS-CoV-2 pandemic. J Infect. 2022 Jan;84(1):40-47. doi: 10.1016/j.jinf.2021.10.022. Epub 2021 Oct 29. PMID: 34757137; PMCID: PMC8591975.	Incomplete data on RSV prevalence
206	Luo HJ, Huang XB, Zhong HL, Ye CX, Tan X, Zhou K, Yuan L, Zhang SF, Zhu X, Lin CJ, Wang WJ, Xu L, Cao KY. Epidemiological characteristics and phylogenic analysis of human respiratory syncytial virus in patients with respiratory infections during 2011-2016 in southern China. Int J Infect Dis. 2020 Jan;90:5-17. doi: 10.1016/j.ijid.2019.10.009. Epub 2019 Oct 18. PMID: 31634614; PMCID: PMC7110755.	Incomplete data on RSV prevalence
207	Luo Q, Li M, Li A, Gong C, Dong M, Huang Q, Luo M, Zhang H, Huang F. Genetic diversity and epidemiological features of respiratory syncytial virus, Beijing, 2015-2019: A multicenter and all-age groups study. J Infect. 2022 Jul;85(1):75-85. doi: 10.1016/j.jinf.2022.04.046. Epub 2022 May 6. PMID: 35533834.	Inappropriate population and RSV prevalence not reported.
208	Macin S, Findik D. Viral Pathogens and Seasonal Distribution in Respiratory Tract Infections. Flora Infeksiyon Hastalıkları ve Klinik Mikrobiyoloji Dergisi. 2020;25(1):69-75. DOI: 10.5578/flora.68758.	Inappropriate population.
209	Madaniyazi L, Seposo X, Ng CFS, Tobias A, Toizumi M, Moriuchi H, Yoshida LM, Hashizume M. Respiratory Syncytial Virus Outbreaks Are Predicted after the COVID-19 Pandemic in Tokyo, Japan. Jpn J Infect Dis. 2022 Mar 24;75(2):209-211. doi: 10.7883/yoken.JJID.2021.312. Epub 2021 Aug 31. PMID: 34470964.	Inappropriate population and RSV prevalence not reported.
210	Malhotra B, Swamy MA, Janardhan Reddy PV, Gupta ML. Viruses causing severe acute respiratory infections (SARI) in children ≤5 years of age at a tertiary care hospital in Rajasthan, India. Indian J Med Res. 2016 Dec;144(6):877-885. doi: 10.4103/ijmr.IJMR_22_15. PMID: 28474624; PMCID: PMC5433280.	Period of study before 2015.
211	Manti S, Giallongo A, Parisi GF, Papale M, Presti S, Lo Bianco M, Spicuzza L, Leonardi S. "Impact of COVID-19 Pandemic and Lockdown on the Epidemiology of RSV-Mediated Bronchiolitis: Experience from Our Centre." Children (Basel). 2022 Nov 9;9(11):1723. doi: 10.3390/children9111723. PMID: 36360451; PMCID: PMC9688686.	Incomplete data on RSV prevalence
212	Márquez-Aguirre AC, Bolaños-Macías J, Moreno J, Buitrago J. Characterization of a cohort five years after an episode of bronchiolitis that required hospitalization in a third level clinic of Bogotá, Colombia. Infectio. 2019;23(3):234-239.	Period of study before 2015.

213	Mathew JL, Singhi S, Ray P, Hagel E, Saghafian-Hedengren S, Bansal A, Ygberg S, Sodhi KS, Kumar BV, Nilsson A. Etiology of community acquired pneumonia among children in India: prospective, cohort study. J Glob Health. 2015 Dec;5(2):050418. doi: 10.7189/jogh.05.020418. PMID: 26528392; PMCID: PMC4623579.	Period of study before 2015.
214	Mattana G, Albitar-Nehme S, Cento V, Colagrossi L, Piccioni L, Raponi M, Raucci U, Vittucci AC, Reale A, Villani A, Bernaschi P, Perno CF. Back to the future (of common respiratory viruses). J Glob Antimicrob Resist. 2022 Mar;28:223-225. doi: 10.1016/j.jgar.2022.01.010. Epub 2022 Jan 21. PMID: 35074567; PMCID: PMC8779783.	Incomplete data on RSV prevalence
215	McCallum GB, Grimwood K, Oguoma VM, Leach AJ, Smith-Vaughan HC, Versteegh LA, Chang AB. The point prevalence of respiratory syncytial virus in hospital and community-based studies in children from Northern Australia: studies in a 'high-risk' population. Rural Remote Health. 2019 Nov;19(4):5267. doi: 10.22605/RRH5267. Epub 2019 Nov 24. PMID: 31759384.	Prevalence not reported by RSV season individually.
216	Mendes ET, Souza LB, Paranhos HL, Santos ICM. Hospital Outbreak of Respiratory Syncytial Virus in Neonatal Intensive Care Unit: The Risk of Admitting External Patients. Infect Control Hosp Epidemiol. 2020 Oct;41(Special Issue: SI):S251-S252. doi: 10.1017/ice.2020.812.	Inappropriate study design. Abstract for Congress.
217	Methi F, Størdal K, Telle K, Larsen VB, Magnusson K. Hospital Admissions for Respiratory Tract Infections in Children Aged 0-5 Years for 2017/2023. Front Pediatr. 2022 Jan 12;9:822985. doi: 10.3389/fped.2021.822985. PMID: 35096720; PMCID: PMC8790534.	Incomplete data on RSV prevalence
218	Mileva S, Ivanova-Todorova E, Tumagelova-Yuzeir K, Ivelina TT, Korsun NS, Alexiev VA. "Periostin and IFN-γ levels in serum and nasopharyngeal aspirate in infants with viral-induced wheezing - 2 year follow-up." Turk J Pediatr. 2022;64(6):1021-1030. doi: 10.24953/turkjped.2022.465. PMID: 36583884.	Inappropriate population.
219	Miyama T, Iritani N, Nishio T, Ukai T, Satsuki Y, Miyata H, Shintani A, Hiroi S, Motomura K, Kobayashi K. Seasonal shift in epidemics of respiratory syncytial virus infection in Japan. Epidemiol Infect. 2021 Feb 11;149:e55. doi: 10.1017/S0950268821000340. PMID: 33568242; PMCID: PMC8060823.	Inappropriate population and RSV prevalence not reported.
220	Moattari A, Aleyasin S, Emami A, Fyruzi M, Pirbonyeh N. The prevalence of human metapneumovirus and respiratory syncytial virus and coinfection with both in hospitalized children with acute respiratory infection in south of Iran. Arch Pediatr Infect Dis [Internet]. 2015;3(3). Available from: www.scopus.com	Period of study before 2015.
221	Mohebi L, Karami H, Mirsalehi N, Ardestani NH, Yavarian J, Mard-Soltani M, Mokhatri-Azad T, Salimi V. A delayed resurgence of respiratory syncytial virus (RSV) during the COVID-19 pandemic: An unpredictable outbreak in a small proportion of children in the Southwest of Iran, April 2022. J Med Virol. 2022 Dec;94(12):5802-5807. doi: 10.1002/jmv.28065. Epub 2022 Aug 23. PMID: 35961780; PMCID: PMC9538802.	Seasonality not reported.

222	Montgomery AS, Lustik MB, Jones MU, Horseman TS. Respiratory Viral Pathogens in Children Evaluated at Military Treatment Facilities in Oahu, Hawaii From 2014 to 2018: Seasonality and Climatic Factors. J Pediatric Infect Dis Soc. 2021 Apr 30;10(4):517-520. doi: 10.1093/jpids/piaa131. PMID: 33219667.	Prevalence not reported by RSV season individually.
223	Moore DP, Green RJ, Cohen C, et al. Epidemiology and aetiology of community-acquired pneumonia in children: South African Thoracic Society guidelines (part 1). S Afr Med J. 2020 Jul;110(7):583-587. doi: 10.7196/SAMJ.2020.v110i7.14997.	Inappropriate study design. Clinical practice guideline.
224	Moore HC, Le H, Mace A, Blyth CC, Yeoh D, Foley D, Martin A. Interrupted time-series analysis showed unintended consequences of non-pharmaceutical interventions on pediatric hospital admissions. J Clin Epidemiol. 2022 Mar;143:1-10. doi: 10.1016/j.jclinepi.2021.11.021. Epub 2021 Nov 18. PMID: 34801694; PMCID: PMC8600916.	Inappropriate population and RSV prevalence not reported.
225	Movva N, Suh M, Reichert H, Hintze B, Sendak MP, Wolf Z, Carr S, Kaminski T, White M, Fisher K, Wood CT, Fryzek JP, Nelson CB, Malcolm WF. "Respiratory Syncytial Virus During the COVID-19 Pandemic Compared to Historic Levels: A Retrospective Cohort Study of a Health System." J Infect Dis. 2022 Aug 15;226(Suppl 2):S175-S183. doi: 10.1093/infdis/jiac220. PMID: 35968868; PMCID: PMC9377040.	Inappropriate population.
226	Moynihan KM, McGarvey T, Barlow A, Heney C, Gibbons K, Clark JE, Schlebusch S, Schlapbach LJ. Testing for Common Respiratory Viruses in Children Admitted to Pediatric Intensive Care: Epidemiology and Outcomes. Pediatr Crit Care Med. 2020 Jun;21(6):e333-e341. doi: 10.1097/PCC.0000000000002302. PMID: 32343113.	Period of study before 2015.
227	Mrcela D, Markic J, Zhao C, Viskovic DV, Milic P, Copac R, Li Y. "Changes following the Onset of the COVID-19 Pandemic in the Burden of Hospitalization for Respiratory Syncytial Virus Acute Lower Respiratory Infection in Children under Two Years: A Retrospective Study from Croatia." Viruses. 2022 Dec 9;14(12):2746. doi: 10.3390/v14122746. PMID: 36560751; PMCID: PMC9785187.	Incomplete data on RSV prevalence
228	Neisi N, Abbasi S, Makvandi M, Salmanzadeh S, Biparva S, Nahidsamiei R, Ghandali MV, Rasti M, Angali KA. Detection of Common Respiratory Viruses in Patients with Acute Respiratory Infections Using Multiplex Real-Time RT-PCR. Jundishapur Journal of Microbiology. 2019;12(11):e96513. DOI: 10.5812/jjm.96513.	Inappropriate population.
229	Nguyen SN, Nguyen TNT, Vu LT, Nguyen TD. Clinical Epidemiological Characteristics and Risk Factors for Severe Bronchiolitis Caused by Respiratory Syncytial Virus in Vietnamese Children. Int J Pediatr. 2021 Nov 15;2021:9704666. doi: 10.1155/2021/9704666. PMID: 34819958; PMCID: PMC8608547.	Incomplete data on RSV prevalence

230	Nitsch-Osuch A, Kuchar E, Topczewska-Cabanek A, Wardyn K, Życińska K, Brydak L. Incidence and Clinical Course of Respiratory Viral Coinfections in Children Aged 0-59 Months. Adv Exp Med Biol. 2016;905:17-23. doi: 10.1007/5584_2015_185. PMID: 26801151; PMCID: PMC7119935.	Inappropriate study design. Case report.
231	O'Bryant SC, Momin Z, Camp E, Jones J, Meskill S. Longitudinal evaluation of pediatric respiratory infections. J Clin Virol. 2022 Mar;148:105084. doi: 10.1016/j.jcv.2022.105084. Epub 2022 Jan 31. PMID: 35101733.	Prevalence not reported by RSV season individually.
232	Ogunbayo AE, Mogotsi MT, Sondlane H, Nkwadipo KR, Sabiu S, Nyaga MM. "Pathogen Profile of Children Hospitalised with Severe Acute Respiratory Infections during COVID-19 Pandemic in the Free State Province, South Africa." Int J Environ Res Public Health. 2022 Aug 21;19(16):10418. doi: 10.3390/ijerph191610418. PMID: 36012053; PMCID: PMC9408356.	Seasonality not reported.
233	Ogunsemowo O, Olaleye DO, Odaibo GN. Genetic diversity of human respiratory syncytial virus circulating among children in Ibadan, Nigeria. PLoS One. 2018 Jan 23;13(1):e0191494. doi: 10.1371/journal.pone.0191494. PMID: 29360861; PMCID: PMC5779668.	Inappropriate population.
234	Ogunsemowo O, Olaleye DO, Odaibo GN. Human Respiratory Syncytial Virus Subtypes A and B Infection Among Children Attending Primary and Secondary Health Care Facilities in Ibadan, Nigeria. Arch Basic Appl Med. 2018 Feb;6(1):73-78. Epub 2018 May 3. PMID: 29905317; PMCID: PMC5997266.	Inappropriate population.
235	Oladele DM, Oladele DP, Ibraheem RM, Abdulkadir MB, Raheem RA, Gobir AA, Adedoyin OT, Johnson ABR. Reappraisal of respiratory syncytial virus as an aetiology of severe acute lower respiratory tract infections in children younger than 5 years in Nigeria. Trans R Soc Trop Med Hyg. 2019 Aug 1;113(8):446-452. doi: 10.1093/trstmh/trz026. PMID: 31034059.	Period of enrolment not reported.
236	Oliveira-Santos M, Santos JA, Soares J, Dias A, Quaresma M. Influence of meteorological conditions on RSV infection in Portugal. Int J Biometeorol. 2016 Dec;60(12):1807-1817. doi: 10.1007/s00484-016-1168-1. Epub 2016 Apr 9. PMID: 27059367.	Prevalence not reported by RSV season individually.
237	Olsen SJ, Winn AK, Budd AP, Prill MM, Steel J, Midgley CM, Kniss K, Burns E, Rowe T, Foust A, et al. Changes in influenza and other respiratory virus activity during the COVID-19 pandemic-United States, 2020-2021. Am J Transplant. 2021 Oct;21(10):3481-3486. doi: 10.1111/ajt.16049. PMID: 34624182; PMCID: PMC8653380.	Inappropriate study design. Review.
238	Olsen SJ, Winn AK, Budd AP, Prill MM, Steel J, Midgley CM, Kniss K, Burns E, Rowe T, Foust A, Jasso G, Merced-Morales A, Davis CT, Jang Y, Jones J, Daly P, Gubareva L, Barnes J, Kondor R, Sessions W, Smith C, Wentworth DE, Garg S, Havers FP, Fry AM, Hall AJ, Brammer L, Silk BJ. Changes in Influenza and Other Respiratory Virus Activity During the COVID-19 Pandemic - United States, 2020-2021. MMWR Morb Mortal Wkly Rep. 2021 Jul 23;70(29):1013-1019. doi: 10.15585/mmwr.mm7029a1. PMID: 34292924; PMCID: PMC8297694.	Inappropriate population and RSV prevalence not reported.

239	Otani K, Saito M, Okamoto M, Tamaki R, Saito-Obata M, Kamigaki T, Lirio IC, Segubre-Mercado E, Tallo V, Lupisan S, Oshitani H. Incidence of lower respiratory tract infection and associated viruses in a birth cohort in the Philippines. BMC Infect Dis. 2022 Mar 30;22(1):313. doi: 10.1186/s12879-022-07289-3. PMID: 35354368; PMCID: PMC8966153.	Prevalence not reported by RSV season individually.
240	Ouafi M, Dubos F, Engelman I, Lazrek M, Guigon A, Bocket L, Hober D, Alidjinou EK. Rapid syndromic testing for respiratory viral infections in children attending the emergency department during COVID-19 pandemic in Lille, France, 2021-2022. J Clin Virol. 2022 Aug;153:105221. doi: 10.1016/j.jcv.2022.105221. Epub 2022 Jun 25. PMID: 35777223; PMCID: PMC9233550.	Inappropriate population.
241	Ozeki S, Kawada JI, Yamashita D, Yasufuku C, Akano T, Kato M, Suzuki K, Tano C, Matsumoto K, Mizutani SH, Mori A, Nishio N, Kidokoro H, Yasui Y, Takahashi Y, Sato Y; Nagoya Collaborative Clinical Research Team. "Impact of the Coronavirus Disease 2019 Pandemic on the Clinical Features of Pediatric Respiratory Syncytial Virus Infection in Japan." Open Forum Infect Dis. 2022 Oct 22;9(11):ofac562. doi: 10.1093/ofid/ofac562. PMID: 36381619; PMCID: PMC9620303.	Incomplete data on RSV prevalence
242	Ozeki S, Oshiro M, Fukumi D, Takeuchi T, Mii S, Nishikado Y. Change Over Time in Seasonality and Severity of Children Hospitalized With Respiratory Syncytial Virus Infection in Japan. Pediatr Infect Dis J. 2022 Aug 1;41(8):614-619. doi: 10.1097/INF.0000000000003568. Epub 2022 Jul 13. PMID: 35544732.	Incomplete data on RSV prevalence
243	Ozkaya-Parlakay A, Gulhan B, Bedir-Demirdag T, Kanik-Yuksek S. Viral Etiology of Bronchiolitis Among Pediatric Patients. Pediatr Infect Dis J. 2019 Sep;38(9):e233. doi: 10.1097/INF.0000000000002382. PMID: 31408060.	Incomplete data on RSV prevalence
244	Park K, Kim D, Seong J, Shin I, Hong J, Park S, Kang SY, Lee H. Epidemiological features and genetic variation of human respiratory syncytial virus (HRSV) infection in Chungnam, Korea. Biomed Res India. 2017;28(2):967-972.	Period of study before 2015.
245	Park K, Sung H, Kim MN. "Reemergence of Parainfluenza Virus Type 3 and Respiratory Syncytial Virus Infections During the COVID-19 Pandemic." Ann Lab Med. 2023 Jan 1;43(1):114-116. doi: 10.3343/alm.2023.43.1.114. Epub 2022 Sep 1. PMID: 36045068; PMCID: PMC9467829.	Inappropriate population and RSV prevalence not reported.
246	Park S, Michelow IC, Choe YJ. Shifting Patterns of Respiratory Virus Activity Following Social Distancing Measures for Coronavirus Disease 2019 in South Korea. J Infect Dis. 2021 Dec 1;224(11):1900-1906. doi: 10.1093/infdis/jiab231. PMID: 34009376; PMCID: PMC8135809.	Inappropriate population.
247	Paul SP, Mukherjee A, McAllister T, Harvey MJ, Clayton BA, Turner PC. Respiratory-syncytial-virus- and rhinovirus-related bronchiolitis in children aged <2 years in an English district general hospital. J Hosp Infect. 2017 Aug;96(4):360-365. doi: 10.1016/j.jhin.2017.04.023. Epub 2017 May 3. PMID: 28559125; PMCID: PMC7114599.	Prevalence not reported by RSV season individually.

248	Pellegrinelli L, Galli C, Bubba L, Seiti A, Anselmi G, Primache V, Signorini L, Delbue S, Binda S, Pariani E. Respiratory syncytial virus in pediatric influenza-like illness cases in Lombardy, Northern Italy, during seven consecutive winter seasons (from 2014-2015 to 2020-2021). Influenza Other Respir Viruses. 2022 May;16(3):481-491. doi: 10.1111/irv.12940. Epub 2021 Dec 17. PMID: 34921508; PMCID: PMC8983902.	Inappropriate population.
249	Perez A, Lively JY, Curns A, Weinberg GA, Halasa NB, Staat MA, Szilagyi PG, Stewart LS, McNeal MM, Clopper B, Zhou Y, Whitaker BL, LeMasters E, Harker E, Englund JA, Klein EJ, Selvarangan R, Harrison CJ, Boom JA, Sahni LC, Michaels MG, Williams JV, Langley GE, Gerber SI, Campbell A, Hall AJ, Rha B, McMorrow M; New Vaccine Surveillance Network Collaborators. "Respiratory Virus Surveillance Among Children with Acute Respiratory Illnesses - New Vaccine Surveillance Network, United States, 2016-2021." MMWR Morb Mortal Wkly Rep. 2022 Oct 7;71(40):1253-1259. doi: 10.15585/mmwr.mm7140a1. PMID: 36201373; PMCID: PMC9541034.	Incomplete data on RSV prevalence
250	Pérez-López A, Al Mana H, Iqbal M, Suleiman M, Hasan MR, Tang P. Variations in respiratory syncytial virus activity following the relaxation of COVID-19 restrictions in Qatar. J Travel Med. 2022 Sep 17;29(6):taac065. doi: 10.1093/jtm/taac065. PMID: 35608003; PMCID: PMC9384098.	Seasonality not reported.
251	Petrarca L, Nenna R, Frassanito A, Pierangeli A, Leonardi S, Scagnolari C, Antonelli G, Papoff P, Moretti C, Midulla F. Acute bronchiolitis: Influence of viral co-infection in infants hospitalized over 12 consecutive epidemic seasons. J Med Virol. 2018 Apr;90(4):631-638. doi: 10.1002/jmv.24994. Epub 2017 Dec 11. PMID: 29226974; PMCID: PMC7166564.	Prevalence not reported by RSV season individually.
252	Pham HT, Nguyen TNT, Tran QA, Ngo TT. Prevalence and Associated Factors with Mixed Coinfections among under 5-Year-Old Children with Severe Viral Pneumonia in Vietnam. Journal of Child Science. 2020;10(1):E74-E79. DOI: 10.1055/s-0040-1713623.	Prevalence not reported by RSV season individually.
253	Phyu WW, Htwe KTZ, Saito R, Kyaw Y, Lin N, Dapat C, Osada H, Chon I, Win SMK, Hibino A, Wagatsuma K, Kyaw LL, Tin HH, Watanabe H. Evolutionary analysis of human respiratory syncytial virus collected in Myanmar between 2015 and 2018. Infect Genet Evol. 2021 Sep;93:104927. doi: 10.1016/j.meegid.2021.104927. Epub 2021 May 18. PMID: 34020068.	Inappropriate population.
254	Piralla A, Mariani B, Rovida F, Baldanti F. Frequency of respiratory viruses among patients admitted to 26 Intensive Care Units in seven consecutive winter-spring seasons (2009-2016) in Northern Italy. J Clin Virol. 2017 Jul;92:48-51. doi: 10.1016/j.jcv.2017.05.004. Epub 2017 May 8. PMID: 28527970; PMCID: PMC7172430.	Inappropriate population.
255	Quandelacy TM, Adams LE, Munoz J, Santiago GA, Kada S, Johansson MA, Alvarado LI, Rivera-Amill V, Paz-Bailey G. Reduced spread of influenza and other respiratory viral infections during the COVID-19 pandemic in southern Puerto Rico. PLoS One. 2022 Apr 27;17(4):e0266095. doi: 10.1371/journal.pone.0266095. PMID: 35476785; PMCID: PMC9045654.	Inappropriate population and RSV

		prevalence not reported.
256	Rabarison JH, Tempia S, Harimanana A, Guillebaud J, Razanajatovo NH, Ratsitorahina M, Heraud JM. Burden and epidemiology of influenza- and respiratory syncytial virus-associated severe acute respiratory illness hospitalization in Madagascar, 2011-2016. Influenza Other Respir Viruses. 2019 Mar;13(2):138-147. doi: 10.1111/irv.12557. Epub 2018 Dec 27. PMID: 30596225; PMCID: PMC6379640.	Incomplete data on RSV prevalence
257	Rahbarimanesh AA, Izadi A, Ghajarzadeh M. Viral Aetiology of Bronchiolitis in Hospitalised Children in a Tertiary Center in Tehran. Maedica (Bucur). 2018 Mar;13(1):17-20. PMID: 29868135; PMCID: PMC5972781.	Prevalence not reported by RSV season individually.
258	Ramaekers K, Keyaerts E, Rector A, Borremans A, Beuselinck K, Lagrou K, Van Ranst M. Prevalence and seasonality of six respiratory viruses during five consecutive epidemic seasons in Belgium. J Clin Virol. 2017 Sep;94:72-78. doi: 10.1016/j.jcv.2017.07.011. Epub 2017 Jul 23. PMID: 28772168.	Inappropriate population.
259	Rankin DA, Haddadin Z, Lipworth L, Stahl AL, Fryzek J, Suh M, Shepard DS, Varjabedian R, Fernandez KN, Salib S, Villarreal J, Bruce M, McHenry R, Spieker AJ, Nelson CB, Halasa NB. Comparison of clinical presentations and burden of respiratory syncytial virus in infants across three distinct healthcare settings in Davidson County, Tennessee. Ther Adv Infect Dis. 2022 Jul 18;9:20499361221112171. doi: 10.1177/20499361221112171. PMID: 35875809; PMCID: PMC9297461.	Duplicate with Haddadin et al., J Pediatr. 2021;234:164-171.e2. doi:10.1016/j.jpeds.2021.03.036
260	Redlberger-Fritz M, Kundi M, Aberle SW, Puchhammer-Stöckl E. Significant impact of nationwide SARS-CoV-2 lockdown measures on the circulation of other respiratory virus infections in Austria. J Clin Virol. 2021 Apr;137:104795. doi: 10.1016/j.jcv.2021.104795. Epub 2021 Mar 16. PMID: 33761423; PMCID: PMC7962988.	Inappropriate population.
261	Reeves RM, van Wijhe M, Tong S, Lehtonen T, Stona L, Teirlinck AC, Fernandez LV, Li Y, Giaquinto C, Fischer TK, Demont C, Heikkinen T, Speltra I, van Boven M, Bøås H, Campbell H; RESCEU Investigators. Respiratory Syncytial Virus-Associated Hospital Admissions in Children Younger Than 5 Years in 7 European Countries Using Routinely Collected Datasets. J Infect Dis. 2020 Oct 7;222(Suppl 7):S599-S605. doi: 10.1093/infdis/jiaa360. PMID: 32815542.	Incomplete data on RSV prevalence
262	Reina J, del Barrio E, Morales C, Busquets M, Norte C. Analysis of the population dynamics of pediatric acute respiratory infections caused by viruses (2015-2016). Med Balear. 2018;33(2):42-47. doi: 10.3306/MEDICINABALEAR.33.02.42.	Language not in English.

263	Reis J, Shaman J. Simulation of four respiratory viruses and inference of epidemiological parameters. Infect Dis Model. 2018 Mar 19;3:23-34. doi: 10.1016/j.idm.2018.03.006. PMID: 30839912; PMCID: PMC6326234.	Period of study before 2015.
264	Ren GL, Wang XF, Xu J, Li J, Meng Q, Xie GQ, Huang B, Zhu WC, Lin J, Tang CH, Ye S, Li Z, Zhu J, Tang Z, Ma MX, Xie C, Wu YW, Liu CX, Yang F, Zhou YZ, Zheng Y, Lan SL, Chen JF, Ye F, He Y, Wu BQ, Chen L, Fu SM, Zheng CZ, Shi Y. Comparison of acute pneumonia caused by SARS-COV-2 and other respiratory viruses in children: a retrospective multi-center cohort study during COVID-19 outbreak. Mil Med Res. 2021 Feb 16;8(1):13. doi: 10.1186/s40779-021-00306-7. PMID: 33593415; PMCID: PMC7886299.	Seasonality not reported.
265	Ren S-, Shao X-, Shi T, Shan W, Chen Q-, Xue J, Tian J-, Zhang T, Zhao G-. Analysis of clinical characteristics and influencing factors of respiratory syncytial virus infection among children under 5 years old in Suzhou. Chin J Dis Control Prev. 2021;25(11):1336-40. Available from: www.scopus.com	Language not in English.
266	Reyes Domínguez AI, Pavlovic Nesic S, Urquía Martí L, Pérez González MDC, Reyes Suárez D, García-Muñoz Rodrigo F. Effects of public health measures during the SARS-CoV-2 pandemic on the winter respiratory syncytial virus epidemic: An interrupted time series analysis. Paediatr Perinat Epidemiol. 2022 May;36(3):329-336. doi: 10.1111/ppe.12829. Epub 2022 Jan 4. PMID: 34981845; PMCID: PMC8692060.	Incomplete data on RSV prevalence
267	Rodgers L, Sheppard M, Smith A, Dietz S, Jayanthi P, Yuan Y, Bull L, Wotiz S, Schwarze T, Azondekon R, Hartnett K, Adjemian J, Kirking HL, Kite-Powell A. Changes in Seasonal Respiratory Illnesses in the United States During the Coronavirus Disease 2019 (COVID-19) Pandemic. Clin Infect Dis. 2021 Jul 15;73(Suppl 1):S110-S117. doi: 10.1093/cid/ciab311. PMID: 33912902; PMCID: PMC8135472.	Inappropriate population and RSV prevalence not reported.
268	Russo M, Avaro MM, Czech A, Benedetti E, Pardon F, Campos AM, Macias EM, Pontoriero A, Baumeister E. Epidemiology and molecular characterization of Respiratory Syncytial Virus in Argentina 2017. Int J Infect Dis. 2018 Aug;73(Supplement):200-200. doi: 10.1016/j.ijid.2018.04.3868.	Inappropriate study design. Abstract for Congress.
269	Ryabichenko TI, Skosyreva GA, Obukhova OO, Kurskaya OG, Kosyanova TG, Gorbenko OM, et al. Etiological structure of acute respiratory viral infections in children hospitalized in 2015-2018. Infect Dis News Opin Train. 2021;10(2):47-53.	Language not in English.
270	Rybak A, Levy C, Angoulvant F, Auvrignon A, Gembara P, Danis K, Vaux S, Levy-Bruhl D, van der Werf S, Béchet S, Bonacorsi S, Assad Z, Lazzati A, Michel M, Kaguelidou F, Faye A, Cohen R, Varon E, Ouldali N. Association of Nonpharmaceutical Interventions During the COVID-19 Pandemic With Invasive Pneumococcal Disease, Pneumococcal Carriage, and Respiratory Viral Infections Among Children in France. JAMA Netw Open. 2022 Jun 1;5(6):e2218959. doi: 10.1001/jamanetworkopen.2022.18959. PMID: 35763298; PMCID: PMC9240903.	Inappropriate population and RSV prevalence not reported.

271	Rybak A, Levy C, Jung C, Béchet S, Batard C, Hassid F, Zouari M, Cahn-Sellem F, Bangert M, Cohen R. Delayed Bronchiolitis Epidemic in French Primary Care Setting Driven by Respiratory Syncytial Virus: Preliminary Data from the Oursyn Study, March 2021. Pediatr Infect Dis J. 2021 Dec 1;40(12):e511-e514. doi: 10.1097/INF.0000000000003270. PMID: 34260480.	Seasonality not reported.
272	Rząd M, Kanecki K, Lewtak K, Tyszko P, Szwejkowska M, Goryński P, Nitsch-Osuch A. "Human Respiratory Syncytial Virus Infections among Hospitalized Children in Poland during 2010-2020: Study Based on the National Hospital Registry." J Clin Med. 2022 Oct 31;11(21):6451. doi: 10.3390/jcm11216451. PMID: 36362679; PMCID: PMC9656302.	Prevalence not reported by RSV season individually.
273	Sáez-López E, Cristóvão P, Costa I, Pechirra P, Conde P, Guiomar R; Portuguese Laboratory Network for the Diagnosis of Influenza Infection; Peres MJ, Viseu R, Lopes P, Soares V, Vale F, Fonseca P, Freitas L, Alves J, Pessanha MA, Toscano C, Mota-Vieira L, Veloso RC, Côrte-Real R, Branquinho P, Pereira-Vaz J, Rodrigues F, Cunha M, Martins L, Mota P, Couto AR, Bruges-Armas J, Almeida S, Rodrigues D. Epidemiology and genetic variability of respiratory syncytial virus in Portugal, 2014-2018. J Clin Virol. 2019 Dec;121:104200. doi: 10.1016/j.jcv.2019.104200. Epub 2019 Oct 10. PMID: 31707201; PMCID: PMC7106440.	Incomplete data on RSV prevalence
274	Sáez-López E, Pechirra P, Costa I, Cristóvão P, Conde P, Machado A, Rodrigues AP, Guiomar R. Performance of surveillance case definitions for respiratory syncytial virus infections through the sentinel influenza surveillance system, Portugal, 2010 to 2018. Euro Surveill. 2019 Nov;24(45):1900140. doi: 10.2807/1560-7917.ES.2019.24.45.1900140. PMID: 31718741; PMCID: PMC6852315.	Incomplete data on RSV prevalence
275	Sánchez García L, Calvo C, Casas I, Pozo F, Pellicer A. Viral respiratory infections in very low birthweight infants at neonatal intensive care unit: prospective observational study. BMJ Paediatr Open. 2020 Sep 22;4(1):e000661. doi: 10.1136/bmjpo-2020-000661. PMID: 33024832; PMCID: PMC7513636.	Inappropriate population and seasonality not reported.
276	Santos RO, Borges IC, Souza ML, Bouzas ML, Nascimento-Carvalho CM. Seasonality of distinct respiratory viruses in a tropical city: implications for prophylaxis. Trop Med Int Health. 2021 Jun;26(6):672-679. doi: 10.1111/tmi.13571. Epub 2021 Apr 18. PMID: 33666303.	Inappropriate population and seasonality not reported.
277	Saravanos GL, Hu N, Homaira N, Muscatello DJ, Jaffe A, Bartlett AW, Wood NJ, Rawlinson W, Kesson A, Lingam R, Britton PN. RSV Epidemiology in Australia Before and During COVID-19. Pediatrics. 2022 Feb 1;149(2):e2021053537. doi: 10.1542/peds.2021-053537. PMID: 35083489.	Inappropriate population.

278	Satav A, Crow R, Potdar V, Dani V, Satav S, Chadha M, Hessong D, Carosone-Link P, Palaskar S, Simões EAF. The Burden of Respiratory Syncytial Virus in Children Under 2 Years of Age in a Rural Community in Maharashtra, India. Clin Infect Dis. 2021 Sep 2;73(Suppl_3):S238-S247. doi: 10.1093/cid/ciab508. PMID: 34472575; PMCID: PMC8411254.	Inappropriate population and RSV prevalence not reported.
279	Sberna G, Lalle E, Valli MB, Bordi L, Garbuglia AR, Amendola A. Changes in the Circulation of Common Respiratory Pathogens among Hospitalized Patients with Influenza-like Illnesses in the Lazio Region (Italy) during Fall Season of the Past Three Years. Int J Environ Res Public Health. 2022 May 13;19(10):5962. doi: 10.3390/ijerph19105962. PMID: 35627498; PMCID: PMC9141595.	Inappropriate population and RSV prevalence not reported.
280	Sert S, Emiroğlu M, Arslan U, Koç O, Örs R. Clinical characteristics and incidence of bacterial and viral pathogens in patients hospitalized with community acquired pneumonia in childhood in Konya between October 2008 and February 2010. Turk Hijyen Deney Biyoloji Dergisi [Internet]. 2016;73(2):101-10. Available from: www.scopus.com	Period of study before 2015.
281	Shati AA, Ali AS, Al-Hakami AM, Asseri AA, Al-Qahatani SM. Pediatric viral respiratory infections in Saudi Arabia: Narrative and descriptive revisits for the etiology, epidemiology and clinical phenotypes with diagnostic challenges highlights. Kuwait Med J. 2020 Jun;52(2):118-128.	Inappropriate study design.
282	Shi HJ, Kim NY, Eom SA, Kim-Jeon MD, Oh SS, Moon BS, Kwon MJ, Eom JS. Effects of Non-Pharmacological Interventions on Respiratory Viruses Other Than SARS-CoV-2: Analysis of Laboratory Surveillance and Literature Review From 2018 to 2021. J Korean Med Sci. 2022 May 30;37(21):e172. doi: 10.3346/jkms.2022.37.e172. PMID: 35638198; PMCID: PMC9151990.	Inappropriate population and RSV prevalence not reported.
283	Shinoda D, Tsukagoshi H, Komuro K, Yoshida D, Yanaoka T, Saito M, Saruki N. Detection of Respiratory Viruses during the Early Phase of the Coronavirus Disease 2019 Pandemic in Ibaraki and Gunma Prefectures, Japan. Jpn J Infect Dis. 2022 Sep 22;75(5):530-532. doi: 10.7883/yoken.JJID.2022.061. Epub 2022 Apr 28. PMID: 35491230.	Inappropriate population.
284	Şık N, Çakan Başerdem KA, Başerdem O, Appak Ö, Sayıner AA, Yılmaz D, Duman M. Distribution of Viral Respiratory Pathogens During the COVID-19 Pandemic: A Single-Center Pediatric Study from Turkey. Turk Arch Pediatr. 2022 May;57(3):354-359. doi: 10.5152/TurkArchPediatr.2022.21350. PMID: 35781241; PMCID: PMC9131830.	Inappropriate population.
285	Sim JY, Chen YC, Hsu WY, Chen WY, Chou Y, Chow JC, Lai YC, Tang HJ, Chen CC, Ho CH, Chang TH. Circulating pediatric respiratory pathogens in Taiwan during 2020: Dynamic change under low COVID-19 incidence. J Microbiol Immunol Infect. 2022 Dec;55(6 Pt 2):1151-1158. doi: 10.1016/j.jmii.2022.03.005. Epub 2022 Apr 14. PMID: 35450828; PMCID: PMC9767794.	Inappropriate population and RSV prevalence not reported.

286	Sitthikarnkha P, Uppala R, Niamsanit S, Sutra S, Thepsuthammarat K, Techasatian L, Niyomkarn W, Teeratakulpisarn J. "Burden of Respiratory Syncytial Virus Related Acute Lower Respiratory Tract Infection in Hospitalized Thai Children: A 6-Year National Data Analysis." Children (Basel). 2022 Dec 17;9(12):1990. doi: 10.3390/children9121990. PMID: 36553433; PMCID: PMC9776945.	Incomplete data on RSV prevalence
287	Sitthikarnkha P, Uppala R, Niamsanit S, Sutra S, Thepsuthammarat K, Techasatian L, Teeratakulpisarn J. Epidemiology of acute lower respiratory tract infection hospitalizations in Thai children: A 5-year national data analysis. Influenza Other Respir Viruses. 2022 Jan;16(1):142-150. doi: 10.1111/irv.12911. Epub 2021 Sep 15. PMID: 34523811; PMCID: PMC8692802.	Prevalence not reported by RSV season individually.
288	Smirnova SS, Lelenkova EB, Markaryan AY, Vyalykh IV, Alimov AV. Clinical, epidemiological and etiological features of severe acute respiratory infections in hospitalized patients. Infektsionnye Bolezni [Internet]. 2021;19(1):64-70. Available from: www.scopus.com	Language not in English.
289	Smith M, Kubale J, Kuan G, Ojeda S, Vydiswaran N, Sanchez N, Gresh L, Latta K, Lopez R, Patel M, Balmaseda A, Gordon A. "Respiratory Syncytial Virus Incidence and Severity in a Community-Based Prospective Cohort of Children Aged 0-14 Years." Open Forum Infect Dis. 2022 Nov 8;9(11):ofac598. doi: 10.1093/ofid/ofac598. PMID: 36447616; PMCID: PMC9697591.	Incomplete data on RSV prevalence
290	Sominina AA, Danilenko DM, Komissarov AB, Fadeev AV, Pisareva MM, Eropkin MY, Konovalova NI, Petrova PA, Shtro AA, Stolyarov KA, Karpova LS, Burtseva EI, Vasin AV. Summary of influenza and other respiratory viruses detected and characterized in Russia during 2017–2018 season. Rus J Infect Immun [Internet]. 2018;8(4):473-88. Available from: www.scopus.com	Language not in English.
291	Sominina AA, Danilenko DM, Stolyarov KA, Karpova LS, Bakaev MI, Levanyuk TP, Burtseva EI, Lioznov DA. Interference of SARS-CoV-2 with other respiratory viral infections agents during pandemic. Epidemiol Vaktsinoprofilaktika [Internet]. 2021;20(4):28-39. Available from: www.scopus.com	Language not in English.
292	Sonawane AA, Shastri J, Bavdekar SB. Respiratory Pathogens in Infants Diagnosed with Acute Lower Respiratory Tract Infection in a Tertiary Care Hospital of Western India Using Multiplex Real Time PCR. Indian J Pediatr. 2019 May;86(5):433-438. doi: 10.1007/s12098-018-2840-8. Epub 2019 Jan 14. PMID: 30637585; PMCID: PMC7091426.	Period of study before 2015.
293	Song SL, Li MQ, Sun YP. [Analysis of respiratory syncytial virus detection outcomes of 973 cases with severe respiratory infection during 2016-2019, Yuhang district of Hangzhou]. Zhonghua Yu Fang Yi Xue Za Zhi. 2021 Feb 6;55(2):263-265. Chinese. doi: 10.3760/cma.j.cn112150-20201125-01395. PMID: 34645190.	Language not in English.
294	Souza AP, Leitão LA, Luisi F, Souza RG, Coutinho SE, Silva JR, Mattiello R, Pitrez PM, Stein RT, Pinto LA. Lack of association between viral load and severity of acute bronchiolitis in infants. J Bras Pneumol. 2016 Jul- Aug;42(4):261-265. doi: 10.1590/S1806-37562015000000241. PMID: 27832233; PMCID: PMC5063442.	Period of study before 2015.

295	Staadegaard L, Caini S, Wangchuk S, Thapa B, de Almeida WAF, de Carvalho FC, Fasce RA, Bustos P, Kyncl J, Novakova L, Caicedo AB, de Mora Coloma DJ, Meijer A, Hooiveld M, Huang QS, Wood T, Guiomar R, Rodrigues AP, Lee VJM, Ang LW, Cohen C, Moyes J, Larrauri A, Delgado-Sanz C, Demont C, Bangert M, Dückers M, van Summeren J, Paget J. Defining the seasonality of respiratory syncytial virus around the world: National and subnational surveillance data from 12 countries. Influenza Other Respir Viruses. 2021 Nov;15(6):732-741. doi: 10.1111/irv.12885. Epub 2021 Jul 13. PMID: 34255934; PMCID: PMC8542954.	Inappropriate population and RSV prevalence not reported.
296	Staadegaard L, Caini S, Wangchuk S, Thapa B, de Almeida WAF, de Carvalho FC, Njouom R, Fasce RA, Bustos P, Kyncl J, Novakova L, Caicedo AB, Coloma DJD, Meijer A, Hooiveld M, Huang S, Wood T, Guiomar R, Rodrigues AP, Danilenko D, Stolyarov K, Lee VJM, Ang L, Cohen C, Moyes J, Larrauri A, Delgado-Sanz C, Le MQ, Hoang PVM, Demont C, Bangert M, van Summeren J, Duckers M, Paget J. The global epidemiology of RSV in community and hospitalized care: findings from 15 countries. Open Forum Infect Dis. 2021 Jul;8(7):ofab159. doi: 10.1093/ofid/ofab159.	Inappropriate study design.
297	Staadegaard L, Caini S, Wangchuk S, Thapa B, de Almeida WAF, de Carvalho FC, Njouom R, Fasce RA, Bustos P, Kyncl J, Novakova L, Caicedo AB, de Mora Coloma DJ, Meijer A, Hooiveld M, Huang S, Wood T, Guiomar R, Rodrigues AP, Danilenko D, Stolyarov K, Lee VJM, Ang LW, Cohen C, Moyes J, Larrauri A, Delgado-Sanz C, Le MQ, Hoang PVM, Demont C, Bangert M, van Summeren J, Dückers M, Paget J. The Global Epidemiology of RSV in Community and Hospitalized Care: Findings From 15 Countries. Open Forum Infect Dis. 2021 Mar 30;8(7):ofab159. doi: 10.1093/ofid/ofab159. PMID: 34337092; PMCID: PMC8320297.	Incomplete data on RSV prevalence
298	Staadegaard L, Meijer A, Rodrigues AP, Huang S, Cohen C, Demont C, van Summeren J, Caini S, Paget J. Temporal Variations in Respiratory Syncytial Virus Epidemics, by Virus Subtype, 4 Countries. Emerg Infect Dis. 2021 May;27(5):1537-1540. doi: 10.3201/eid2705.204615. PMID: 33900190; PMCID: PMC8084496.	Inappropriate population and RSV prevalence not reported.
299	Stamm P, Sagoschen I, Weise K, Plachter B, Münzel T, Gori T, Vosseler M. Influenza and RSV incidence during COVID-19 pandemic-an observational study from in-hospital point-of-care testing. Med Microbiol Immunol. 2021 Dec;210(5-6):277-282. doi: 10.1007/s00430-021-00720-7. Epub 2021 Oct 4. PMID: 34604931; PMCID: PMC8487758.	Inappropriate population.
300	Subissi L, Bossuyt N, Reynders M, Gérard M, Dauby N, Bourgeois M, Delaere B, Quoilin S, Van Gucht S, Thomas I, Barbezange C. Capturing respiratory syncytial virus season in Belgium using the influenza severe acute respiratory infection surveillance network, season 2018/19. Euro Surveill. 2020 Oct;25(39):1900627. doi: 10.2807/1560-7917.ES.2020.25.39.1900627. PMID: 33006303; PMCID: PMC7531071.	Seasonality not reported.

301	Suh M, Movva N, Jiang X, Bylsma LC, Reichert H, Fryzek JP, Nelson CB. "Respiratory Syncytial Virus Is the Leading Cause of United States Infant Hospitalizations, 2009-2019: A Study of the National (Nationwide) Inpatient Sample." J Infect Dis. 2022 Aug 15;226(Suppl 2):S154-S163. doi: 10.1093/infdis/jiac120. PMID: 35968878; PMCID: PMC9377046.	Incomplete data on RSV prevalence
302	Sun YP, Qiang HS, Lei SY, Zheng XY, Zhang HX, Su YY, Zheng ZZ, Zhang J, Lin XZ, Zhou YL. Epidemiological Features, Risk Factors, and Disease Burden of Respiratory Viruses among Hospitalized Children with Acute Respiratory Tract Infections in Xiamen, China. Jpn J Infect Dis. 2022 Nov 22;75(6):537-542. doi: 10.7883/yoken.JJID.2022.097. Epub 2022 Jun 30. PMID: 35768274.	Prevalence not reported by RSV season individually.
303	Swamy MA, Malhotra B, Janardhan Reddy PV, Tiwari J. Profile of respiratory pathogens causing acute respiratory infections in hospitalised children at Rajasthan: a 4-year study. Indian J Med Microbiol. 2018 Apr-Jun;36(2):163-171. doi: 10.4103/ijmm.IJMM_18_84. PMID: 30084405.	Prevalence not reported by RSV season individually.
304	Tabatabai J, Ihling CM, Rehbein RM, Schnee SV, Hoos J, Pfeil J, Grulich-Henn J, Schnitzler P. Molecular epidemiology of respiratory syncytial virus in hospitalised children in Heidelberg, Southern Germany, 2014-2017. Infect Genet Evol. 2022 Mar;98:105209. doi: 10.1016/j.meegid.2022.105209. Epub 2022 Jan 12. PMID: 35032683.	Incomplete data on RSV prevalence
305	Tan J, Wu J, Jiang W, Huang L, Ji W, Yan Y, Wang M, Shao X. Etiology, clinical characteristics and coinfection status of bronchiolitis in Suzhou. BMC Infect Dis. 2021 Feb 1;21(1):135. doi: 10.1186/s12879-021-05772-x. PMID: 33522910; PMCID: PMC7851904.	Prevalence not reported by RSV season individually.
306	Tan KWJ, Yung CF, Maiwald M, Saffari SE, Thoon KC, Chong CY. Respiratory viral infections in hospitalised paediatric patients in the tropics. J Paediatr Child Health. 2021 Apr;57(4):559-565. doi: 10.1111/jpc.15267. Epub 2020 Nov 13. PMID: 33185937.	Prevalence not reported by RSV season individually.
307	Tao RJ, Luo XL, Xu W, Mao B, Dai RX, Li CW, Yu L, Gu F, Liang S, Lu HW, Chen KB, Bai JW, Ji XB, Gu SY, Sun XL, Dai FH, Jiang P, Cao WJ, Xu JF. Viral infection in community-acquired pneumonia patients with fever: a prospective observational study. J Thorac Dis. 2018 Jul;10(7):4387-4395. doi: 10.21037/jtd.2018.06.33. PMID: 30174887; PMCID: PMC6105945.	Inappropriate population.
308	Teck KS, Mac Guad R, Van Rostenberghe AH, Hua GS. Prevalence, risk factors and clinical characteristics of respiratory syncytial virus-associated lower respiratory tract infections in Kelantan, Malaysia. J Med Virol. 2019 Sep;91(9):1608-1615. doi: 10.1002/jmv.25500. Epub 2019 May 29. PMID: 31074499.	Period of enrolment not reported.
309	Tempia S, Walaza S, Bhiman JN, McMorrow ML, Moyes J, Mkhencele T, Meiring S, Quan V, Bishop K, McAnerney JM, von Gottberg A, Wolter N, Du Plessis M, Treurnicht FK, Hellferscee O, Dawood H, Naby F, Variava E, Siwele C, Baute N, Nel J, Reubenson G, Zar HJ, Cohen C. Decline of influenza and respiratory syncytial virus detection in facility-based surveillance during the COVID-19 pandemic, South Africa,	Inappropriate population.

	January to October 2020. Euro Surveill. 2021 Jul;26(29):2001600. doi: 10.2807/1560-7917.ES.2021.26.29.2001600. PMID: 34296675; PMCID: PMC8299743.	
310	Terliesner N, Unterwalder N, Edelmann A, Corman V, Knaust A, Rosenfeld L, Gratopp A, Ringe H, Martin L, von Bernuth H, Mall MA, Kallinich T. "Viral infections in hospitalized children in Germany during the COVID-19 pandemic: Association with non-pharmaceutical interventions." Front Pediatr. 2022 Aug 11;10:935483. doi: 10.3389/fped.2022.935483. PMID: 36034546; PMCID: PMC9403271.	Incomplete data on RSV prevalence
311	Thomas E, Mattila JM, Lehtinen P, Vuorinen T, Waris M, Heikkinen T. Burden of Respiratory Syncytial Virus Infection During the First Year of Life. J Infect Dis. 2021 Mar 3;223(5):811-817. doi: 10.1093/infdis/jiaa754. PMID: 33350450.	Inappropriate population.
312	Thongpan I, Suntronwong N, Vichaiwattana P, Wanlapakorn N, Vongpunsawad S, Poovorawan Y. Respiratory syncytial virus, human metapneumovirus, and influenza virus infection in Bangkok, 2016-2017. PeerJ. 2019 Apr 11;7:e6748. doi: 10.7717/peerj.6748. PMID: 30997293; PMCID: PMC6462397.	Inappropriate population.
313	Toivonen L, Karppinen S, Schuez-Havupalo L, Teros-Jaakkola T, Mertsola J, Waris M, Peltola V. Respiratory syncytial virus infections in children 0-24 months of age in the community. J Infect. 2020 Jan;80(1):69-75. doi: 10.1016/j.jinf.2019.09.002. Epub 2019 Sep 12. PMID: 31521741.	Period of study before 2015.
314	Tokak S, Gulseren YD, Ozdemir M. Determination of Epidemiology and Seasonal Distribution of Viral Agents Detected in Children with Respiratory Tract Infection. Journal of Pediatric Infection. 2019;13(4):E158-E164. DOI: 10.5578/ced.201950. Published: Dec 2019.	Incomplete data on RSV prevalence
315	Torres-Fernandez D, Casellas A, Mellado MJ, Calvo C, Bassat Q. Acute bronchiolitis and respiratory syncytial virus seasonal transmission during the COVID-19 pandemic in Spain: A national perspective from the pediatric Spanish Society (AEP). J Clin Virol. 2021 Dec;145:105027. doi: 10.1016/j.jcv.2021.105027. Epub 2021 Nov 9. PMID: 34781241; PMCID: PMC8575537.	Incomplete data on RSV prevalence
316	Treggiari D, Piubelli C, Formenti F, Silva R, Perandin F. "Resurgence of Respiratory Virus after Relaxation of COVID-19 Containment Measures: A Real-World Data Study from a Regional Hospital of Italy." Int J Microbiol. 2022 Nov 25;2022:4915678. doi: 10.1155/2022/4915678. PMID: 36466968; PMCID: PMC9718619.	Inappropriate population and RSV prevalence not reported.
317	Tripathi S, Al-Sayyed B, Gladfelter TR. Comparative epidemiology, hospital course, and outcomes of viral respiratory infections in hospitalized pediatric patients. Indian J Med Microbiol. 2021 Jan;39(1):24-29. doi: 10.1016/j.ijmmb.2020.10.011. Epub 2020 Nov 9. PMID: 33610252.	Prevalence not reported by RSV season individually.

318	Tsitsiklis A, Osborne CM, Kamm J, Williamson K, Kalantar K, Dudas G, Caldera S, Lyden A, Tan M, Neff N, Soesanto V, Harris JK, Ambroggio L, Maddux AB, Carpenter TC, Reeder RW, Locandro C, Simões EAF, Leroue MK, Hall MW, Zuppa AF, Carcillo J, Meert KL, Sapru A, Pollack MM, McQuillen PS, Notterman DA, Dean JM, Zinter MS, Wagner BD, DeRisi JL, Mourani PM, Langelier CR. Lower respiratory tract infections in children requiring mechanical ventilation: a multicentre prospective surveillance study incorporating airway metagenomics. Lancet Microbe. 2022 Apr;3(4):e284-e293. doi: 10.1016/S2666-5247(21)00304-9. Epub 2022 Mar 9. PMID: 35544065; PMCID: PMC9446282.	Prevalence not reported by RSV season individually.
319	Tsou P, Vadivelan A, Kovvuri M, Garg N, Thangavelu M, Wang Y, Raj S. Association between multiple respiratory viral infections and pediatric intensive care unit admission among infants with bronchiolitis. Arch Pediatr. 2020 Jan;27(1):39-44. doi: 10.1016/j.arcped.2019.11.006. Epub 2019 Nov 25. PMID: 31780096; PMCID: PMC7127245.	Prevalence not reported by RSV season individually.
320	Ueno F, Tamaki R, Saito M, Okamoto M, Saito-Obata M, Kamigaki T, Suzuki A, Segubre-Mercado E, Aloyon HD, Tallo V, Lupisan SP, Oshitani H; RSV Working Group in the Philippines. Age-specific incidence rates and risk factors for respiratory syncytial virus-associated lower respiratory tract illness in cohort children under 5 years old in the Philippines. Influenza Other Respir Viruses. 2019 Jul;13(4):339-353. doi: 10.1111/irv.12639. Epub 2019 Mar 19. PMID: 30891896; PMCID: PMC6586181.	Incomplete data on RSV prevalence
321	Uhteg K, Amadi A, Forman M, Mostafa HH. Circulation of Non-SARS-CoV-2 Respiratory Pathogens and Coinfection with SARS-CoV-2 Amid the COVID-19 Pandemic. Open Forum Infect Dis. 2021 Dec 8;9(3):ofab618. doi: 10.1093/ofid/ofab618. PMID: 35211632; PMCID: PMC8863080.	Inappropriate population.
322	Ujiie M, Tsuzuki S, Nakamoto T, Iwamoto N. Resurgence of Respiratory Syncytial Virus Infections during COVID-19 Pandemic, Tokyo, Japan. Emerg Infect Dis. 2021 Nov;27(11):2969-2970. doi: 10.3201/eid2711.211565. Epub 2021 Aug 13. PMID: 34388086; PMCID: PMC8544984.	Inappropriate population and RSV prevalence not reported.
323	Üzüm Ö, Kanık A, Eliaçık K, Hortu HÖ, Demirçelik Y, Yan M, Helvacı M, Demir BK. Comparison of clinically related factors and treatment approaches in patients with acute bronchiolitis. Turk Pediatri Ars. 2020 Dec 16;55(4):376-385. doi: 10.14744/TurkPediatriArs.2020.46144. PMID: 33414655; PMCID: PMC7750333.	Prevalence not reported by RSV season individually.
324	Van Brusselen D, De Troeyer K, Ter Haar E, Vander Auwera A, Poschet K, Van Nuijs S, Bael A, Stobbelaar K, Verhulst S, Van Herendael B, Willems P, Vermeulen M, De Man J, Bossuyt N, Vanden Driessche K. Bronchiolitis in COVID-19 times: a nearly absent disease? Eur J Pediatr. 2021 Jun;180(6):1969-1973. doi: 10.1007/s00431-021-03968-6. Epub 2021 Jan 30. PMID: 33517482; PMCID: PMC7847293.	Incomplete data on RSV prevalence
325	van Summeren J, Meijer A, Aspelund G, Casalegno JS, Erna G, Hoang U, Lina B; VRS study group in Lyon; de Lusignan S, Teirlinck AC, Thors V, Paget J. Low levels of respiratory syncytial virus activity in Europe during the 2020/21 season: what can we expect in the coming	Inappropriate study design. Review.

	summer and autumn/winter? Euro Surveill. 2021 Jul;26(29):2100639. doi: 10.2807/1560-7917.ES.2021.26.29.2100639. Erratum in: Euro Surveill. 2021 Jul;26(30): PMID: 34296672; PMCID: PMC8299745.	
326	Vanderburg S, Wijayaratne G, Danthanarayana N, Jayamaha J, Piyasiri B, Halloluwa C, Sheng T, Amarasena S, Kurukulasooriya R, Nicholson BP, Peiris JSM, Gray GC, Gunasena S, Nagahawatte A, Bodinayake CK, Woods CW, Devasiri V, Tillekeratne LG. Outbreak of severe acute respiratory infection in Southern Province, Sri Lanka in 2018: a cross-sectional study. BMJ Open. 2020 Nov 6;10(11):e040612. doi: 10.1136/bmjopen-2020-040612. PMID: 33158834; PMCID: PMC7651749.	Seasonality not reported.
327	Varela FH, Scotta MC, Polese-Bonatto M, Sartor ITS, Ferreira CF, Fernandes IR, Zavaglia GO, de Almeida WAF, Arakaki-Sanchez D, Pinto LA, Nader Bastos GA, Nasi LA, Falavigna M, Pitrez PM, Stein RT; COVIDa study group. Absence of detection of RSV and influenza during the COVID-19 pandemic in a Brazilian cohort: Likely role of lower transmission in the community. J Glob Health. 2021 Mar 1;11:05007. doi: 10.7189/jogh.11.05007. PMID: 33791096; PMCID: PMC7979153.	Inappropriate population and seasonality not reported.
328	Vargas Muñoz SM, De Vivero Haddad S, Beltran AM, Bonilla Gonzalez C, Naranjo Vanegas M, Moreno-Lopez S, Rueda-Guevara P, Barrera P, Piñeros JG, Mejía LM, Mesa ML, Restrepo-Gualteros S, Baquero Castañeda OL, Ramírez Varela A. "Incidence, etiology, sociodemographic and clinical characterization of acute respiratory failure in pediatric patients at a high-altitude city: A multicenter cohort study." Front Pediatr. 2022 Dec 15;10:1009375. doi: 10.3389/fped.2022.1009375. PMID: 36619524; PMCID: PMC9815757.	Inappropriate population.
329	Vásquez-Hoyos P, Diaz-Rubio F, Monteverde-Fernandez N, Jaramillo-Bustamante JC, Carvajal C, Serra A, Karsies T, Rotta AT, González-Dambrauskas S; LARed Network. Reduced PICU respiratory admissions during COVID-19. Arch Dis Child. 2020 Oct 7:archdischild-2020-320469. doi: 10.1136/archdischild-2020-320469. Epub ahead of print. PMID: 33028512.	Inappropriate population.
330	Vaux S, Viriot D, Forgeot C, Pontais I, Savitch Y, Barondeau-Leuret A, Smadja S, Valette M, Enouf V, Parent du Chatelet I. Bronchiolitis epidemics in France during the SARS-CoV-2 pandemic: The 2020-2021 and 2021-2022 seasons. Infect Dis Now. 2022 Sep;52(6):374-378. doi: 10.1016/j.idnow.2022.06.003. Epub 2022 Jun 23. PMID: 35753628; PMCID: PMC9222408.	Incomplete data on RSV prevalence
331	Vihikangas T, Palmu S, Koivisto AM, Heikkilä P. Changes in Bronchiolitis Incidence During the Last Two Decades in Tampere, Finland: A Retrospective Study. Pediatr Infect Dis J. 2022 Nov 1;41(11):867-871. doi: 10.1097/INF.0000000000003662. Epub 2022 Jul 28. PMID: 35895894; PMCID: PMC9555828.	Prevalence not reported by RSV season individually.
332	Vila J, Lera E, Andrés C, Piñana M, Rello-Saltor V, Tobeña-Rué M, Balcells J, Benítez-Díaz Z, Aller MB, Muñoz R, Vázquez A, Rodrigo C, Soler-Palacín P, Antón A. "The burden of non-SARS-CoV2 viral lower respiratory tract infections in hospitalized children in Barcelona (Spain): A long-term, clinical, epidemiologic and economic study." Influenza Other Respir Viruses. 2023 Jan;17(1):e13085. doi: 10.1111/irv.13085. Epub 2022 Dec 20. PMID: 36541036; PMCID: PMC9835422.	Incomplete data on RSV prevalence

333	Wagatsuma K, Koolhof IS, Shobugawa Y, Saito R. Decreased human respiratory syncytial virus activity during the COVID-19 pandemic in Japan: an ecological time-series analysis. BMC Infect Dis. 2021 Aug 3;21(1):734. doi: 10.1186/s12879-021-06461-5. PMID: 34344351; PMCID: PMC8329631.	Inappropriate population and RSV prevalence not reported.
334	Walczak M. Respiratory syncytial virus – epidemiologically important pathogen causing respiratory infection in children: Diagnostics, treatment, prophylaxis. Prz Pediatr [Internet]. 2020;49(1):27-34. Available from: www.scopus.com	Language not in English.
335	Wang H, Zheng Y, de Jonge MI, Wang R, Verhagen LM, Chen Y, Li L, Xu Z, Wang W. "Lockdown measures during the COVID-19 pandemic strongly impacted the circulation of respiratory pathogens in Southern China." Sci Rep. 2022 Oct 8;12(1):16926. doi: 10.1038/s41598-022-21430-x. PMID: 36209167; PMCID: PMC9547377.	Seasonality not reported.
336	Wang J, Xiao T, Xiao F, Hong S, Wang S, Lin J, Li Y, Wang X, Yan K, Zhuang D. Time Distributions of Common Respiratory Pathogens Under the Spread of SARS-CoV-2 Among Children in Xiamen, China. Front Pediatr. 2021 Apr 12;9:584874. doi: 10.3389/fped.2021.584874. PMID: 33912516; PMCID: PMC8075055.	Inappropriate population.
337	Wang L, Davis PB, Berger NA, Kaelber DC, Volkow ND, Xu R. "Disruption in seasonality, patient characteristics and disparities of respiratory syncytial virus infection among young children in the US during and before the COVID-19 pandemic: 2010-2022." medRxiv [Preprint]. 2022 Nov 29:2022.11.29.22282887. doi: 10.1101/2022.11.29.22282887. PMID: 36482981; PMCID: PMC9727767.	Inappropriate population and RSV prevalence not reported.
338	Weinberger Opek M, Yeshayahu Y, Glatman-Freedman A, Kaufman Z, Sorek N, Brosh-Nissimov T. Delayed respiratory syncytial virus epidemic in children after relaxation of COVID-19 physical distancing measures, Ashdod, Israel, 2021. Euro Surveill. 2021 Jul;26(29):2100706. doi: 10.2807/1560-7917.ES.2021.26.29.2100706. PMID: 34296678; PMCID: PMC8299746.	Incomplete data on RSV prevalence
339	White DA, Madhi SA, Jeena P, Zar HJ, Morrow BM, Masekela R, Risenga S, Green RJ. Acute viral bronchiolitis in South Africa: Viral aetiology and clinical epidemiology. S Afr Med J. 2016 May;106(5):29-31. doi: 10.7196/SAMJ.2016.v106i5.10444.	Inappropriate study design. Review.
340	Wildenbeest JG, Zuurbier RP, Korsten K, van Houten MA, Billard MN, Derksen-Lazet N, Snape MD, Drysdale SB, Robinson H, Pollard AJ, Heikkinen T, Cunningham S, Leach A, Martinón-Torres F, Rodríguez-Tenreiro Sánchez C, Gómez-Carballa A, Bont LJ; RESCEU Investigators. Respiratory Syncytial Virus Consortium in Europe (RESCEU) Birth Cohort Study: Defining the Burden of Infant Respiratory Syncytial Virus Disease in Europe. J Infect Dis. 2020 Oct 7;222(Suppl 7):S606-S612. doi: 10.1093/infdis/jiaa310. PMID: 32794574.	Inappropriate study design. Protocol.
341	Wilson PT, Baiden F, Brooks JC, Giessler KM, Apio G, Punguyire D, Moresky RT, Sylverken J, Nyarko-Jectey K, Tagbor H, LaRussa PS. Respiratory Pathogens in Children 1 Month to 5 Years of Age Presenting With Undifferentiated Acute Respiratory Distress in 2 District-Level	Inappropriate population.

	Hospitals in Ghana. J Pediatric Infect Dis Soc. 2019 Sep 25;8(4):361-364. doi: 10.1093/jpids/piy090. PMID: 30189029; PMCID: PMC7107477.	
342	Wright PF, Hoen AG, Jarvis JD, Zens MS, Dade EF, Karagas MR, Taube J, Brickley EB. Bronchiolitis hospitalizations in rural New England: clues to disease prevention. Ther Adv Infect Dis. 2022 May 27;9:20499361221099447. doi: 10.1177/20499361221099447. PMID: 35651526; PMCID: PMC9150225.	Incomplete data on RSV prevalence
343	Wrotek A, Kobiałka M, Grochowski B, Kamińska I, Pędziwiatr K, Skoczek-Wojciechowska A, Godek M, Jackowska T. Respiratory Complications in Children Hospitalized with Respiratory Syncytial Virus Infection. Adv Exp Med Biol. 2020;1279:113-120. doi: 10.1007/5584_2020_530. PMID: 32314316.	Inappropriate population.
344	Xu W, Guo L, Dong X, Li X, Zhou P, Ni Q, Zhou X, Wagner AL, Li L. Detection of Viruses and Mycoplasma pneumoniae in Hospitalized Patients with Severe Acute Respiratory Infection in Northern China, 2015-2016. Jpn J Infect Dis. 2018 Mar 22;71(2):134-139. doi: 10.7883/yoken.JJID.2017.412. Epub 2018 Feb 28. PMID: 29491245.	Inappropriate population.
345	Yan XL, Li YN, Tang YJ, Xie ZP, Gao HC, Yang XM, Li YM, Liu LJ, Duan ZJ. Clinical characteristics and viral load of respiratory syncytial virus and human metapneumovirus in children hospitaled for acute lower respiratory tract infection. J Med Virol. 2017 Apr;89(4):589-597. doi: 10.1002/jmv.24687. Epub 2016 Sep 28. PMID: 27632796; PMCID: PMC7166468.	Period of study before 2015.
346	Yang Q, Xiao X, Gu X, Liang D, Cao T, Mou J, Huang C, Chen L, Liu J. Surveillance of common respiratory infections during the COVID-19 pandemic demonstrates the preventive efficacy of non-pharmaceutical interventions. Int J Infect Dis. 2021 Apr;105:442-447. doi: 10.1016/j.ijid.2021.02.027. Epub 2021 Feb 11. PMID: 33582375; PMCID: PMC7877810.	Inappropriate population and seasonality not reported.
347	Yao T, Ai H, Yu X, Sun H, Wu S, Zhang X. Aetiological and epidemic analysis of 22,990 children with acute respiratory infection. Med J Wuhan Univ. 2016;37(6):1018, 1022, and 1030. Available from: www.scopus.com	Language not in English.
348	Yao Y, Li A, Song W. Epidemiology of pathogens causing acute respiratory infections in children in Beijing during 2016 to 2018. Chin J Microbiol Immunol. 2019;39(2):88-93. Available from: www.scopus.com	Language not in English.
349	Ye S, Wang T. Laboratory epidemiology of respiratory viruses in a large children's hospital: A STROBE-compliant article. Medicine (Baltimore). 2018 Jul;97(30):e11385. doi: 10.1097/MD.0000000000011385. PMID: 30045260; PMCID: PMC6078760.	Inappropriate population because the data were collected from

		records rather than directly from patients.
350	Ye Q, Wang D. Epidemiological changes of common respiratory viruses in children during the COVID-19 pandemic. J Med Virol. 2022 May;94(5):1990-1997. doi: 10.1002/jmv.27570. Epub 2022 Jan 11. PMID: 34981839; PMCID: PMC9015628.	Inappropriate population. The information is unclear regarding whether the population consists of inpatients or outpatients.
351	Yen CY, Wu WT, Chang CY, Wong YC, Lai CC, Chan YJ, Wu KG, Hung MC. Viral etiologies of acute respiratory tract infections among hospitalized children - A comparison between single and multiple viral infections. J Microbiol Immunol Infect. 2019 Dec;52(6):902-910. doi: 10.1016/j.jmii.2019.08.013. Epub 2019 Sep 30. PMID: 31607575; PMCID: PMC7105047.	Prevalence not reported by RSV season individually.
352	Yeoh DK, Foley DA, Minney-Smith CA, Martin AC, Mace AO, Sikazwe CT, Le H, Levy A, Blyth CC, Moore HC. Impact of Coronavirus Disease 2019 Public Health Measures on Detections of Influenza and Respiratory Syncytial Virus in Children During the 2020 Australian Winter. Clin Infect Dis. 2021 Jun 15;72(12):2199-2202. doi: 10.1093/cid/ciaa1475. PMID: 32986804; PMCID: PMC7543326.	Incomplete data on RSV prevalence
353	Yilmaz H, Irvem A, Guner AE, Kazezoglu C, Kocatas A. "Investigation of respiratory tract coinfections in Coronavirus disease 2019 infected and suspected cases." North Clin Istanb. 2022 Oct 20;9(5):421-428. doi: 10.14744/nci.2022.82608. PMID: 36447585; PMCID: PMC9677056.	Inappropriate population and RSV prevalence not reported.
354	Yon DK, Min CY, Ha EK, Jee HM, Jung YH, Lee KS, Sheen YH, Han MY. Clinical characteristics and genetic variation in respiratory syncytial virus isolated from infants hospitalized due to acute bronchiolitis in Korea during winter season 2016-2017. Allergy Asthma Respir Dis. 2018;6(2):110-115. doi: 10.4168/aard.2018.6.2.110.	Language not in English.
355	Yoon Y, Jung G, Ri S, Choung JT, Yoo Y. Clinical characteristics of lower respiratory tract infection in low birth weight children. Allergy Asthma & Respiratory Disease. 2018 Jul;6(4):211-218. DOI: 10.4168/aard.2018.6.4.211.	Language not in English.
356	Yorsaeng R, Suntronwong N, Thongpan I, Chuchaona W, Lestari FB, Pasittungkul S, Puenpa J, Atsawawaranunt K, Sharma C, Sudhinaraset N, Mungaomklang A, Kitphati R, Wanlapakorn N, Poovorawan Y. The impact of COVID-19 and control measures on public health in Thailand, 2020. PeerJ. 2022 Feb 16;10:e12960. doi: 10.7717/peerj.12960. PMID: 35190788; PMCID: PMC8857899.	Inappropriate population and RSV

		prevalence not reported.
357	Yuan H, Yeung A, Yang W. Interactions among common non-SARS-CoV-2 respiratory viruses and influence of the COVID-19 pandemic on their circulation in New York City. Influenza Other Respir Viruses. 2022 Jul;16(4):653-661. doi: 10.1111/irv.12976. Epub 2022 Mar 12. PMID: 35278037; PMCID: PMC9111828.	Inappropriate population and RSV prevalence not reported.
358	Yum S, Hong K, Sohn S, Kim J, Chun BC. Trends in Viral Respiratory Infections During COVID-19 Pandemic, South Korea. Emerg Infect Dis. 2021;27(6):1685-1688. doi: 10.3201/eid2706.210135. PMID: 34013875; PMCID: PMC8153859.	Inappropriate population and RSV prevalence not reported.
359	Yun KW, Wallihan R, Desai A, Alter S, Ambroggio L, Cohen DM, El-Assal O, Marzec S, Florin TA, Keaton M, Shah SS, Ruddy RM, Sharpe S, Leber AL, Everhart K, Mejias A, Ramilo O; Children's Hospitals Initiative for Research in Pneumonia. Clinical Characteristics and Etiology of Community-acquired Pneumonia in US Children, 2015-2018. Pediatr Infect Dis J. 2022 May 1;41(5):381-387. doi: 10.1097/INF.0000000000003475. PMID: 35143427.	Prevalence not reported by RSV season individually.
360	Zama D, Leardini D, Biscardi L, Corsini I, Pierantoni L, Andreozzi L, Lanari M. "Activity of a Pediatric Emergency Department of a Tertiary Center in Bologna, Italy, during SARS-CoV-2 Pandemic." Pediatr Rep. 2022 Aug 30;14(3):366-374. doi: 10.3390/pediatric14030043. PMID: 36136082; PMCID: PMC9505070.	Incomplete data on RSV prevalence
361	Zar HJ, Nduru P, Stadler JAM, et al. Early-life respiratory syncytial virus lower respiratory tract infection in a South African birth cohort: epidemiology and effect on lung health [published correction appears in Lancet Glob Health. 2022 May;10(5):e626]. Lancet Glob Health. 2020;8(10):e1316-e1325. doi: 10.1016/S2214-109X(20)30251-5.	Inappropriate study design. Editorial letter.
362	Zeytinli UO, Durmuslu AO, Aslan FG, Dincer SD, Eker P, Aksaray S. "Distribution of respiratory viruses: Evaluation of multiplex PCR results of 3074 patients." North Clin Istanb. 2022 Oct 26;9(5):501-504. doi: 10.14744/nci.2021.28034. PMID: 36447578; PMCID: PMC9677051.	Inappropriate population and RSV prevalence not reported.
363	Zhang H, Zhu A, Gao GF, Li Z. Epidemiological and Clinical Characteristics of Respiratory Syncytial Virus Infections in Children Aged <5 Years in China, from 2014−2018. Vaccines (Basel). 2022 May 20;10(5):810. doi: 10.3390/vaccines10050810. PMID: 35632566; PMCID: PMC9147122.	Incomplete data on RSV prevalence

364	Zhang M, Gao J, Guo Q, Zhang X, Zhang W. "Changes of respiratory syncytial virus infection in children before and after the COVID-19 pandemic in Henan, China." J Infect. 2023 Feb;86(2):154-225. doi: 10.1016/j.jinf.2022.12.011. Epub 2022 Dec 15. PMID: 36528226; PMCID: PMC9751002.	Incomplete data on RSV prevalence
365	Zhang Y, Qiao L, Yao J, Yu N, Mu X, Huang S, Hu B, Li W, Qiu F, Zeng F, Chen C, Zhou Y, Zhang B, Cai T, Wang W, Wu X, Zhou Y, Wang G, Situ B, Lan S, Li N, Li X, Li Z, Li X, Wang C, Yang C, Feng P, Wang H, Zhu S, Xiong Y, Luo M, Shen W, Hu X, Zheng L. Epidemiological and clinical characteristics of respiratory viruses in 4403 pediatric patients from multiple hospitals in Guangdong, China. BMC Pediatr. 2021 Jun 17;21(1):284. doi: 10.1186/s12887-021-02759-0. PMID: 34140022; PMCID: PMC8212487.	Inappropriate population.
366	Zhang YX, Shi T, Su QR, Deng JK. [Clinical characteristics and related factors of human respiratory syncytial viruses infection in premature infants within 2 years after birth in Shenzhen Children's Hospital]. Zhonghua Yi Xue Za Zhi. 2021 Sep 28;101(36):2873-2877. Chinese. doi: 10.3760/cma.j.cn112137-20210226-00505. PMID: 34587727.	Language not in English.
367	Zhu Y, Li W, Yang B, Qian R, Wu F, He X, Zhu Q, Liu J, Ni Y, Wang J, Mao S. Epidemiological and virological characteristics of respiratory tract infections in children during COVID-19 outbreak. BMC Pediatr. 2021 Apr 22;21(1):195. doi: 10.1186/s12887-021-02654-8. PMID: 33888063; PMCID: PMC8060686.	Inappropriate population.
**No.**	**Studies excluded from the systematic review via hand-searching.**	**Reasons for excluding**
1	Adams G, et al. The 2022 RSV surge was driven by multiple viral lineages. medRxiv [Preprint]. 2023 Jan 5:2023.01.04.23284195. doi: 10.1101/2023.01.04.23284195. Update in: N Engl J Med. 2023 Feb 22;: PMID: 36656774; PMCID: PMC9844019.	Inappropriate study due to not being peer-reviewed.
2	Alaib H, Algariri N, Ahmed H, Bebars A, Alamri F, Durmush R, Ayaz M, Hamadelnil W, Alboriaki B, Altamimi B, Alalyani M, Aljasser DS, Aboud M. Frequency and Seasonal Variations of Viruses Causing Respiratory Tract Infections in Children Pre- and Post-COVID-19 Pandemic in Riyadh (2017-2022). Cureus. 2023 Jan 6;15(1):e33467. doi: 10.7759/cureus.33467. PMID: 36628396; PMCID: PMC9823271.	Prevalence not reported by RSV season individually
3	Alqahtani MH, Alqahtani MF, Asiri M, Alghamdi S, Alshagawi Z, Alzahrani S. Bronchiolitis in Infants; Five Years' Experience of a Teaching Hospital. Infect Drug Resist. 2023 Aug 28;16:5647-5664. doi: 10.2147/IDR.S385615. PMID: 37662975; PMCID: PMC10473431.	Prevalence not reported by RSV season individually
4	Alrayes T, Wait A, Spencer P, Merolla DM, Lampe K, Salimnia H, Kannikeswaran N. Features of an Atypical RSV Surge During the COVID-19 Pandemic. Clin Pediatr (Phila). 2023 May;62(4):265-268. doi: 10.1177/00099228221124677. Epub 2022 Oct 1. PMID: 36189937; PMCID: PMC9527150.	Duplicate with previous search via databases and registers
5	Amarin JZ, et al. Clinical characteristics and outcomes of children with single or co-detected rhinovirus-associated acute respiratory infection in Middle Tennessee. BMC Infect Dis. 2023 Mar 7;23(1):136. doi: 10.1186/s12879-023-08084-4. PMID: 36882755; PMCID: PMC9990557.	Inappropriate population

6	Bandeira T, Carmo M, Lopes H, Gomes C, Martins M, Guzman C, Bangert M, Rodrigues F, Januário G, Tomé T, Azevedo I. Burden and severity of children's hospitalizations by respiratory syncytial virus in Portugal, 2015-2018. Influenza Other Respir Viruses. 2023 Jan;17(1):e13066. doi: 10.1111/irv.13066. Epub 2022 Nov 14. PMID: 36377322; PMCID: PMC9835409.	Diagnostic technique not reported
7	Bernet Sánchez A, et al. Clinical relevance of viral codetection in infants with respiratory syncytial virus bronchiolitis. Enferm Infecc Microbiol Clin (Engl Ed). 2023 Jul 17:S2529-993X(23)00195-8. doi: 10.1016/j.eimce.2023.07.001. Epub ahead of print. PMID: 37468350.	Incomplete data on RSV prevalence
8	Bögli J, Güsewell S, Strässle R, Kahlert CR, Albrich WC. Pediatric hospital admissions, case severity, and length of hospital stay during the first 18 months of the COVID-19 pandemic in a tertiary children's hospital in Switzerland. Infection. 2023 Apr;51(2):439-446. doi: 10.1007/s15010-022-01911-x. Epub 2022 Sep 5. PMID: 36065045; PMCID: PMC9444086.	Duplicate with previous search via databases and registers
9	Brady M, Duffy R, Domegan L, Salmon A, Maharjan B, O'Broin C, Bennett C, Christle J, Connell J, Feeney L, Nurdin N, Mallon P, Doran P, McNamara R, O'Grady S, McDermott S, Petty-Saphon N, O'Donnell J. Establishing severe acute respiratory infection (SARI) surveillance in a sentinel hospital, Ireland, 2021 to 2022. Euro Surveill. 2023 Jun;28(23):2200740. doi: 10.2807/1560-7917.ES.2023.28.23.2200740. PMID: 37289427; PMCID: PMC10318943.	Inappropriate population
10	Buchholz U, Lehfeld AS, Tolksdorf K, Cai W, Reiche J, Biere B, Dürrwald R, Buda S. Respiratory infections in children and adolescents in Germany during the COVID-19 pandemic. J Health Monit. 2023 Jun 14;8(2):20-38. doi: 10.25646/11437. PMID: 37408711; PMCID: PMC10318561.	Prevalence not reported by RSV season individually
11	Casalegno JS, et al. Application of a forecasting model to mitigate the consequences of unexpected RSV surge: Experience from the post-COVID-19 2021/22 winter season in a major metropolitan centre, Lyon, France. J Glob Health. 2023 Feb 3;13:04007. doi: 10.7189/jogh.13.04007. PMID: 36757127; PMCID: PMC9893715.	Incomplete data on RSV prevalence
12	Castagno E, Raffaldi I, Del Monte F, Garazzino S, Bondone C. New epidemiological trends of respiratory syncytial virus bronchiolitis during COVID-19 pandemic. World J Pediatr. 2023 May;19(5):502-504. doi: 10.1007/s12519-022-00623-4. Epub 2022 Sep 26. PMID: 36163542; PMCID: PMC9512956.	Duplicate with previous search via databases and registers
13	Chaiut W, Sapbamrer R, Dacha S, Sudjaritruk T, Parwati I, Sumarpo A, Malasao R. Characteristics of Respiratory Syncytial Virus Infection in Hospitalized Children Before and During the COVID-19 Pandemic in Thailand. J Prev Med Public Health. 2023 May;56(3):212-220. doi: 10.3961/jpmph.23.019. Epub 2023 Mar 23. PMID: 37287198; PMCID: PMC10248108.	Incomplete data on RSV prevalence
14	Chen D, Cao L, Li W. Etiological and clinical characteristics of severe pneumonia in pediatric intensive care unit (PICU). BMC Pediatr. 2023 Jul 15;23(1):362. doi: 10.1186/s12887-023-04175-y. PMID: 37454044; PMCID: PMC10349420.	Prevalence not reported by RSV season individually
15	Chi H, Chung CH. Respiratory Syncytial Virus Outbreak in Infants and Young Children during COVID-19 Pandemic in Taiwan. Children (Basel). 2023 Mar 28;10(4):629. doi: 10.3390/children10040629. PMID: 37189878; PMCID: PMC10137034.	Diagnostic technique not reported

16	Ciofi Degli Atti M, Rizzo C, D'Amore C, Ravà L, Reale A, Barbieri MA, Bernaschi P, Russo C, Villani A, Perno CF, Raponi M. Acute respiratory infection emergency access in a tertiary care children hospital in Italy, prior and after the SARS-CoV-2 emergence. Influenza Other Respir Viruses. 2023 Mar 20;17(3):e13102. doi: 10.1111/irv.13102. PMID: 36950039; PMCID: PMC10026100.	Incomplete data on RSV prevalence
17	Cocchio S, Prandi GM, Furlan P, Venturato G, Saia M, Marcon T, Tremolada G, Baldo V. Respiratory Syncytial Virus in Veneto Region: Analysis of Hospital Discharge Records from 2007 to 2021. Int J Environ Res Public Health. 2023 Mar 4;20(5):4565. doi: 10.3390/ijerph20054565. PMID: 36901576; PMCID: PMC10002215.	Diagnostic technique not reported
18	Costa VGD, Gomes AJC, Bittar C, Geraldini DB, Previdelli da Conceição PJ, Cabral ÁS, Carvalho T, Biselli JM, Provazzi PJS, Campos GRF, Sanches PRDS, Costa PI, Nogueira ML, Araujo JP Jr, Spilki FR, Calmon MF, Rahal P. Burden of Influenza and Respiratory Syncytial Viruses in Suspected COVID-19 Patients: A Cross-Sectional and Meta-Analysis Study. Viruses. 2023 Mar 1;15(3):665. doi: 10.3390/v15030665. PMID: 36992374; PMCID: PMC10055802.	Inappropriate population
19	De Maio F, Fiori B, Bianco DM, Sanguinetti M, Sali M. Respiratory viruses in the pre and post-pandemic periods in an Italian tertiary hospital. Immun Inflamm Dis. 2023 Aug;11(8):e909. doi: 10.1002/iid3.909. PMID: 37647421; PMCID: PMC10427785.	Inappropriate population
20	Dervaux B, Van Berleere M, Lenne X, Wyckaert M, Dubos F. Impact of RSV test positivity, patient characteristics, and treatment characteristics on the cost of hospitalization for acute bronchiolitis in a French university medical center (2010-2015). Front Pediatr. 2023 Jul 14;11:1126229. doi: 10.3389/fped.2023.1126229. PMID: 37528879; PMCID: PMC10390249.	Prevalence not reported by RSV season individually
21	Divarathna MVM, Rafeek RAM, Morel AJ, Aththanayake C, Noordeen F. Epidemiology and risk factors of respiratory syncytial virus associated acute respiratory tract infection in hospitalized children younger than 5 years from Sri Lanka. Front Microbiol. 2023 Jun 26;14:1173842. doi: 10.3389/fmicb.2023.1173842. PMID: 37434712; PMCID: PMC10330818.	Prevalence not reported by RSV season individually
22	Englund JA, Cohen RA, Bianco V, Domachowske JB, Langley JM, Madhi SA, Zaman K, Bueso A, Ceballos A, Cousin L, Gandhi S, Gruselle O, Jose L, Klein NP, Koen A, Puthanakit T, Shi M, Silas P, Tangsathapornpong A, Teeratakulpisarn J, Vesikari T, Haars G, Leach A, Stoszek SK, Dieussaert I. Evaluation of Clinical Case Definitions for Respiratory Syncytial Virus Lower Respiratory Tract Infection in Young Children. J Pediatric Infect Dis Soc. 2023 May 31;12(5):273-281. doi: 10.1093/jpids/piad028. PMID: 37142551; PMCID: PMC10231393.	Inappropriate population
23	Esparza-Miranda LA, Juárez-Tobías S, Muñoz-Escalante JC, Oliva-Jara UA, Cadena-Mota S, Wong-Chew RM, Noyola DE. Clinical and Epidemiologic Characteristics of Infants Hospitalized with Respiratory Syncytial Virus Infection During the 2022-2023 Season in Mexico. Pediatr Infect Dis J. 2023 Oct 1;42(10):e382-e384. doi: 10.1097/INF.0000000000004013. Epub 2023 Jun 22. PMID: 37345924.	RSV-season after 2021/22
24	Fan Y, Li D, Wang P, Ren L, Chen X. Case-control study of relationship of infection by respiratory viruses with acute otitis media in Chinese children. Heliyon. 2023 Mar 11;9(3):e14422. doi: 10.1016/j.heliyon.2023.e14422. PMID: 36967868; PMCID: PMC10036650.	Inappropriate population
25	Feiler MO, Yucel R, Liu Z, Caserta M, Lawrence BP, Pason CH, Hardy DJ, Thevenet-Morrison K, Dozier A, Jusko TA. Trends and Non-Clinical Predictors of Respiratory Syncytial Virus (RSV) and Influenza Diagnosis in an Urban Pediatric Population. Int J Pediatr Res. 2023;9(1):112. doi: 10.23937/2469-5769/1510112. Epub 2023 Mar 6. PMID: 37124477; PMCID: PMC10139760.	Inappropriate population

26	Fontes V, Ferreira H, Ribeiro M, Pinheiro A, Maramaldo C, Pereira E, Batista L, Júnior A, Lobato L, Silva F, Sousa L, Lima W, Lima C, Soczek S, Carvalho R, Santos M, Fernandes E, Sousa E, Neto L. High Incidence of Respiratory Syncytial Virus in Children with Community-Acquired Pneumonia from a City in the Brazilian Pre-Amazon Region. Viruses. 2023 May 31;15(6):1306. doi: 10.3390/v15061306. PMID: 37376604; PMCID: PMC10305439.	Incomplete data on RSV prevalence
27	Ghrieb Z, et al. Impact of the COVID-19 pandemic on antiviral drug development for other community-acquired respiratory viruses' infections. Therapie. 2023 May-Jun;78(3):241-245. doi: 10.1016/j.therap.2022.07.010. Epub 2022 Aug 1. PMID: 36030128; PMCID: PMC9341168.	Inappropriate population and RSV prevalence not reported
28	Goya S, Lucion MF, Shilts MH, Juárez MDV, Gentile A, Mistchenko AS, Viegas M, Das SR. Evolutionary dynamics of respiratory syncytial virus in Buenos Aires: Viral diversity, migration, and subgroup replacement. Virus Evol. 2023 Jan 25;9(1):vead006. doi: 10.1093/ve/vead006. PMID: 36880065; PMCID: PMC9985318.	Prevalence not reported by RSV season individually
29	Gsenger J, Bruckner T, Ihling CM, Rehbein RM, Schnee SV, Hoos J, Manuel B, Pfeil J, Schnitzler P, Tabatabai J. RSV-CLASS -Clinical Assessment Severity Score: An easy-to-use clinical disease severity score for respiratory syncytial virus infection in hospitalized children. J Med Virol. 2023 Feb;95(2):e28541. doi: 10.1002/jmv.28541. PMID: 36727642.	Prevalence not reported by RSV season individually
30	Habbous S, Hota S, Allen VG, Henry M, Hellsten E. Changes in hospitalizations and emergency department respiratory viral diagnosis trends before and during the COVID-19 pandemic in Ontario, Canada. PLoS One. 2023 Jun 16;18(6):e0287395. doi: 10.1371/journal.pone.0287395. PMID: 37327212; PMCID: PMC10275476.	Incomplete data on RSV prevalence
31	Hauch R, Hinrichs M, Ruhwald R, Schrum J, Rutkowski S, Woessmann W, Winkler B. Impact of COVID-19 Related Restrictions on Infections in Children with Cancer or after Hematopoietic SCT. Klin Padiatr. 2023 May;235(3):159-166. English. doi: 10.1055/a-2000-5388. Epub 2023 Feb 27. Erratum in: Klin Padiatr. 2023 Apr 12;: PMID: 36848939.	Inappropriate population
32	Hönemann M, Thiem S, Bergs S, Berthold T, Propach C, Siekmeyer M, Frille A, Wallborn T, Maier M, Pietsch C. In-Depth Analysis of the Re-Emergence of Respiratory Syncytial Virus at a Tertiary Care Hospital in Germany in the Summer of 2021 after the Alleviation of Non-Pharmaceutical Interventions Due to the SARS-CoV-2 Pandemic. Viruses. 2023 Mar 29;15(4):877. doi: 10.3390/v15040877. PMID: 37112857; PMCID: PMC10144477.	Incomplete data on RSV prevalence
33	Huang K, Li HY, Chen MH, Zhu TT, Zhang XY, Lyu FF, Lin L, Su MS, Dong L. [Analysis of the clinical features and the risk factors of severe human metapneumovirus-associated community-acquired pneumonia in children]. Zhonghua Er Ke Za Zhi. 2023 Apr 2;61(4):322-327. Chinese. doi: 10.3760/cma.j.cn112140-20221231-01079. PMID: 37011977.	Language not in English
34	Illan Montero J, Berger A, Levy J, Busson L, Hainaut M, Goetghebuer T. Retrospective comparison of respiratory syncytial virus and metapneumovirus clinical presentation in hospitalized children. Pediatr Pulmonol. 2023 Jan;58(1):222-229. doi: 10.1002/ppul.26188. Epub 2022 Oct 20. PMID: 36202614.	Period of study before 2015
35	Jarju S, Senghore E, Brotherton H, Affleck L, Saidykhan A, Jallow S, Krubally E, Sinjanka E, Ndene MN, Bajo F, Sanyang MM, Saidy B, Bah A, Mohammed NI, Forrest K, Clarke E, Dalessandro U, Sesay AK, Usuf E, Cerami C, Roca A, Kampmann B, de Silva TI. Circulation of respiratory viruses during the COVID-19 pandemic in The Gambia. Gates Open Res. 2023 Mar 27;6:148. doi: 10.12688/gatesopenres.14155.3. PMID: 36726685; PMCID: PMC9883272.	Inappropriate population

36	Karbuz A, İşançlı DK, Kılıçaslan Ö, Kırmacı Ç, Emre I, Beşel L, Barış A, Tutak GA, Aktaş E, Arat Z. The effects of measures taken during the COVID-19 pandemic on the seasonal dynamics of respiratory viruses in children. Turk J Pediatr. 2023;65(4):592-602. doi: 10.24953/turkjped.2023.154. PMID: 37661674.	Incomplete data on RSV prevalence
37	Kelly ME, et al. Etiologies of influenza-like illness and severe acute respiratory infections in Tanzania, 2017-2019. PLOS Glob Public Health. 2023 Feb 9;3(2):e0000906. doi: 10.1371/journal.pgph.0000906. PMID: 36962965; PMCID: PMC10021583.	Inappropriate population and RSV prevalence not reported
38	Khasawneh AI, Himsawi NM, Abu-Raideh JA, Sammour A, Abu Safieh H, Obeidat A, Azab M, Tarifi AA, Al Khawaldeh A, Al-Momani H, Al Shboul S, Saleh T. Prevalence of SARS-COV-2 and other respiratory pathogens among a Jordanian subpopulation during Delta-to-Omicron transition: Winter 2021/2022. PLoS One. 2023 Mar 30;18(3):e0283804. doi: 10.1371/journal.pone.0283804. PMID: 36996148; PMCID: PMC10062597.	Inappropriate population
39	Kırca F, Aydoğan S, Gozalan A, Güler E, Uyan Erten AZ, Özşen Uygur AS, Doğan A, Dinc B. Impact of non-pharmaceutical interventions on circulating respiratory viruses during the COVID-19 pandemic in Turkey. Ann Saudi Med. 2023 May-Jun;43(3):143-153. doi: 10.5144/0256-4947.2023.143. Epub 2023 Jun 1. PMID: 37270680; PMCID: PMC10317495.	Inappropriate population
40	Kışlal FM, Hanilçe Y, Altaş B, Büyükbaşaran ZE, Güven D. The disappearance of respiratory syncytial virus and influenza viruses in children during the second year of the COVID-19 pandemic - are non-pharmaceutical interventions as effective as vaccines? Eur Rev Med Pharmacol Sci. 2023 Apr;27(8):3777-3783. doi: 10.26355/eurrev_202304_32178. PMID: 37140326.	Inappropriate population
41	Kitagawa D, Kitano T, Furumori M, Suzuki S, Shintani Y, Suzuki Y, Nakano A, Nakano R, Nishiyama A, Yoshida S, Yano H, Maeda K, Nakamura F. Epidemiology of respiratory tract infections using multiplex PCR in a Japanese acute care hospital during the COVID19 pandemic. Heliyon. 2023 Mar 11;9(3):e14424. doi: 10.1016/j.heliyon.2023.e14424. PMID: 36919088; PMCID: PMC10007720.	Inappropriate population
42	Klivleyeva N, et al. Spread of Pathogens Causing Respiratory Viral Diseases Before and During CoVID-19 Pandemic in Kazakhstan. Indian J Microbiol. 2023 Mar;63(1):129-138. doi: 10.1007/s12088-023-01064-x. Epub 2023 Feb 28. PMID: 37168842; PMCID: PMC9972336.	Inappropriate population
43	Kohns Vasconcelos M, Meyer Sauteur PM, Keitel K, Santoro R, Egli A, Coslovsky M, Seiler M, Lurà M, Köhler H, Loevy N, Kahlert CR, Heininger U, Van den Anker J, Bielicki JA. Detection of mostly viral pathogens and high proportion of antibiotic treatment initiation in hospitalised children with community-acquired pneumonia in Switzerland - baseline findings from the first two years of the KIDS-STEP trial. Swiss Med Wkly. 2023 Feb 20;153:40040. doi: 10.57187/smw.2023.40040. PMID: 36800889.	Prevalence not reported by RSV season individually
44	Krumkamp R, Kohsar M, Nolte K, Hogan B, Eibach D, Jaeger A, Akenten CW, Drosten C, Boahen KG, Sarpong N, Eckerle I, Binger T, Owusu-Dabo E, May J, Kreuels B. Pathogens associated with hospitalization due to acute lower respiratory tract infections in children in rural Ghana: a case-control study. Sci Rep. 2023 Feb 10;13(1):2443. doi: 10.1038/s41598-023-29410-5. PMID: 36765075; PMCID: PMC9916495.	Period of study before 2015
45	Kuitunen I, et al. The end of COVID-19 restrictions for children in autumn 2021 was followed by an unusually early, high-peak respiratory syncytial virus epidemic. Acta Paediatr. 2023 Jan;112(1):146-148. doi: 10.1111/apa.16511. Epub 2022 Aug 13. PMID: 35933711; PMCID: PMC9538114.	Incomplete data on RSV prevalence

46	Lai A, Bergna A, Fabiano V, Ventura CD, Fumagalli G, Mari A, Loiodice M, Zuccotti GV, Zehender G. Epidemiology and molecular analyses of respiratory syncytial virus in the 2021-2022 season in northern Italy. Front Microbiol. 2024 Jan 4;14:1327239. doi: 10.3389/fmicb.2023.1327239. PMID: 38239726; PMCID: PMC10794773.	Incomplete data on RSV prevalence/inappropriate population
47	Li M, Wang J, Yao Z, Liao H, Su S, Yang X, Xie M, Zheng Y. Metagenomic-based pathogen surveillance for children with severe pneumonia in pediatric intensive care unit. Front Public Health. 2023 Jun 15;11:1177069. doi: 10.3389/fpubh.2023.1177069. PMID: 37397737; PMCID: PMC10309210.	Inappropriate population
48	Lin SC, Wang HC, Lin WC, Kuo YT, Hsu YH, Tsai YT, Lu SC, Wang YH, Chen SY. Viral Pneumonia during the COVID-19 Pandemic, 2019-2021 Evoking Needs for SARS-CoV-2 and Additional Vaccinations. Vaccines (Basel). 2023 Apr 27;11(5):905. doi: 10.3390/vaccines11050905. PMID: 37243009; PMCID: PMC10220939.	Prevalence not reported by RSV season individually
49	Liu YN, Zhang YF, Xu Q, Qiu Y, Lu QB, Wang T, Zhang XA, Lin SH, Lv CL, Jiang BG, Li H, Li ZJ, Gao GF, Yang WZ, Hay SI, Wang LP, Fang LQ, Liu W; Chinese Center for Disease Control and Prevention Etiology Surveillance Study Team of Acute Respiratory Infections. Infection and co-infection patterns of community-acquired pneumonia in patients of different ages in China from 2009 to 2020: a national surveillance study. Lancet Microbe. 2023 May;4(5):e330-e339. doi: 10.1016/S2666-5247(23)00031-9. Epub 2023 Mar 28. PMID: 37001538.	Prevalence not reported by RSV season individually
50	Mai W, Ren Y, Tian X, Al-Mahdi AY, Peng R, An J, Lin Q, Hu X, Wang G, Sun C, Lu Z, Du J, Xiao M, Yin F. Comparison of common human respiratory pathogens among hospitalized children aged ≤ 6 years in Hainan Island, China, during spring and early summer in 2019-2021. J Med Virol. 2023 Apr;95(4):e28692. doi: 10.1002/jmv.28692. PMID: 36946502.	Incomplete data on RSV prevalence
51	Malveste Ito CR, Moreira ALE, Silva PAND, Santos MO, Santos APD, Rézio GS, Brito PN, Rezende APC, Fonseca JG, Peixoto FAO, Wastowski IJ, Goes VM, Estrela MC, Souza PZ, Carneiro LC, Avelino MAG. Viral Coinfection of Children Hospitalized with Severe Acute Respiratory Infections during COVID-19 Pandemic. Biomedicines. 2023 May 9;11(5):1402. doi: 10.3390/biomedicines11051402. PMID: 37239073; PMCID: PMC10216660.	Inappropriate population
52	Martinón-Torres F, Carmo M, Platero L, Drago G, López-Belmonte JL, Bangert M, Díez-Domingo J. Clinical and economic hospital burden of acute respiratory infection (BARI) due to respiratory syncytial virus in Spanish children, 2015-2018. BMC Infect Dis. 2023 Jun 8;23(1):385. doi: 10.1186/s12879-023-08358-x. PMID: 37291530; PMCID: PMC10249572.	Incomplete data on RSV prevalence
53	Minney-Smith CA, Foley DA, Sikazwe CT, Levy A, Smith DW. The seasonality of respiratory syncytial virus in Western Australia prior to implementation of SARS-CoV-2 non-pharmaceutical interventions. Influenza Other Respir Viruses. 2023 Mar 9;17(3):e13117. doi: 10.1111/irv.13117. PMID: 36970572; PMCID: PMC10035409.	Incomplete data on RSV prevalence
54	Miyama T, et al. Exploring the threshold for the start of respiratory syncytial virus infection epidemic season using sentinel surveillance data in Japan. Front Public Health. 2023 Feb 3;11:1062726. doi: 10.3389/fpubh.2023.1062726. PMID: 36817928; PMCID: PMC9936060.	Incomplete data on RSV prevalence
55	Moscovich DP, Averbuch D, Kerem E, Cohen-Cymberknoh M, Berkun Y, Brooks R, Reiff S, Meir MB, Wolf D, Breuer O. Pediatric respiratory admissions and related viral infections during the COVID-19 pandemic. Pediatr Pulmonol. 2023 Jul;58(7):2076-2084. doi: 10.1002/ppul.26434. Epub 2023 Apr 25. PMID: 37097057.	Pooled into one RSV-prevalence are two or more RSV seasons.

56	Mossad SB. The perfect storm: An unseasonably early RSV annual epidemic, a severe annual flu epidemic, and a smoldering COVID-19 pandemic. Cleve Clin J Med. 2023 May 1;90(5):297-306. doi: 10.3949/ccjm.90a.23007. PMID: 37127335.	Inappropriate study design
57	Moumbeket Yifomnjou MH, Monamele GC, Njankouo-Ripa M, Fatawou Modiyinji A, Ngoupo PA, Boyomo O, Njouom R. Viral co-infection with human respiratory syncytial virus in suspected acute and severe respiratory tract infections during COVID-19 pandemic in Yaoundé, Cameroon, 2020-2021. Influenza Other Respir Viruses. 2023 Mar;17(3):e13131. doi: 10.1111/irv.13131. PMID: 36991539; PMCID: PMC10060445.	Inappropriate population
58	Moyes J, Tempia S, Walaza S, McMorrow ML, Treurnicht F, Wolter N, von Gottberg A, Kahn K, Cohen AL, Dawood H, Variava E, Cohen C. The burden of RSV-associated illness in children aged < 5 years, South Africa, 2011 to 2016. BMC Med. 2023 Apr 11;21(1):139. doi: 10.1186/s12916-023-02853-3. PMID: 37038125; PMCID: PMC10088270.	Incomplete data on RSV prevalence
59	Munkstrup C, Lomholt FK, Emborg HD, Møller KL, Krog JS, Trebbien R, Vestergaard LS. Early and intense epidemic of respiratory syncytial virus (RSV) in Denmark, August to December 2022. Euro Surveill. 2023 Jan;28(1):2200937. doi: 10.2807/1560-7917.ES.2023.28.1.2200937. PMID: 36695451; PMCID: PMC9817209.	Incomplete data on RSV prevalence
60	Nasrullah A, Gangu K, Garg I, Javed A, Shuja H, Chourasia P, Shekhar R, Sheikh AB. Trends in Hospitalization and Mortality for Influenza and Other Respiratory Viruses during the COVID-19 Pandemic in the United States. Vaccines (Basel). 2023 Feb 10;11(2):412. doi: 10.3390/vaccines11020412. PMID: 36851289; PMCID: PMC9966237.	Inappropriate population
61	Nduaguba SO, Tran PT, Choi Y, Winterstein AG. Respiratory syncytial virus reinfections among infants and young children in the United States, 2011-2019. PLoS One. 2023 Feb 16;18(2):e0281555. doi: 10.1371/journal.pone.0281555. PMID: 36795639; PMCID: PMC9934310.	Incomplete data on RSV prevalence
62	Nguyen E, Saw C, Morkos M, Abass F, Foley D, Bulsara M. Unusual local epidemic of paediatric respiratory syncytial virus during a time of global pandemic. J Paediatr Child Health. 2023 Mar;59(3):464-469. doi: 10.1111/jpc.16326. Epub 2023 Jan 10. PMID: 36625316.	Incomplete data on RSV prevalence
63	Nyawanda BO, Murunga N, Otieno NA, Bigogo G, Nyiro JU, Vodicka E, Bulterys M, Nokes DJ, Munywoki PK, Emukule GO. Estimates of the national burden of respiratory syncytial virus in Kenyan children aged under 5 years, 2010-2018. BMC Med. 2023 Mar 31;21(1):122. doi: 10.1186/s12916-023-02787-w. PMID: 37004034; PMCID: PMC10067313.	Incomplete data on RSV prevalence
64	Nygaard U, et al. Hospital admissions and need for mechanical ventilation in children with respiratory syncytial virus before and during the COVID-19 pandemic: a Danish nationwide cohort study. Lancet Child Adolesc Health. 2023 Mar;7(3):171-179. doi: 10.1016/S2352-4642(22)00371-6. Epub 2023 Jan 9. PMID: 36634692; PMCID: PMC9940917.	Incomplete data on RSV prevalence
65	Ohnishi T, Kang Y, Kawano Y, Kunikata T, Ichihashi K. An atypical surge in RSV infections among children in Saitama, Japan in 2021. IJID Reg. 2022 Dec 21;7:124-126. doi: 10.1016/j.ijregi.2022.12.006. PMID: 37009576; PMCID: PMC10063375.	Incomplete data on RSV prevalence

66	Okuyan O, Elgormus Y, Dumur S, Sayili U, Uzun H. New Generation of Systemic Inflammatory Markers for Respiratory Syncytial Virus Infection in Children. Viruses. 2023 May 25;15(6):1245. doi: 10.3390/v15061245. PMID: 37376545; PMCID: PMC10305612.	Prevalence not reported by RSV season individually
67	O'Neill GK, Taylor J, Kok J, Dwyer DE, Dilcher M, Hua H, Levy A, Smith D, Minney-Smith CA, Wood T, Jelley L, Huang QS, Trenholme A, McAuliffe G, Barr I, Sullivan SG. Circulation of influenza and other respiratory viruses during the COVID-19 pandemic in Australia and New Zealand, 2020-2021. Western Pac Surveill Response J. 2023 Jul 27;14(3):1-9. doi: 10.5365/wpsar.2023,14.3.948. PMID: 37946717; PMCID: PMC10630701.	Inappropriate population
68	Ono T, et al. Molecular Diversity of Human Respiratory Syncytial Virus before and during the COVID-19 Pandemic in Two Neighboring Japanese Cities. Microbiol Spectr. 2023 Aug 17;11(4):e0260622. doi: 10.1128/spectrum.02606-22. Epub 2023 Jul 6. PMID: 37409937; PMCID: PMC10433803.	Incomplete data on RSV prevalence
69	Osman S, Alaa Adeen A, Hetta O, Alsiraihi A, Bader M, Aloufi A, Abushouk A, Al-Hindi MY. Epidemiology and Risk Factor Analysis of Children with Bronchiolitis Admitted to the Intensive Care Unit at a Tertiary Care Center in Saudi Arabia. Children (Basel). 2023 Mar 30;10(4):646. doi: 10.3390/children10040646. PMID: 37189894; PMCID: PMC10136570.	Prevalence not reported by RSV season individually
70	Oster Y, et al. Viral and Bacterial Respiratory Pathogens during the COVID-19 Pandemic in Israel. Microorganisms. 2023 Jan 9;11(1):166. doi: 10.3390/microorganisms11010166. PMID: 36677458; PMCID: PMC9864990.	Incomplete data on RSV prevalence
71	Ouldali N, Deceuninck G, Lefebvre B, Gilca R, Quach C, Brousseau N, Tapiero B, De Wals P. Increase of invasive pneumococcal disease in children temporally associated with RSV outbreak in Quebec: a time-series analysis. Lancet Reg Health Am. 2023 Feb 15;19:100448. doi: 10.1016/j.lana.2023.100448. PMID: 36852331; PMCID: PMC9958468.	Inappropriate population
72	Papachristou E, Rokka C, Sotiriadou T, Maneka L, Vassilakis A, Sapounas S, Paraskevis D, Jahaj E, Kotanidou A, Lagiou P, Magiorkinis G. Low circulation of respiratory syncytial and influenza viruses during autumn-winter 2021 in the industrial workplace and long-term healthcare facilities in Athens, Greece. Front Med (Lausanne). 2023 Jan 9;9:1025147. doi: 10.3389/fmed.2022.1025147. PMID: 36698808; PMCID: PMC9869044.	Inappropriate population
73	Petrocelli PA, Cunsolo V, Melito M, Scuderi G, Testa R, Messina S, Tucci F, Sardone L, Colligiani D, Nardone M, Rapi S, Stenner E. Diagnosis of Respiratory Syncytial Virus (RSV) infection in children by Respiratory Panel utilized during the COVID-19 pandemic. Ann Ist Super Sanita. 2023 Jan-Mar;59(1):31-36. doi: 10.4415/ANN_23_01_05. PMID: 36974702.	Incomplete data on RSV prevalence
74	Pierangeli A, et al. Genetic diversity and its impact on disease severity in respiratory syncytial virus subtype-A and -B bronchiolitis before and after pandemic restrictions in Rome. J Infect. 2023 Oct;87(4):305-314. doi: 10.1016/j.jinf.2023.07.008. Epub 2023 Jul 24. PMID: 37495189.	Incomplete data on RSV prevalence
75	Posada MJG, Dajil HJC, Díaz APN, Castillo Vidal JDD, Barreto DJS, Sanchez MC, Coll HS, Mattar S. Not all respiratory infections were SARS-CoV-2 during the pandemic, analysis in a clinic on the Colombian Caribbean coast. J Infect Public Health. 2023 Sep;16(9):1403-1409. doi: 10.1016/j.jiph.2023.06.008. Epub 2023 Jun 16. PMID: 37480671; PMCID: PMC10270728.	Inappropriate population

76	Pury S, Álvarez MS, García Oro MC. Detección molecular de Mycoplasma pneumoniae en muestras respiratorias de niños hospitalizados. [Molecular detection of Mycoplasma pneumoniae in respiratory samples from hospitalized children]. Rev Fac Cien Med Univ Nac Cordoba. 2023 Mar 31;80(1):20-24. Spanish. doi: 10.31053/1853.0605.v80.n1.37514. PMID: 37018359; PMCID: PMC10142670.	Language not in English
77	Ramos N, Panzera Y, Frabasile S, Tomás G, Calleros L, Marandino A, Goñi N, Techera C, Grecco S, Fuques E, Coppola L, Ramas V, Morel MN, Mogdasy C, Chiparelli H, Arbiza J, Pérez R, Delfraro A. A multiplex-NGS approach to identifying respiratory RNA viruses during the COVID-19 pandemic. Arch Virol. 2023 Feb 14;168(3):87. doi: 10.1007/s00705-023-05717-6. PMID: 36786897; PMCID: PMC9926447.	Inappropriate population
78	Rao S, et al. Shifting Epidemiology and Severity of Respiratory Syncytial Virus in Children During the COVID-19 Pandemic. JAMA Pediatr. 2023 Jul 1;177(7):730-732. doi: 10.1001/jamapediatrics.2023.1088. PMID: 37184852; PMCID: PMC10186203.	Incomplete data on RSV prevalence
79	Ren L, et al. Epidemiological and clinical characteristics of respiratory syncytial virus and influenza infections in hospitalized children before and during the COVID-19 pandemic in Central China. Influenza Other Respir Viruses. 2023 Feb 2;17(2):e13103. doi: 10.1111/irv.13103. PMID: 36824393; PMCID: PMC9895987.	Incomplete data on RSV prevalence
80	Riepl A, Straßmayr L, Voitl P, Ehlmaier P, Voitl JJM, Langer K, Kuzio U, Mühl-Riegler A, Mühl B, Diesner-Treiber SC. The surge of RSV and other respiratory viruses among children during the second COVID-19 pandemic winter season. Front Pediatr. 2023 Feb 1;11:1112150. doi: 10.3389/fped.2023.1112150. PMID: 36816380; PMCID: PMC9929140.	Incomplete data on RSV prevalence
81	Shi T, Zhao X, Zhang X, Meng L, Li D, Liu X, Zheng H, Yu D, Wang T, Li R, Li J, Shen X, Ren X. Immediate and long-term changes in the epidemiology, infection spectrum, and clinical characteristics of viral and bacterial respiratory infections in Western China after the COVID-19 outbreak: a modeling study. Arch Virol. 2023 Mar 28;168(4):120. doi: 10.1007/s00705-023-05752-3. PMID: 36976267; PMCID: PMC10044131.	Incomplete data on RSV prevalence
82	Shichijo K, Fukura S, Takeuchi S, Tayama T, Ono A, Ichihara Y, Suga K, Sato H, Takechi A, Matsumoto S, Fujino S, Taniguchi T, Takahashi A, Watanabe T, Kondo S. A surge in respiratory syncytial virus infection-related hospitalizations associated with the COVID-19 pandemic: An observational study at pediatric emergency referral hospitals in Tokushima Prefecture. PLOS Glob Public Health. 2023 Jun 2;3(6):e0001974. doi: 10.1371/journal.pgph.0001974. PMID: 37267243; PMCID: PMC10237384.	Incomplete data on RSV prevalence
83	Smyrnaios A, Risnes K, Krokstad S, Nordbø SA, Heimdal I, Christensen A, Døllner H. The Contribution of Viruses and Bacteria to Childhood Community-acquired Pneumonia: 11-Year Observational Study From Norway. Pediatr Infect Dis J. 2023 Jun 1;42(6):456-460. doi: 10.1097/INF.0000000000003867. Epub 2023 Feb 24. PMID: 36795570.	Prevalence not reported by RSV season individually
84	Song J, et al. Circulation pattern and genetic variation of human respiratory syncytial virus in China during 2008-2021. J Med Virol. 2023 Mar;95(3):e28611. doi: 10.1002/jmv.28611. PMID: 36846911.	Inappropriate population and RSV prevalence not reported
85	Steponavičienė A, et al. Influenza and Respiratory Syncytial Virus Infections in Pediatric Patients during the COVID-19 Pandemic: A Single-Center Experience. Children (Basel). 2023 Jan 7;10(1):126. doi: 10.3390/children10010126. PMID: 36670676; PMCID: PMC9856748.	Incomplete data on RSV prevalence

86	Suzuki M, Hayakawa K, Asai Y, Terada M, Kitajima K, Tsuzuki S, Moriya A, Moriya K, Uchiyama-Nakamura F, Ohmagari N. Characteristics of hospitalized COVID-19 patients with other respiratory pathogens identified by rapid diagnostic test. J Infect Chemother. 2023 May;29(5):539-545. doi: 10.1016/j.jiac.2023.02.006. Epub 2023 Feb 20. PMID: 36813162; PMCID: PMC9939390.	Inappropriate population
87	Tabatabai J, Ihling CM, Manuel B, Rehbein RM, Schnee SV, Hoos J, Pfeil J, Grulich-Henn J, Schnitzler P. Viral Etiology and Clinical Characteristics of Acute Respiratory Tract Infections in Hospitalized Children in Southern Germany (2014-2018). Open Forum Infect Dis. 2023 Mar 1;10(3):ofad110. doi: 10.1093/ofid/ofad110. PMID: 36968956; PMCID: PMC10034757.	Incomplete data on RSV prevalence
88	Tang KM, Hametz P, Southern W. RSV causes more severe respiratory illness than influenza in admitted children under 2-years-old. Pediatr Pulmonol. 2023 Jun;58(6):1738-1745. doi: 10.1002/ppul.26394. Epub 2023 Apr 4. PMID: 37014143.	Prevalence not reported by RSV season individually
89	Tang X, et al. Comparison of the clinical features of human bocavirus and metapneumovirus lower respiratory tract infections in hospitalized children in Suzhou, China. Front Pediatr. 2023 Jan 10;10:1074484. doi: 10.3389/fped.2022.1074484. PMID: 36704137; PMCID: PMC9871608.	Incomplete data on RSV prevalence
90	Tiew WT, Chen YC, Hsiao HL, Chen CL, Chen CJ, Chiu CH. Impact of multiplex polymerase chain reaction syndromic panel on antibiotic use among hospitalized children with respiratory tract illness during COVID-19 pandemic. J Microbiol Immunol Infect. 2023 Aug;56(4):688-694. doi: 10.1016/j.jmii.2023.01.009. Epub 2023 Jan 16. PMID: 36681556; PMCID: PMC9841733.	Prevalence not reported by RSV season individually
91	Uwak I, Johnson N, Mustapha T, Rahman M, Tonpay T, Regan AK, Mendoza-Sanchez I. Quantifying changes in respiratory syncytial virus-associated hospitalizations among children in Texas during COVID-19 pandemic using records from 2006 to 2021. Front Pediatr. 2023 Mar 13;11:1124316. doi: 10.3389/fped.2023.1124316. PMID: 36994433; PMCID: PMC10040829.	Incomplete data on RSV prevalence
92	Vila J, Lera E, Peremiquel-Trillas P, Andrés C, Martínez L, Barceló I, Carsi A, Balcells J, Ángel Rodrigo-Pendás J, Soler-Palacín P, Rodrigo C, Antón A. Increased RSV-A Bronchiolitis Severity in RSV-Infected Children Admitted to a Reference Center in Catalonia (Spain) Between 2014 and 2018. J Pediatric Infect Dis Soc. 2023 Apr 18;12(3):180-183. doi: 10.1093/jpids/piad009. PMID: 36744919.	Incomplete data on RSV prevalence
93	Wadilo F, Feleke A, Gebre M, Mihret W, Seyoum T, Melaku K, Howe R, Mulu A, Mihret A. Viral etiologies of lower respiratory tract infections in children < 5 years of age in Addis Ababa, Ethiopia: a prospective case-control study. Virol J. 2023 Jul 23;20(1):163. doi: 10.1186/s12985-023-02131-x. PMID: 37481644; PMCID: PMC10363322.	Prevalence not reported by RSV season individually
94	Wildenbeest JG, Billard MN, Zuurbier RP, Korsten K, Langedijk AC, van de Ven PM, Snape MD, Drysdale SB, Pollard AJ, Robinson H, Heikkinen T, Cunningham S, O'Neill T, Rizkalla B, Dacosta-Urbieta A, Martinón-Torres F, van Houten MA, Bont LJ; RESCEU Investigators. The burden of respiratory syncytial virus in healthy term-born infants in Europe: a prospective birth cohort study. Lancet Respir Med. 2023 Apr;11(4):341-353. doi: 10.1016/S2213-2600(22)00414-3. Epub 2022 Nov 10. PMID: 36372082; PMCID: PMC9764871.	Incomplete data on RSV prevalence
95	Yamashita S, Ikegame S, Nakatomi K, Sakurai Y, Shuto H, Sato N, Mizoguchi Y, Uehara M, Nakashima N, Okamoto I, Koto H. Respiratory Virus Infections during the COVID-19 Pandemic Revealed by Multiplex PCR Testing in Japan. Microbiol Spectr. 2023 Feb 1;11(2):e0416222. doi: 10.1128/spectrum.04162-22. Epub ahead of print. PMID: 36723071; PMCID: PMC10100992.	Inappropriate population

96	Yan Y, et al. Prevalence, variation, and transmission patterns of human respiratory syncytial virus from pediatric patients in Hubei, China during 2020-2021. Virol Sin. 2023 Jun;38(3):363-372. doi: 10.1016/j.virs.2023.05.001. Epub 2023 May 3. PMID: 37146717; PMCID: PMC10311268.	Incomplete data on RSV prevalence
97	Zarur-Torralvo S, Stand-Niño I, Flórez-García V, Mendoza H, Viana-Cárdenas E. Viruses responsible for acute respiratory infections before (2016-2019) and during (2021) circulation of the SARS-CoV-2 virus in pediatric patients in a reference center at Barranquilla Colombia: A pattern analysis. J Med Virol. 2023 Jan;95(1):e28439. doi: 10.1002/jmv.28439. PMID: 36573419; PMCID: PMC9880681.	Prevalence not reported by RSV season individually
98	Zhang J, Yang T, Zou M, Wang L, Sai L. The epidemiological features of respiratory tract infection using the multiplex panels detection during COVID-19 pandemic in Shandong province, China. Sci Rep. 2023 Apr 18;13(1):6319. doi: 10.1038/s41598-023-33627-9. PMID: 37072619; PMCID: PMC10112310.	Inappropriate population
99	Zhu L, Luo T, Yuan Y, Yang S, Niu C, Gong T, Wang X, Xie X, Luo J, Liu E, Fu Z, Tian D. Epidemiological characteristics of respiratory viruses in hospitalized children during the COVID-19 pandemic in southwestern China. Front Cell Infect Microbiol. 2023 Apr 4;13:1142199. doi: 10.3389/fcimb.2023.1142199. PMID: 37153160; PMCID: PMC10157792.	Pooled into one RSV-prevalence are two or more RSV seasons.
